# Synthesis and Biological Evaluation of RBG Derivatives as Nrf2 Activators for the Treatment of Parkinson’s Disease

**DOI:** 10.3390/ijms27073326

**Published:** 2026-04-07

**Authors:** Wen-Qing Shi, Jia-Hua Zhuang, Qiu-Heng Zhang, Guo-Qiang Lin, Shaopeng Yu, Yao Chen, Jun-Huan Fu, Jiange Zhang, Shoujiao Peng, Gu-Zhou Chen, Wenbo Ye

**Affiliations:** 1 The Research Center of Chiral Drugs, Innovation Research Institute of Traditional Chinese Medicine, Shanghai University of Traditional Chinese Medicine, Shanghai 201203, China; s2099406681@163.com (W.-Q.S.); 22023635@shutcm.edu.cn (J.-H.Z.); zhangqh12300@163.com (Q.-H.Z.); lingq@sioc.ac.cn (G.-Q.L.); yushaopeng0405@163.com (S.Y.); chenyao768@163.com (Y.C.); 22024752@shutcm.edu.cn (J.-H.F.); jgzhang@shutcm.edu.cn (J.Z.); chenguzhou824619@shutcm.edu.cn (G.-Z.C.); 2 The Shanghai Institute of Organic Chemistry, University of Chinese Academy of Sciences, Shanghai 200032, China

**Keywords:** resibufogenin derivatives, Nrf2 activation, structural modification, neuroprotective effects, Parkinson’s disease

## Abstract

Nuclear factor erythroid 2-related factor 2 (Nrf2) is a master regulator of the cellular antioxidant response and a promising therapeutic target for Parkinson’s disease (PD). Resibufogenin (RBG), a bioactive bufadienolide from toad venom, has been identified as a potential Nrf2 agonist; however, its application is limited by cytotoxicity and poor drug-like properties. Herein, we report the rational design, synthesis, and biological evaluation of a series of RBG derivatives modified at the C3, C14–C15, and C17 positions. Systematic structure–activity relationship (SAR) studies identified **2-5c**, featuring a C3 2-chloroacryloyl group and a C17 pyrimidine substitution, as a potential Nrf2 activator (EC_50_ = 4.18 μM), exhibiting approximately 7-fold greater activity than RBG. Importantly, **2-5c** demonstrated neuroprotective effects in MPP^+^-induced BV2 microglial cells and effectively ameliorated motor deficits in an MPTP-induced PD mouse model. These findings suggest that **2-5c** represents a promising candidate for further investigation in the development of novel Nrf2-based therapies for PD.

## 1. Introduction

Nuclear factor erythroid 2-related factor 2 (Nrf2), a pivotal transcriptional regulator in cellular oxidative stress responses, belongs to the CNC-bZIP transcription factor family. It governs the expression of over 200 cytoprotective genes by binding to antioxidant response elements (ARE) [1,2]. Under physiological conditions, Nrf2 is maintained at low basal levels through Keap1-dependent ubiquitination via complex formation with the Keap1 protein. During oxidative stress, Nrf2 dissociates from the Keap1 complex and translocates into the nucleus, where it binds to ARE promoter regions to activate antioxidant gene expression [3,4]. Nrf2 has emerged as a critical pharmacological target, with targeted modulation of the Keap1-Nrf2 signaling pathway being recognized as a key strategy for mitigating or preventing neuronal death [5,6,7,8,9,10,11]. Extensive evidence indicates that nuclear factor erythroid 2-related factor 2 (Nrf2) plays a critical role in both the pathological progression and therapeutic strategies of PD [12,13,14,15,16,17,18,19,20], making Nrf2 activation a promising novel therapeutic approach for PD.

PD is a prevalent neurodegenerative disorder primarily affecting the motor system, characterized by progressive loss of dopaminergic neurons in the substantia nigra pars compacta (SNpc) and pathological aggregation of α-synuclein [21]. Extensive in vivo and in vitro studies have demonstrated that bioactive compounds derived from natural products can exert neuroprotective effects by functioning as Nrf2 agonists to activate antioxidant defense mechanisms. Representative examples include salidroside [22,23,24], resveratrol [19,25], ginsenosides [26], tanshinone [27,28], sulforaphane [29,30], and curcumin [31,32,33]—all of which enhance Nrf2 signaling to combat PD. Consequently, the identification of Nrf2-targeting lead compounds from natural products represents a robust and promising therapeutic strategy for PD.

Resibufogenin (RBG), a principal bioactive constituent of the traditional Chinese medicine Chan Su (toad venom), possesses a unique steroidal structure and diverse pharmacological activities, including anticancer, anti-inflammatory, cardiotonic, blood pressure-regulating, and respiratory-enhancing effects [34,35,36,37,38,39,40]. Despite being one of the most thoroughly investigated constituents of toad venom, RBG’s development as a single-agent therapeutic has been hindered by cardiotoxicity and unfavorable physicochemical properties [41,42,43,44,45]. Current modification approaches, while progressing, are hampered by synthetic challenges, confining most efforts to the derivatization of the C3-hydroxy group. Meanwhile, pharmacological profiling remains overwhelmingly centered on anticancer effects [46].

Since 2019, our team has focused on exploring the synthesis of Chan Su active components. Utilizing a “biocatalytic–chemical hybrid” strategy starting from androstenedione, we have successfully established an efficient synthetic route for RBG [47]. Meanwhile, a recent study from our group has revealed that RBG acts as a novel regulator of the Keap1-Nrf2-TFR1/ARE axis, suggesting its potential neuroprotective effects through modulation of microglial homeostasis [48]. Herein, the current project conducted comprehensive structural modifications of RBG, focusing on multiple sites including ring A, ring D, and ring E (notably, the proposed modifications of the D-ring oxidation state and E-ring structure represent innovative attempts that have not been reported in the literature). The synthesized compounds were evaluated using ARE-Luc-BV2 cells. Their cytoprotective effects were further validated in BV2 cellular models, and the in vivo efficacy and safety were assessed in a PD mouse model based on behavioral performance.

## 2. Results and Discussion

### 2.1. Chemistry

**Figure 1:**
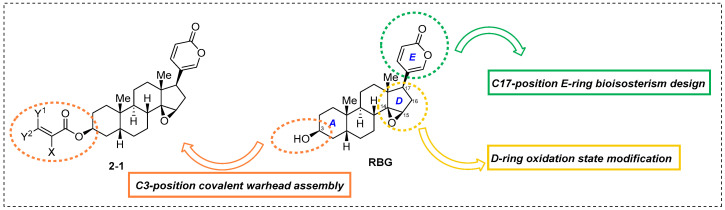
Design of RBG derivatives as Nrf2 agonists.

Structurally, RBG comprises a tetracyclic steroidal scaffold bearing a C17 β-pyranone ring and C14–C15 β-configured epoxy groups. As illustrated in Figure 1, the following rational design strategies were implemented: 1. Covalent warhead conjugation at the C3-hydroxyl group (A-ring) to enhance target engagement; 2. bioisosteric replacement of the E-ring to improve drug-like properties; 3. oxidation state modulation of the D-ring to fine-tune metabolic stability and safety profiles. These strategic modifications enable diversified structural exploration of RBG, facilitating a comprehensive structure–activity relationship (SAR) analysis to identify optimal candidates.

#### 2.1.1. Preparation of C3 Derivatives

**Scheme 1:**
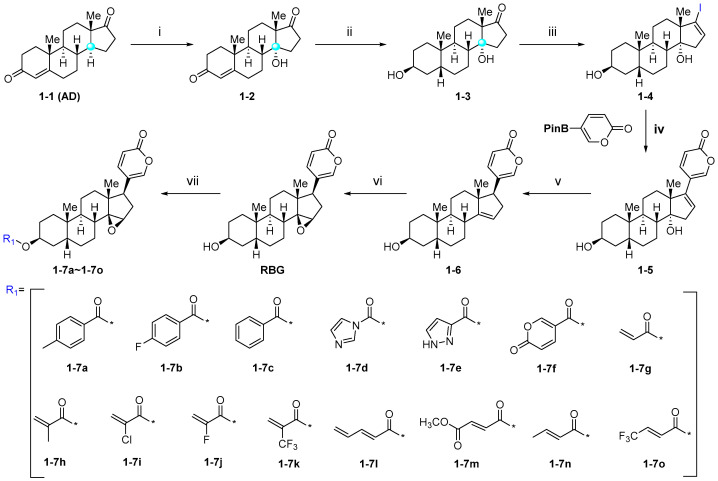
General procedures of RBG-derived C3-region analogues. Reagents and conditions: (i) P450_lun_; (ii) Pd/C, H_2_, 4-Me-pyridine; then K-selectride, THF, −7 °C; (iii) N_2_H_4_·H_2_O, Et_3_N, EtOH, 60 °C, then Et_3_N, I_2_, THF; (iv) Pd(dppf)Cl_2_, K_3_PO_4_, DMF, 60 °C; (v) Crabtree, H_2_, DCM; (vi) NBA, acetone: H_2_O: HOAc; (vii) Et_3_N, EDCI, DMAP, DCM. *: Represents the connection point with the main ring framework.

The synthesis of **1-7a**~**1-7o** is illustrated in Scheme 1. Based on the established RBG synthesis route [47], a series of 3-position esterified derivatives (**1-7a** to **1-7o**) were designed and synthesized through the condensation reaction of the C3 hydroxyl group with different acyl groups.

#### 2.1.2. Preparation of C17 Derivatives

**Scheme 2:**
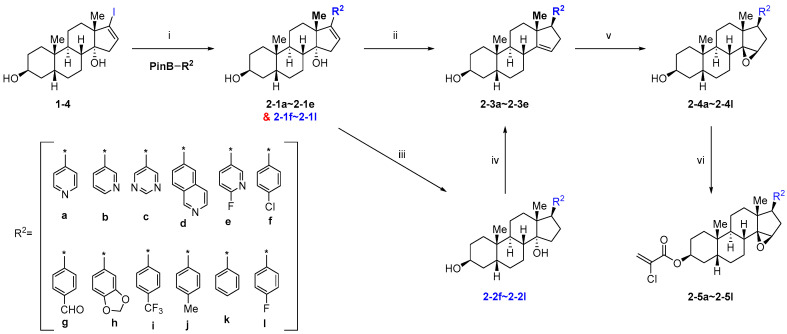
General procedures of RBG-derived C17-region analogues. Reagents and conditions: (i) Pd(dppf)Cl_2_, K_3_PO_4_, DMF, 60 °C; (ii) Crabtree, H_2_, DCM; (iii) Pd/C, H_2_, MeOH; (iv) MsOH, cyclopentyl methyl ether, −40 °C; (v) NBA, acetone: H_2_O: HOAc (vi) 2-chloroacrylic acid, EDCI, DMAP, DCM. *: Represents the connection point with the main ring framework.

The synthesis of **2-4a**~**2-4l**, as illustrated in Scheme 2, followed a procedure analogous to that of **1-7a**~**1-7r**. Starting from the Barton vinyl iodide **1-4**, the reaction was carried out using various forms of pinacolborane to afford Suzuki coupling intermediates **2-1a**~**2-1l**. Notably, when R^2^ represented a phenyl or substituted phenyl group (e.g., R^2^ = f~l), Crabtree’s catalytic hydrogenation simultaneously eliminated the 14-hydroxy group, directly yielding olefinic product **2-3f**~**2-3l**. In contrast, for aromatic heterocyclic R^2^ systems (e.g., R^2^ = a~e), formation of **2-3a**~**2-3e** required sequential Pd/C-catalyzed hydrogenation followed by methanesulfonic acid-mediated dehydration. Finally, epoxidation of the resulting intermediates provided target derivatives **2-4a**~**2-4l**.

#### 2.1.3. Preparation of C14-15 Derivatives

**Scheme 3:**
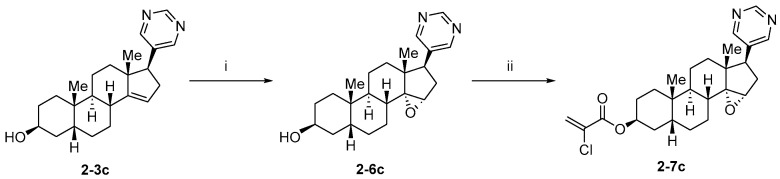
General procedures of RBG-derived D-region analogues. Reagents and conditions: (i) *m*-CPBA, Na_2_CO_3_, DCM, 0 °C to r.t.; (ii) 2-chloroacrylic acid, EDCI, DMAP, DCM.

The synthesis of **2-6c**, **2-7c** is shown in Scheme 3, the olefinic **2-3c** was first epoxidized using *m*-CPBA to afford epoxide **2-6c**, which then underwent analogous esterification to yield product **2-7c**.

The structures of target compounds were confirmed by ^1^H NMR, ^13^C NMR spectroscopy and HRMS spectrometry. Spectral information of all synthesized compounds is included in the Supplementary Materials.

### 2.2. Biological Activities of All the Molecules and Structure–Activity Relationships

To preliminarily assess the agonist activity of the synthetic compounds on Nrf2, we conducted a luciferase reporter gene assay using stably transfected ARE-Luc-BV2 cells [48]. The activation of Nrf2 was quantified by measuring the relative luminescence intensity, expressed as the fold induction relative to the control. The modification results of RBG are shown in Table 1, Table 2 and Table 3, respectively.

To evaluate the impact of C17 E-ring modifications on Nrf2 agonistic activity, we designed a series of R2-substituted analogues using bioisosteric replacement strategies (Table 2, **2-4a–2-4l**). Key findings revealed: neither phenyl nor substituted phenyl derivatives conferred activity superior to RBG; however, *N*-heterocyclic derivatives (e.g., pyridine, pyrimidine) maintained activity comparable to the lead compound. These findings suggest that while the simple aryl systems may provide essential π-π stacking interactions with the target protein, the hydrogen-bonding capacity conferred by heteroatoms appears equally crucial for optimal activity.

Building upon the current research findings, we implemented a fragment-merging strategy for in-depth structural optimization. Using the high-potency lead **1-7i** (exhibiting 4.5-fold higher relative fluorescence intensity than RBG) as the template, we retained its crucial pharmacophore (the C3 2-chloroacryloyl group) while systematically modifying the R2 position (Table 2, **2-5a~2-5l**). Notably, several derivatives demonstrated superior Nrf2 agonistic activity compared to the lead **1-7i**. Specifically, multiple compounds exhibited Nrf2 agonist activity superior to that of the preferred molecule **1-7i**. Compounds with substituents of 2-pyridine, 3-pyridine, and pyrimidinyl groups had relative fluorescence intensities of 5.48 ± 0.97, 5.08 ± 0.12, and 8.76 ± 0.40 respectively, representing 5–8 fold enhancements over RBG. This result indicates that for the target compounds, the multi-site synergy is beneficial for the improvement of Nrf2 agonist activity.

Finally, we systematically evaluated the effects of different substituents of the D-ring on Nrf2 agonistic activity. While retaining the key pharmacophore (pyrimidinyl group at C17), structural modifications were made to the oxidation state at C14–C17. The structure–activity relationship (SAR) analysis revealed that: The presence of a carbon–carbon double bond (regardless of its position at C16–C17 or C14–C15) slightly enhanced activity (e.g., Table 3 **2-1c** and **2-3c** showed higher activity than **2-2c**). Compounds with an α-OH at C14 exhibited lower activity than their epoxy-substituted counterparts (e.g., Table 3 **2-2c** was less active than **2-4c** and **2-6c**). Although the C14,15α-epoxide derivative (**2-6c**) showed marginally higher activity than the β-epoxide, introducing a 2-chloroacryloyl group at C3 reversed this trend, with the β-epoxide derivative displaying significantly superior activity to the α-configuration. Although no D-ring-modified compounds with improved activity were identified, these findings provide important theoretical insights for further structural optimization of RBG.

To more accurately evaluate the structure–activity relationship of the synthetic compounds and reduce the false positive rate, we further determined the cell viability and half-maximal effective concentration (EC_50_) of the compounds exhibiting promising activity in the preliminary screening using the ARE-Luc-BV2 cell model. The results are summarized in Table 4.

According to the results in Table 4, the C3-chloroacryloyl substituted **1-7i** displayed substantial cytotoxicity. In contrast, the C17-pyrimidine modified analogue **2-5c** exhibited markedly enhanced safety, demonstrating negligible cytotoxicity at 5 μM while showing 4-fold greater Nrf2 agonistic activity compared to the RBG positive control (EC_50_ = 4.18 ± 0.08 μM). Given this optimal activity–toxicity profile, **2-5c** was selected as our promising candidate for subsequent investigation.

### 2.3. Neuroprotective Effects of Compound ***2-5c*** Against MPP^+^-Induced Cytotoxicity in BV2 Cells

The neurotoxin 1-methyl-4-phenylpyridinium (MPP^+^) selectively impairs dopaminergic neurons, recapitulating key features of PD neurodegeneration. To evaluate the potential neuroprotective effects of **2-5c**, we established an in vitro Parkinson’s model by treating BV2 microglial cells with 800 μM MPP^+^ for 12 h following 6 h pretreatment with **2-5c** (0.5–2 μM). CCK-8 viability assays revealed that **2-5c** pretreatment significantly protected against MPP^+^-induced cytotoxicity in a dose-dependent manner (Table 5).

### 2.4. ***2-5c*** Improved Abnormal Movement in Acute MPTP-Induced PD Mice

**Figure 2:**
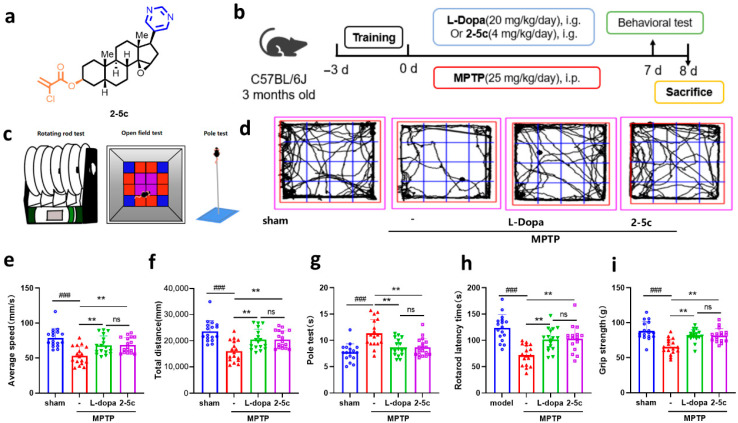
The MPTP-induced mice restored motor deficit by oral treatment of **2-5c**. (**a**) The structure of **2-5c**. (**b**) The experimental protocol. (**c**) Images for rotating rod test, open field and pole test. (**d**) Representative trajectory plots in the open field test. (**e**) Measuring the average velocity during open field exploration. (**f**) The total distance traveled was quantified during open field exploration. (**g**) Total time was measured in the climbing pole test. (**h**) The latency to fall from the rotarod was measured to assess motor coordination. (**i**) Forelimb grip strength (^###^ *p* < 0.001 vs. sham, ** *p* < 0.01 vs. model, *n* = 6).

We investigated whether **2-5c** confers effective neuroprotection in vivo. The neurotoxin 1-methyl-4-phenyl-1,2,3,6-tetrahydropyridine (MPTP) and its metabolite, 1-methyl-4-phenylpyridinium ion (MPP^+^), are well-established agents for modeling PD, as they induce mitochondrial dysfunction and cellular oxidative stress. One week after the final MPTP injection, the mice exhibited symptoms including limb incoordination and motor deficits. Subsequently, behavioral tests including the pole climb, open field, rotating rod and forelimb grip strength assays were conducted to evaluate motor coordination in the PD model mice after treatment, as shown in Figure 2.

By assessing the movement speed and distance traveled in the open field over a specified period, we evaluated the spontaneous locomotor activity of the experimental animals. As shown in Figure 2d–f, MPTP-treated mice exhibited a significant reduction in total distance moved, decreased movement velocity, and impaired motor function compared to the control group. In contrast, administration of L-Dopa or **2-5c** markedly enhanced both the total distance traveled and the average speed, demonstrating that **2-5c** effectively improves spontaneous locomotor activity in MPTP-induced mice.

Pole climbing, rod rotating, and forelimb grasping tests were employed to evaluate motor coordination in mice. As shown in Figure 2g–i, MPTP-treated mice exhibited significantly prolonged pole climbing time, reduced rod rotating time, and markedly decreased forelimb grip strength compared to the control group. In contrast, administration of L-Dopa or **2-5c** significantly improved these parameters, including shorter pole climbing time, extended rod rotating time, and enhanced forelimb strength relative to the MPTP group. Taken together, these findings reveal the efficacy of **2-5c** in mitigating motor impairments in an MPTP-induced mouse model.

### 2.5. ***2-5c*** Protected Dopaminergic Neurons from Oxidative Stress Induced by MPTP in the SNpc

**Figure 3:**
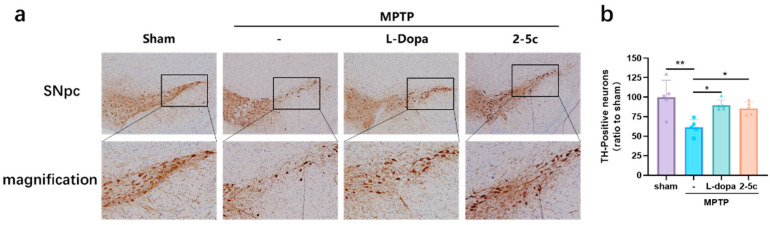
Rescue of dopaminergic neurons in the MPTP-induced mouse model by oral treatment of **2-5c**. The mice were treated with L-Dopa or **2-5c**, and their midbrain slices (sacrificed 8 days later) were observed by immunohistochemistry with anti-TH antibody. (**a**) Representative immunohistochemical. Staining (brown, TH) in the SNpc. (**b**) Quantitative analysis of TH-positive neurons in the SNpc by Imag J. The results are shown as the mean ± SEM. * *p* < 0.05, ** *p* < 0.01, vs. the MPTP-treated group (one-way ANOVA with Tukey’s multiple comparison test).

MPP^+^ is selectively taken up by dopaminergic neurons and accumulates within mitochondria, resulting in diminished mitochondrial membrane potential and elevated oxidative stress. Accordingly, MPTP administration triggered degeneration of dopaminergic neurons in the substantia nigra pars compacta (SNpc), marked by a substantial reduction in tyrosine hydroxylase (TH)-positive cells. As illustrated in Figure 3, MPTP treatment resulted in a significant decrease in the density of tyrosine hydroxylase (TH)-positive neurons. In contrast, both L-Dopa and **2-5c** administration markedly restored TH-positive neuron density. Collectively, these results indicate that although the restorative efficacy of **2-5c** was moderately lower than that of L-Dopa, it still exhibited significant neuroprotective effects against MPTP-induced neurotoxicity in vivo.

## 3. Discussion

**Scheme 4:**
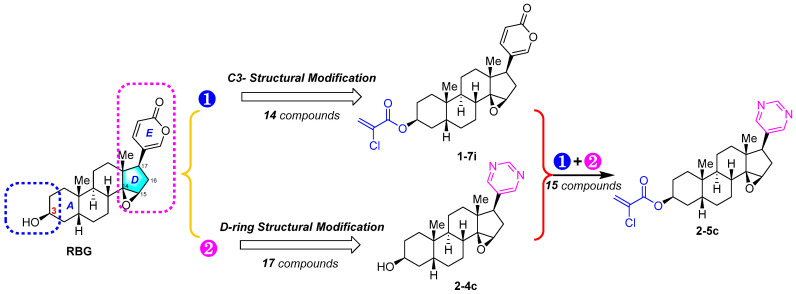
The structural modification strategy and results of RBG.

In this study, we systematically investigated the activation effect of RBG derivatives modified at multiple sites on Nrf2 by measuring their impact on the fluorescence intensity of ARE-Luc-BV2 cells. Through rational modifications at the C3, C14–C15, and C17 positions, we identified **2-5c** as a potential Nrf2 activator (EC_50_ = 4.18 μM) (Scheme 4).

The SAR analysis revealed several key insights. Modifications at the C3 position indicated that the introduction of an acryloyl fragment (e.g., **1-7i**) could enhance activity, albeit with increased cytotoxicity. In contrast, bioisosteric replacement of the C17 E-ring with nitrogen-containing heterocycles, particularly the pyrimidine moiety in **2-5c**, substantially improved both potency and safety. This suggests that the hydrogen-bonding capacity of the heteroatom plays a critical role in optimizing target engagement. Furthermore, modifications to the D-ring oxidation state (C14–C15) revealed that the β-epoxide configuration, when combined with the C3 2-chloroacryloyl group, was more favorable for activity, underscoring the importance of multi-site synergistic effects.

The neuroprotective efficacy of **2-5c** was validated both in vitro and in vivo. In MPP^+^-treated BV2 cells, **2-5c** pretreatment significantly improved cell viability. In the MPTP-induced PD mouse model, oral administration of **2-5c** effectively ameliorated motor deficits, as evidenced by improved performance in open field, rotarod, pole climb, and grip strength tests. These behavioral improvements were correlated with a significant rescue of tyrosine hydroxylase (TH)-positive dopaminergic neurons in the SNpc.

Despite the promising findings obtained in this study, certain limitations should be acknowledged. Through the ARE-Luc-BV2 cell model, we systematically investigated the activation effects of RBG derivatives modified at multiple sites on Nrf2, identifying **2-5c** as a potential candidate with favorable activity, and further validated its in vivo efficacy. However, the current study remains limited in terms of mechanistic exploration and is insufficient to clearly elucidate the precise targeting mechanism of **2-5c**. Therefore, future research should focus on in-depth mechanistic target analysis, combined with a comprehensive efficacy evaluation system, to further clarify its mode of action and therapeutic potential.

## 4. Materials and Methods

### 4.1. Synthesis

All reactions were carried out under a nitrogen atmosphere with dry solvents under anhydrous conditions, unless otherwise noted. All reagents and solvents were commercially available and used without further purification. Reactions were monitored by thin layer chromatography (TLC) on silica gel plates under ultraviolet (UV) light. Product purification was performed as flash column chromatography separations on silica gel (300–400 mesh, Greagent). Nuclear magnetic resonance (NMR) spectra were recorded on a BRUKER AVANCE NEO 600 MHz spectrometer (Bruker Co., Fällanden, Switzerland) or a BRUKER AVANCE NEO 400 MHz spectrometer (Bruker Co., Switzerland). The spectra were calibrated by using residual undeuterated solvents (for ^1^H NMR) and deuterated solvents (for ^13^C NMR) as internal references: undeuterated chloroform (δH = 7.26 ppm) and CDCl_3_ (δC = 77.16 ppm); undeuterated methanol (δH = 3.31 ppm) and methanol-d4 (δC = 49.00 ppm); NMR data were reported as chemical shifts (*δ*, ppm), coupling constants (*J*, Hz), multiplicity and integration. High resolution mass spectra (HRMS) were obtained from 6545 Accurate-Mass Q-TOF (Agilent Technologies, Inc., Santa Clara, CA, USA).

#### Characterization for All Compounds (**1-7a**–**2-8c**)

*Part 1. Preparation and characterization of C3 derivatives* **1-7a**–**1-7o**.

4-dimethylaminopyridine (4.8 mg, 0.4 mmol), triethylamine (15.8 mg, 21.8 μL, 0.16 mmol), and the acyl chloride reagent (R1-Cl, 3 equiv) were added to a solution of resibufogenin (30.0 mg, 0.08 mmol) in dichloromethane (1 mL). After reacting for 15 h, the reaction was quenched with saturated ammonium chloride (5 mL) and extracted with ethyl acetate (3 × 6 mL). The organic layer was washed with a saturated sodium chloride solution and dried over anhydrous sodium sulfate. The crude product obtained after concentration was purified by column chromatography (petroleum ether: EtOAc) to give RBG C3 derivatives **1-7a**–**1-7o**.

(1*R*,2a*R*,3a*S*,3b*R*,5a*R*,7*S*,9a*S*,9b*S*,11a*R*)-9a,11a-dimethyl-1-(2-oxo-2*H*-pyran-5-yl)hexadeca -hydronaphtho[1′,2′:6,7]indeno[1,7a-b]oxiren-7-yl 4-methylbenzoate (**1-7a**). White solid, yield in 20%. [α]_D_^22.4^ = 11.00 (c 0.100, MeOH). ^1^H NMR (400 MHz, CDCl_3_) δ = 7.93 (d, *J* = 8.0 Hz, 2H), 7.49 (dd, *J* = 9.7, 2.2 Hz, 1H), 7.28 (s, 1H), 7.24 (d, *J* = 8.0 Hz, 2H), 6.31 (d, *J* = 9.7 Hz, 1H), 5.35 (s, 1H), 4.29 (d, *J* = 5.8 Hz, 1H), 2.95 (t, *J* = 9.3 Hz, 1H), 2.6–2.46 (m, 2H), 2.41 (s, 3H), 2.32–2.10 (m, 3H), 1.85–1.74 (m, 3H), 1.70–1.61 (m, 6H), 1.56–1.48 (m, 2H), 1.37 (d, *J* = 12.5 Hz, 1H), 1.28–1.18 (m, 2H), 0.96 (s, 3H), 0.75 (s, 3H). ^13^C NMR (101 MHz, CDCl_3_) δ = 166.1, 162.2, 148.7, 145.8, 143.5, 129.7, 129.2, 128.4, 120.4, 115.9, 86.4, 71.0, 65.2, 50.0, 47.5, 43.8, 41.2, 40.8, 37.4, 35.7, 34.4, 31.0, 30.9, 29.8, 26.7, 25.4, 22.6, 21.8, 21.1, 20.3, 19.0. HRMS-ESI (*m*/*z*): [M + H]^+^ calcd for C_32_H_39_O_5_^+^: 503.2792; found: 503.2799. m.p.: 214–215 °C.

(1*R*,2a*R*,3a*S*,3b*R*,5a*R*,7*S*,9a*S*,9b*S*,11a*R*)-9a,11a-dimethyl-1-(2-oxo-2*H*-pyran-5-yl)hexadeca -hydronaphtho[1′,2′:6,7]indeno[1,7a-b]oxiren-7-yl 4-fluorobenzoate (**1-7b**). White solid, yield in 25%. [α]_D_^22.4^ = 2.00 (c 0.100, MeOH). ^1^H NMR (400 MHz, CDCl_3_) δ = 8.04 (s, 2H), 7.47 (d, *J* = 9.7 Hz, 1H), 7.24 (s, 1H), 7.09 (t, *J* = 8.2 Hz, 2H), 6.29 (d, *J* = 9.6 Hz, 1H), 5.33 (s, 1H), 4.27 (d, *J* = 5.1 Hz, 1H), 2.92 (t, *J* = 9.1 Hz, 1H), 2.63–2.44 (m, 2H), 2.31–2.07 (m, 3H), 1.76 (d, *J* = 15.8 Hz, 4H), 1.63 (d, *J* = 7.4 Hz, 6H), 1.48 (t, *J* = 12.7 Hz, 2H), 1.34–1.16 (m, 5H), 0.94 (s, 3H), 0.72 (s, 3H). ^13^C NMR (101 MHz, CDCl_3_) δ = 167.0, 165.1, 164.5, 162.2, 148.7, 145.8, 132.2, 132.1, 127.4, 120.4, 115.7, 86.4, 71.5, 65.2, 50.0, 47.5, 43.8, 41.2, 40.8, 37.5, 35.7, 34.4, 31.1, 31.0, 29.8, 26.7, 25.4, 22.7, 21.1, 20.3, 19.0. HRMS-ESI (*m*/*z*): [M + H]^+^ calcd for C_31_H_36_FO_5_^+^: 507.2541; found: 507.2549. m.p.: 217–218 °C.

(1*R*,2a*R*,3a*S*,3b*R*,5a*R*,7*S*,9a*S*,9b*S*,11a*R*)-9a,11a-dimethyl-1-(2-oxo-2*H*-pyran-5-yl)hexadeca -hydronaphtho[1′,2′:6,7]indeno[1,7a-b]oxiren-7-yl benzoate (**1-7c**). White solid, yield in 26%. [α]_D_^22.6^ = 10.91 (c 0.100, MeOH). ^1^H NMR (400 MHz, CDCl_3_) δ = 8.05 (d, *J* = 7.2 Hz, 2H), 7.62–7.39 (m, 4H), 7.28 (d, *J* = 1.8 Hz, 1H), 6.31 (d, *J* = 9.7 Hz, 1H), 5.37 (s, 1H), 4.29 (d, *J* = 5.9 Hz, 1H), 2.95 (t, *J* = 9.4 Hz, 1H), 2.65–2.44 (m, 2H), 2.35–2.10 (m, 3H), 1.94–1.75 (m, 4H), 1.69–1.61 (m, 5H), 1.58–1.48 (m, 3H), 1.40–1.19 (m,2H), 0.97 (s, 3H), 0.75 (s, 3H). ^13^C NMR (101 MHz, CDCl_3_) δ = 166.0, 148.7, 145.8, 132.9, 131.2, 129.7, 128.5, 120.4, 116.0, 86.4, 71.3, 65.2, 50.1, 47.6, 43.9, 41.3, 40.8, 37.5, 35.8, 34.4, 31.1, 31.0, 26.8, 25.5, 22.7, 21.1, 20.3, 19.0. HRMS-ESI (*m*/*z*): [M + H]^+^ calcd for C_31_H_37_O_5_^+^: 489.2636; found: 489.2645. m.p.: 206–207 °C.

(1*R*,2a*R*,3a*S*,3b*R*,5a*R*,7*S*,9a*S*,9b*S*,11a*R*)-9a,11a-dimethyl-1-(2-oxo-2*H*-pyran-5-yl)hexadeca -hydronaphtho[1′,2′:6,7]indeno[1,7a-b]oxiren-7-yl 1*H*-imidazole-1-carboxylate (**1-7d**). White solid, yield in 26%. [α]_D_^22.6^ = 7.53 (c 0.170, MeOH). ^1^H NMR (400 MHz, CDCl_3_) δ = 8.19 (s, 1H), 7.78 (dd, *J* = 9.7, 2.3 Hz, 1H), 7.45 (s, 1H), 7.23 (s, 1H), 7.11 (s, 1H), 6.25 (d, *J* = 9.7 Hz, 1H), 5.35 (s, 1H), 3.53 (s, 1H), 2.51–2.34 (m, 2H), 2.08–1.94 (m, 3H), 1.92–1.82 (m, 2H), 1.77–1.64 (m, 5H), 1.63–1.50 (m, 3H), 1.48–1.33 (m, 3H), 1.29–1.25 (m, 2H), 1.04 (s, 3H), 0.79 (s, 3H). ^13^C NMR (101 MHz, CDCl_3_) δ = 162.1, 149.7, 147.0, 122.3, 115.5, 76.2, 74.6, 59.9, 47.9, 45.4, 39.7, 39.4, 37.3, 35.5, 33.7, 32.5, 30.6,30.4, 25.7, 25.1, 24.0, 21.2, 20.8, 17.0. HRMS-ESI (*m*/*z*): [M + H]^+^ calcd for C_28_H_35_N_2_O_5_^+^: 479.2540; found: 479.2572. m.p.: 244–245 °C.

(1*R*,2a*R*,3a*S*,3b*R*,5a*R*,7*S*,9a*S*,9b*S*,11a*R*)-9a,11a-dimethyl-1-(2-oxo-2*H*-pyran-5-yl)hexadeca -hydronaphtho[1′,2′:6,7]indeno[1,7a-b]oxiren-7-yl 1*H*-pyrazole-3-carboxylate (**1-7e**). White solid, yield in 16%. [α]_D_^22.7^ = 2.00 (c 0.100, MeOH). ^1^H NMR (400 MHz, CDCl_3_) δ = 7.80 (d, *J* = 10.1 Hz, 2H), 7.24 (s, 1H), 6.85 (s, 1H), 6.25 (d, *J* = 9.7 Hz, 1H), 5.38 (s, 1H), 3.55 (s, 1H), 2.54–2.34 (m, 2H), 2.07–1.88 (m, 4H), 1.81 (d, *J* = 12.6 Hz, 2H), 1.70–1.48 (m, 8H), 1.36–1.18 (m, 4H), 1.02 (s, 3H), 0.78 (s, 3H). ^13^C NMR (101 MHz, CDCl_3_) δ = 162.1, 149.7, 147.0, 122.3, 115.5, 76.2, 74.6, 59.9, 47.9, 45.4, 39.7, 39.4, 37.3, 35.5, 33.7, 32.5, 30.6,30.4, 25.7, 25.1, 24.0, 21.2, 20.8, 17.0 HRMS-ESI (*m*/*z*): [M + H]^+^ calcd for C_28_H_35_N_2_O_5_^+^: 479.2540; found: 479.2553. m.p.: 280–281 °C.

(1*R*,2a*R*,3a*S*,3b*R*,5a*R*,7*S*,9a*S*,9b*S*,11a*R*)-9a,11a-dimethyl-1-(2-oxo-2*H*-pyran-5-yl)hexadeca -hydronaphtho[1′,2′:6,7]indeno[1,7a-b]oxiren-7-yl 2-oxo-2*H*-pyran-5-carboxylate (**1-7f**). White solid, yield in 63%. [α]_D_^22.8^ = 15.71 (c 0.140, MeOH). ^1^H NMR (400 MHz, CDCl_3_) δ = 8.28 (dd, *J* = 2.5, 1.0 Hz, 1H), 7.77 (dd, *J* = 9.8, 2.5 Hz, 2H), 7.23 (d, *J* = 1.8 Hz, 1H), 6.34 (dd, *J* = 9.8, 0.9 Hz, 1H), 6.23 (d, *J* = 9.7 Hz, 1H), 5.30 (s, 1H), 3.52 (s, 1H), 2.49–2.32 (m, 2H), 2.05–1.82 (m, 4H), 1.77–1.49 (m, 9H), 1.46–1.16 (m, 5H), 1.02 (s, 3H), 0.78 (s, 3H). ^13^C NMR (101 MHz, CDCl_3_) δ = 162.1, 149.7, 147.0, 122.3, 115.5, 76.2, 74.6, 59.9, 47.9, 45.4, 39.7, 39.4, 37.3, 35.5, 33.7, 32.5, 30.6, 30.4, 25.7, 25.1, 24.0, 21.2, 20.8, 17.0. HRMS-ESI (*m*/*z*): [M + Na]^+^ calcd for C_30_H_34_NaO_7_^+^: 529.2197; found: 529.2204. m.p.: 223–224 °C.

(1*R*,2a*R*,3a*S*,3b*R*,5a*R*,7*S*,9a*S*,9b*S*,11a*R*)-9a,11a-dimethyl-1-(2-oxo-2*H*-pyran-5-yl)hexadeca -hydronaphtho[1′,2′:6,7]indeno[1,7a-b]oxiren-7-yl acrylate (**1-7g**). White solid, yield in 53%. [α]_D_^22.8^ = −3.40 (c 0.100, MeOH). ^1^H NMR (400 MHz, CDCl_3_) δ = 7.78 (dd, *J* = 9.8, 2.4 Hz, 1H), 7.23 (d, *J* = 1.7 Hz, 1H), 6.39 (dd, *J* = 17.3, 1.5 Hz, 1H), 6.24 (dd, *J* = 9.8, 0.6 Hz, 1H), 6.13 (dd, *J* = 17.3, 10.4 Hz, 1H), 5.81 (dd, *J* = 10.4, 1.5 Hz, 1H), 5.17 (s, 1H), 3.53 (s, 1H), 2.50–2.33 (m, 2H), 2.03–1.83 (m, 4H), 1.76–1.65 (m, 3H), 1.65–1.56 (m, 3H), 1.55–1.45 (m, 3H), 1.45–1.32 (m, 3H), 1.30–1.28 (m, 2H), 1.00 (s, 3H), 0.78 (s, 3H). ^13^C NMR (101 MHz, CDCl_3_) δ = 165.8, 162.1, 149.7, 147.1, 130.4, 129.3, 122.3, 115.4, 74.8, 70.60, 60.0, 47.9, 45.4, 39.6, 39.5, 37.0, 35.4, 33.7, 32.5, 30.6, 25.8, 25.2, 23.9, 21.2, 20.8, 17.0. HRMS-ESI (*m*/*z*): [M + Na]^+^ calcd for C_27_H_34_NaO_5_^+^: 461.2298; found: 461.2307. m.p.: 172–173 °C.

(1*R*,2a*R*,3a*S*,3b*R*,5a*R*,7*S*,9a*S*,9b*S*,11a*R*)-9a,11a-dimethyl-1-(2-oxo-2*H*-pyran-5-yl)hexadeca -hydronaphtho[1′,2′:6,7]indeno[1,7a-b]oxiren-7-yl methacrylate (**1-7h**). White solid, yield in 28%. [α]_D_^22.9^ = 9.60 (c 0.050, MeOH). ^1^H NMR (400 MHz, CDCl_3_) δ = 7.48 (d, *J* = 9.5 Hz, 1H), 7.25 (s, 1H), 6.30 (d, *J* = 9.6 Hz, 1H), 6.09 (s, 1H), 5.53 (s, 1H), 5.16 (s, 1H), 4.26 (d, *J* = 5.3 Hz, 1H), 2.92 (t, *J* = 9.2 Hz, 1H), 2.54–2.39 (m, 2H), 2.33–2.01 (m, 3H),1.94 (s, 3H), 1.74–1.47 (m, 11H), 1.39–1.12 (m, 4H), 0.91 (s, 3H), 0.72 (s, 3H). ^13^C NMR (101 MHz, CDCl_3_) δ = 166.9, 162.1, 148.7, 145.8, 137.3, 125.1, 120.4, 116.0, 86.4, 70.8, 65.2, 50.1, 47.6, 43.9, 41.3, 40.8, 37.4, 35.7, 34.4, 31.0, 29.8, 26.7, 25.3, 22.6, 21.1, 20.3, 19.0, 18.5. HRMS-ESI (*m*/*z*): [M + H]^+^ calcd for C_28_H_37_O_5_^+^: 453.2636; found: 453.2639. m.p.: 197–198 °C.

(1*R*,2a*R*,3a*S*,3b*R*,5a*R*,7*S*,9a*S*,9b*S*,11a*R*)-9a,11a-dimethyl-1-(2-oxo-2*H*-pyran-5-yl)hexadeca -hydronaphtho[1′,2′:6,7]indeno[1,7a-b]oxiren-7-yl 2-chloroacrylate (**1-7i**). White solid, yield in 60%. [α]_D_^22.9^ = −5.00 (c 0.080, MeOH). ^1^H NMR (400 MHz, CDCl_3_) δ = 7.78 (d, *J* = 9.8 Hz, 1H), 7.24 (s, 1H), 6.51 (s, 1H), 6.25 (d, *J* = 9.7 Hz, 1H), 6.00 (s, 1H), 5.21 (s, 1H), 3.53 (s, 1H),2.50–2.33 (m, 2H), 2.06–1.84 (m, 4H), 1.75–1.71 (m, 2H), 1.65–1.61 (m, 3H),1.56–1.52 (m, 3H), 1.46–1.33 (m, 4H), 1.30–1.23 (m, 2H), 1.01 (s, 3H), 0.78(s,3H). ^13^C NMR (101 MHz, CDCl_3_) δ = 162.2, 161.4, 149.7, 147.1, 132.4, 125.4, 122.3, 115.5, 74.7, 73.2, 60.0, 47.9, 45.4, 39.7, 39.5, 37.1, 35.5, 33.7, 32.6, 30.6, 30.5, 25.8, 25.2, 24.0, 21.2, 20.9, 17.0. HRMS-ESI (*m*/*z*): [M + Na]^+^ calcd for C_27_H_33_ClNaO_5_^+^: 495.1909; found: 495.1916. m.p.: 110–111 °C.

(1*R*,2a*R*,3a*S*,3b*R*,5a*R*,7*S*,9a*S*,9b*S*,11a*R*)-9a,11a-dimethyl-1-(2-oxo-2*H*-pyran-5-yl)hexadeca -hydronaphtho[1′,2′:6,7]indeno[1,7a-b]oxiren-7-yl 2-fluoroacrylate (**1-7j**). White solid, yield in 44%. [α]_D_^22.9^ = 0.00 (c 0.130, MeOH). ^1^H NMR (400 MHz, CDCl_3_) δ = 7.78 (d, *J* = 9.7 Hz, 1H), 7.23 (s, 1H), 6.24 (d, *J* = 9.8 Hz, 1H), 5.65 (dd, *J* = 43.3, 2.9 Hz, 1H), 5.31 (dd, *J* = 13.0, 2.9 Hz, 1H), 5.24 (s, 1H), 3.53 (s, 1H), 2.50–2.33 (m, 2H), 2.04–1.83 (m, 4H),1.75–1.58 (m, 6H), 1.58–1.49 (m, 3H), 1.44–1.31 (m, 3H), 1.25–1.22 (m, 2H),1.01 (s, 3H), 0.78 (s, 3H). ^13^C NMR (101 MHz, CDCl_3_) δ = 162.1, 160.1, 155.2, 152.6, 149.7, 147.1, 122.3, 115.4, 102.5, 74.7, 72.6, 60.0, 47.9, 45.4, 39.7, 39.4, 37.0, 35.4, 33.7, 32.5, 30.5, 25.7, 25.1, 23.9, 21.2, 20.8, 17.0. HRMS-ESI (*m*/*z*): [M + Na]^+^ calcd for C_27_H_33_FNaO_5_^+^: 479.2204; found: 479.2212. m.p.: 168–169 °C.

(1*R*,2a*R*,3a*S*,3b*R*,5a*R*,7*S*,9a*S*,9b*S*,11a*R*)-9a,11a-dimethyl-1-(2-oxo-2*H*-pyran-5-yl)hexadeca -hydronaphtho[1′,2′:6,7]indeno[1,7a-b]oxiren-7-yl 2-(trifluoromethyl)acrylate (**1-7k**). White solid, yield in 26%. [α]_D_^22.9^ = −3.40 (c 0.100, MeOH). ^1^H NMR (400 MHz, CDCl_3_) δ = 7.78 (dd, *J* = 9.7, 1.9 Hz, 1H), 7.23 (s, 1H), 6.72 (s, 1H), 6.41 (s, 1H), 6.24 (d, *J* = 9.8 Hz, 1H), 5.28 (s, 1H), 3.53 (s, 1H), 2.47–2.35 (m, 2H), 2.04–1.85 (m, 4H), 1.73–1.68 (m, 2H), 1.65–1.59 (m, 4H), 1.57–1.50 (m, 3H), 1.46–1.33 (m, 3H), 1.25–1.22 (m, 2H), 1.00 (s, 3H), 0.78 (s, 3H). ^13^C NMR (101 MHz, CDCl_3_) δ = 162.1, 160.9, 149.7, 147.1, 132.8, 132.0, 122.3, 120.2, 115.5, 74.7, 72.7, 60.0, 47.9, 45.4, 39.7, 39.5, 36.9, 35.4, 33.7, 32.6, 30.5, 30.3, 25.8, 25.2, 23.9, 21.2, 20.8, 17.0. HRMS-ESI (*m*/*z*): [M + H]^+^ calcd for C_28_H_34_F_3_O_5_^+^: 507.2353; found: 507.2362. m.p.: 197–198 °C.

(1*R*,2a*R*,3a*S*,3b*R*,5a*R*,7*S*,9a*S*,9b*S*,11a*R*)-9a,11a-dimethyl-1-(2-oxo-2*H*-pyran-5-yl)hexadeca -hydronaphtho[1′,2′:6,7]indeno[1,7a-b]oxiren-7-yl (*E*)-penta-2,4-dienoate (**1-7l**). White solid, yield in 50%. [α]_D_^23.1^ = 18.00 (c 0.060, MeOH). ^1^H NMR (400 MHz, CDCl_3_) δ = 7.78 (dd, *J* = 9.7, 2.3 Hz, 1H), 7.30–7.19 (m, 2H), 6.46 (dt, *J* = 16.9, 10.5 Hz, 1H), 6.24 (d, *J* = 9.8 Hz, 1H), 5.93 (d, *J* = 15.4 Hz, 1H), 5.61 (d, *J* = 16.9 Hz, 1H), 5.49 (d, *J* = 10.1 Hz, 1H), 5.17 (s, 1H), 3.53 (s, 1H), 2.49–2.33 (m, 2H), 2.02–1.83 (m, 4H), 1.75–1.64 (m,3H), 1.62–1.56 (m, 3H), 1.54–1.47 (m, 3H), 1.45–1.31 (m, 3H), 1.30–1.17 (m,2H), 1.00 (s, 3H), 0.78 (s, 3H). ^13^C NMR (101 MHz, CDCl_3_) δ = 166.4, 162.1, 149.7, 147.1, 144.5, 134.9, 125.6, 123.0, 122.3, 115.4, 74.7, 70.4, 60.0, 47.9, 45.4, 39.6, 39.4, 37.0, 35.4, 33.7, 32.5, 30.6, 25.8, 25.2, 23.9, 21.2, 20.8, 17.0. HRMS-ESI (*m*/*z*): [M + H]^+^ calcd for C_29_H_37_O_5_^+^: 465.2636; found: 465.2666. m.p.: 99–100 °C.

(1*R*,2a*R*,3a*S*,3b*R*,5a*R*,7*S*,9a*S*,9b*S*,11a*R*)-9a,11a-dimethyl-1-(2-oxo-2*H*-pyran-5-yl)hexadeca -hydronaphtho[1′,2′:6,7]indeno[1,7a-b]oxiren-7-yl methyl fumarate (**1-7m**). White solid, yield in 58%. [α]_D_^23.1^ = 4.67 (c 0.120, MeOH). ^1^H NMR (400 MHz, CDCl_3_) δ = 7.78 (dd, *J* = 9.8, 2.4 Hz, 1H), 7.23 (s, 1H), 6.94–6.75 (m, 2H), 6.24 (d, *J* = 9.8 Hz, 1H), 5.21 (s, 1H), 3.81 (s, 3H), 3.53 (s, 1H), 2.50–2.32 (m, 2H), 2.05–1.80 (m, 4H), 1.74–1.62 (m, 4H), 1.61–1.46 (m, 5H), 1.45–1.32 (m, 3H), 1.31–1.17 (m, 2H), 1.00 (s, 3H), 0.78(s, 3H). ^13^C NMR (101 MHz, CDCl_3_) δ = 165.7, 164.5, 162.1, 149.7, 147.1, 134.6, 133.1, 122.3, 115.4, 74.7, 71.8, 60.0, 52.5, 47.9, 45.4, 39.6, 39.5, 37.0, 35.4, 33.7, 32.5, 30.5, 25.7, 25.1, 23.9, 21.2, 20.8, 17.0. HRMS-ESI (*m*/*z*): [M + Na]^+^ calcd for C_29_H_36_NaO_7_^+^: 519.2353; found: 519.2362. m.p.: 165–166 °C.

(1*R*,2a*R*,3a*S*,3b*R*,5a*R*,7*S*,9a*S*,9b*S*,11a*R*)-9a,11a-dimethyl-1-(2-oxo-2*H*-pyran-5-yl)hexadeca -hydronaphtho[1′,2′:6,7]indeno[1,7a-b]oxiren-7-yl (*E*)-but-2-enoate (**1-7n**). White solid, yield in 40%. [α]_D_^23.1^ = 11.99 (c 0.140, MeOH). ^1^H NMR (400 MHz, CDCl_3_) δ = 7.48 (d, *J* = 9.6 Hz, 1H), 7.25 (s, 1H), 6.95–6.80 (m, 1H), 6.30 (d, *J* = 9.5 Hz, 1H), 5.85 (d, *J* = 15.5 Hz, 1H), 5.14 (s,1H), 4.26 (d, *J* = 5.2 Hz, 1H), 2.92 (t, *J* = 9.2 Hz, 1H), 2.58–2.42 (m, 2H), 2.31–2.14 (m, 2H), 2.04 (t, *J* = 13.9 Hz, 1H), 1.87 (d, *J* = 6.5 Hz, 3H), 1.73–1.58 (m, 7H), 1.58–1.45 (m, 4H), 1.32–1.24 (m, 2H), 1.25–1.15 (m, 1H), 0.90 (s, 3H), 0.72 (s, 3H). ^13^C NMR (101 MHz, CDCl_3_) δ = 166.2, 162.1, 148.7, 145.8, 144.2, 123.6, 120.4, 115.9, 86.4, 70.3, 65.2, 50.0, 47.6, 43.8, 41.2, 40.8, 37.2, 35.6, 34.4, 30.9, 29.8, 26.7, 25.3, 22.5, 21.1, 20.3, 19.0, 18.1. HRMS-ESI (*m*/*z*): [M + H]^+^ calcd for C_28_H_37_O_5_^+^: 453.2636; found: 453.2644. m.p.: 212–213 °C.

(1*R*,2a*R*,3a*S*,3b*R*,5a*R*,7*S*,9a*S*,9b*S*,11a*R*)-9a,11a-dimethyl-1-(2-oxo-2*H*-pyran-5-yl)hexadeca -hydronaphtho[1′,2′:6,7]indeno[1,7a-b]oxiren-7-yl (*E*)-4,4,4-trifluorobut-2-enoate (**1-7o**). White solid, yield in 86%. [α]_D_^23.1^ = 3.25 (c 0.080, MeOH). ^1^H NMR (400 MHz, CDCl_3_) δ = 7.77 (dd, *J* = 9.7, 2.3 Hz, 1H), 7.23 (d, *J* = 1.6 Hz, 1H), 6.82–6.69 (m, 1H), 6.51 (dd, *J* = 15.8, 1.8 Hz, 1H), 6.23 (d, *J* = 9.7 Hz, 1H), 5.22 (s, 1H), 3.52 (s, 1H), 2.50–2.32 (m, 2H), 2.06–1.83 (m,4H), 1.74–1.58 (m, 6H), 1.57–1.48 (m, 3H), 1.43–1.30 (m, 3H), 1.29–1.19 (m, 2H), 1.01 (s, 3H), 0.78 (s, 3H). ^13^C NMR (101 MHz, CDCl_3_) δ = 163.5, 162.1, 149.7, 147.0, 131.4, 131.1, 129.5, 122.3, 115.4, 74.7, 72.3, 60.0, 47.9, 45.4, 39.6, 39.4, 37.0, 35.4, 33.7, 32.5, 30.5, 30.4, 25.7, 25.1, 23.9, 21.2, 20.8, 17.0. HRMS-ESI (*m*/*z*): [M + Na]^+^ calcd for C_28_H_33_F_3_NaO_5_^+^: 529.2172; found: 529.2186. m.p.: 185–186 °C.

*Part 2. Preparation and characterization of* **2-1a**~**2-1l**.

Et_3_N (1.8 mL, 13 mmol) and N_2_H_4_∙H_2_O (0.5 mL, 13 mmol, 85% *w*/*w*) were added to a stirred solution of compound **1-3** (200.0 mg, 0.65 mmol) in EtOH (3 mL). The mixture was heated to 60 °C and allowed to stir at that temperature for 6 h before the reaction solvent was removed under vacuum. The crude residue was directly used in the next step without further purification. A solution of I_2_ (495 mg, 1.95 mmol) in THF (3 mL) at 0 °C was slowly added to a stirred solution of the crude hydrazone and Et_3_N (1.8 mL, 13 mmol) in THF (10 mL). The mixture was warmed to room temperature and allowed to stir at that temperature for 3 h before it was quenched with saturated aq. Na_2_SO_3_ (50 mL). The resultant mixture was extracted with EtOAc (3 × 50 mL), and the combined organic phases were washed with saturated aq. NaHCO_3_ (100 mL) and brine (100 mL), dried over anhydrous Na_2_SO_4_, and filtered. The solvent was evaporated under vacuum, and the crude residue was directly used in the next step without further purification. Pd(dppf)Cl_2_ (47.6 mg, 0.065 mmol) and K_3_PO_4_ (534 mg, 2.4 mmol) were added to a stirred solution of crude vinyl iodide, boronic acid pinacol ester **5** (0.78 mmol) in DMF (11 mL). The mixture was heated to 60 °C and allowed to stir at that temperature for 5 h before the reaction solvent was removed under vacuum. The residue was dissolved in EtOAc (200 mL), and the organic phase was washed with saturated aq. NH_4_Cl (100 mL) and brine (100 mL), dried over anhydrous Na_2_SO_4_, and filtered. The solvent was evaporated under vacuum, and the residue was purified by flash column chromatography with EtOAc:petroleum ether (3:2) to give **2-1a~2-1l**.

(3*S*,5*R*,8*R*,9*S*,10*S*,13*R*,14*R*)-10,13-dimethyl-17-(pyridin-4-yl)-1,2,3,4,5,6,7,8,9,10,11,12,13,15 -tetradecahydro-14*H*-cyclopenta[*a*]phenanthrene-3,14-diol (**2-1a**). White solid, yield in 27% for two steps. ^1^H NMR (400 MHz, CDCl_3_) δ = 6.14 (dd, *J* = 3.3, 1.9 Hz, 1H), 8.53 (d, *J* = 5.2 Hz, 2H), 7.25 (d, *J* = 4.7 Hz, 2H), 4.13 (s, 1H), 2.49 (dd, *J* = 16.8, 1.9 Hz, 1H), 2.34 (dd, *J* = 16.8, 3.3 Hz, 1H), 2.26–2.11 (m, 2H), 2.11–2.02 (m, 1H), 2.03–1.90 (m, 2H), 1.84 (s, 1H), 1.78–1.71 (m, 2H), 1.69–1.62 (m, 2H), 1.54 (d, *J* = 1.6 Hz, 2H), 1.53–1.46 (m, 3H), 1.45–1.41 (m, 1H), 1.39–1.36 (m, 1H), 1.35–1.32 (m, 1H), 1.30–1.21 (m, 1H), 1.14 (s,3H), 1.02 (s, 3H). ^13^C NMR (101 MHz, CDCl_3_) δ 151.2, 149.8, 144.2, 129.0, 121.4, 84.9, 67.1, 53.7, 41.2, 37.0, 36.7, 35.5, 33.5, 31.7, 30.0, 27.9, 27.8, 26.5, 23.2, 21.6, 21.0, 19.9. RMS-ESI (*m*/*z*): [M + H]^+^ calcd for C_24_H_34_NO_2_^+^: 368.2584, found: 368.2586. m.p.: 175–176 °C.

(3*S*,5*R*,8*R*,9*S*,10*S*,13*R*,14*R*)-10,13-dimethyl-17-(pyridin-3-yl)-1,2,3,4,5,6,7,8,9,10,11,12,13,15 -tetradecahydro-14*H*-cyclopenta[*a*]phenanthrene-3,14-diol (**2-1b**). White solid, yield in 30% for two steps. ^1^H NMR (600 MHz, CDCl_3_) δ = 8.62 (d, *J* = 2.3 Hz, 1H), 8.49 (dd, *J* = 4.8, 1.6 Hz, 1H), 7.64 (dt, *J* = 7.9, 1.9 Hz, 1H), 7.24 (dd, *J* = 7.9, 4.9 Hz, 1H), 5.99 (dd, *J* = 3.2, 1.8 Hz, 1H), 4.13 (t, *J* = 2.9 Hz, 1H), 2.49 (dd, *J* = 16.5, 1.8 Hz, 1H), 2.34 (dd, *J* = 16.5, 3.2 Hz, 1H), 2.24–2.16 (m, 2H), 2.10 (td, *J* = 14.0, 3.1 Hz, 1H), 2.00–1.93 (m, 2H), 1.91 (s, 1H), 1.79–1.73 (m, 1H), 1.69–1.68 (m, 1H), 1.67–1.66 (m, 1H), 1.66–1.63 (m, 1H), 1.52–1.48 (m, 2H), 1.47–1.34 (m, 4H), 1.28–1.25 (m, 1H), 1.11 (s, 3H), 1.02 (s, 3H). ^13^C NMR (101 MHz, CDCl_3_) δ = 150.5, 148.5, 148.2, 134.1, 132.7, 127.1, 123.2, 84.9, 67.3, 54.0, 41.3, 37.2, 36.9, 35.7, 33.7, 31.9, 30.1, 28.1, 27.9, 26.7, 23.4, 21.7, 21.0, 20.1. HRMS-ESI (*m*/*z*): [M + H]^+^ calcd for C_24_H_34_NO_2_^+^: 368.2584, found: 368.2589. m.p.: 182–183 °C.

(3*S*,5*R*,8*R*,9*S*,10*S*,13*R*,14*R*)-10,13-dimethyl-17-(pyrimidin-5-yl)-1,2,3,4,5,6,7,8,9,10,11, 2,13,15-tetradecahydro-14*H*-cyclopenta[*a*]phenanthrene-3,14-diol (**2-1c**). White solid, yield in 44% for two steps. [α]_D_^23.3^ = 46.00 (c 0.200, MeOH). ^1^H NMR (400 MHz, CDCl_3_) δ = 9.10 (s, 1H), 8.73 (s, 2H), 6.10 (dd, *J* = 3.3, 1.8 Hz, 1H), 4.14 (d, *J* = 3.0 Hz, 1H), 2.53 (dd, *J* = 16.8, 1.9 Hz, 1H), 2.37 (dd, *J* = 16.7, 3.3 Hz, 1H), 2.21 (td, *J* = 12.1, 5.4 Hz, 2H), 2.09 (td, *J* = 14.0, 3.0 Hz, 1H), 2.02–1.91 (m, 2H), 1.80–1.74 (m, 1H), 1.70–1.61 (m, 3H), 1.53–1.47 (m, 3H), 1.45–1.43 (m, 1H), 1.42–1.33 (m, 3H), 1.29–1.24 (m, 1H), 1.12 (s, 3H), 1.03 (s, 3H).

^13^C NMR (101 MHz, CDCl_3_) δ 157.3, 154.5, 147.3, 130.6, 129.0, 84.7, 67.1, 53.9, 41.4, 37.1, 36.7, 35.6, 33.5, 31.8, 30.0, 27.9, 27.8, 26.5, 23.2, 21.6, 20.9, 19.9. HRMS-ESI (*m*/*z*): [M + H]^+^ calcd for C_23_H_33_N_2_O^+^: 369.2537, found: 369.2535. m.p.: 197–198 °C.

(3*S*,5*R*,8*R*,9*S*,10*S*,13*R*,14*R*)-17-(isoquinolin-6-yl)-10,13-dimethyl-1,2,3,4,5,6,7,8,9,10,11,12, 13,15-tetradecahydro-14*H*-cyclopenta[*a*]phenanthrene-3,14-diol (**2-1d**). White solid, yield in 44% for two steps. ^1^H NMR (400 MHz, CDCl_3_) δ = 9.22 (s, 1H), 8.49 (d, *J* = 5.6 Hz, 1H), 7.92 (s, 1H), 7.75 (d, *J* = 3.6 Hz, 2H), 7.61 (d, *J* = 5.7 Hz, 1H), 6.09 (s, 1H), 4.14 (s, 1H), 2.58–2.50 (m, 1H), 2.42–2.34 (m, 1H), 2.33–2.19 (m, 2H),2.17–2.07 (m, 1H), 2.05–1.97 (m, 2H), 1.86–1.70 (m, 3H), 1.68 (d, *J* = 4.2 Hz, 1H), 1.56–1.46 (m, 4H), 1.45–1.41 (m, 1H), 1.40–1.34 (m, 2H),1.31–1.27 (m, 1H), 1.21 (s, 3H), 1.04 (s, 3H). ^13^C NMR (101 MHz, CDCl_3_) δ 152.7, 142.9, 135.9, 134.9, 130.1, 128.7, 126.9, 126.4, 124.6, 120.2, 84.9, 67.1, 54.0, 41.2, 37.1, 36.8, 35.6, 33.6, 31.8, 30.0, 28.3, 27.8, 26.6, 23.3, 21.6, 21.1, 20.0. HRMS-ESI (*m*/*z*): [M + H]^+^ calcd for C_28_H_36_NO_2_^+^: 418.2741, found: 418.2746. m.p.: 157–158 °C.

(3*S*,5*R*,8*R*,9*S*,10*S*,13*R*,14*R*)-17-(6-fluoropyridin-3-yl)-10,13-dimethyl-1,2,3,4,5,6,7,8,9,10,11, 12,13,15-tetradecahydro-14*H*-cyclopenta[*a*]phenanthrene-3,14-diol. (**2-1e**). White solid, yield in 45% for two steps. ^1^H NMR (400 MHz, CDCl_3_) δ = 8.10 (d, *J* = 4.6 Hz, 1H), 7.64 (t, *J* = 8.4 Hz, 1H), 7.15 (t, *J* = 6.1 Hz, 1H), 6.03 (d, *J* = 1.8 Hz, 1H), 4.13 (s, 1H), 2.52 (d, *J* = 16.4 Hz, 1H), 2.43–2.33 (m, 1H), 2.27–2.13 (m, 2H), 2.13–2.05 (m, 1H), 2.00–1.92 (m, 2H), 1.79–1.69 (m, 2H), 1.68–1.61 (m, 2H), 1.51–1.43 (m, 3H), 1.42–1.33 (m, 4H), 1.28–1.23 (m, 1H), 1.10 (s, 3H), 1.01 (s, 3H). ^13^C NMR (101 MHz, CDCl_3_) δ 162.2, 159.8, 145.9, 145.8, 145.7, 145.6, 139.8, 139.8, 130.5, 130.4, 121.0, 121.0, 119.8, 119.5, 84.4, 67.1, 54.8, 41.5, 37.2, 36.7, 35.6, 33.6, 31.9, 30.0, 27.8, 27.4, 27.3, 26.6, 23.2, 21.6, 20.8, 19.9. HRMS-ESI (*m*/*z*): [M + H]^+^ calcd for C_24_H_33_FNO_2_^+^: 386.2490, found: 386.2493. m.p.: 156–157 °C.

(3*S*,5*R*,8*R*,9*S*,10*S*,13*R*,14*R*)-17-(4-chlorophenyl)-10,13-dimethyl-1,2,3,4,5,6,7,8,9,10,11,12, 13,15-tetradecahydro-14*H*-cyclopenta[*a*]phenanthrene-3,14-diol (**2-1f**). White solid, yield in 62% for two steps. ^1^H NMR (400 MHz, CDCl_3_) δ = 7.28 (s, 4H), 5.89 (s, 1H), 4.13 (s, 1H), 2.50–2.41 (m, 1H), 2.34–2.27 (m, 1H), 2.26–2.12 (m, 2H), 2.13–2.04 (m, 1H), 1.99–1.91 (m, 2H), 1.78–1.71 (m, 1H), 1.70–1.62 (m, 3H), 1.51–1.45 (m, 3H), 1.43–1.40 (m, 1H), 1.40–1.31 (m, 3H), 1.28–1.23 (m,1H), 1.10 (s, 3H), 1.02 (s, 3H). ^13^C NMR (101 MHz, CDCl_3_) δ 152.4, 135.3, 133.1, 128.4, 128.2, 125.6, 84.8, 67.2, 53.8, 41.0, 37.1, 36.8, 35.6, 33.6, 31.8, 30.0, 28.0, 27.8, 26.6, 23.3, 21.6, 20.9, 20.0. HRMS-ESI (*m*/*z*): [M + H]^+^ calcd for C_25_H_34_ClO_2_^+^: 401.2242, found: 401.2240. m.p.: 114–115 °C.

4-((3*S*,5*R*,8*R*,9*S*,10*S*,13*R*,14*R*)-3,14-dihydroxy-10,13-dimethyl-2,3,4,5,6,7,8,9,10,11,12,13,14, 15-tetradecahydro-1*H*-cyclopenta[*a*]phenanthren-17-yl)benzaldehyde (**2-1g**). White solid, yield in 41% for two steps. ^1^H NMR (400 MHz, CDCl_3_) δ = 9.99 (s, 1H), 7.82 (d, J = 8.1 Hz, 2H), 7.53 (d, J = 8.0 Hz, 2H), 6.09 (t, J = 2.6 Hz, 1H), 4.13 (s, 1H), 2.55–2.46 (m, 1H), 2.39–2.32 (m, 1H), 2.27–2.15 (m, 2H), 2.15–2.06 (m, 1H), 2.03–1.92 (m, 2H), 1.79–1.74 (m, 1H), 1.73–1.60 (m, 4H), 1.53–1.46 (m,3H), 1.45–1.41 (m, 1H), 1.41–1.33 (m, 2H), 1.29–1.25 (m, 1H), 1.16 (s,3H), 1.03 (s, 3H). ^13^C NMR (101 MHz, CDCl_3_) δ 191.8, 152.6, 143.1, 135.1, 129.8, 128.3, 127.3, 84.9, 67.1, 53.9, 41.3, 37.1, 36.7, 35.6, 33.6, 31.8, 30.0, 28.0, 27.8, 26.6, 23.3, 21.6, 21.1, 20.0. HRMS-ESI (*m*/*z*): [M + H]^+^ calcd for C_26_H_35_O_3_^+^: 395.2581, found: 395.2591. m.p.: 164–165 °C.

(3*S*,5*R*,8*R*,9*S*,10*S*,13*R*,14*R*)-17-(benzo[*d*][1,3]dioxol-5-yl)-10,13-dimethyl-1,2,3,4,5,6,7,8,9, 10,11,12,13,15-tetradecahydro-14*H*-cyclopenta[*a*]phenanthrene-3,14-diol (**2-1h**). White solid, yield in 50% for two steps. ^1^H NMR (400 MHz, CDCl_3_) δ = 6.88–6.82 (m, 2H), 6.79–6.72 (m, 1H),5.95 (s, 2H), 5.79 (dd, J = 3.3, 1.8 Hz, 1H), 4.13 (s, 1H), 2.47–2.37 (m, 1H), 2.31–2.24 (m, 1H), 2.22–2.13 (m, 2H), 2.12–2.06 (m, 1H), 2.01–1.89 (m, 2H), 1.78–1.71 (m, 1H), 1.70–1.63 (m, 3H), 1.54–1.44 (m, 4H),1.43–1.40 (m, 1H), 1.39–1.33 (m, 2H), 1.28–1.22 (m, 1H), 1.09 (s, 3H),1.02 (s, 3H). ^13^C NMR (101 MHz, CDCl_3_) δ 153.1, 147.5, 146.9, 131.0, 124.1, 120.4, 108.1, 107.5, 101.0, 84.8, 67.2, 53.8, 40.8, 37.1, 36.8, 35.6, 33.6, 31.8, 30.0, 28.2, 27.8, 26.6, 23.3, 21.6, 20.9, 20.0. HRMS-ESI (*m*/*z*): [M + H]^+^ calcd for C26H35O3^+^: 395.2581, found: 395.2589. m.p.: 119–120 °C.

(3*S*,5*R*,8*R*,9*S*,10*S*,13*R*,14*R*)-10,13-dimethyl-17-(4-(trifluoromethyl)phenyl)-1,2,3,4,5,6,7,8,9, 10,11,12,13,15-tetradecahydro-14*H*-cyclopenta[*a*]phenanthrene-3,14-diol (**2-1i**). White solid, yield in 55% for two steps. ^1^H NMR (400 MHz, CDCl_3_) δ = 7.56 (d, *J* = 8.2 Hz, 2H), 7.46 (d, *J* = 8.1 Hz, 2H), 6.00 (s, 1H), 4.13 (t, *J* = 2.9 Hz, 1H), 2.53–2.44 (m, 1H), 2.38–2.29 (m, 1H), 2.27–2.13 (m, 2H), 2.13–2.04 (m, 1H), 2.02–1.92 (m, 2H), 1.80–1.72 (m, 1H), 1.71–1.62 (m, 3H), 1.54–1.45 (m, 4H), 1.43 (d, *J* = 3.8 Hz, 1H), 1.41–1.34 (m, 2H), 1.29–1.23 (m, 1H), 1.13 (s, 3H), 1.03 (s, 3H). ^13^C NMR (101 MHz, CDCl_3_) δ 152.4, 140.4, 127.2, 127.1, 125.2, 125.2, 125.1, 125.1, 84.9, 67.1, 53.9, 41.1, 37.1, 36.7, 35.6, 33.6, 31.8, 30.0, 28.0, 27.8, 26.6, 23.2, 21.6, 21.0, 20.0. ^19^F NMR (376 MHz, CDCl_3_) δ = −62.52. HRMS-ESI (*m*/*z*): [M + NH_4_]^+^ calcd for C_26_H_37_F_3_NO_2_^+^: 452.2771, found: 452.2791. m.p.: 140–141 °C.

4-((3*S*,5*R*,8*R*,9*S*,10*S*,13*R*,14*R*)-3,14-dihydroxy-10,13-dimethyl-2,3,4,5,6,7,8,9,10,11,12,13,14, 15-tetradecahydro-1*H*-cyclopenta[*a*]phenanthren-17-yl)benzonitrile (**2-1j**). White solid, yield in 48% for two steps. ^1^H NMR (400 MHz, CDCl_3_) δ = 7.59 (d, *J* = 8.1 Hz, 2H), 7.46 (d, *J* = 8.0 Hz, 2H), 6.06 (t, *J* = 1.8 Hz, 1H), 4.13 (s, 1H), 2.54–2.45 (m, 1H), 2.38–2.30 (m, 1H), 2.25–2.11 (m, 2H), 2.10–2.03 (m, 1H), 2.02–1.91 (m, 2H), 1.76 (d, *J* = 13.5 Hz, 1H), 1.71–1.62 (m, 3H), 1.57 (s, 1H), 1.53–1.46 (m, 3H), 1.43 (d, *J* = 4.6 Hz, 1H), 1.40–1.33 (m, 2H), 1.26 (d, *J* = 14.0 Hz, 1H), 1.13 (s, 3H), 1.02 (s, 3H). ^13^C NMR (101 MHz, CDCl_3_) δ 152.1, 141.5, 132.1, 128.5, 127.4, 119.0, 110.7, 84.9, 67.1, 53.9, 41.3, 37.1, 36.7, 35.5, 33.5, 31.7, 30.0, 28.0, 27.8, 26.5, 23.2, 21.6, 21.0, 19.9. HRMS-ESI (*m*/*z*): [M + Na]^+^ calcd for C_26_H_33_NO_2_Na^+^: 414.2404, found: 414.2412. m.p.: 194–195 °C.

(3*S*,5*R*,8*R*,9*S*,10*S*,13*R*,14*R*)-10,13-dimethyl-17-phenyl-1,2,3,4,5,6,7,8,9,10,11,12,13,15-tetrad -ecahydro-14*H*-cyclopenta[*a*]phenanthrene-3,14-diol (**2-1k**). White solid, yield in 50% for two steps. ^1^H NMR (400 MHz, CDCl_3_) δ = 7.38 (d, *J* = 6.9 Hz, 2H), 7.33 (t, *J* = 7.2 Hz, 2H), 7.29 (d, *J* = 6.4 Hz, 1H), 5.92 (dd, *J* = 3.3, 1.8 Hz, 1H), 4.15 (s, 1H), 2.48 (dd, *J* = 16.3, 1.8 Hz, 1H), 2.32 (dd, *J* = 16.3, 3.2 Hz, 1H), 2.28–2.17 (m, 2H), 2.16–2.09 (m, 1H), 2.03–1.94 (m, 2H), 1.80–1.64 (m, 4H), 1.53–1.47 (m, 3H), 1.46–1.43 (m, 1H), 1.43–1.34 (m, 3H), 1.30–1.25 (m, 1H), 1.15 (s, 3H), 1.05 (s, 3H). ^13^C NMR (101 MHz, CDCl_3_) δ 153.5, 136.9, 128.2, 127.2, 126.9, 125.1, 84.9, 67.2, 53.8, 41.0, 37.1, 36.8, 35.6, 33.6, 31.8, 30.0, 28.1, 27.8, 26.6, 23.3, 21.6, 20.9, 20.0. HRMS-ESI (*m*/*z*): [M + Na]^+^ calcd for C_25_H_34_O_2_Na^+^: 389.2451, found: 389.2457. m.p.: 134–135 °C.

(3*S*,5*R*,8*R*,9*S*,10*S*,13*R*,14*R*)-17-(4-fluorophenyl)-10,13-dimethyl-1,2,3,4,5,6,7,8,9,10,11,12, 13,15-tetradecahydro-14*H*-cyclopenta[*a*]phenanthrene-3,14-diol (**2-1l**). White solid, yield in 56% for two steps. ^1^H NMR (400 MHz, methanol-*d*_4_) δ = 7.38 (dd, *J* = 8.0, 5.6 Hz, 2H), 7.01 (t, *J* = 8.8 Hz, 2H), 5.84–5.78 (m, 1H), 4.05 (s, 1H), 2.44 (d, *J* = 16.3 Hz, 1H), 2.31–2.20 (m, 2H), 2.20–2.07 (m, 2H), 2.07–1.95 (m, 2H), 1.81–1.73 (m, 1H), 1.70–1.59 (m, 3H), 1.49–1.45 (m, 4H), 1.39–1.28 (m, 3H), 1.27–1.20 (m, 1H), 1.11 (s, 3H), 1.03 (s, 3H). ^13^C NMR (101 MHz, methanol-*d*_4_) δ 163.3, 160.9, 151.9, 133.5, 133.5, 128.6, 128.5, 124.3, 124.3, 114.4, 114.2, 84.7, 66.5, 66.4, 53.1, 48.2, 48.0, 47.8, 47.6, 47.4, 47.2, 47.0, 40.0, 37.5, 36.7, 35.2, 33.0, 31.6, 29.7, 27.4, 27.1, 26.5, 22.4, 21.2, 20.3, 19.7. HRMS-ESI (*m*/*z*): [M + Na]^+^ calcd for C_25_H_33_FO_2_Na^+^: 407.2357, found: 407.2360. m.p.: 151–152 °C.

*Part 3. Preparation and characterization of* **2-3a**~**2-3l**.

Pd/C (116.4 mg, 80% *w*/*w*, wetted with 55% water) was added to a stirred solution of **2-1a**~**2-1e** (0.218 mmol) in MeOH (1.1 mL). The reaction mixture was allowed to stir at room temperature under H_2_ atmosphere for 10 h before it was filtered through a pad of Celite and washed with EtOAc (100 mL). The solvent was evaporated under vacuum, and the residue was purified by flash column chromatography with EtOAc:petroleum ether (2:1) to give compound **2-2a**~**2-2e**.

MsOH (35 μL, 0.54 mmol) at −40 °C was added to a stirred solution of **2-2a**~**2-2e** (0.028 mmol) in cyclpentyl methyl ether (207.6 mg, 0.24 mL). The mixture was allowed to stir at that temperature for 10 h before it was quenched with Et_3_N (4.0 mL). The resultant mixture was evaporated under vacuum, and the residue was purified by flash column chromatography with EtOAc:petroleum ether (1:2) to give **2-3a**~**2-3e**.

(3*S*,5*R*,8*R*,9*S*,10*S*,13*R*,14*R*)-10,13-dimethyl-17-(pyridin-4-yl)hexadecahydro-14*H*-cyclopen -ta[*a*]phenanthrene-3,14-diol (**2-2a**). White solid, yield in 70%. ^1^H NMR (400 MHz, CDCl_3_) δ = 8.47 (d, *J* = 5.0 Hz, 2H), 7.13 (d, *J* = 5.0 Hz, 2H), 4.13 (s, 1H), 3.45 (t, *J* = 9.3 Hz, 1H), 2.19–1.89 (m, 4H), 1.88–1.71 (m, 5H), 1.71–1.59 (m, 2H), 1.57–1.44 (m, 3H), 1.47–1.37 (m, 2H), 1.36 (s, 1H), 1.30–1.12 (m, 4H), 0.97 (s, 3H), 0.55 (s, 3H). ^13^C NMR (101 MHz, CDCl_3_) δ 151.1, 149.0, 124.4, 85.3, 67.0, 50.9, 48.8, 39.0, 36.5, 35.5, 33.7, 33.4, 32.5, 30.1, 30.0, 27.8, 26.4, 23.9, 23.6, 21.3, 19.8, 16.8. HRMS-ESI (*m*/*z*): [M + H]^+^ calcd for C_24_H_36_NO_2_^+^: 370.2741, found: 370.2744. m.p.: 190–191 °C.

(3*S*,5*R*,8*R*,9*S*,10*S*,13*R*,14*R*)-10,13-dimethyl-17-(pyridin-4-yl)-2,3,4,5,6,7,8,9,10,11,12,13,16, 17-tetradecahydro-1*H*-cyclopenta[*a*]phenanthren-3-ol (**2-3a**). White solid, yield in 48%. ^1^H NMR (400 MHz, CDCl_3_) δ = 8.51 (s, 2H), 7.16 (d, *J* = 5.3 Hz, 2H), 5.30 (s, 1H), 4.13 (t, *J* = 2.8 Hz, 1H), 3.15–3.06 (m, 1H), 2.79–2.67 (m, 1H), 2.51–2.41 (m, 1H), 2.11–2.02 (m, 1H), 1.99–1.88 (m, 3H), 1.79–1.69 (m, 2H), 1.63–1.59 (m, 1H), 1.58–1.52 (m, 3H), 1.49–1.42 (m, 4H), 1.39–1.34 (m, 1H), 1.31–1.25 (m, 2H), 0.97 (s, 3H), 0.58 (s, 3H). ^13^C NMR (101 MHz, CDCl_3_) δ 154.2, 150.9, 149.2, 124.1, 116.6, 67.0, 58.3, 48.9, 41.2, 39.8, 36.4, 35.4, 35.4, 33.8, 33.4, 29.8, 28.0, 26.3, 24.1, 23.6, 21.8, 18.9. HRMS-ESI (*m*/*z*): [M + H]^+^ calcd for C_24_H_34_NO^+^:352.2635, found: 352.2640. m.p.: 191–192 °C.

(3*S*,5*R*,8*R*,9*S*,10*S*,13*R*,14*R*)-10,13-dimethyl-17-(pyridin-3-yl)hexadecahydro-14*H*-cyclopen -ta[*a*]phenanthrene-3,14-diol (**2-2b**). White solid, yield in 72%. ^1^H NMR (400 MHz, CDCl_3_) δ = 8.49–8.40 (m, 2H), 7.56 (d, *J* = 7.9 Hz, 1H), 7.25–7.20 (m, 1H), 4.13 (s, 1H), 3.48 (t, *J* = 9.4 Hz, 1H), 2.18–2.06 (m, 2H), 2.07–2.01 (m, 1H), 2.00–1.91 (m, 3H), 1.87–1.82 (m, 1H), 1.81–1.71 (m, 3H), 1.70–1.59 (m, 2H), 1.54–1.50 (m, 1H), 1.50–1.45 (m, 2H), 1.44 (d, *J* = 9.3 Hz, 1H), 1.41–1.36 (m, 2H), 1.35 (s, 1H), 1.29–1.18 (m, 3H), 0.97 (s, 3H), 0.57 (s, 3H). ^13^C NMR (101 MHz, CDCl_3_) δ 150.2, 147.1, 136.9, 136.4, 122.9, 85.3, 67.0, 49.0, 48.6, 39.1, 36.5, 35.5, 33.7, 33.5, 32.5, 30.0, 29.9, 27.8, 26.4, 24.4, 23.6, 21.3, 19.7, 16.7. HRMS-ESI (*m*/*z*): [M + H]^+^ calcd for C_24_H_36_NO_2_^+^: 370.2741, found: 370.2745. m.p.: 180–181 °C.

(3*S*,5*R*,8*R*,9*S*,10*S*,13*R*,14*R*)-10,13-dimethyl-17-(pyridin-3-yl)-2,3,4,5,6,7,8,9,10,11,12,13,16, 17-tetradecahydro-1*H*-cyclopenta[*a*]phenanthren-3-ol (**2-3b**). White solid, yield in 50%. ^1^H NMR (400 MHz, CDCl_3_) δ = 8.48 (d, *J* = 10.9 Hz, 2H), 7.57 (d, *J* = 8.6 Hz, 1H), 7.25–7.20 (m, 1H), 5.30 (s, 1H), 4.12 (s, 1H), 3.17–3.07 (m, 1H), 2.79–2.66 (m, 1H), 2.54–2.42 (m, 1H), 2.10–2.04 (m, 1H), 2.03–1.94 (m, 2H), 1.89–1.83 (m, 1H), 1.81–1.68 (m, 2H), 1.63–1.52 (m, 4H), 1.50–1.40 (m, 4H), 1.39–1.33 (m, 1H), 1.30–1.24 (m, 2H), 0.97 (s, 3H), 0.59 (s, 3H). ^13^C NMR (101 MHz, CDCl_3_) δ 154.4, 150.1, 147.5, 135.9, 123.0, 116.7, 67.0, 56.5, 48.7, 41.0, 39.8, 36.4, 35.5, 35.4, 34.2, 33.4, 29.8, 28.0, 26.3, 24.1, 23.6, 21.7, 19.1. HRMS-ESI (*m*/*z*): [M + H]^+^ calcd for C_24_H_34_NO^+^: 352.2635, found: 352.2638. m.p.: 175–176 °C.

(3*S*,5*R*,8*R*,9*S*,10*S*,13*R*,14*R*)-10,13-dimethyl-17-(pyrimidin-5-yl)hexadecahydro-14*H*-cyclo-penta[*a*]phenanthrene-3,14-diol (**2-2c**). White solid, yield in 78%. [α]_D_^23.2^ = −18.57 (c 0.140, MeOH). ^1^H NMR (400 MHz, CDCl_3_) δ = 9.06 (s, 1H), 8.59 (s, 2H), 4.14 (s, 1H), 3.47 (t, *J* = 9.5 Hz, 1H), 2.19–2.09 (m, 2H), 2.06–1.91 (m, 3H), 1.88–1.81 (m, 2H), 1.81–1.73 (m, 2H), 1.69 (t, *J* = 6.5 Hz, 1H), 1.67–1.61 (m, 2H), 1.63–1.58 (m, 1H), 1.53–1.50 (m, 1H), 1.49–1.45 (m, 2H), 1.44–1.41 (m, 1H), 1.41–1.37 (m, 2H), 1.36–1.32 (m, 1H), 1.30–1.21 (m, 2H), 1.20–1.14 (m, 1H), 0.97 (s, 3H), 0.60 (s, 3H) ppm. ^13^C NMR (101 MHz, CDCl_3_) δ 157.0, 156.8, 134.5, 85.1, 67.0, 48.7, 47.1, 39.0, 36.5, 35.5, 33.7, 33.4, 32.5, 30.0, 29.8, 27.8, 26.4, 24.1, 23.5, 21.3, 19.7, 16.7. HRMS-ESI (*m*/*z*): [M + H]^+^ calcd for C_23_H_35_N_2_O_2_^+^: 371.2693, found: 371.2700. m.p.: 180–181 °C.

(3*S*,5*R*,8*R*,9*S*,10*S*,13*R*,14*R*)-10,13-dimethyl-17-(pyrimidin-5-yl)-2,3,4,5,6,7,8,9,10,11,12,13, 16,17-tetradecahydro-1*H*-cyclopenta[*a*]phenanthren-3-ol (**2-3c**). White solid, yield in 55%. [α]_D_^23.2^ = −5.00 (c 0.040, MeOH). ^1^H NMR (400 MHz, CDCl_3_) δ = 9.07 (s, 1H), 8.61 (s, 2H), 5.30 (s, 1H), 4.11 (s, 1H), 3.10–3.02 (m, 1H), 2.79–2.67 (m, 1H), 2.56–2.45 (m, 1H), 2.12–2.04 (m, 1H), 2.01–1.91 (m, 2H), 1.85–1.75 (m, 2H), 1.73–1.67 (m, 1H), 1.60–1.51 (m, 4H), 1.49–1.40 (m, 4H), 1.39–1.32 (m, 1H), 1.30–1.24 (m, 2H), 0.96 (s, 3H), 0.62 (s, 3H). HRMS-ESI (*m*/*z*): [M + H]^+^ calcd for C_23_H_33_N_2_O^+^: 353.2587, found: 353.2598. m.p.: 131–132 °C.

(3*S*,5*R*,8*R*,9*S*,10*S*,13*R*,14*R*)-17-(isoquinolin-6-yl)-10,13-dimethylhexadecahydro-14*H*-cyclo -penta[*a*]phenanthrene-3,14-diol (**2-2d**). White solid, yield in 75%. ^1^H NMR (400 MHz, CDCl_3_) δ = 9.22 (s, 1H), 8.47 (d, *J* = 5.7 Hz, 1H), 7.80 (s, 1H), 7.74 (d, *J* = 8.5 Hz, 1H), 7.67–7.55 (m, 2H), 4.15 (s, 1H), 3.69 (t, *J* = 9.4 Hz, 1H), 2.38–2.23 (m, 1H), 2.23–2.12 (m, 1H), 2.09–2.02 (m, 1H), 2.01–1.93 (m, 2H), 1.91–1.82 (m, 3H), 1.79–1.62 (m, 4H), 1.50 (d, *J* = 12.1 Hz, 3H), 1.41 (t, *J* = 6.7 Hz, 3H), 1.34 (d, *J* = 5.6 Hz, 1H), 1.25–1.16 (m, 2H), 0.97 (s, 3H), 0.59 (s, 3H). ^13^C NMR (101 MHz, CDCl_3_) δ 152.3, 142.3, 141.0, 134.5, 132.8, 128.7, 126.3, 125.5, 120.1, 85.4, 67.0, 51.5, 48.9, 39.1, 36.6, 35.5, 33.8, 33.5, 32.5, 30.2, 30.1, 27.8, 26.4, 24.6, 23.6, 21.3, 19.8, 16.9. HRMS-ESI (*m*/*z*): [M + H]^+^ calcd for C_28_H_38_NO_2_^+^: 420.2897, found: 420.2900. m.p.: 137–138 °C.

(3*S*,5*R*,8*R*,9*S*,10*S*,13*R*,14*R*)-17-(isoquinolin-6-yl)-10,13-dimethyl-2,3,4,5,6,7,8,9,10,11,12,13, 16,17-tetradecahydro-1*H*-cyclopenta[*a*]phenanthren-3-ol (**2-3d**). White solid, yield in 48%. ^1^H NMR (400 MHz, CDCl_3_) δ = 9.23 (s, 1H), 8.48 (d, *J* = 5.7 Hz, 1H), 7.82 (s, 1H), 7.76 (d, *J* = 8.5 Hz, 1H), 7.67–7.60 (m, 2H), 5.35 (s, 1H), 4.14 (s, 1H), 3.38–3.30 (m, 1H), 2.94–2.82 (m, 1H), 2.62–2.52 (m, 1H), 2.13–1.93 (m, 4H), 1.82–1.70 (m, 2H), 1.65–1.45 (m, 8H), 1.40–1.35 (m, 1H), 1.32–1.26 (m, 2H), 0.98 (s, 3H), 0.60 (s, 3H). ^13^C NMR (101 MHz, CDCl_3_) δ 154.4, 152.2, 142.1, 141.2, 134.7, 132.3, 128.8, 126.2, 125.8, 120.3, 116.8, 67.1, 59.1, 49.0, 41.3, 39.8, 36.4, 35.5, 35.4, 34.6, 33.4, 29.8, 28.0, 26.3, 24.1, 23.6, 21.8, 19.1. HRMS-ESI (*m*/*z*): [M + H]^+^ calcd for C_28_H_36_NO^+^: 402.2791, found: 402.2798. m.p.: 112–113 °C.

(3*S*,5*R*,8*R*,9*S*,10*S*,13*R*,14*R*)-17-(6-fluoropyridin-3-yl)-10,13-dimethylhexadecahydro-14*H*-cyclopenta[*a*]phenanthrene-3,14-diol (**2-2e**). White solid, yield in 67%. ^1^H NMR (400 MHz, CDCl_3_) δ = 8.03 (d, *J* = 4.9 Hz, 1H), 7.77–7.67 (m, 1H), 7.17–7.09 (m, 1H), 4.12 (t, *J* = 2.9 Hz, 1H), 3.80 (t, *J* = 9.5 Hz, 1H), 2.11–2.03 (m, 2H), 2.02–1.96 (m, 1H), 1.96–1.87 (m, 2H), 1.85–1.79 (m, 1H), 1.79–1.72 (m, 2H), 1.69–1.59 (m, 2H), 1.53–1.43 (m, 4H), 1.42–1.36 (m, 3H), 1.35–1.30 (m, 1H), 1.27–1.16 (m, 3H), 0.96 (s, 3H), 0.61 (s, 3H). ^13^C NMR (101 MHz, CDCl_3_) δ 163.8, 161.5, 144.8, 144.6, 140.4, 140.3, 123.7, 123.4, 120.8, 120.7, 85.2, 67.0, 49.2, 43.5, 43.4, 39.1, 36.5, 35.5, 33.5, 33.4, 32.5, 30.0, 29.9, 29.9, 27.8, 26.4, 24.3, 23.6, 21.2, 19.8, 17.0. HRMS-ESI (*m*/*z*): [M + H]^+^ calcd for C_24_H_35_FNO_2_^+^: 388.2646, found: 388.2648. m.p.: 109–110 °C.

(3*S*,5*R*,8*R*,9*S*,10*S*,13*R*,14*R*)-17-(6-fluoropyridin-3-yl)-10,13-dimethyl-2,3,4,5,6,7,8,9,10,11, 12,13,16,17-tetradecahydro-1H-cyclopenta[a]phenanthren-3-ol (**2-3e**). White solid, yield in 67%. ^1^H NMR (400 MHz, CDCl_3_) δ = 8.06 (d, *J* = 4.8 Hz, 1H), 7.76 (t, *J* = 8.4 Hz, 1H), 7.18–7.08 (m, 1H), 5.29 (d, *J* = 2.4 Hz, 1H), 4.12 (s, 1H), 3.48–3.39 (m, 1H), 2.77–2.65 (m, 1H), 2.51–2.39 (m, 1H), 2.12–2.06 (m, 1H), 2.03–1.93 (m, 2H), 1.88–1.73 (m, 2H), 1.72–1.66 (m, 1H), 1.63–1.51 (m, 5H), 1.49–1.42 (m, 3H), 1.40–1.33 (m, 1H), 1.30–1.21 (m, 2H), 0.97 (s, 3H), 0.64 (s, 3H). ^13^C NMR (101 MHz, CDCl_3_) δ 163.6, 161.3, 154.3, 145.0, 144.8, 140.3, 140.3, 123.6, 123.3, 120.9, 120.9, 116.5, 67.1, 50.6, 50.6, 49.3, 41.0, 41.0, 39.9, 36.4, 35.5, 35.4, 34.3, 33.3, 29.8, 28.0, 26.3, 24.1, 23.6, 21.9, 19.3. HRMS-ESI (*m*/*z*): [M + H]^+^ calcd for C_24_H_33_FNO^+^: 370.2541, found: 370.2566. m.p.: 83–84 °C.

**2-1f**~**2-1l** (0.25 mmol) was added to a stirred solution of Crabtree catalyst (100.6 mg, 0.125 mmol) in CH_2_Cl_2_ (0.84 mL). The reaction mixture was allowed to stir at room temperature under H_2_ atmosphere until the start material was consumed, then the resultant mixture was evaporated under vacuum and purified by flash column chromatography with EtOAc:petroleum ether (1:12) to give compound **2-3f**~**2-3l**.

(3*S*,5*R*,8*R*,9*S*,10*S*,13*R*,14*R*)-17-(4-chlorophenyl)-10,13-dimethyl-2,3,4,5,6,7,8,9,10,11,12,13, 16,17-tetradecahydro-1*H*-cyclopenta[*a*]phenanthren-3-ol (**2-3f**). White solid, yield in 76%. ^1^H NMR (400 MHz, CDCl_3_) δ = 7.25 (d, *J* = 5.7 Hz, 2H), 7.17 (d, *J* = 8.2 Hz, 2H), 5.29 (s, 1H), 4.12 (s, 1H), 3.14–3.04 (m, 1H), 2.73–2.61 (m, 1H), 2.50–2.39 (m, 1H), 2.09–1.94 (m, 3H), 1.88–1.82 (m, 1H), 1.80–1.68 (m, 2H), 1.63–1.50 (m, 4H), 1.48–1.39 (m, 4H), 1.36 (d, *J* = 15.3 Hz, 1H), 1.30–1.23 (m, 2H), 0.97 (s, 3H), 0.56 (s, 3H). ^13^C NMR (101 MHz, CDCl_3_) δ 154.4, 139.9, 131.7, 129.9, 128.0, 116.7, 67.1, 58.4, 48.6, 41.1, 39.8, 36.4, 35.5, 35.4, 34.6, 33.4, 29.8, 28.0, 26.3, 24.1, 23.6, 21.8, 18.9. HRMS-ESI (*m*/*z*): [M + H]^+^ calcd for C_25_H_34_ClO^+^: 385.2293, found: 385.2305. m.p.: 107–108 °C.

4-((3*S*,5*R*,8*R*,9*S*,10*S*,13*R*,14*R*)-3-hydroxy-10,13-dimethyl-2,3,4,5,6,7,8,9,10,11,12,13,16,17-tetrad -ecahydro-1*H*-cyclopenta[*a*]phenanthren-17-yl)benzaldehyde (**2-3g**). White solid, yield in 69%. ^1^H NMR (400 MHz, CDCl_3_) δ = 9.98 (s, 1H), 7.81 (d, *J* = 7.8 Hz, 2H), 7.40 (d, *J* = 8.0 Hz, 2H), 5.30 (s, 1H), 4.12 (s, 1H), 3.24–3.17 (m, 1H), 2.83–2.72 (m, 1H), 2.54–2.43 (m, 1H), 2.11–1.93 (m, 3H), 1.91–1.85 (m, 1H), 1.81–1.67 (m, 2H), 1.62–1.50 (m, 4H), 1.49–1.42 (m, 4H), 1.39–1.33 (m, 1H), 1.30–1.23 (m, 2H), 0.97 (s, 3H), 0.57 (s, 3H). ^13^C NMR (101 MHz, CDCl_3_) δ 192.1, 154.2, 149.3, 134.7, 129.4, 129.2, 116.7, 67.1, 59.2, 49.1, 41.2, 39.8, 36.4, 35.5, 35.4, 34.4, 33.4, 29.8, 28.0, 26.9, 26.3, 24.1, 23.6, 21.8, 19.0. HRMS-ESI (*m*/*z*): [M + H]^+^ calcd for C_26_H_35_O_2_^+^: 379.2632, found: 379.2627. m.p.: 130–131 °C.

(3*S*,5*R*,8*R*,9*S*,10*S*,13*R*,14*R*)-17-(benzo[*d*][1,3]dioxol-5-yl)-10,13-dimethyl-2,3,4,5,6,7,8,9,10,11,12, 13,16,17-tetradecahydro-1*H*-cyclopenta[*a*]phenanthren-3-ol (**2-3h**). White solid, yield in 81%. ^1^H NMR (400 MHz, CDCl_3_) δ = 6.75 (d, *J* = 8.1 Hz, 2H), 6.71–6.67 (m, 1H), 5.93 (s, 2H), 5.27 (s, 1H), 4.12 (t, *J* = 2.9 Hz, 1H), 3.10–3.00 (m, 1H), 2.67–2.58 (m, 1H), 2.48–2.37 (m, 1H), 2.10–1.92 (m, 3H), 1.92–1.82 (m, 1H), 1.79–1.64 (m, 2H), 1.62–1.51 (m, 4H), 1.48–1.34 (m, 5H), 1.30–1.23 (m, 2H), 0.97 (s, 3H), 0.60 (s, 3H). ^13^C NMR (101 MHz, CDCl_3_) δ 154.4, 147.2, 145.7, 135.3, 121.5, 121.4, 116.8, 116.7, 109.1, 109.0, 107.7, 107.6, 100.7, 67.1, 58.8, 48.5, 41.1, 40.8, 39.8, 36.5, 35.5, 35.4, 34.9, 33.4, 29.8, 28.0, 26.3, 24.1, 23.6, 21.8, 18.9. HRMS-ESI (*m*/*z*): [M + H]^+^ calcd for C_26_H_35_O_3_^+^: 395.2581, found: 395.2589. m.p.: 128–129 °C.

(3*S*,5*R*,8*R*,9*S*,10*S*,13*R*,14*R*)-10,13-dimethyl-17-(4-(trifluoromethyl)phenyl)-2,3,4,5,6,7,8,9, 10,11,12,13,16,17-tetradecahydro-1*H*-cyclopenta[*a*]phenanthren-3-ol (**2-3i**). White solid, yield in 78%. ^1^H NMR (400 MHz, CDCl_3_) δ = 7.54 (d, *J* = 8.1 Hz, 2H), 7.35 (d, *J* = 8.1 Hz, 2H), 5.30 (d, *J* = 2.4 Hz, 1H), 4.12 (t, *J* = 2.8 Hz, 1H), 3.22–3.14 (m, 1H), 2.79–2.70 (m, 1H), 2.53–2.42 (m, 1H), 2.12–1.93 (m, 3H), 1.91–1.84 (m, 1H), 1.81–1.67 (m, 2H), 1.65–1.59 (m, 2H), 1.57–1.52 (m, 2H), 1.51–1.42 (m, 4H), 1.39–1.34 (m, 1H), 1.31–1.25 (m, 2H), 0.97 (s, 3H), 0.57 (s, 3H). ^13^C NMR (101 MHz, CDCl_3_) δ 154.3, 145.7, 128.8, 124.8, 124.8, 124.7, 124.7, 116.7, 67.1, 58.9, 48.8, 41.1, 39.8, 36.4, 35.4, 35.4, 34.5, 33.4, 29.8, 28.0, 26.3, 24.1, 23.6, 21.8, 19.0. ^19^F NMR (376 MHz, CDCl_3_) δ = −62.29. HRMS-ESI (*m*/*z*): [M + H]^+^ calcd for C_26_H_34_F_3_O^+^: 419.2556, found: 419.2560. m.p.: 85–86 °C.

4-((3*S*,5*R*,8*R*,9*S*,10*S*,13*R*,14*R*)-3-hydroxy-10,13-dimethyl-2,3,4,5,6,7,8,9,10,11,12,13,16,17-tetradecahydro-1*H*-cyclopenta[*a*]phenanthren-17-yl)benzonitrile (**2-3j**). White solid, yield in 65%. ^1^H NMR (400 MHz, CDCl_3_) δ = 7.58 (d, *J* = 8.0 Hz, 2H), 7.34 (d, *J* = 8.1 Hz, 2H), 5.29 (s, 1H), 4.12 (t, *J* = 3.0 Hz, 1H), 3.22–3.13 (m, 1H), 2.78–2.64 (m, 1H), 2.53–2.42 (m, 1H), 2.12–1.92 (m, 3H), 1.89–1.82 (m, 1H), 1.81–1.67 (m, 2H), 1.59–1.51 (m, 4H), 1.50–1.40 (m, 4H), 1.39–1.33 (m, 1H), 1.30–1.23 (m, 2H), 0.97 (s, 3H), 0.56 (s, 3H). ^13^C NMR (101 MHz, CDCl_3_) δ 154.2, 147.4, 131.7, 129.3, 119.2, 116.6, 109.9, 67.0, 59.1, 49.1, 41.1, 39.8, 36.4, 35.4, 35.4, 34.3, 33.4, 29.8, 28.0, 26.3, 24.1, 23.6, 21.8, 19.0. HRMS-ESI (*m*/*z*): [M + Na]^+^ calcd for C_26_H_33_NONa^+^: 398.2454, found: 398.2453. m.p.: 101–102 °C.

(3*S*,5*R*,8*R*,9*S*,10*S*,13*R*,14*R*)-10,13-dimethyl-17-phenyl-2,3,4,5,6,7,8,9,10,11,12,13,16,17-tetra -decahydro-1*H*-cyclopenta[*a*]phenanthren-3-ol (**2-3k**). White solid, yield in 69%. ^1^H NMR (400 MHz, CDCl_3_) δ = 7.30 (t, *J* = 7.3 Hz, 2H), 7.26–7.18 (m, 3H), 5.31 (s, 1H), 4.12 (s, 1H), 3.15 (t, *J* = 9.0 Hz, 1H), 2.74 (t, *J* = 13.2 Hz, 1H), 2.52–2.41 (m, 1H), 2.13–2.07 (m, 1H), 2.05–1.96 (m, 2H), 1.90 (d, *J* = 10.6 Hz, 1H), 1.81–1.68 (m, 2H), 1.64–1.51 (m, 4H), 1.50–1.41 (m, 4H), 1.39–1.33 (m, 1H), 1.31–1.23 (m, 2H), 0.98 (s, 3H), 0.59 (s, 3H). ^13^C NMR (101 MHz, CDCl_3_) δ 154.4, 141.4, 128.6, 127.8, 126.1, 116.8, 67.1, 59.1, 48.6, 41.1, 39.8, 36.5, 35.5, 35.4, 34.5, 33.4, 29.8, 28.0, 26.3, 24.1, 23.6, 21.8, 19.0. HRMS-ESI (*m*/*z*): [M + H]^+^ calcd for C_25_H_35_O^+^: 351.2682, found: 351.2678. m.p.: 135–137 °C.

(3*S*,5*R*,8*R*,9*S*,10*S*,13*R*,14*R*)-17-(4-fluorophenyl)-10,13-dimethyl-2,3,4,5,6,7,8,9,10,11,12,13, 16,17-tetradecahydro-1*H*-cyclopenta[*a*]phenanthren-3-ol (**2-3l**). White solid, yield in 75%. ^1^H NMR (400 MHz, CDCl_3_) δ = 7.23–7.14 (m, 2H), 6.98 (t, *J* = 8.6 Hz, 2H), 5.30 (s, 1H), 4.12 (s, 1H), 3.15–3.06 (m, 1H), 2.71–2.61 (m, 1H), 2.49–2.40 (m, 1H), 2.10–1.93 (m, 3H), 1.88–1.81 (m, 1H), 1.79–1.65 (m, 2H), 1.62–1.51 (m, 4H), 1.48–1.35 (m, 5H), 1.32–1.24 (m, 2H), 0.97 (s, 3H), 0.56 (s, 3H). ^13^C NMR (101 MHz, CDCl_3_) δ 162.7, 160.2, 154.4, 137.0, 129.9, 129.8, 116.7, 114.7, 114.5, 67.1, 58.3, 48.4, 41.1, 39.8, 36.4, 35.5, 35.4, 34.8, 33.4, 29.8, 28.0, 26.9, 26.3, 24.1, 23.6, 21.8, 18.9. ^19^F NMR (376 MHz, CDCl_3_) δ = −117.52. HRMS-ESI (*m*/*z*): [M + H]^+^ calcd for C_25_H_34_FO^+^: 369.2588, found: 369.2579. m.p.: 99–100 °C.

*Part 4. Preparation and characterization of* **2-4a**~**2-4l**.

*N*-bromoacetamide (17.7 mg, 0.128 mmol) was added to a stirred solution of **2-3a**~**2-3l** (0.085 mmol) in acetone/ethylic acid/water (2.2 mL, 3:2:1). The mixture was allowed to stir at that temperature for 5 h before it was quenched with saturated aq. NaHCO_3_ (8 mL). The resultant mixture was extracted with EtOAc (3 × 10 mL), and the combined organic phases were washed with brine (20 mL), dried over anhydrous Na_2_SO_4_, and filtered. The solvent was evaporated under vacuum, and the residue was purified by flash column chromatography with EtOAc:petroleum ether (1:2) to give **2-4a**~**2-4l**.

(1*R*,2a*R*,3a*S*,3b*R*,5a*R*,7*S*,9a*S*,9b*S*,11a*R*)-9a,11a-dimethyl-1-(pyridin-4-yl)hexadecahydrona -phtho[1′,2′:6,7]indeno[1,7a-*b*]oxiren-7-ol (**2-4a**). White solid, yield in 40%. [α]_D_^23.1^ = 0.80 (c 0.050, MeOH). ^1^H NMR (400 MHz, CDCl_3_) δ = 8.45 (s, 2H), 7.28 (d, *J* = 5.0 Hz, 2H), 4.15 (t, *J* = 3.0 Hz, 1H), 3.58 (s, 1H), 2.81 (d, *J* = 10.0 Hz, 1H), 2.40 (dd, *J* = 15.4, 9.9 Hz, 1H), 2.21 (d, *J* = 15.3 Hz, 1H), 2.03–1.93 (m, 2H), 1.91–1.82 (m, 2H), 1.81–1.72 (m, 2H), 1.67–1.59 (m, 2H), 1.58–1.55 (m, 2H), 1.53–1.47 (m, 3H), 1.38–1.30 (m, 2H), 1.28–1.19 (m, 2H), 0.98 (s, 3H), 0.62 (s, 3H). ^13^C NMR (101 MHz, CDCl_3_) δ 154.0, 149.1, 125.7, 74.6, 66.9, 60.0, 53.6, 45.4, 40.1, 39.4, 36.0, 35.5, 33.6, 33.3, 32.8, 29.6, 27.9, 25.9, 23.8, 21.2, 20.9, 16.9. HRMS-ESI (*m*/*z*): [M + H]^+^ calcd for C_24_H_34_NO_2_^+^: 368.2584, found: 368.2588. m.p.: 120–121 °C.

(1*R*,2a*R*,3a*S*,3b*R*,5a*R*,7*S*,9a*S*,9b*S*,11a*R*)-9a,11a-dimethyl-1-(pyridin-3-yl)hexadecahydrona -phtho[1′,2′:6,7]indeno[1,7a-*b*]oxiren-7-ol (**2-4b**). White solid, yield in 48%. ^1^H NMR (400 MHz, CDCl_3_) δ = 8.43–8.31 (m, 2H), 7.88 (d, *J* = 8.1 Hz, 1H), 7.19 (dd, *J* = 8.1, 4.7 Hz, 1H), 4.14 (s, 1H), 3.57 (s, 1H), 2.84 (d, *J* = 10.1 Hz, 1H), 2.50–2.38 (m, 1H), 2.16 (d, *J* = 15.3 Hz, 1H), 2.03–1.92 (m, 2H), 1.90–1.81 (m, 2H), 1.80–1.73 (m, 2H), 1.67–1.59 (m, 2H), 1.58–1.55 (m, 1H), 1.54–1.51 (m, 2H), 1.51–1.49 (m, 1H), 1.49–1.45 (m, 1H), 1.40–1.29 (m, 2H), 1.28–1.17 (m, 2H), 0.98 (s, 3H), 0.60 (s, 3H). ^13^C NMR (101 MHz, CDCl_3_) δ 151.3, 147.1, 140.5, 137.7, 123.1, 74.7, 66.9, 60.0, 51.2, 45.4, 39.9, 39.4, 36.0, 35.5, 33.6, 33.3, 33.3, 29.6, 27.9, 25.9, 23.8, 21.2, 20.8, 17.3. HRMS-ESI (*m*/*z*): [M + H]^+^ calcd for C_24_H_34_NO_2_^+^: 368.2584, found: 368.2594. m.p.: 161–162 °C.

(1*R*,2a*R*,3a*S*,3b*R*,5a*R*,7*S*,9a*S*,9b*S*,11a*R*)-9a,11a-dimethyl-1-(pyrimidin-5-yl)hexadecahydr -onaphtho[1′,2′:6,7]indeno[1,7a-*b*]oxiren-7-ol (**2-4c**). White solid, yield in 52%. [α]_D_^23.1^ = 284.17 (c 0.120, MeOH). ^1^H NMR (400 MHz, CDCl_3_) δ = 9.02 (s, 1H), 8.71 (s, 2H), 4.15 (s, 1H), 3.61 (s, 1H), 2.77 (d, *J* = 8.6 Hz, 1H), 2.52–2.42 (m, 1H), 2.19 (d, *J* = 15.3 Hz, 1H), 2.07–1.91 (m, 2H), 1.91–1.83 (m, 1H), 1.81–1.73 (m, 2H), 1.68–1.60 (m, 2H), 1.59–1.55 (m, 3H), 1.55–1.47 (m, 4H), 1.40–1.31 (m, 2H), 1.26–1.20 (m, 1H), 0.99 (s, 3H), 0.64 (s, 3H). ^13^C NMR (101 MHz, CDCl_3_) δ 158.4, 156.4, 137.9, 74.5, 66.8, 59.7, 49.1, 45.4, 39.5, 39.4, 36.0, 35.6, 33.5, 33.3, 32.9, 29.6, 27.9, 25.8, 23.8, 21.2, 20.8, 17.6. HRMS-ESI (*m*/*z*): [M + H]^+^ calcd for C_23_H_33_N_2_O_2_^+^: 369.2537, found: 369.2534. m.p.: 155–156 °C.

(1*R*,2a*R*,3a*S*,3b*R*,5a*R*,7*S*,9a*S*,9b*S*,11a*R*)-1-(isoquinolin-6-yl)-9a,11a-dimethylhexadecahydr -onaphtho[1′,2′:6,7]indeno[1,7a-*b*]oxiren-7-ol (**2-4d**). White solid, yield in 40%. ^1^H NMR (600 MHz, CDCl_3_) δ = 9.20 (s, 1H), 8.45 (d, *J* = 5.8 Hz, 1H), 7.91–7.76 (m, 2H), 7.70 (d, *J* = 8.6 Hz, 1H), 7.60 (d, *J* = 5.7 Hz, 1H), 4.16 (t, *J* = 2.9 Hz, 1H), 3.64 (s, 1H), 3.08 (d, *J* = 9.7 Hz, 1H), 2.55–2.48 (m, 1H), 2.32 (d, *J* = 15.4 Hz, 1H), 2.04–1.98 (m, 1H), 1.95 (dd, *J* = 14.0, 2.9 Hz, 1H), 1.91–1.84 (m, 1H), 1.84–1.81 (m, 1H), 1.80–1.75 (m, 1H), 1.69–1.65 (m, 1H), 1.65–1.62 (m, 1H), 1.60–1.53 (m, 5H), 1.52–1.48 (m, 1H), 1.40–1.32 (m, 3H), 1.26–1.21 (m, 1H), 0.99 (s, 3H), 0.61 (s, 3H). ^13^C NMR (151 MHz, CDCl_3_) δ 156.7, 152.4, 144.5, 142.3, 134.3, 133.7, 128.1, 125.6, 120.1, 74.9, 66.9, 60.4, 54.5, 45.6, 40.3, 39.4, 36.1, 35.6, 33.8, 33.5, 33.4, 29.6, 28.0, 25.9, 23.8, 21.3, 20.9, 17.2. HRMS-ESI (*m*/*z*): [M + H]^+^ calcd for C_28_H_36_NO_2_^+^: 418.2741, found: 418.2756. m.p.: 150–151 °C.

(1R,2aR,3aS,3bR,5aR,7S,9aS,9bS,11aR)-1-(6-fluoropyridin-3-yl)-9a,11a-dimethylhexadeca -hydronaphtho[1′,2′:6,7]indeno[1,7a-b]oxiren-7-ol (**2-4e**). White solid, yield in 52%. ^1^H NMR (400 MHz, CDCl_3_) δ = 8.07–8.00 (m, 1H), 7.98 (d, *J* = 4.7 Hz, 1H), 7.14–7.09 (m, 1H), 4.14 (t, *J* = 2.9 Hz, 1H), 3.60 (s, 1H), 3.23 (d, *J* = 10.0 Hz, 1H), 2.46–2.34 (m, 1H), 2.23–2.17 (m, 1H), 2.03–1.92 (m, 2H), 1.90–1.81 (m, 2H), 1.81–1.72 (m, 1H), 1.65–1.56 (m, 4H), 1.53–1.46 (m, 4H), 1.38–1.30 (m, 2H), 1.28–1.18 (m, 2H), 0.98 (s, 3H), 0.66 (s, 3H). ^13^C NMR (101 MHz, CDCl_3_) δ 163.2, 160.9, 144.7, 144.5, 142.1, 142.1, 127.0, 126.7, 121.4, 121.3, 74.8, 66.9, 60.1, 45.4, 43.5, 39.4, 39.0, 36.0, 35.5, 33.5, 33.3, 32.2, 29.6, 27.9, 25.8, 23.8, 21.1, 20.8, 15.8. HRMS-ESI (*m*/*z*): [M + H]^+^ calcd for C_24_H_33_FNO_2_^+^:386.2490, found: 386.2496. m.p.: 84–85 °C.

(1*R*,2a*R*,3a*S*,3b*R*,5a*R*,7*S*,9a*S*,9b*S*,11a*R*)-1-(4-chlorophenyl)-9a,11a-dimethylhexadecahyd-ronaphtho[1′,2′:6,7]indeno[1,7a-*b*]oxiren-7-ol (**2-4f**). White solid, yield in 18%. ^1^H NMR (400 MHz, CDCl_3_) δ = 7.25 (d, *J* = 7.1 Hz, 2H), 7.18 (d, *J* = 8.2 Hz, 2H), 4.15 (s, 1H), 3.55 (s, 1H), 2.81 (d, *J* = 10.2 Hz, 1H), 2.45–2.36 (m, 1H), 2.17 (d, *J* = 15.3 Hz, 1H), 2.03–1.90 (m, 2H), 1.88–1.81 (m, 1H), 1.79–1.70 (m, 2H), 1.66–1.58 (m, 2H), 1.57–1.50 (m, 5H), 1.48–1.44 (m, 1H), 1.37–1.27 (m, 3H), 1.21 (d, *J* = 13.7 Hz, 1H), 0.98 (s, 3H), 0.60 (s, 3H). ^13^C NMR (101 MHz, CDCl_3_) δ 143.6, 131.7, 131.4, 127.7, 74.8, 66.9, 60.2, 53.7, 45.3, 40.2, 39.4, 36.1, 35.5, 33.7, 33.5, 33.3, 29.6, 27.9, 25.9, 23.8, 21.3, 20.8, 17.0. HRMS-ESI (*m*/*z*): [M + H]^+^ calcd for C_25_H_34_ClO_2_^+^: 401.2242, found: 401.2247. m.p.: 110–111 °C.

4-((1*R*,2a*R*,3a*S*,3b*R*,5a*R*,7*S*,9a*S*,9b*S*,11a*R*)-7-hydroxy-9a,11a-dimethylhexadecahydronap-htho[1′,2′:6,7]indeno[1,7a-*b*]oxiren-1-yl)benzaldehyde (**2-4g**). White solid, yield in 42%. ^1^H NMR (600 MHz, CDCl_3_) δ = 9.96 (s, 1H), 7.75 (d, *J* = 8.6 Hz, 2H), 7.49 (d, *J* = 7.8 Hz, 2H), 4.15 (t, *J* = 2.8 Hz, 1H), 3.60 (s, 1H), 2.94 (d, *J* = 8.5 Hz, 1H), 2.47–2.40 (m, 1H), 2.25 (d, *J* = 15.4 Hz, 1H), 2.02–1.96 (m, 1H), 1.95–1.91 (m, 1H), 1.90–1.83 (m, 1H), 1.80–1.74 (m, 2H), 1.66–1.58 (m, 3H), 1.57–1.52 (m, 4H), 1.51–1.47 (m, 1H), 1.40–1.27 (m, 3H), 1.24–1.19 (m, 1H), 0.98 (s, 3H), 0.60 (s, 3H). ^13^C NMR (151 MHz, CDCl_3_) δ 192.2, 152.6, 134.3, 131.0, 129.2, 74.8, 66.9, 60.2, 54.4, 45.7, 40.3, 39.4, 36.0, 35.6, 33.7, 33.3, 33.2, 29.6, 27.9, 25.9, 23.8, 21.2, 20.9, 17.0. HRMS-ESI (*m*/*z*): [M + H]^+^ calcd for C_26_H_35_O_3_^+^: 395.2581, found: 395.2565. m.p.: 150–151 °C.

(1*R*,2a*R*,3a*S*,3b*R*,5a*R*,7*S*,9a*S*,9b*S*,11a*R*)-1-(6-bromobenzo[d][1,3]dioxol-5-yl)-9a,11a-dime-thylhexadecahydronaphtho[1′,2′:6,7]indeno[1,7a-*b*]oxiren-7-ol (**2-4h**). White solid, yield in 39%. ^1^H NMR (400 MHz, Methanol-*d*_4_) δ = 7.22 (s, 1H), 6.94 (s, 1H), 5.94 (s, 2H), 4.06 (s, 1H), 3.65 (s, 1H), 3.50 (d, *J* = 10.5 Hz, 1H), 2.48–2.37 (m, 1H), 2.08 (d, *J* = 15.4 Hz, 1H), 2.02–1.92 (m, 3H), 1.91–1.82 (m, 1H), 1.80–1.70 (m, 2H), 1.65–1.58 (m, 2H), 1.57–1.46 (m, 5H), 1.35–1.27 (m, 3H), 1.24–1.16 (m, 1H), 0.99 (s, 3H), 0.67 (s, 3H). ^13^C NMR (101 MHz, MeOD) δ 147.3, 146.3, 137.6, 116.3, 111.0, 110.8, 101.6, 75.1, 66.3, 60.5, 51.2, 45.5, 39.1, 38.9, 36.0, 35.1, 33.8, 32.9, 32.7, 29.3, 27.1, 25.7, 22.9, 20.9, 20.5, 14.9. HRMS-ESI (*m*/*z*): [M + H]^+^ calcd for C_26_H_34_BrO_4_^+^: 489.1635, found: 489.1627. m.p.: 130–131 °C.

(1*R*,2a*R*,3a*S*,3b*R*,5a*R*,7*S*,9a*S*,9b*S*,11a*R*)-9a,11a-dimethyl-1-(4-(trifluoromethyl)phenyl)hex -adecahydronaphtho[1′,2′:6,7]indeno[1,7a-*b*]oxiren-7-ol (**2-4i**). White solid, yield in 41%. ^1^H NMR (400 MHz, CDCl_3_) δ = 7.49–7.40 (m, 4H), 4.15 (t, *J* = 2.9 Hz, 1H), 3.58 (s, 1H), 2.90 (d, *J* = 8.4 Hz, 1H), 2.47–2.37 (m, 1H), 2.22 (d, *J* = 15.3 Hz, 1H), 2.03–1.90 (m, 2H), 1.89–1.82 (m, 1H), 1.80–1.73 (m, 2H), 1.67–1.56 (m, 4H), 1.52–1.45 (m, 4H), 1.40–1.32 (m, 2H), 1.28–1.19 (m, 2H), 0.98 (s, 3H), 0.59 (s, 3H). ^13^C NMR (101 MHz, CDCl_3_) δ 149.1, 130.6, 124.5, 124.5, 124.5, 124.4, 74.8, 66.9, 60.2, 54.1, 45.5, 40.2, 39.4, 36.1, 35.5, 33.7, 33.3, 33.3, 29.6, 27.9, 26.9, 25.9, 23.8, 21.2, 20.9, 17.0; ^19^F NMR (376 MHz, CDCl_3_) δ = −62.23. HRMS-ESI (*m*/*z*): [M + H]^+^ calcd for C_26_H_34_F_3_O_2_^+^: 435.2505, found: 435.2511. m.p.: 100–101 °C.

4-((1*R*,2a*R*,3a*S*,3b*R*,5a*R*,7*S*,9a*S*,9b*S*,11a*R*)-7-hydroxy-9a,11a-dimethylhexadecahydronap -htho[1′,2′:6,7]indeno[1,7a-*b*]oxiren-1-yl)benzonitrile (**2-4j**). White solid, yield in 43%. ^1^H NMR (400 MHz, CDCl_3_) δ = 7.51 (d, *J* = 8.3 Hz, 2H), 7.44 (d, *J* = 8.0 Hz, 2H), 4.15 (t, *J* = 2.9 Hz, 1H), 3.58 (s, 1H), 2.89 (d, *J* = 9.7 Hz, 1H), 2.47–2.38 (m, 1H), 2.20 (d, *J* = 15.3 Hz, 1H), 2.03–1.89 (m, 2H), 1.88–1.82 (m, 1H), 1.80–1.72 (m, 2H), 1.67–1.58 (m, 2H), 1.57–1.51 (m, 5H), 1.50–1.46 (m, 1H), 1.38–1.28 (m, 3H), 1.24–1.19 (m, 1H), 0.98 (s, 3H), 0.58 (s, 3H). ^13^C NMR (101 MHz, CDCl_3_) δ 150.7, 131.5, 131.1, 119.3, 109.5, 74.7, 66.9, 60.1, 54.3, 45.7, 40.2, 39.4, 36.0, 35.5, 33.7, 33.3, 33.2, 29.6, 27.9, 25.9, 23.8, 21.2, 20.8, 17.0. HRMS-ESI (*m*/*z*): [M + Na]^+^ calcd for C_26_H_33_NO_2_Na^+^: 414.2404, found: 414.2403. m.p.: 195–196 °C.

(1*R*,2a*R*,3a*S*,3b*R*,5a*R*,7*S*,9a*S*,9b*S*,11a*R*)-9a,11a-dimethyl-1-phenylhexadecahydronaphtho -[1′,2′:6,7]indeno[1,7a-*b*]oxiren-7-ol (**2-4k**). White solid, yield in 40%. ^1^H NMR (400 MHz, CDCl_3_) δ = 7.31 (d, *J* = 7.6 Hz, 2H), 7.23 (t, *J* = 7.5 Hz, 2H), 7.13 (t, *J* = 7.2 Hz, 1H), 4.15 (t, *J* = 2.9 Hz, 1H), 3.56 (s, 1H), 2.85 (d, *J* = 8.4 Hz, 1H), 2.45–2.36 (m, 1H), 2.25 (d, *J* = 15.2 Hz, 1H), 2.03–1.91 (m, 2H), 1.90–1.81 (m, 1H), 1.80–1.71 (m, 2H), 1.66–1.59 (m, 2H), 1.57–1.52 (m, 3H), 1.51–1.46 (m, 3H), 1.41–1.32 (m, 2H), 1.32–1.24 (m, 1H), 1.25–1.18 (m, 1H), 0.98 (s, 3H), 0.60 (s, 3H). ^13^C NMR (101 MHz, CDCl_3_) δ 145.1, 130.3, 127.6, 125.6, 74.8, 67.0, 60.3, 54.3, 45.3, 40.4, 39.4, 36.1, 35.6, 33.8, 33.4, 33.4, 29.6, 27.9, 25.9, 23.8, 21.3, 20.9, 17.0. HRMS-ESI (*m*/*z*): [M + Na]^+^ calcd for C_25_H_34_O_2_Na^+^: 389.2455, found: 389.2451. m.p.: 70–71 °C.

(1*R*,2a*R*,3a*S*,3b*R*,5a*R*,7*S*,9a*S*,9b*S*,11a*R*)-1-(4-fluorophenyl)-9a,11a-dimethylhexadecahydr -onaphtho[1′,2′:6,7]indeno[1,7a-*b*]oxiren-7-ol (**2-4l**). White solid, yield in 21%. ^1^H NMR (600 MHz, CDCl_3_) δ = 7.27 (d, *J* = 6.7 Hz, 2H), 6.93–6.87 (m, 2H), 4.15 (t, *J* = 2.9 Hz, 1H), 3.55 (s, 1H), 2.83 (d, *J* = 8.7 Hz, 1H), 2.45–2.37 (m, 1H), 2.21–2.15 (m, 1H), 2.00–1.91 (m, 2H), 1.90–1.83 (m, 1H), 1.77–1.70 (m, 2H), 1.65–1.59 (m, 2H), 1.56–1.54 (m, 2H), 1.53–1.51 (m, 2H), 1.51–1.46 (m, 2H), 1.38–1.33 (m, 2H), 1.34–1.29 (m, 2H), 1.24–1.19 (m, 1H), 0.98 (s, 3H), 0.59 (s, 3H) ppm; ^13^C NMR (151 MHz, CDCl_3_) δ 162.0, 160.3, 140.7, 140.7, 131.6, 131.6, 114.3, 114.1, 74.8, 66.9, 65.7, 60.2, 53.5, 45.2, 40.2, 39.4, 36.1, 35.5, 33.7, 33.6, 33.3, 29.6, 27.9, 25.9, 23.8, 21.3, 20.8, 17.0. HRMS-ESI (*m*/*z*): [M + H]^+^ calcd for C_25_H_34_FO_2_^+^: 385.2537, found: 385.2550. m.p.: 148–149 °C.

*Part 5. Preparation and characterization of* **2-5a**~**2-5l**.

2-chloroacrylic acid (11.6 mg, 0.109 mmol), EDCI (20.1 mg, 0.109 mmol) and DMAP (9.9 mg, 0.081 mmol) were added to a stirred solution of **2-4a**~**2-4l** (0.054 mmol) in CH_2_Cl_2_ (0.54 mL). The reaction mixture was allowed to stir at room temperature under N_2_ atmosphere for 10 h. The solvent was evaporated under vacuum, and the residue was purified by flash column chromatography with EtOAc:petroleum ether (1:2) to give compound **2-5a**~**2-5l**.

(1*R*,2a*R*,3a*S*,3b*R*,5a*R*,7*S*,9a*S*,9b*S*,11a*R*)-9a,11a-dimethyl-1-(pyridin-4-yl)hexadecahydrona -phtho[1′,2′:6,7]indeno[1,7a-*b*]oxiren-7-yl 2-chloroacrylate (**2-5a**). White solid, yield in 50%. ^1^H NMR (400 MHz, CDCl_3_) δ = 8.37 (d, *J* = 5.3 Hz, 2H), 7.18 (d, *J* = 5.2 Hz, 2H), 6.44 (s, 1H), 5.93 (s, 1H), 5.16 (s, 1H), 3.52 (s, 1H), 2.73 (d, *J* = 9.9 Hz, 1H), 2.39–2.29 (m, 1H), 2.15 (d, *J* = 15.3 Hz, 1H), 1.99–1.88 (m, 2H), 1.86–1.75 (m, 1H), 1.72–1.63 (m, 3H), 1.61–1.55 (m, 3H), 1.54–1.44 (m, 4H), 1.43–1.22 (m, 3H), 1.21–1.13 (m, 1H), 0.94 (s, 3H), 0.55 (s, 3H). ^13^C NMR (101 MHz, CDCl_3_) δ 161.3, 153.8, 149.2, 132.2, 125.6, 125.3, 74.5, 73.1, 60.0, 53.5, 45.3, 40.0, 39.5, 37.0, 35.3, 33.6, 32.8, 30.5, 30.4, 25.7, 25.0, 23.9, 21.2, 20.8, 16.9. HRMS-ESI (*m*/*z*): [M + H]^+^ calcd for C_27_H_35_ClNO_3_^+^: 456.2300, found: 456.2303. m.p.: 135–136 °C.

(1*R*,2a*R*,3a*S*,3b*R*,5a*R*,7*S*,9a*S*,9b*S*,11a*R*)-9a,11a-dimethyl-1-(pyridin-3-yl)hexadecahydrona -phtho[1′,2′:6,7]indeno[1,7a-*b*]oxiren-7-yl 2-chloroacrylate (**2-5b**). White solid, yield in 53%. [α]_D_^23.0^ = 0.00 (c 0.050, MeOH). ^1^H NMR (400 MHz, CDCl_3_) δ = 8.38 (s, 2H), 7.90 (d, *J* = 8.0 Hz, 1H), 7.21 (dd, *J* = 8.0, 4.8 Hz, 1H), 6.54–6.47 (m, 1H), 5.99 (s, 1H), 5.23 (s, 1H), 3.59 (s, 1H), 2.91–2.81 (m, 1H), 2.50–2.40 (m, 1H), 2.22–2.12 (m, 1H), 2.05–1.92 (m, 2H), 1.91–1.81 (m, 1H), 1.80–1.69 (m, 3H), 1.67–1.62 (m, 3H), 1.61–1.51 (m, 4H), 1.50–1.31 (m, 3H), 1.28–1.20 (m, 1H), 1.00 (s, 3H), 0.60 (s, 3H). ^13^C NMR (101 MHz, CDCl_3_) δ 161.3, 151.0, 146.8, 140.6, 137.9, 132.2, 125.2, 74.6, 73.2, 60.0, 51.1, 45.4, 39.7, 39.6, 37.0, 35.3, 33.7, 33.2, 30.5, 30.4, 25.7, 25.0, 23.9, 21.2, 20.7, 17.2. HRMS-ESI (*m*/*z*): [M + H]^+^ calcd for C_27_H_35_ClNO_3_^+^: 456.2300, found: 456.2301. m.p.: 157–158 °C.

(1*R*,2a*R*,3a*S*,3b*R*,5a*R*,7*S*,9a*S*,9b*S*,11a*R*)-9a,11a-dimethyl-1-(pyrimidin-5-yl)hexadecahydr-onaphtho[1′,2′:6,7]indeno[1,7a-*b*]oxiren-7-yl 2-chloroacrylate (**2-5c**). White solid, yield in 58%. [α]_D_^22.7^ = 1.60 (c 0.050, MeOH). ^1^H NMR (400 MHz, CDCl_3_) δ = 9.02 (s, 1H), 8.71 (s, 2H), 6.52 (s, 1H), 6.00 (s, 1H), 5.23 (s, 1H), 3.61 (s, 1H), 2.80–2.74 (m, 1H), 2.54–2.44 (m, 1H), 2.24–2.17 (m, 1H), 2.07–1.93 (m, 2H), 1.92–1.82 (m, 1H), 1.81–1.70 (m, 3H), 1.66 (s, 1H), 1.64–1.59 (m, 4H), 1.58–1.53 (m, 2H), 1.51–1.30 (m, 3H), 1.27–1.22 (m, 1H), 1.01 (s, 3H), 0.64 (s, 3H). ^13^C NMR (101 MHz, CDCl_3_) δ 161.3, 158.3, 156.5, 132.2, 125.3, 74.3, 73.1, 59.7, 49.1, 45.4, 39.6, 39.4, 37.0, 35.3, 33.5, 32.9, 30.4, 30.4, 26.9, 25.6, 25.0, 23.8, 21.2, 20.7, 17.6. HRMS-ESI (*m*/*z*): [M + H]^+^ calcd for C_26_H_34_ClN_2_O_3_^+^: 457.2252, found: 457.2260. m.p.: 150–151 °C.

(1*R*,2a*R*,3a*S*,3b*R*,5a*R*,7*S*,9a*S*,9b*S*,11a*R*)-1-(isoquinolin-6-yl)-9a,11a-dimethylhexadecahydr -onaphtho[1′,2′:6,7]indeno[1,7a-*b*]oxiren-7-yl 2-chloroacrylate (**2-5d**). White solid, yield in 58%.[α]_D_^22.6^ = 1.33 (c 0.050, MeOH). ^1^H NMR (600 MHz, CDCl_3_) δ = 9.20 (s, 1H), 8.45 (d, *J* = 5.7 Hz, 1H), 7.85 (s, 2H), 7.71 (d, *J* = 8.5 Hz, 1H), 7.61 (d, *J* = 5.7 Hz, 1H), 6.52 (d, *J* = 1.3 Hz, 1H), 6.00 (d, *J* = 1.3 Hz, 1H), 5.24 (s, 1H), 3.66 (s, 1H), 3.11–3.06 (m, 1H), 2.57–2.50 (m, 1H), 2.32 (s, 1H), 2.06–1.95 (m, 2H), 1.91–1.81 (m, 2H), 1.77–1.71 (m, 2H), 1.69–1.63 (m, 3H), 1.61–1.53 (m, 4H), 1.46–1.34 (m, 2H), 1.28–1.24 (m, 2H), 1.01 (s, 3H), 0.61 (s, 3H). ^13^C NMR (151 MHz, CDCl_3_) δ 161.3, 152.2, 144.5, 142.0, 134.4, 133.9, 132.2, 128.5, 128.2, 125.6, 125.3, 120.2, 74.8, 73.2, 60.4, 54.4, 45.6, 40.2, 39.6, 37.0, 35.4, 33.8, 33.5, 30.5, 30.4, 25.7, 25.1, 23.9, 21.3, 20.8, 17.2. HRMS-ESI (*m*/*z*): [M + H]^+^ calcd for C_31_H_37_ClNO_3_^+^: 506.2456, found: 506.2457. m.p.: 166–167 °C.

(1*R*,2a*R*,3a*S*,3b*R*,5a*R*,7*S*,9a*S*,9b*S*,11a*R*)-1-(6-fluoropyridin-3-yl)-9a,11a-dimethylhexadeca -hydronaphtho[1′,2′,:6,7]indeno[1,7a-*b*]oxiren-7-yl 2-chloroacrylate (**2-5e**). White solid, yield in 58%. [α]_D_^22.7^ = 10.57 (c 0.140, MeOH). ^1^H NMR (600 MHz, CDCl_3_) δ = 8.03 (t, *J* = 8.7 Hz, 1H), 7.98 (s, 1H), 7.12 (t, *J* = 6.3 Hz, 1H), 6.51 (s, 1H), 6.00 (s, 1H), 5.23 (s, 1H), 3.61 (s, 1H), 3.27–3.18 (m, 1H), 2.46–2.37 (m, 1H), 2.25–2.18 (m, 1H), 2.03–1.92 (m, 2H), 1.90–1.82 (m, 2H), 1.76–1.69 (m, 2H), 1.67–1.60 (m, 3H), 1.59–1.49 (m, 5H), 1.45–1.31 (m, 2H), 1.28–1.20 (m, 2H), 1.00 (s, 3H), 0.67 (s, 3H). ^13^C NMR (151 MHz, CDCl_3_) δ 162.8, 162.8, 161.3, 144.7, 144.6, 142.1, 142.0, 132.2, 126.9, 126.8, 125.2, 121.4, 121.4, 74.7, 73.1, 60.2, 45.4, 43.5, 39.6, 38.9, 37.0, 35.3, 33.5, 32.1, 30.4, 30.4, 25.7, 25.0, 23.9, 21.2, 20.7, 15.7. HRMS-ESI (*m*/*z*): [M + H]^+^ calcd for C_27_H_34_ClFNO_3_^+^:474.2206, found: 474.2220. m.p.: 75–76 °C.

(1*R*,2a*R*,3a*S*,3b*R*,5a*R*,7*S*,9a*S*,9b*S*,11a*R*)-1-(4-chlorophenyl)-9a,11a-dimethylhexadecahydr -onaphtho[1′,2′:6,7]indeno[1,7a-*b*]oxiren-7-yl 2-chloroacrylate (**2-5f**). White solid, yield in 28%. [α]_D_^22.6^ = −22.22 (c 0.100, MeOH). ^1^H NMR (600 MHz, CDCl_3_) δ = 7.24 (s, 2H), 7.22–7.15 (m, 2H), 6.51 (d, *J* = 1.3 Hz, 1H), 6.00 (d, *J* = 1.4 Hz, 1H), 5.22 (t, *J* = 2.9 Hz, 1H), 3.56 (s, 1H), 2.85–2.80 (m, 1H), 2.45–2.39 (m, 1H), 2.22–2.14 (m, 1H), 2.03–1.93 (m, 2H), 1.89–1.83 (m, 1H), 1.76–1.69 (m, 3H), 1.66–1.59 (m, 3H), 1.58–1.55 (m, 2H), 1.54–1.51 (m, 2H), 1.50–1.46 (m, 1H), 1.45–1.38 (m, 1H), 1.35–1.31 (m, 1H), 1.25–1.21 (m, 1H), 1.00 (s, 3H), 0.60 (s, 3H). ^13^C NMR (151 MHz, CDCl_3_) δ 161.3, 143.5, 132.3, 131.7, 131.4, 127.7, 125.2, 74.7, 73.2, 60.2, 53.6, 45.3, 40.1, 39.6, 37.0, 35.3, 33.7, 33.5, 30.5, 30.4, 25.7, 25.0, 23.9, 21.3, 20.7, 17.0. HRMS-ESI (*m*/*z*): [M + NH_4_]^+^ calcd for C_28_H_38_Cl_2_NO_3_^+^: 506.2223, found: 506.2209. m.p.: 112–113 °C.

(1*R*,2a*R*,3a*S*,3b*R*,5a*R*,7*S*,9a*S*,9b*S*,11a*R*)-1-(4-formylphenyl)-9a,11a-dimethylhexadecahydr -onaphtho[1′,2′:6,7]indeno[1,7a-*b*]oxiren-7-yl 2-chloroacrylate (**2-5g**). White solid, yield in 49%. [α]_D_^22.6^ = 5.53 (c 0.130, MeOH). ^1^H NMR (400 MHz, CDCl_3_) δ = 9.96 (s, 1H), 7.75 (d, *J* = 8.0 Hz, 2H), 7.50 (d, *J* = 7.9 Hz, 2H), 6.52 (s, 1H), 6.00 (s, 1H), 5.23 (s, 1H), 3.61 (s, 1H), 2.99–2.91 (m, 1H), 2.50–2.39 (m, 1H), 2.31–2.22 (m, 1H), 2.07–1.92 (m, 2H), 1.92–1.81 (m, 1H), 1.81–1.69 (m, 3H), 1.69–1.58 (m, 4H), 1.58–1.55 (m, 2H), 1.53–1.49 (m, 1H), 1.47–1.31 (m, 3H), 1.26–1.21 (m, 2H), 1.01 (s, 3H), 0.61 (s, 3H). ^13^C NMR (101 MHz, CDCl_3_) δ 192.2, 161.3, 152.5, 134.3, 132.2, 131.0, 129.2, 125.2, 74.6, 73.2, 60.2, 54.4, 45.7, 40.2, 39.6, 37.0, 35.3, 33.7, 33.2, 30.5, 30.4, 25.7, 25.1, 23.9, 21.2, 20.8, 17.0. HRMS-ESI (*m*/*z*): [M + H]^+^ calcd for C_29_H_36_ClO_4_^+^: 483.2297, found: 483.2296. m.p.: 149–150 °C.

(1*R*,2a*R*,3a*S*,3b*R*,5a*R*,7*S*,9a*S*,9b*S*,11a*R*)-1-(6-bromobenzo[*d*][1,3]dioxol-5-yl)-9a,11a-dimet -hylhexadecahydronaphtho[1′,2′:6,7]indeno[1,7a-*b*]oxiren-7-yl 2-chloroacrylate (**2-5h**). White solid, yield in 50%. [α]_D_^22.9^ = −11.50 (c 0.040, MeOH). ^1^H NMR (400 MHz, CDCl_3_) δ = 7.27 (s, 1H), 6.92 (s, 1H), 6.51 (s, 1H), 6.00 (s, 1H), 5.92 (d, *J* = 3.1 Hz, 2H), 5.23 (s, 1H), 3.57 (s, 1H), 3.49 (d, *J* = 10.2 Hz, 1H), 2.45–2.34 (m, 1H), 2.17–2.11 (m, 1H), 2.04–1.92 (m, 3H), 1.91–1.81 (m, 1H), 1.77–1.69 (m, 2H), 1.67–1.62 (m, 2H), 1.56–1.50 (m, 4H), 1.47–1.30 (m, 3H), 1.26–1.20 (m, 2H), 1.00 (s, 3H), 0.69 (s, 3H). ^13^C NMR (101 MHz, CDCl_3_) δ 161.3, 147.3, 146.1, 137.6, 132.3, 125.2, 116.7, 111.5, 111.3, 101.5, 75.1, 73.2, 60.6, 51.2, 45.7, 39.5, 39.2, 37.0, 35.3, 33.8, 33.4, 30.5, 30.4, 25.7, 25.1, 23.9, 21.2, 20.8, 15.9. HRMS-ESI (*m*/*z*): [M + Na]^+^ calcd for C_29_H_34_BrClO_5_Na^+^: 599.1170, found: 599.1171. m.p.: 157–158 °C.

(1*R*,2a*R*,3a*S*,3b*R*,5a*R*,7*S*,9a*S*,9b*S*,11a*R*)-9a,11a-dimethyl-1-(4-(trifluoromethyl)phenyl)hex -adecahydronaphtho[1′,2′:6,7]indeno[1,7a-*b*]oxiren-7-yl 2-chloroacrylate (**2-5i**). White solid, yield in 55%. [α]_D_^22.7^ = 9.80 (c 0.100, MeOH). ^1^H NMR (400 MHz, CDCl_3_) δ = 7.50–7.40 (m, 4H), 6.51 (s, 1H), 6.00 (s, 1H), 5.23 (s, 1H), 3.59 (s, 1H), 2.91 (d, *J* = 10.0 Hz, 1H), 2.50–2.39 (m, 1H), 2.27–2.19 (m, 1H), 2.05–1.92 (m, 2H), 1.92–1.82 (m, 1H), 1.80–1.69 (m, 3H), 1.68–1.62 (m, 3H), 1.56–1.51 (m, 4H), 1.50–1.31 (m, 3H), 1.28–1.21 (m, 2H), 1.01 (s, 3H), 0.60 (s, 3H). ^13^C NMR (101 MHz, CDCl_3_) δ 161.3, 149.1, 132.3, 130.6, 125.2, 124.6, 124.5, 124.5, 124.5, 74.6, 73.2, 60.2, 54.1, 45.4, 40.1, 39.6, 37.0, 35.3, 33.7, 33.3, 30.5, 30.4, 25.7, 25.1, 23.9, 21.3, 20.8, 17.0. HRMS-ESI (*m*/*z*): [M + H]^+^ calcd for C_29_H_35_ClFO_3_^+^: 523.2149, found: 523.2160. m.p.: 138–139 °C.

(1*R*,2a*R*,3a*S*,3b*R*,5a*R*,7*S*,9a*S*,9b*S*,11a*R*)-1-(4-cyanophenyl)-9a,11a-dimethylhexadecahydr -onaphtho[1′,2′:6,7]indeno[1,7a-*b*]oxiren-7-yl 2-chloroacrylate (**2-5j**). White solid, yield in 43%. [α]_D_^22.8^ = 3.67 (c 0.120, MeOH). ^1^H NMR (400 MHz, CDCl_3_) δ = 7.51 (d, *J* = 8.0 Hz, 2H), 7.44 (d, *J* = 8.0 Hz, 2H), 6.51 (s, 1H), 6.00 (s, 1H), 5.22 (s, 1H), 3.59 (s, 1H), 2.94–2.85 (m, 1H), 2.49–2.39 (m, 1H), 2.25–2.15 (m, 1H), 2.04–1.91 (m, 2H), 1.91–1.80 (m, 1H), 1.80–1.69 (m, 3H), 1.68–1.59 (m, 3H), 1.59–1.50 (m, 4H), 1.50–1.31 (m, 3H), 1.28–1.21 (m, 2H), 1.00 (s, 3H), 0.58 (s, 3H). ^13^C NMR (101 MHz, CDCl_3_) δ 161.3, 150.6, 132.2, 131.5, 131.1, 125.2, 119.3, 109.5, 74.6, 73.1, 60.1, 54.3, 45.6, 40.1, 39.5, 37.0, 35.3, 33.7, 33.2, 30.5, 30.4, 25.7, 25.0, 23.8, 21.2, 20.8, 17.0. HRMS-ESI (*m*/*z*): [M + H]^+^ calcd for C_29_H_35_ClNO_3_^+^: 488.2300, found: 480.2298. m.p.: 202–203 °C.

(1*R*,2a*R*,3a*S*,3b*R*,5a*R*,7*S*,9a*S*,9b*S*,11a*R*)-9a,11a-dimethyl-1-phenylhexadecahydronaphtho [1′,2′:6,7]indeno[1,7a-*b*]oxiren-7-yl 2-chloroacrylate (**2-5k**). White solid, yield in 43%. [α]_D_^22.3^ = 0.67 (c 0.150, MeOH). ^1^H NMR (400 MHz, CDCl_3_) δ = 7.34–7.27 (m, 2H), 7.25–7.20 (m, 2H), 7.18–7.09 (m, 1H), 6.51 (s, 1H), 6.00 (s, 1H), 5.23 (s, 1H), 3.57 (s, 1H), 2.91–2.81 (m, 1H), 2.47–2.36 (m, 1H), 2.29–2.21 (m, 1H), 2.05–1.93 (m, 2H), 1.92–1.81 (m, 1H), 1.79–1.69 (m, 3H), 1.68–1.59 (m, 3H), 1.55–1.50 (m, 4H), 1.49–1.31 (m, 3H), 1.28–1.19 (m, 1H), 1.00 (s, 3H), 0.61 (s, 3H). ^13^C NMR (151 MHz, CDCl_3_) δ 161.3, 145.0, 132.2, 130.3, 127.6, 125.6, 125.2, 74.7, 73.2, 60.4, 54.3, 45.3, 40.2, 39.6, 37.1, 35.3, 33.8, 33.4, 30.5, 30.4, 25.7, 25.0, 23.9, 21.3, 20.8, 17.0. HRMS-ESI (*m*/*z*): [M + Na]^+^ calcd for C_28_H_35_ClO_3_Na^+^: 477.2167, found: 477.2177. m.p.: 138–139 °C.

(1*R*,2a*R*,3a*S*,3b*R*,5a*R*,7*S*,9a*S*,9b*S*,11a*R*)-1-(4-fluorophenyl)-9a,11a-dimethylhexadecahydr -onaphtho[1′,2′:6,7]indeno[1,7a-*b*]oxiren-7-yl 2-chloroacrylate (**2-5l**). White solid, yield in 28%. [α]_D_^23.2^ = 9.80 (c 0.100, MeOH). ^1^H NMR (600 MHz, CDCl_3_) δ = 7.27 (s, 2H), 6.90 (t, *J* = 8.8 Hz, 2H), 6.51 (s, 1H), 5.99 (s, 1H), 5.23 (s, 1H), 3.56 (s, 1H), 2.88–2.79 (m, 1H), 2.46–2.38 (m, 1H), 2.22–2.14 (m, 1H), 2.04–1.93 (m, 2H), 1.90–1.80 (m, 1H), 1.75–1.68 (m, 3H), 1.67–1.60 (m, 3H), 1.59–1.50 (m, 4H), 1.50–1.45 (m, 1H), 1.45–1.38 (m, 1H), 1.37–1.30 (m, 1H), 1.27–1.20 (m, 1H), 1.00 (s, 3H), 0.59 (s, 3H). ^13^C NMR (151 MHz, CDCl_3_) δ 162.0, 161.3, 160.4, 140.7, 140.6, 132.3, 131.6, 131.6, 125.2, 114.3, 114.1, 74.7, 73.2, 60.2, 53.5, 45.2, 40.1, 39.6, 37.1, 35.3, 33.8, 33.6, 30.5, 30.4, 25.7, 25.1, 23.9, 21.3, 20.7, 17.0. HRMS-ESI (*m*/*z*): [M + H]^+^ calcd for C_28_H_35_ClFO_3_^+^: 490.2519, found: 490.2519. m.p.: 112–113 °C.

*Part 6. Preparation and characterization of* **2-6c**.

*m*-CPBA (6.4 mg, 0.037 mmol) in an ice bath was added to a stirred solution of **2-3c** (10 mg, 0.028 mmol) in CH_2_Cl_2_ (0.14 mL). The reaction mixture was allowed to stir at room temperature under N_2_ atmosphere for 2 h. The solvent was evaporated under vacuum, and the residue was purified by flash column chromatography with EtOAc:petroleum ether (1:2) to give compound **2-6c** (5 mg, 50%), as a white solid.

*Part 7. Preparation of* **2-7**, **2-8c**.

The following refers to the procedure described in Part 6, except that resibufogenin was replaced with **2-6c** or **2-2c**, and the acyl chloride reagent was replaced with α-chloroacrylic acid chloride.

(1*R*,2a*R*,3a*S*,3b*R*,5a*R*,7*S*,9a*S*,9b*S*,11a*R*)-9a,11a-dimethyl-1-(pyrimidin-5-yl)hexadecahyd-ronaphtho[1′,2′:6,7]indeno[1,7a-*b*]oxiren-7-ol (**2-6c**). White solid, yield in 50%. [α]_D_^23.1^ = 8.80 (c 0.100, MeOH). ^1^H NMR (400 MHz, CDCl_3_) δ = 9.07 (s, 1H), 8.49 (s, 2H), 4.13 (s, 1H), 3.60 (s, 1H), 2.85–2.76 (m, 1H), 2.25–2.13 (m, 2H), 2.10–1.99 (m, 2H), 1.98–1.79 (m, 2H), 1.79–1.72 (m, 1H), 1.71–1.55 (m, 5H), 1.54–1.48 (m, 2H), 1.47–1.40 (m, 1H), 1.38–1.24 (m, 3H), 1.24–1.16 (m, 1H), 1.15–1.08 (m, 1H), 0.98 (s, 3H), 0.59 (s, 3H). ^13^C NMR (101 MHz, CDCl_3_) δ 157.1, 156.9, 132.9, 72.8, 66.9, 57.8, 44.6, 42.8, 36.4, 36.3, 35.3, 34.0, 33.2, 32.5, 29.8, 29.2, 27.9, 25.6, 23.5, 20.7, 19.8, 16.1. HRMS-ESI (*m*/*z*): [M + H]^+^ calcd for C_23_H_33_N_2_O_2_^+^: 369.2537, found: 369.2549. m.p.: 169–170 °C.

(1*R*,2a*R*,3a*S*,3b*R*,5a*R*,7*S*,9a*S*,9b*S*,11a*R*)-9a,11a-dimethyl-1-(pyrimidin-5-yl)hexadecahyd-ronaphtho[1′,2′:6,7]indeno[1,7a-*b*]oxiren-7-yl 2-chloroacrylate (**2-7c**). White solid, yield in 49%. ^1^H NMR (600 MHz, CDCl_3_) δ = 9.08 (s, 1H), 8.49 (s, 2H), 6.50 (d, *J* = 1.3 Hz, 1H), 5.98 (d, *J* = 1.3 Hz, 1H), 5.20 (q, *J* = 2.9 Hz, 1H), 3.61 (s, 1H), 2.82 (dd, *J* = 11.3, 6.7 Hz, 1H), 2.25–2.16 (m, 2H), 2.09–2.00 (m, 2H), 1.95–1.88 (m, 1H), 1.83 (td, *J* = 12.0, 3.6 Hz, 1H), 1.74–1.68 (m, 3H), 1.68–1.58 (m, 4H), 1.54–1.49 (m, 1H), 1.45–1.36 (m, 2H), 1.34–1.28 (m, 1H), 1.26–1.20 (m, 1H), 1.16–1.11 (m, 1H), 1.00 (s, 3H), 0.60 (s, 3H). ^13^C NMR (151 MHz, CDCl_3_) δ 161.2, 157.2, 156.9, 132.8, 132.3, 125.1, 73.3, 72.7, 57.9, 44.6, 42.7, 37.3, 36.6, 35.1, 33.9, 32.5, 30.7, 30.2, 29.2, 25.4, 25.0, 23.6, 20.7, 19.7, 16.1. HRMS-ESI (*m*/*z*): [M + H]^+^ calcd for C_26_H_34_ClN_2_O_3_^+^:457.2252, found: 457.2251. m.p.: 145–146 °C.

(1*R*,2a*R*,3a*S*,3b*R*,5a*R*,7*S*,9a*S*,9b*S*,11a*R*)-14-hydroxy-10,13-dimethyl-17-(pyrimidin-5-yl)he -xadecahydro-1H-cyclopenta[*a*]phenanthren-3-yl 2-chloroacrylate (**2-8c**). White solid, yield in 49%. [α]_D_^23.2^ = 7.78 (c 0.090, MeOH). ^1^H NMR (400 MHz, CDCl_3_) δ = 9.07 (s, 1H), 8.60 (s, 2H), 6.51 (s, 1H), 5.99 (s, 1H), 5.22 (s, 1H), 3.48 (t, *J* = 9.4 Hz, 1H), 2.20–2.10 (m, 2H), 2.10–2.01 (m, 1H), 1.99–1.92 (m, 2H), 1.89–1.77 (m, 3H), 1.74–1.70 (m, 1H), 1.68–1.63 (m, 1H), 1.62–1.49 (m, 3H), 1.48–1.37 (m, 4H), 1.36–1.26 (m, 3H), 1.21–1.15 (m, 1H), 1.00 (s, 3H), 0.61 (s, 3H). ^13^C NMR (101 MHz, CDCl_3_) δ 161.3, 157.0, 156.8, 134.4, 132.3, 125.1, 85.0, 73.4, 48.7, 47.1, 39.1, 37.5, 35.2, 33.8, 32.6, 30.9, 30.4, 29.7, 26.2, 24.9, 24.0, 23.6, 21.2, 19.7, 16.7. HRMS-ESI (*m*/*z*): [M + H]^+^ calcd for C_26_H_36_ClN_2_O_3_^+^:460.2487, found: 460.2447. m.p.: 142–143 °C.

### 4.2. Biology

#### 4.2.1. Cell Culture and Cell-Based Lucia Luciferase Assay

Microglia BV2 cells were purchased from the Shanghai Institute of Biochemistry and Cell Biology, Chinese Academy of Sciences. All cells were cultivated in DMEM. Fetal bovine serum (FBS, 10%) and antibiotics (1% penicillin-streptomycin) were added to the medium, and the cells were kept in a humid environment of 37 °C and 5% CO_2_.

The experiment was conducted with ARE-Luc-BV2 cells. The cells were cultured following our previous research protocol [49]. The cells were cultivated in a 96-well plate at a density of 2 × 10^4^ cells per well and subjected to compounds or tert-butylhydroquinone (t-BHQ, a positive control) for 12 h. Subsequently, the previous culture media was dumped, and D-fluorescein potassium salt was added. Then, the luciferase activity was measured using a microplate reader (Thermo Fisher Scientific, Waltham, MA, USA). The data were expressed as a percentage relative to the control.

#### 4.2.2. Animals

Male C57/B6J mice (12 weeks, 25–27 g) were maintained in a 22 to 24 °C comfortable room on a 12 h light/dark cycle and were allowed free access to food and water. Following a week of behavior evaluations, subsequent analyses were conducted by sectioning the brain tissue. All experimental protocols were approved by the ethical committee of the Shanghai University of Traditional Chinese Medicine (PZSHUTCM2506260006).

#### 4.2.3. MPTP Subacute PD Models and Treatment

MPTP-induced PD models were developed as described previously [50]. Briefly, mice were separated equally into groups (n = 6/group) and given PBS, MPTP (30 mg/kg, biyuntian, intraperitoneal), **2-5c** (4 mg/kg, oral gavage), and L-Dopa (20 mg/kg, taoshu, oral gavage). Both **2-5c** and L-Dopa were formulated as homogeneous suspensions using a vehicle consisting of 5% DMSO and 95% aqueous sodium carboxymethyl cellulose (5% CMC-Na). These were given 1 h before MPTP injection, and drug administration lasted 7 days. Following a week of behavior evaluations, the mice were transcardially perfused with cold phosphate-buffered saline (PBS), and the brain tissues were extracted and processed for immunohistochemistry.

#### 4.2.4. Behavioral Analyses

Behavioral analyses were performed as previously described [51,52]. For the rotarod test, mice underwent three consecutive days of acclimatization training prior to the formal experiment. In formal experiments, the rotarod speed was uniformly accelerated from 4 rpm/min to 40 rpm/min in 5 min, and the latency to fall was recorded using the Rotarod Instrument (Panlab, Holliston, MA, USA). For the pole test, a vertical wooden pole with a rough surface (diameter 1 cm, height 55 cm) was used. Mice were similarly acclimatized for three consecutive days before testing. For the formal test, each mouse was placed at the top of the pole in a head-up position, and the time required for the animal to descend to the floor (defined as both hind limbs touching the ground) was recorded. For the open field test, each mouse was gently placed into the center of the open field arena by the researcher, with efforts made to standardize the starting position across trials. The animals were then allowed to explore freely while their movement trajectories were automatically recorded and analyzed using VisuTrack software (version 2.0, Shanghai Xinruan Information Technology, Shanghai, China) to determine total distance traveled and average locomotor speed. Grip strength of the forelimbs was assessed using an automatic grip strength meter (Sansbio, Nanjing, China). During testing, each mouse was held by the tail and allowed to grasp a horizontal bar with its forepaws. The animal was then gently pulled backward until it released the bar. This procedure was repeated at least five times per mouse, and the maximum grip strength values were recorded and averaged to obtain the final result for each animal.

#### 4.2.5. Immunohistochemical (IHC)

To commence the immunohistochemical staining, brain tissue sections were rinsed in phosphate-buffered saline (PBS) and subsequently incubated in 3% hydrogen peroxide (H_2_O_2_) for 10 min to quench endogenous peroxidase activity. Following a further PBS wash, non-specific binding sites were blocked by immersion in 10% goat serum, and the tissues were permeabilized with 0.1% Triton X-100 in PBS. The sections were then subjected to incubation with a primary anti-TH antibody at 4 °C overnight. For signal detection, a biotin-conjugated goat anti-mouse secondary antibody was employed, followed by development with diaminobenzidine. Imaging was ultimately conducted using an optical microscope (Nikon, Tokyo, Japan).

#### 4.2.6. Statistical Analysis

Results were presented as the means ± SEM. Statistical analysis was carried out by GraphPad Prism 9 software. As for three or more groups, the differences between them were compared by one-way ANOVA multiple comparisons. A difference of *p* < 0.05 was considered statistically significant.

## 5. Conclusions

In summary, through systematic structural modification of RBG, we successfully obtained 45 derivatives and preliminarily elucidated their structure–activity relationships. Among them, **2-5c**, incorporating a 2-chloroacryloyloxy group at the C3 position and a pyrimidine ring at the C17 position, exhibited relatively potent activity, with an Nrf2 agonistic activity (EC_50_ = 4.18 μM) approximately 7-fold higher than that of RBG based on the ARE-Luc-BV2 cell luciferase assay, along with a favorable safety profile. In a mouse model of PD, **2-5c** alleviated motor deficits and demonstrated protective effects on dopaminergic neurons. This study provides a useful reference for understanding the SAR of RBG and lays a foundation for further exploration of Nrf2-based therapeutic strategies for PD, suggesting that **2-5c** represents a promising candidate for further investigation and optimization in this direction.

## Figures and Tables

**Table 1:** In vitro Nrf2 agonistic activities of C3 analogues ^a^.

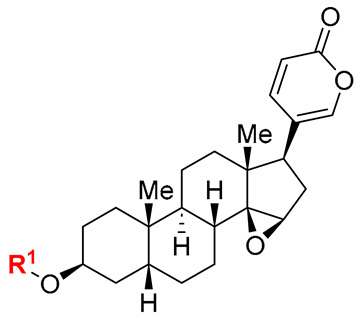					
Compd.	R^1^	Relative Fluorescence Intensity@5 μM (Mean ± SD)	Compd.	R^1^	Relative Fluorescence Intensity@5 μM (Mean ± SD)
RBG	--	1.05 ± 0.05	**1-7g**	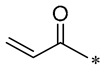	1.82 ± 0.09
tBHQ	--	1.94 ± 0.09	**1-7h**	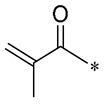	1.12 ± 0.03
**1-7a**	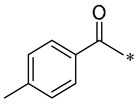	0.91 ± 0.02	**1-7i**	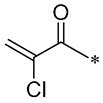	4.87 ± 0.16
**1-7b**	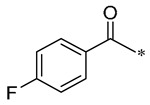	1.04 ± 0.03	**1-7j**	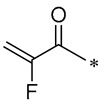	1.55 ± 0.02
**1-7c**	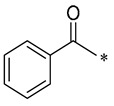	1.17 ± 0.03	**1-7k**	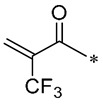	1.11 ± 0.02
**1-7 d**	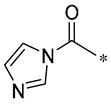	0.87 ± 0.06	**1-7l**	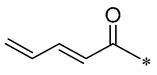	1.06 ± 0.04
**1-7e**	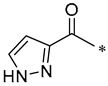	0.83 ± 0.05	**1-7m**	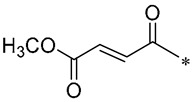	1.10 ± 0.01
**1-7f**	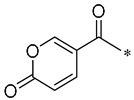	1.08 ± 0.05	**1-7n**	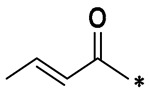	0.81 ± 0.05
			**1-7o**	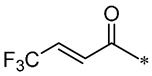	0.50 ± 0.05

^a^ Data are presented as means ± SD of *n* = 3. *: Represents the connection point with the main ring framework.

**Table 2:** In vitro Nrf2 agonistic activities of C17 analogues ^a^.

	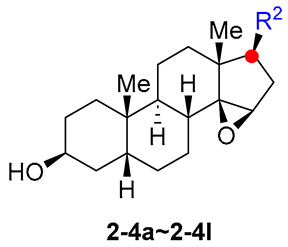		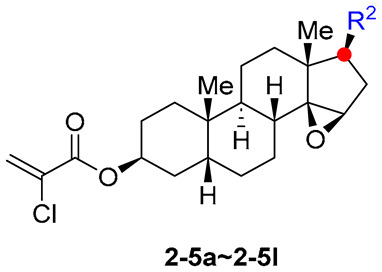	
R^2^	Compd.	Relative Fluorescence Intensity@5 μM (Mean ± SD)	Compd.	Relative Fluorescence Intensity@5 μM (Mean ± SD)
--	RBG	1.05 ± 0.05	**1-7i**	4.87 ± 0.16
--	TBHQ	1.94 ± 0.09	--	--
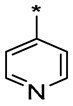	**2-4a**	0.99 ± 0.01	**2-5a**	5.48 ± 0.97
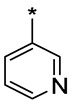	**2-4b**	1.07 ± 0.05	**2-5b**	5.08 ± 0.12
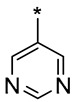	**2-4c**	0.94 ± 0.06	**2-5c**	8.76 ± 0.40
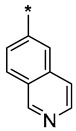	**2-4d**	0.86 ± 0.02	**2-5d**	1.09 ± 0.03
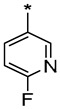	**2-4e**	0.88 ± 0.04	**2-5e**	1.05 ± 0.03
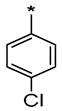	**2-4f**	0.73 ± 0.05	**2-5f**	0.87 ± 0.07
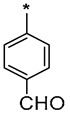	**2-4g**	0.96 ± 0.07	**2-5g**	1.52 ± 0.07
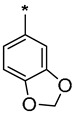	**2-4h**	0.75 ± 0.05	**2-5h**	0.95 ± 0.06
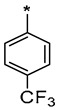	**2-4i**	0.65 ± 0.09	**2-5i**	0.80 ± 0.03
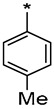	**2-4j**	1.11 ± 0.06	**2-5j**	0.79 ± 0.05
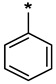	**2-4k**	0.69 ± 0.03	**2-5k**	1.00 ± 0.03
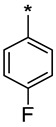	**2-4l**	0.74 ± 0.04	**2-5l**	0.92 ± 0.04

^a^ Data are presented as means ± SD of *n* = 3. *: Represents the connection point with the main ring framework.

**Table 3:** In vitro Nrf2 agonistic activities of D-ring analogues ^a^.

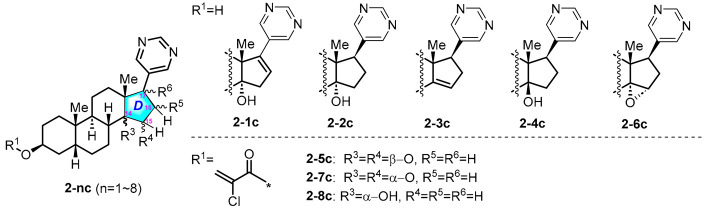			
Compd.	Relative Fluorescence Intensity@5 μM (Mean ± SD)	Compd.	Relative Fluorescence Intensity@5 μM (Mean ± SD)
RBG	1.05 ± 0.05	**2-4c**	0.94 ± 0.06
TBHQ	1.94 ± 0.09	**2-5c**	8.76 ± 0.40
**2-1c**	0.85 ± 0.06	**2-6c**	1.14 ± 0.05
**2-2c**	0.78 ± 0.04	**2-7c**	1.54 ± 0.07
**2-3c**	0.86 ± 0.05	**2-8c**	2.17 ± 0.18

^a^ Data are presented as means ± SD of *n* = 3. *: Represents the connection point with the main ring framework.

**Table 4:** The cell viability and EC_50_ of the highly active compounds ^a^.

Compd.	Cell Viability at 5 μM (%)	Nrf2 EC_50_ (μM)
RBG	--	28.2 ± 1.0
**1-7i**	25.0 ± 2.9	--
**2-5a**	112 ± 11	4.69 ± 0.02
**2-5b**	91 ± 3	4.01 ± 0.20
**2-5c**	97.0 ± 2.9	4.18 ± 0.08
**2-6c**	92.0 ± 3.7	11.75 ± 0.13
**2-7c**	104.7 ± 1.7	8.62 ± 0.03
**2-8c**	102 ± 10	11.82 ± 0.12

^a^ Data are presented as means ± SD of *n* = 3.

**Table 5:** Protective effects of **2-5c** against MPP^+^-induced damage in BV2 cells ^a^.

Control	Cell Viability with 800 μM MPP^+^ (%)	Cell Viability with MPP^+^ 800 μM + 2-5c 0.5 μM (%)	Cell Viability with MPP^+^ 800 μM + 2-5c 1 μM (%)	Cell Viability with MPP^+^ 800 μM + 2-5c 2 μM (%)
100 ± 4	58.0 ± 0.9	69. 5 ± 6.3	73.9 ± 2.8	74.2 ± 2.5

^a^ Data are presented as means ± SD of *n* = 3.

## Data Availability

The original contributions presented in this study are included in the article/Supplementary Materials. Further inquiries can be directed to the corresponding authors.

## Supplementary Materials

The following supporting information can be downloaded at: https://www.mdpi.com/article/10.3390/ijms27073326/s1.
