# Supplementary material for: Synthesis and Biological Evaluation of RBG Derivatives as Nrf2 Activators for the Treatment of Parkinson’s Disease
Source: Int J Mol Sci. 2026 Apr 7;27(7):3326. doi: 10.3390/ijms27073326 (PMC13073808; doi:10.3390/ijms27073326)

Supporting Information

Synthesis and Biological Evaluation of RBG derivativesas as Nrf2 activators for the treatment of Parkinson's disease

Wen-Qing Shi [1,†], Jia-Hua Zhuang [1,†], Qiu-Heng Zhang [†], Guo-Qiang Lin [1,2], Shaopeng Yu [1], Yao Chen [1], Jun-Huan Fu [1], Jiange Zhang [1], Shoujiao Peng [1,*], Gu-Zhou Chen [1], Wenbo Ye [1,*]

1. General Information

Nuclear magnetic resonance (NMR) spectra were recorded on a BRUKER AVANCE NEO 600 MHz spectrometer (Bruker Co., Switzerland) or a BRUKER AVANCE NEO 400 MHz spectrometer (Bruker Co., Switzerland). The spectra were calibrated by using residual undeuterated solvents (for ^{1}H NMR) and deuterated solvents (for ^{13}C NMR) as internal references: undeuterated chloroform (δH = 7.26 ppm) and $CDCl_3$ (δC = 77.16 ppm); undeuterated methanol (δH = 3.31 ppm) and methanol-d4 (δC = 49.00 ppm); NMR data were reported as chemical shift (*δ*, ppm), coupling constants (*J*, Hz), multiplicity and integration. High resolution mass spectra (HRMS) were obtained from 6545 Accurate-Mass Q-TOF (Agilent Technologies, Inc., America).

2. Experimental Procedures and Compounds Characterization

Preparation and Characterization of C17 and C14, 15 derivatives

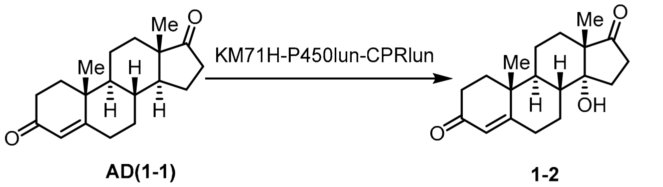

Compound 14*α*-OH-AD **1-2**: Under optimal conditions (pH = 7.5, 2 g/L AD, 72 h), KM71H-P450lun-CPRlun was cultured and induced in 1 L of MMH medium at a 2 L shake flask. The fermentation broth was extracted twice with an equal volume of ethyl acetate. The organic solvant was removed in vacuo, and the residue was purified by flash column chromatography with EtOAc: petroleum ether (2:1) to give compound 14*α*-OH-AD **1-2** (0.98 g, 50%) as a white solid.

^{1}H NMR (400 MHz, CDC l3) δ = 5.78 (s, 1H), 2.55-2.35 (m, 6H), 2.08 (dq, *J* = 12.8, 4.3 Hz, 1H), 2.03-1.93 (m, 3H), 1.82 (qd, *J* = 13.6, 5.3 Hz, 3H), 1.72-1.63 (m, 2H), 1.60 (d, J = 8.3 Hz, 1H), 1.51-1.37 (m, 3H), 1.25 (s, 3H), 1.08 (s, 3H).

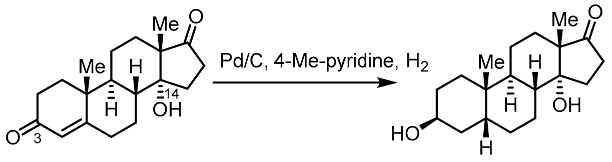

1-2 **3β-OH, 14α-OH-AD**

Compound **3β-OH, 14*α*-OH-AD**: To a stirred solution of **1-2** (5.0 g, 5.0 mmol) in 4-methylpyridine (55 mL) was added Pd/C (1.81 g, 20% w/w, wetted with 55% water). The reaction mixture was allowed to stir at room temperature under H_2 atmosphere for 20 h before it was filtered through a pad of Celite and washed with EtOAc (400 mL). The filtrate was washed with saturated aq. citric acid (3 × 300 mL), brine (300 mL), dried over Na_2SO_4, and filtered. The solvent was evaporated under vacuum, and the residue was purified by flash column chromatography with EtOAc: petroleum ether (1:2) to give compound **3β-OH, 14*α*-OH-AD** (4.1 g, 82%) as a white solid.

^{1}H NMR (600 MHz, $CDCl_3$) δ = 2.72 (t, *J* = 14.3 Hz, 1H), 2.50-2.37 (m, 3H), 2.22 (dq, *J* = 14.7, 3.0 Hz, 1H), 2.14 (td, *J* = 12.0, 4.6 Hz, 1H), 2.11-2.05 (m, 2H), 2.03-1.94 (m, 2H), 1.94-1.83 (m, 4H), 1.64-1.57 (m, 3H), 1.54-1.45 (m, 3H), 1.45-1.39 (m, 2H), 1.09 (s, 3H), 1.05 (s, 3H).

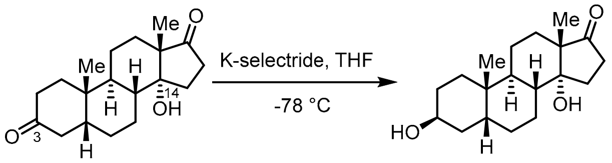

3β-OH, 14α-OH-AD **1-3**

Compound **1-3**: To a stirred solution of compound **3β-OH, 14*α*-OH-AD** (3.5 g, 11.5 mmol) in THF (35 mL) was added K-selectride (15 mL, 1.0 M in THF, 15 mmol) at -78 °C. The mixture was allowed to stir at that temperature for 2 h before it was quenched with saturated aq. NH_4Cl (50 mL). The resultant mixture was extracted with EtOAc (3 × 100 mL), and the combined organic phases were washed with saturated aq. $NaHCO_3$ (200 mL), brine (200 mL), dried over anhydrous Na_2SO_4, and filtered. The solvent was evaporated under vacuum, and the residue was purified by flash column chromatography with EtOAc: petroleum ether (1:2) to give compound **1-3** (2.5 g, 71%) as a white solid.

^{1}H NMR (400 MHz, $CDCl_3$) δ = 4.15 (s, 1H), 2.49-2.31 (m, 2H), 2.07-1.87 (m, 5H), 1.87-1.73 (m, 3H), 1.70-1.58 (m, 2H), 1.56-1.51 (m, 3H), 1.49-1.42 (m, 3H), 1.41-1.26 (m, 5H), 1.03 (s, 3H), 1.02 (s, 3H).

^{1}H NMR Spectrum of 1-7a (400 MHz, CDCl$_3$)

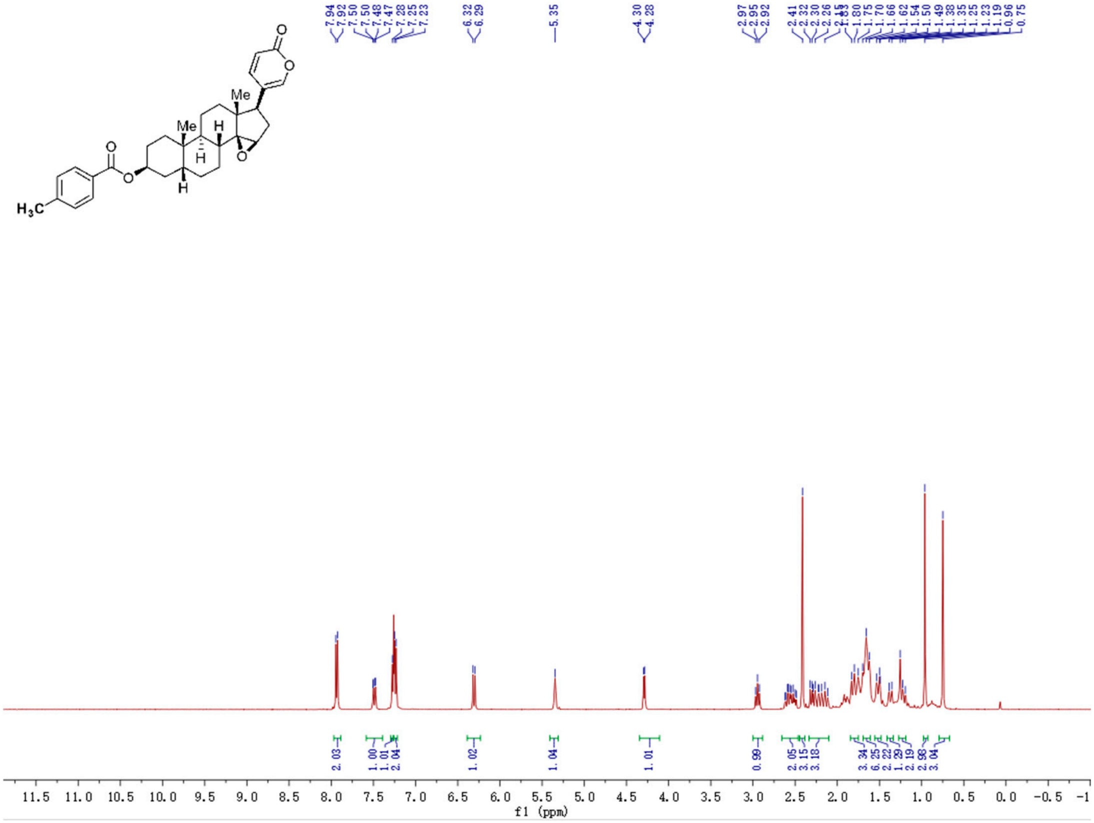

^{13}C NMR Spectrum of 1-7a (101 MHz, CDCl$_3$)

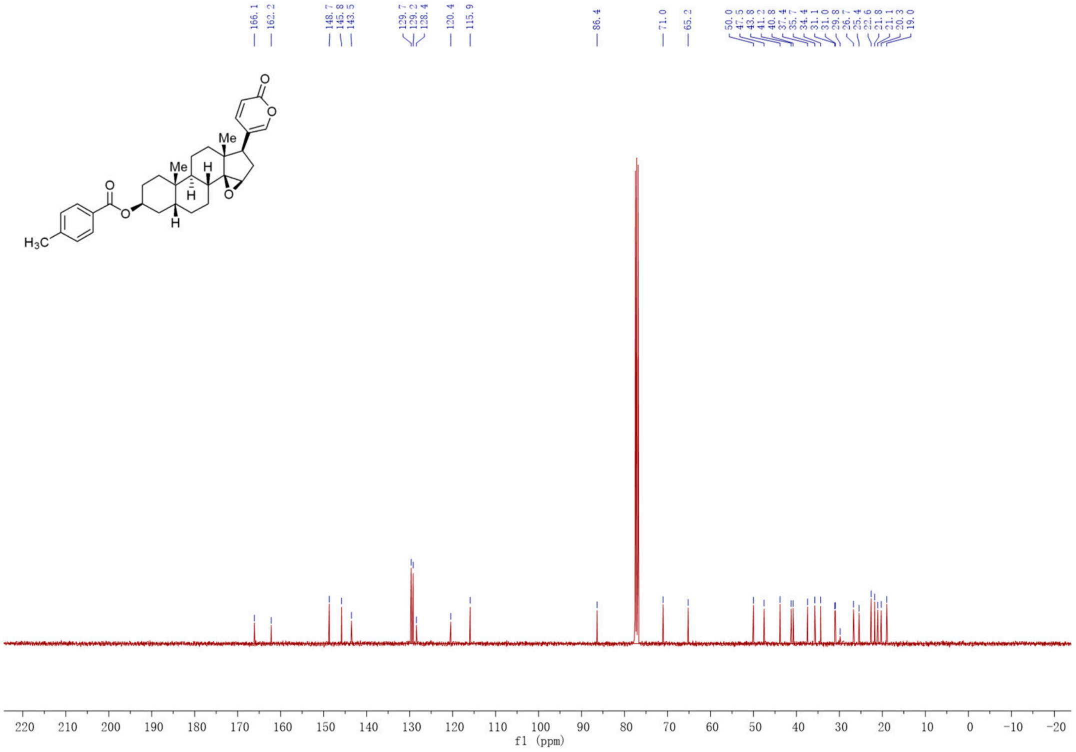

^{1}H NMR Spectrum of 1-7b (400 MHz, $CDCl_3$)

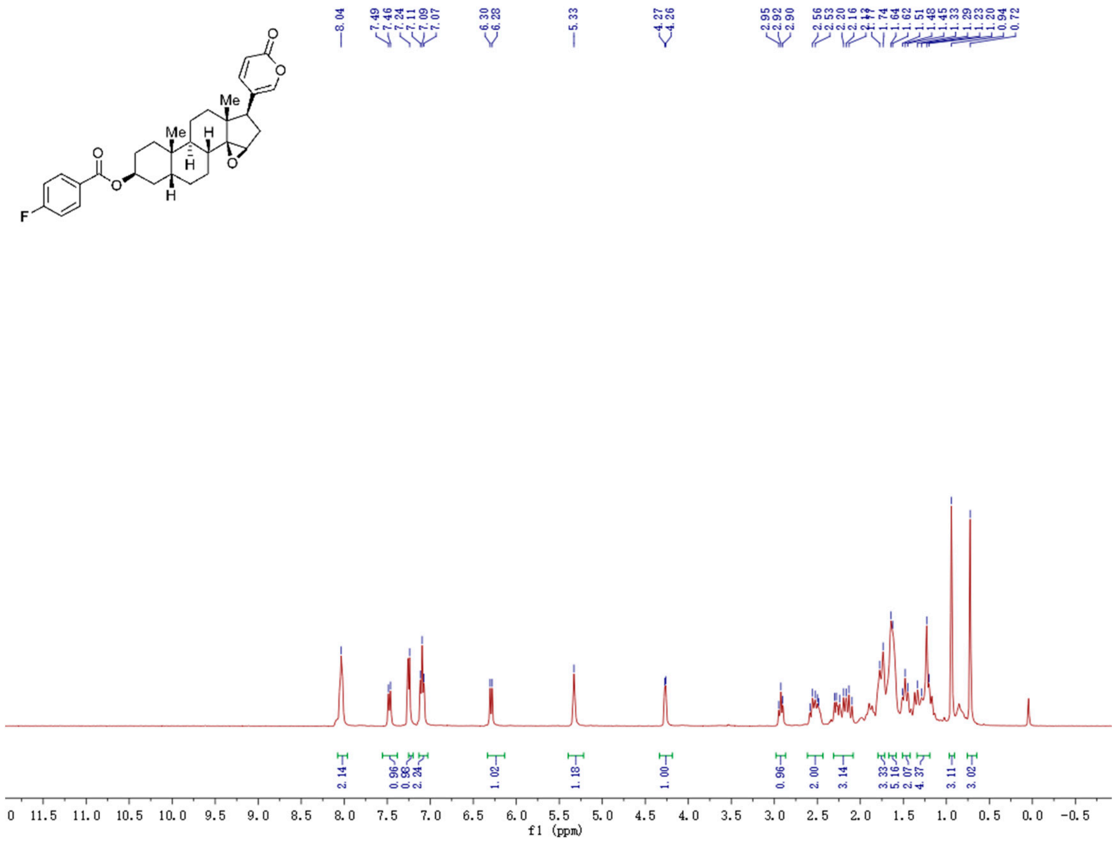

^{13}C NMR Spectrum of 1-7b (101 MHz, $CDCl_3$)

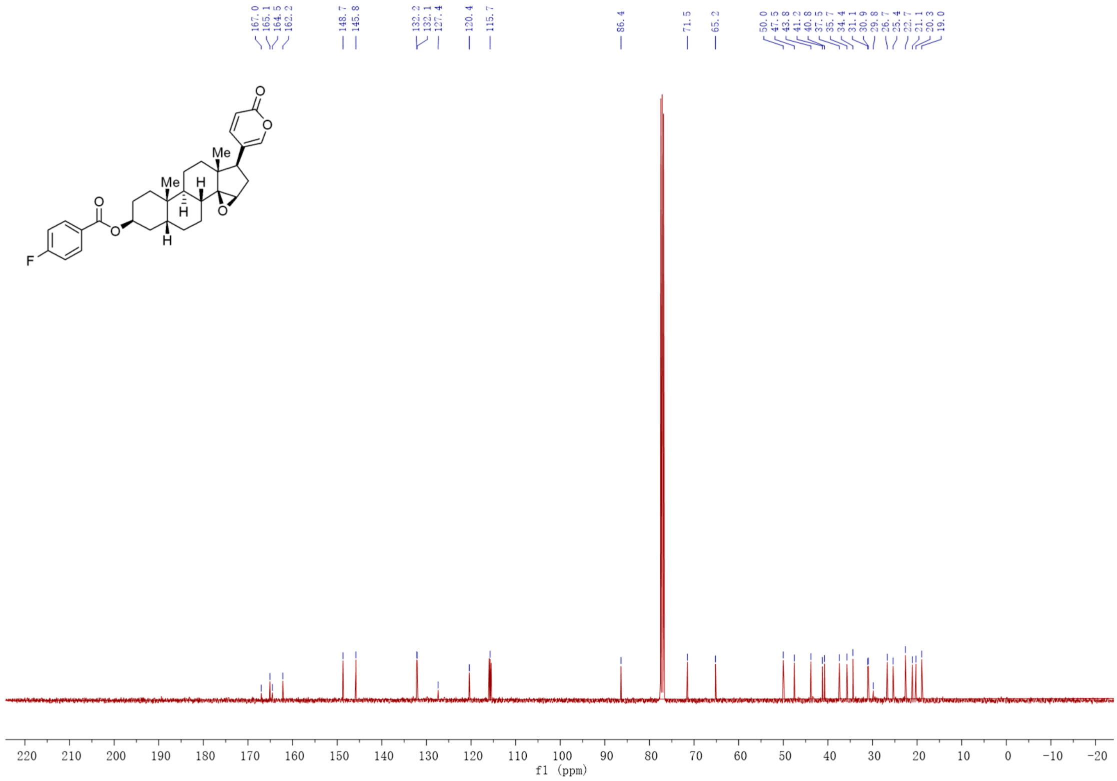

^{1}H NMR Spectrum of 1-7c (400 MHz, $CDCl_3$)

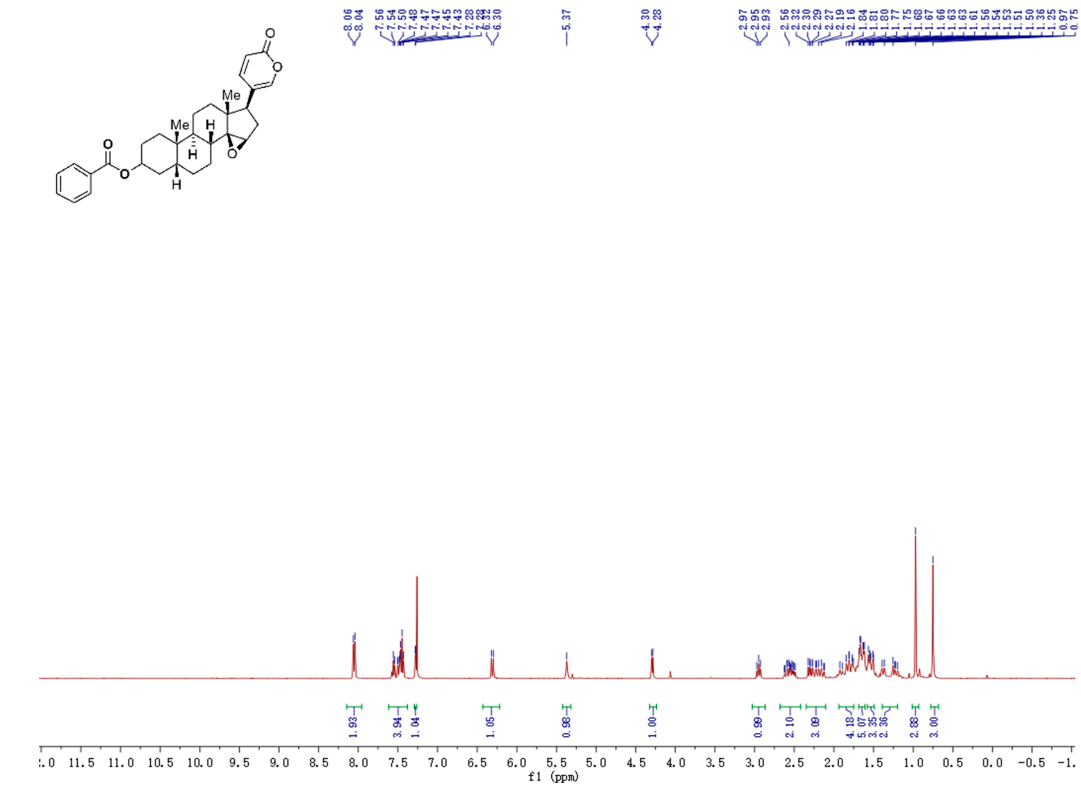

^{13}C NMR Spectrum of 1-7c (101 MHz, $CDCl_3$)

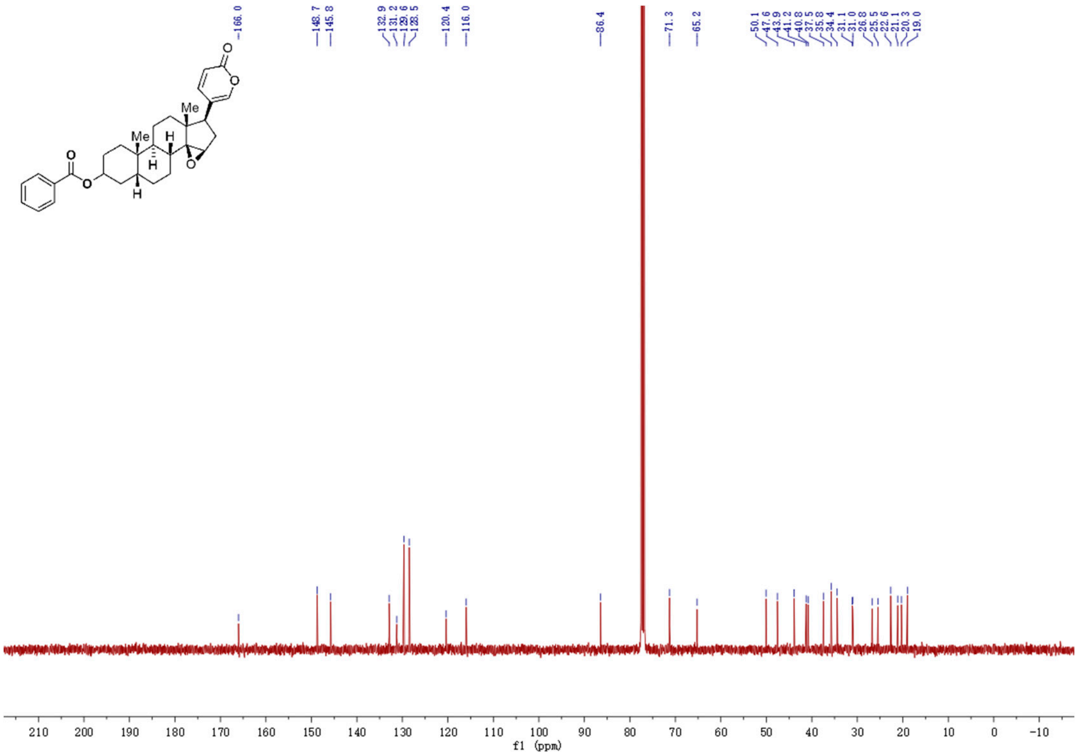

^{1}H NMR Spectrum of 1-7d (400 MHz, $CDCl_3$)

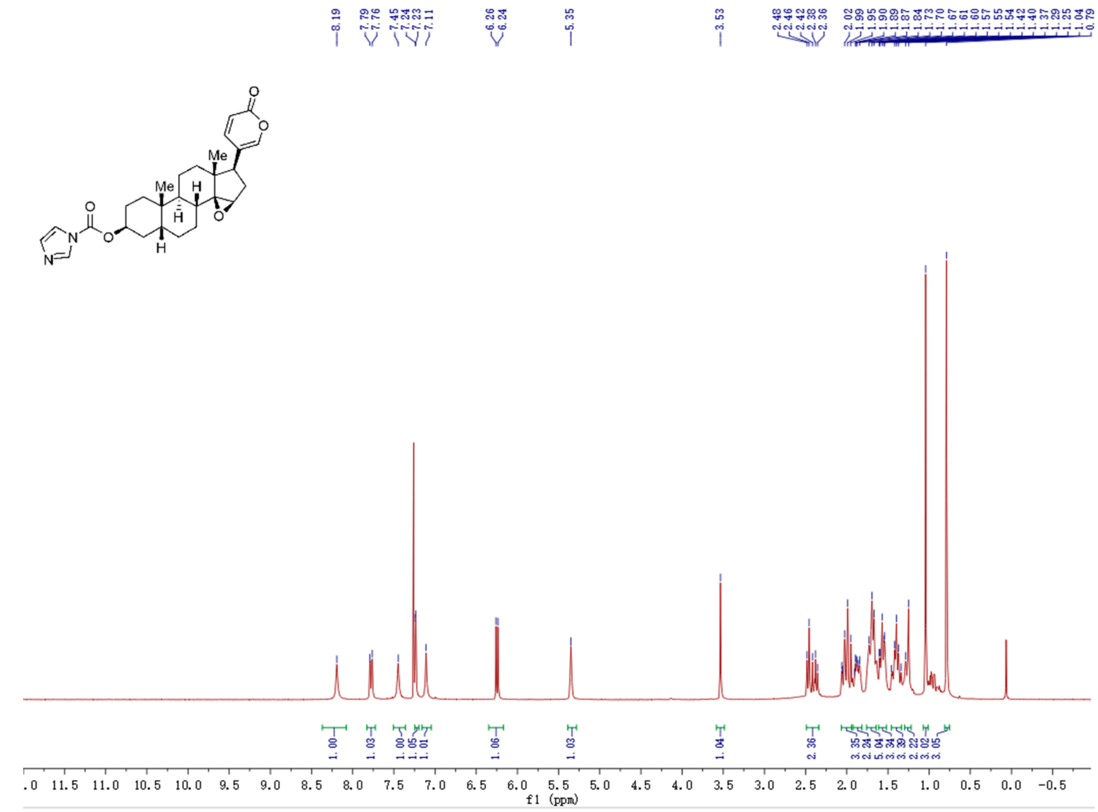

^{13}C NMR Spectrum of 1-7d (101 MHz, $CDCl_3$)

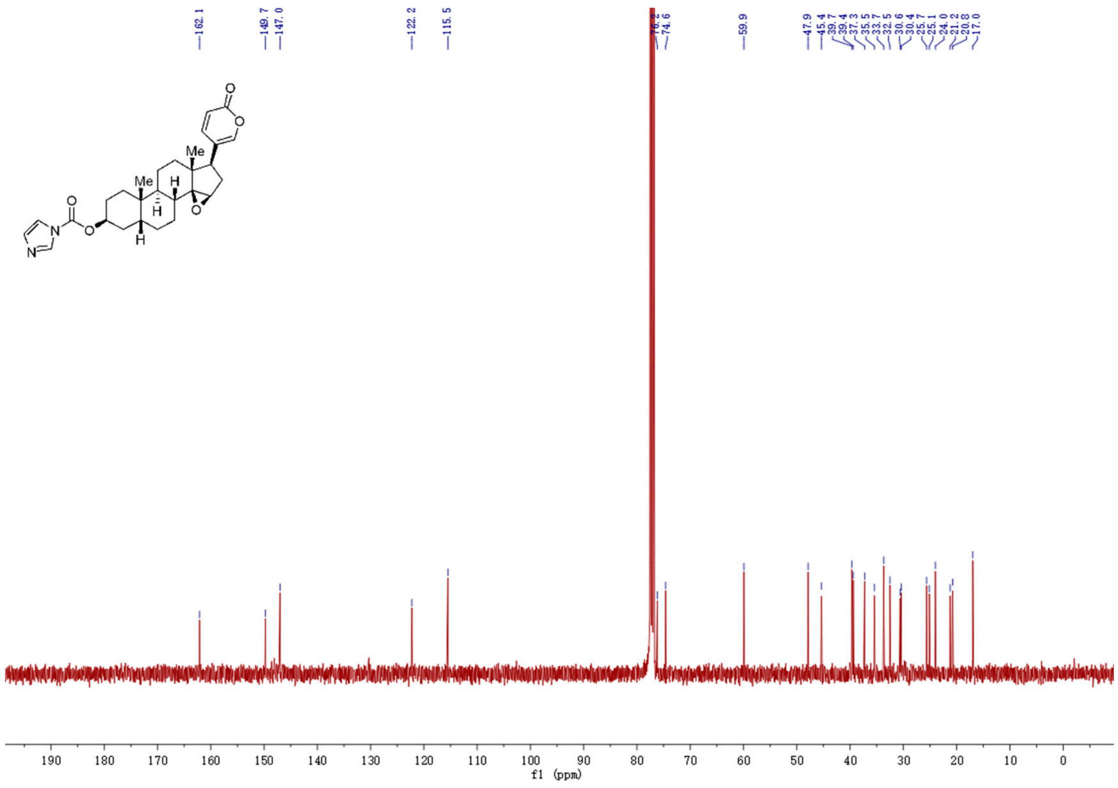

^{1}H NMR Spectrum of 1-7e(400 MHz, $CDCl_3$)

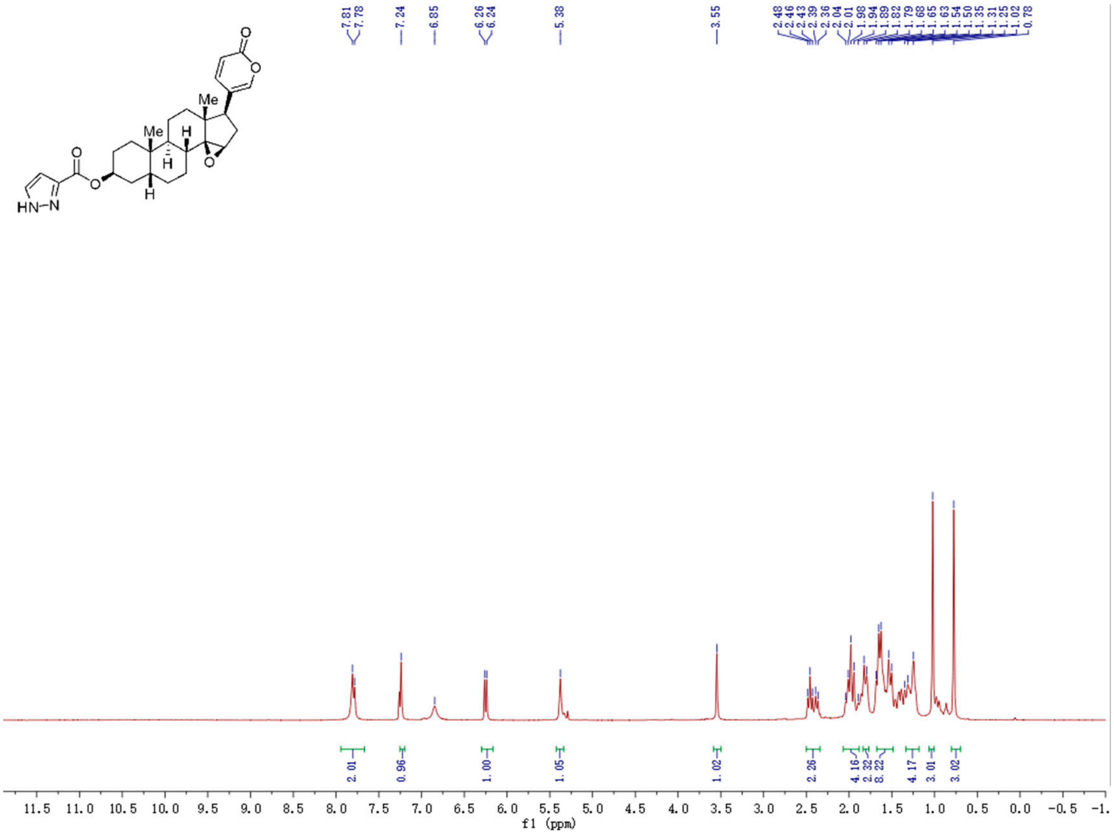

^{13}C NMR Spectrum of 1-7e (101 MHz, $CDCl_3$)

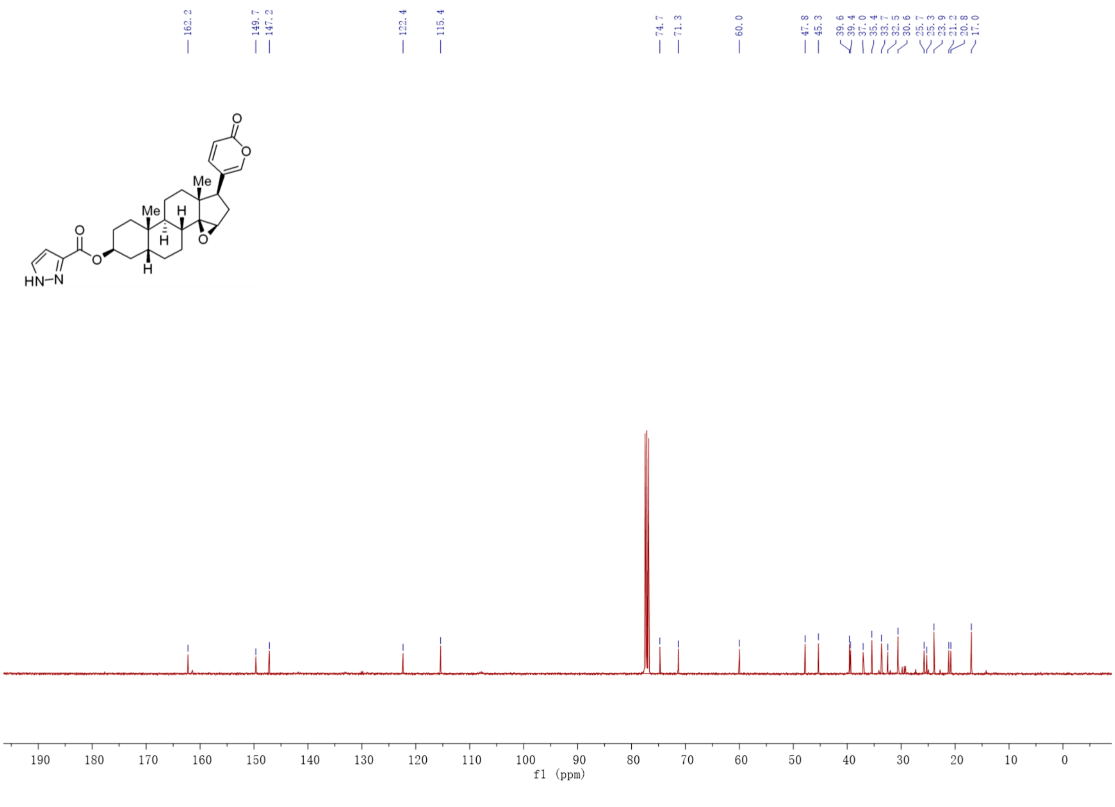

^{1}H NMR Spectrum of 1-7f (400 MHz, $CDCl_3$)

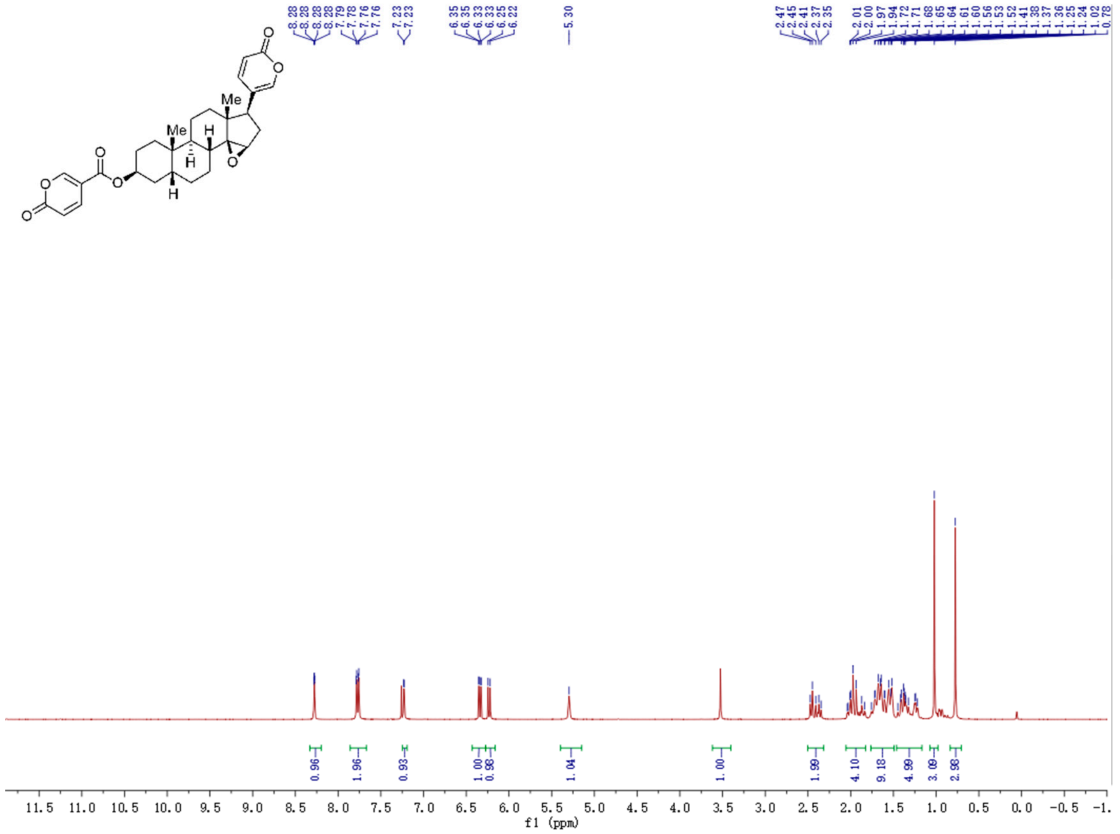

^{13}C NMR Spectrum of 1-7f (101 MHz, $CDCl_3$)

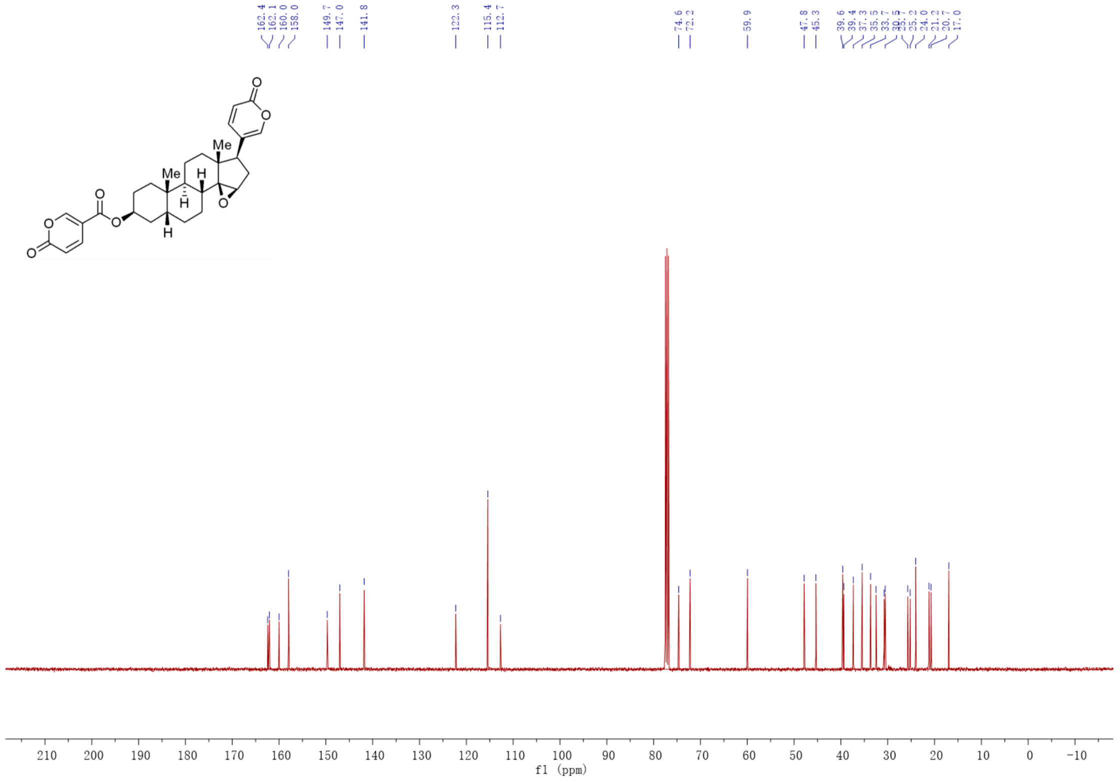

^{1}H NMR Spectrum of 1-7g (400 MHz, $CDCl_3$)

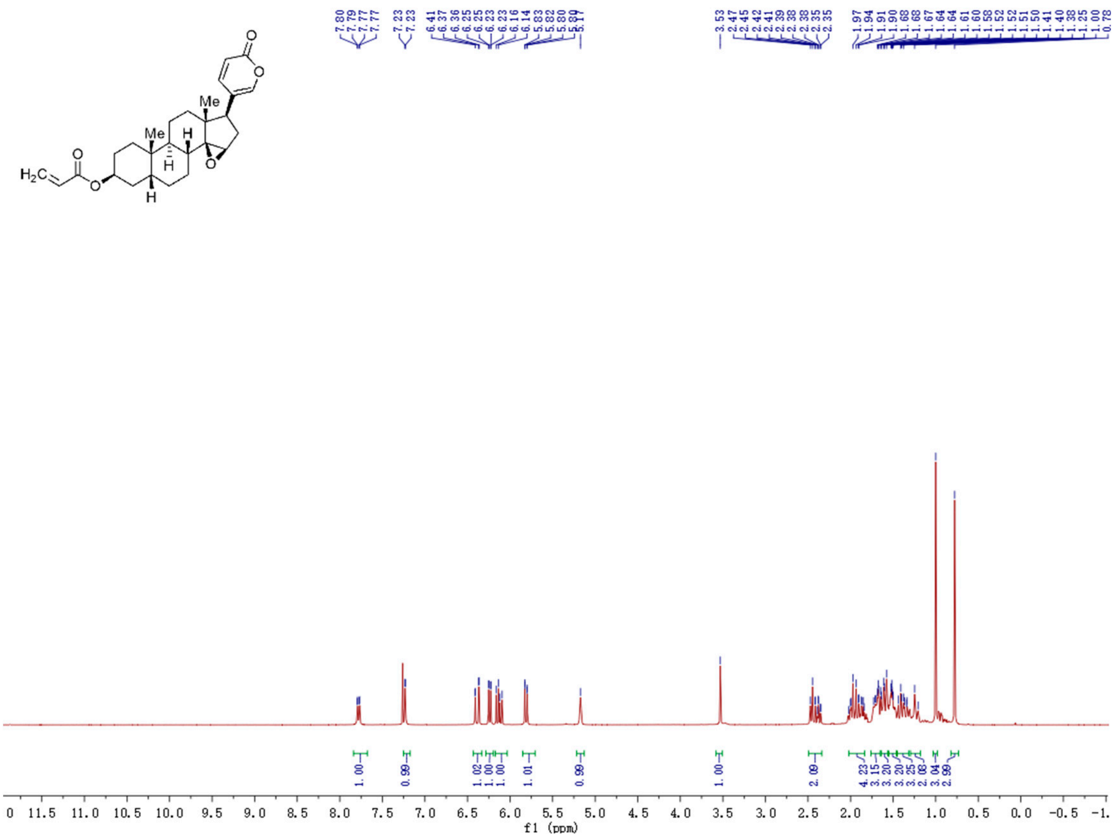

^{13}C NMR Spectrum of 1-7g (101 MHz, $CDCl_3$)

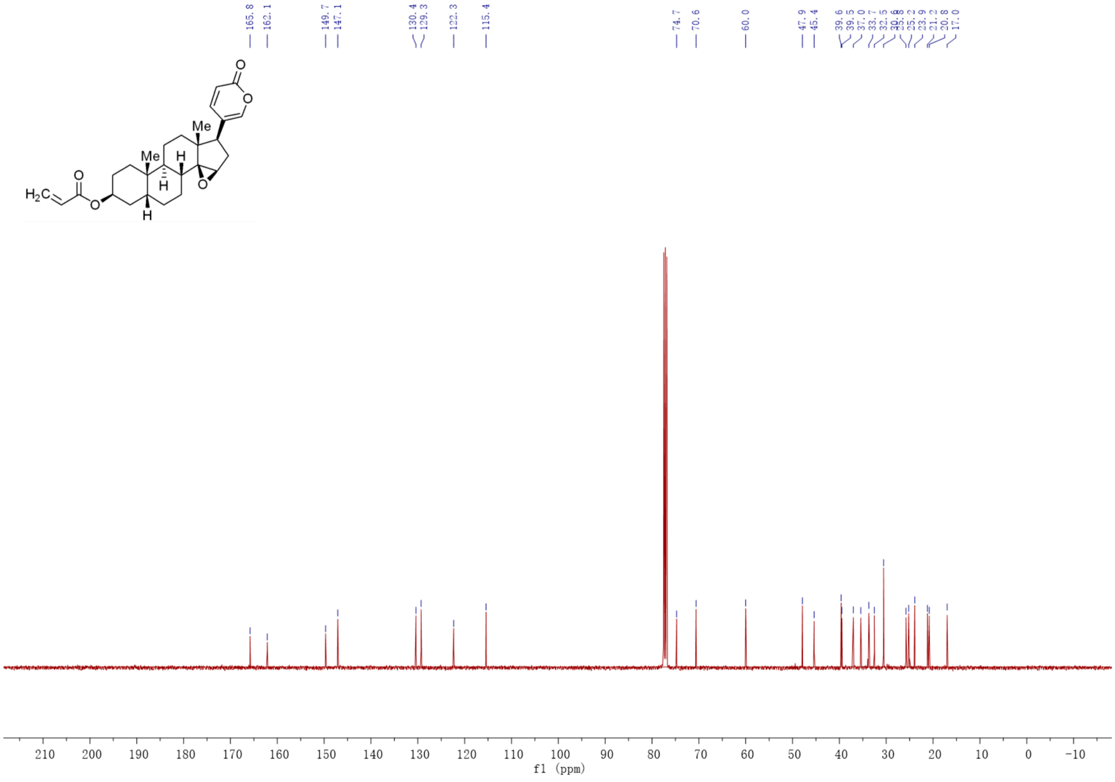

^{1}H NMR Spectrum of 1-7h (400 MHz, $CDCl_3$)

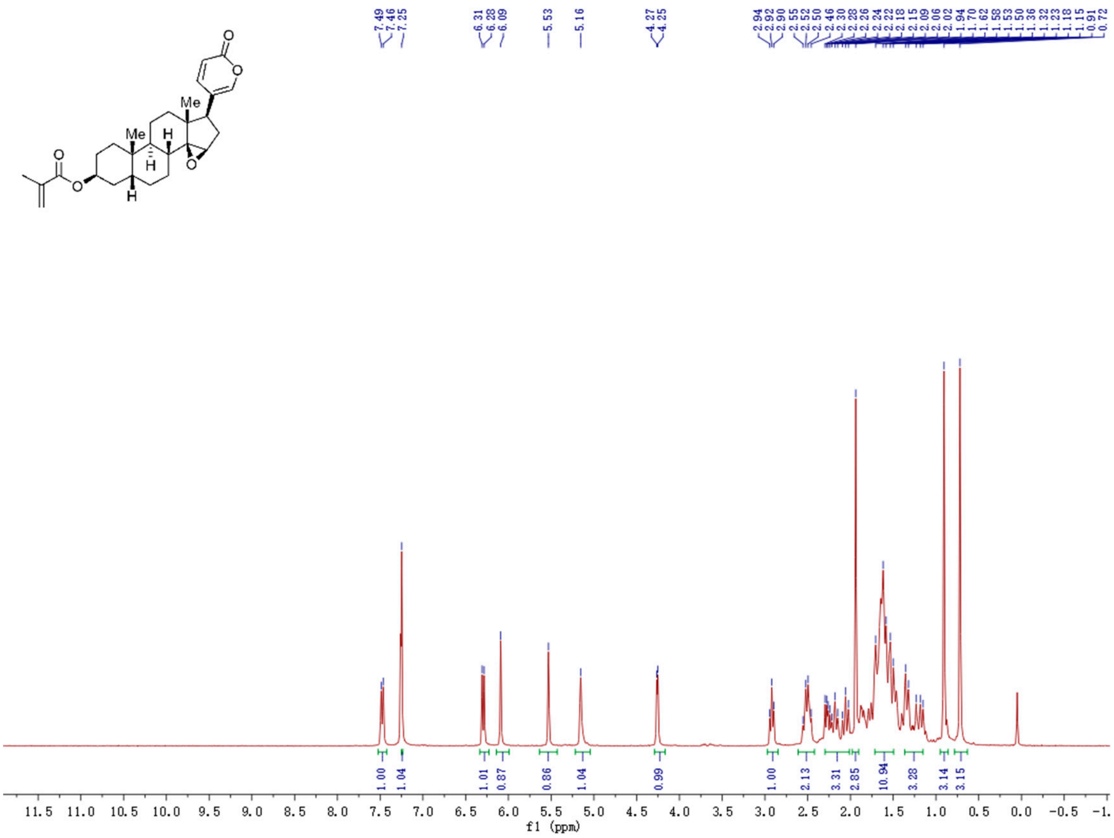

^{13}C NMR Spectrum of 1-7h (101 MHz, $CDCl_3$)

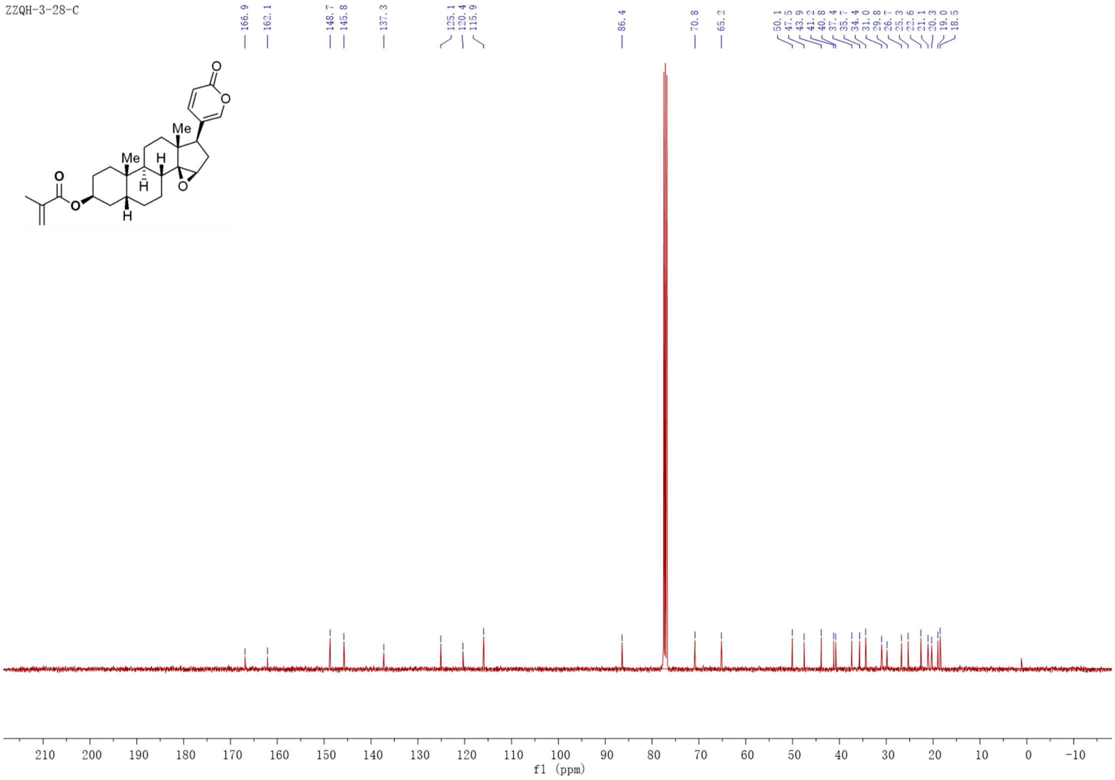

^{1}H NMR Spectrum of 1-7i (400 MHz, $CDCl_3$)

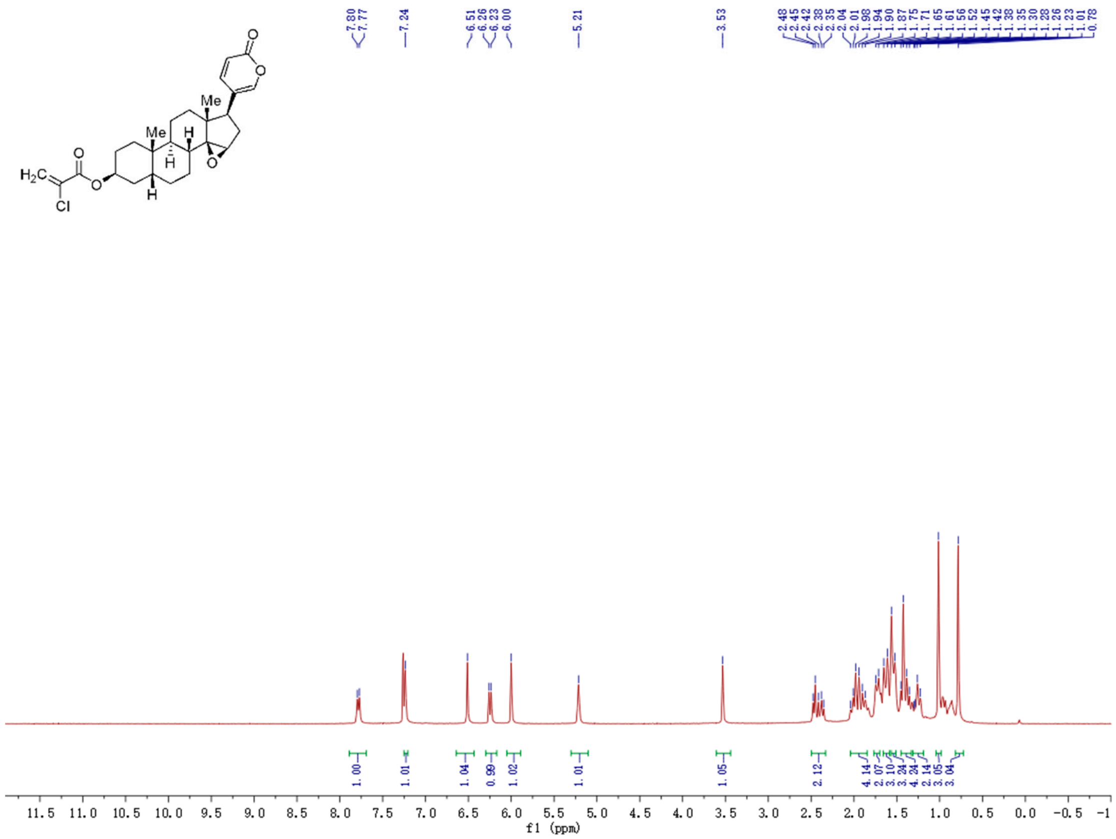

^{13}C NMR Spectrum of 1-7i (101 MHz, $CDCl_3$)

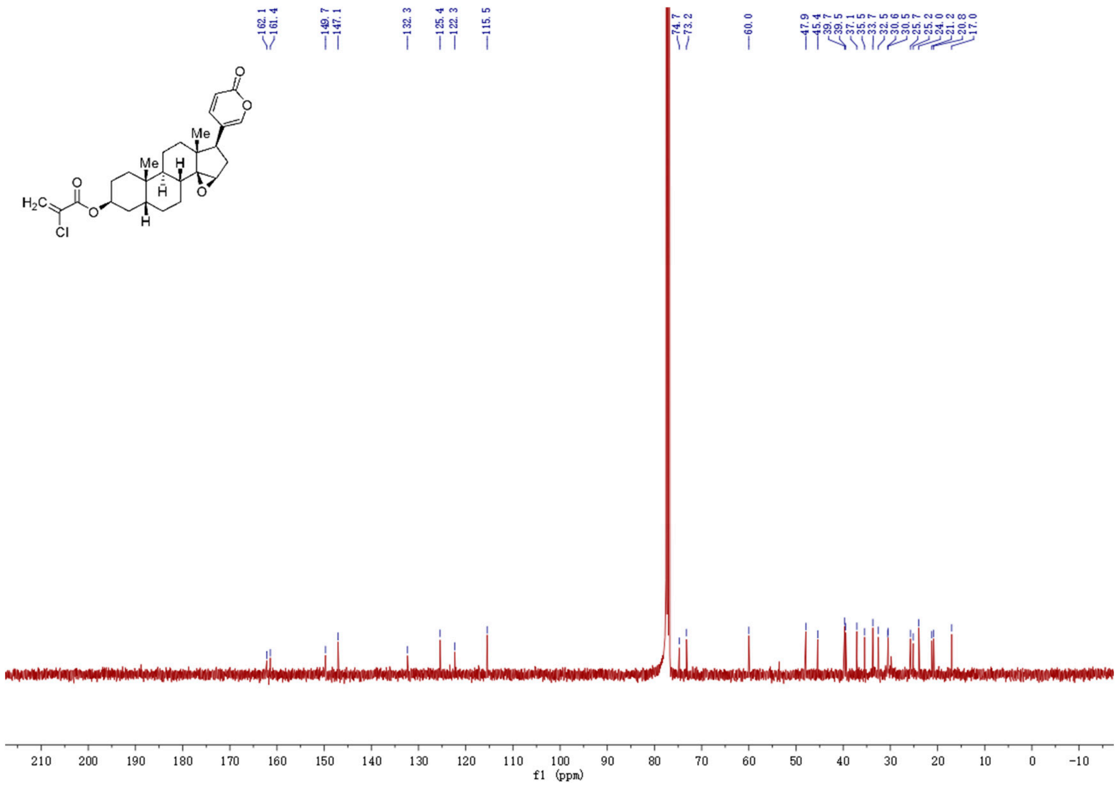

^{1}H NMR Spectrum of 1-7j (400 MHz, $CDCl_3$)

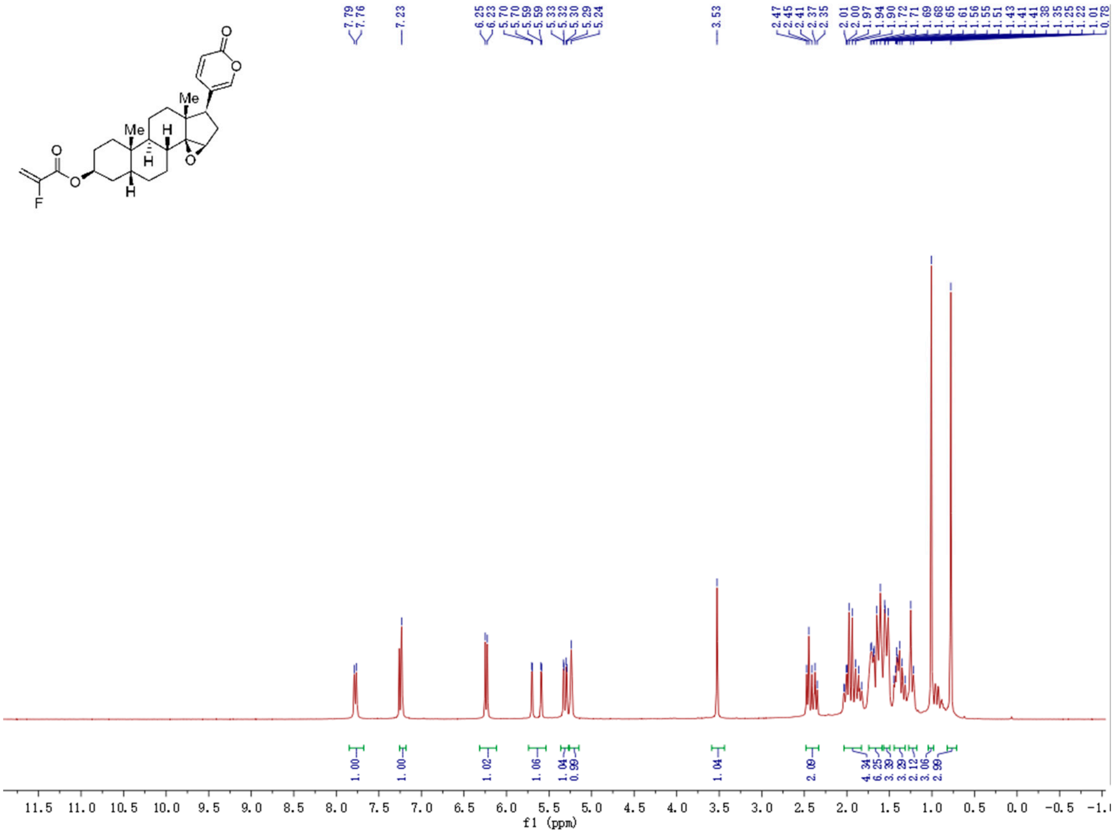

^{13}C NMR Spectrum of 1-7j (101 MHz, $CDCl_3$)

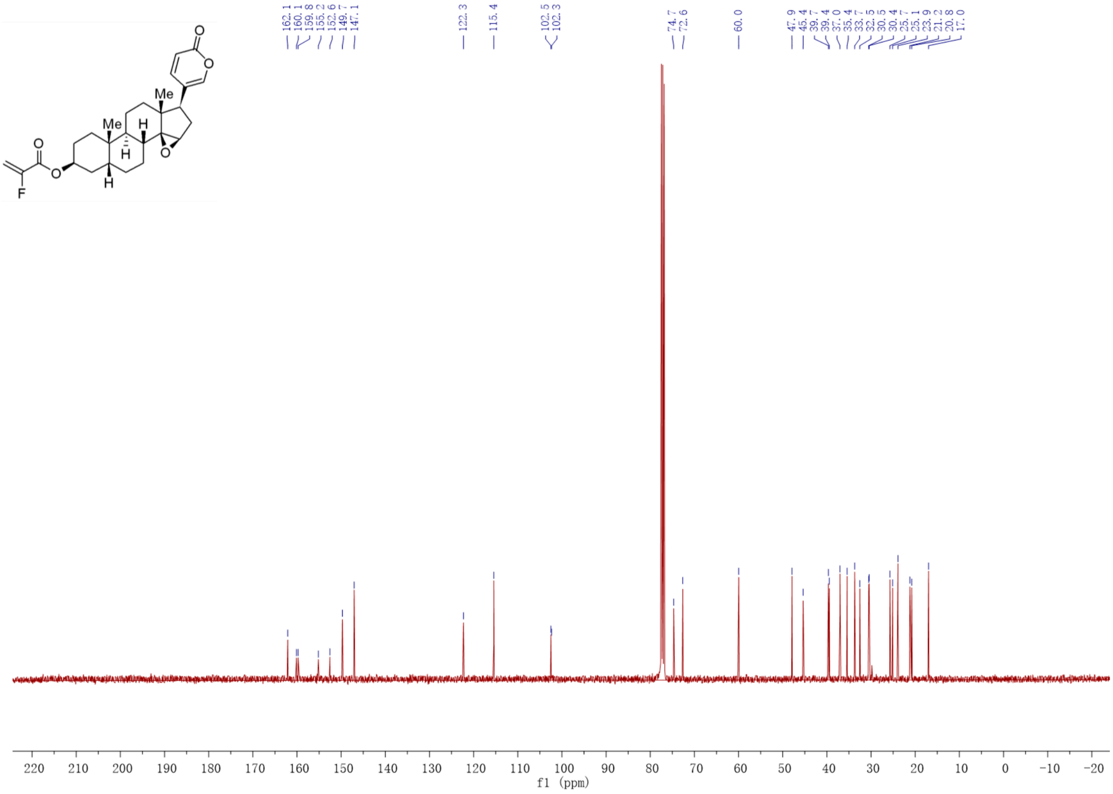

^{1}H NMR Spectrum of 1-7k (400 MHz, $CDCl_3$)

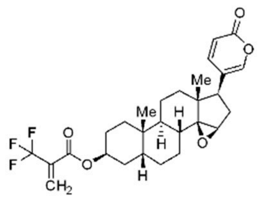

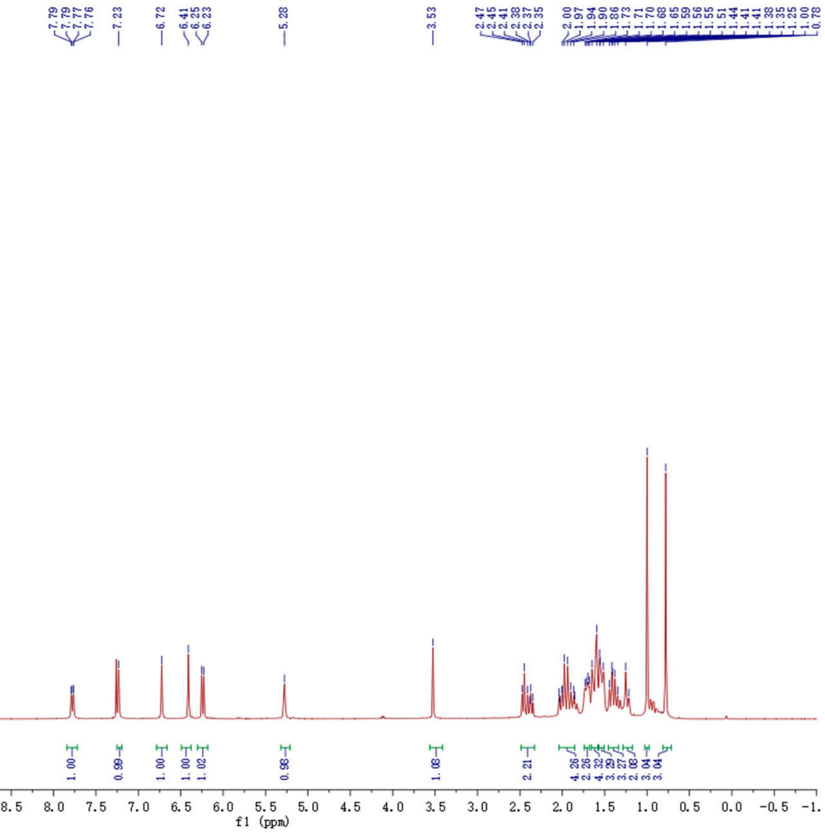

^{13}C NMR Spectrum of 1-7j (101 MHz, $CDCl_3$)

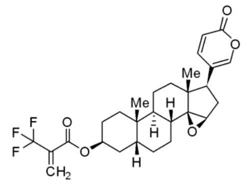

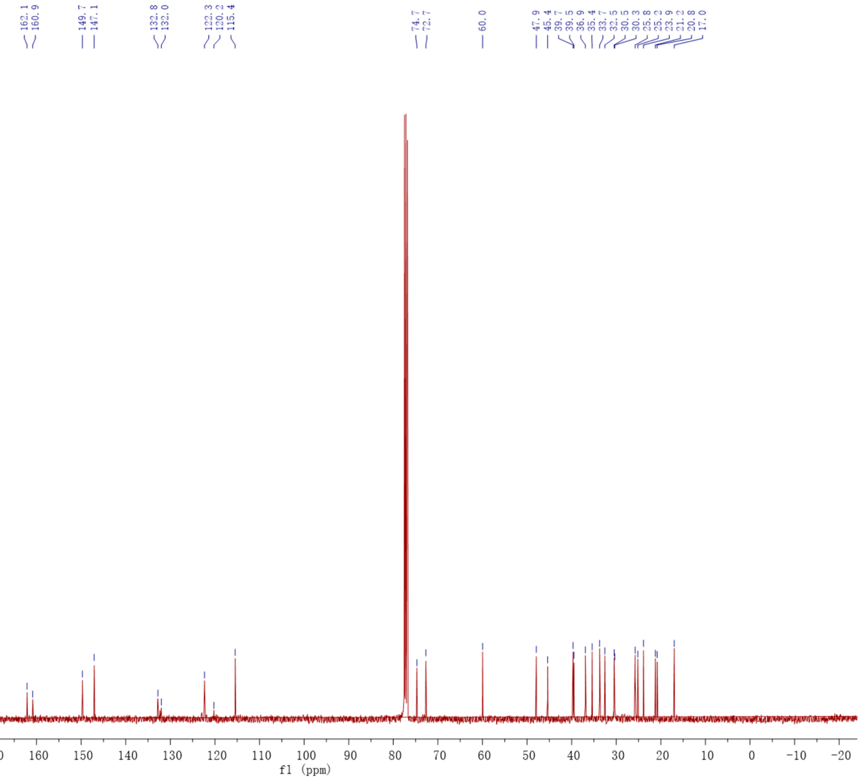

^{1}H NMR Spectrum of 1-7l (400 MHz, $CDCl_3$)

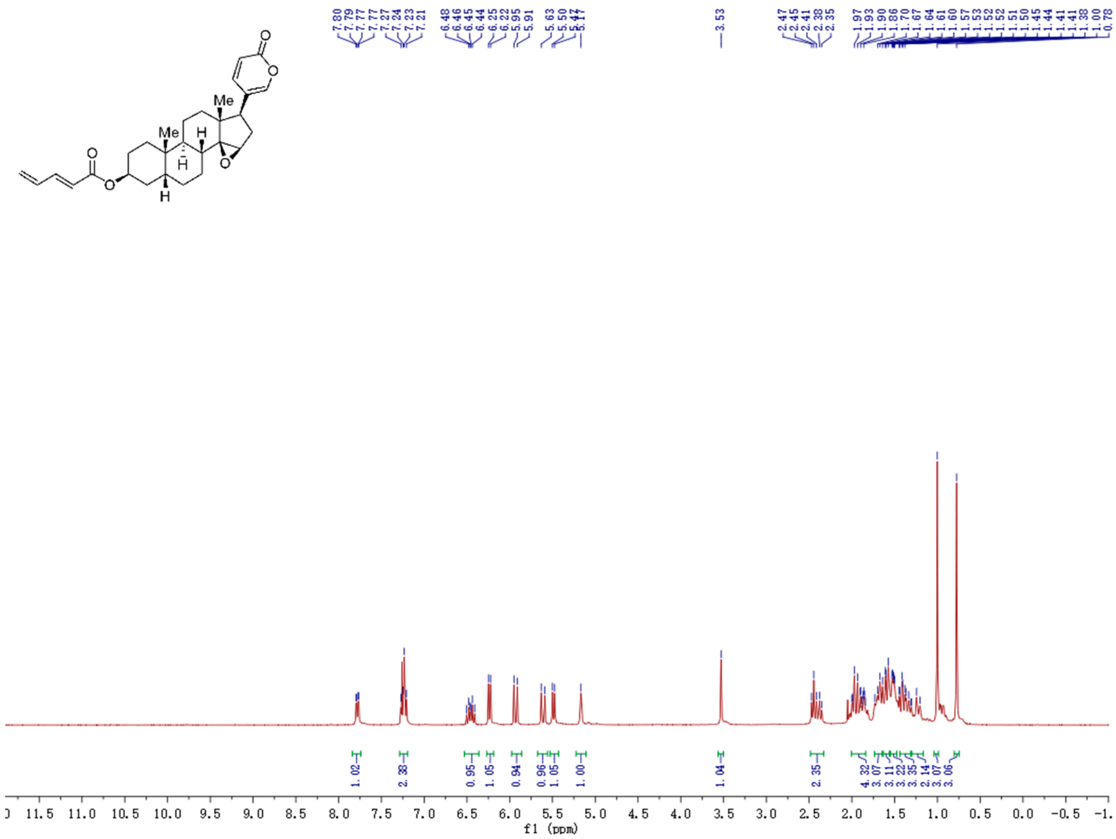

^{13}C NMR Spectrum of 1-7l (101 MHz, $CDCl_3$)

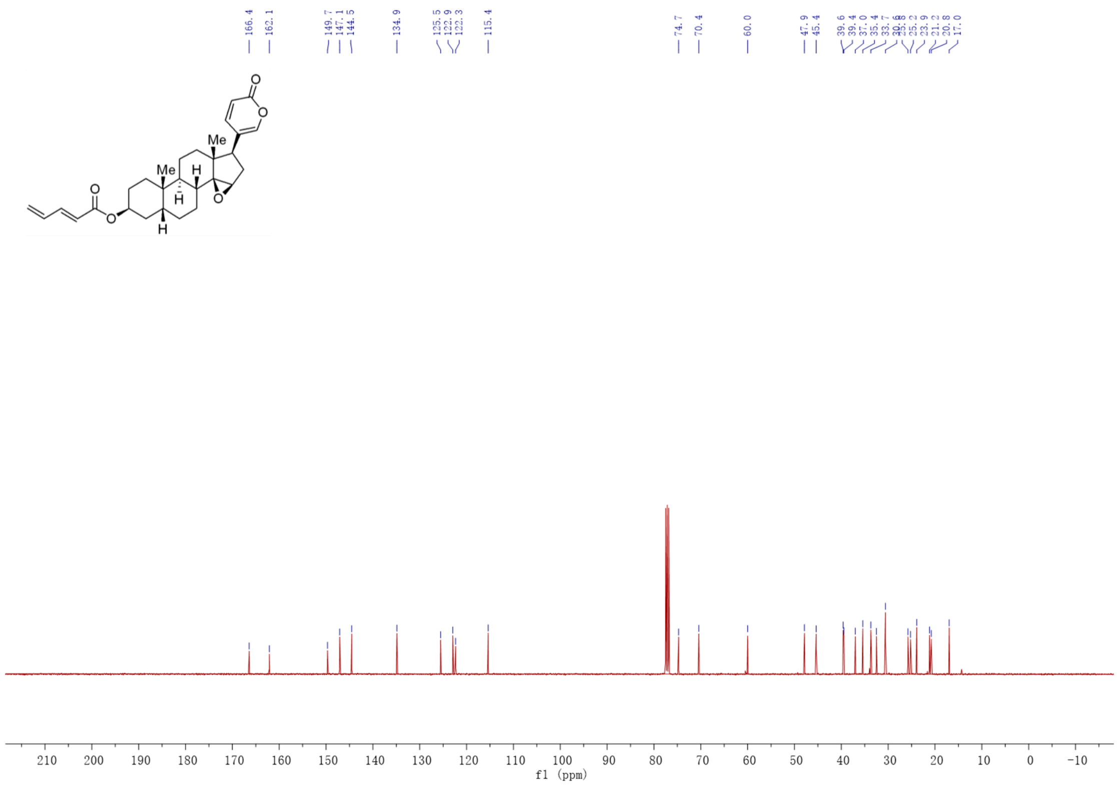

^{1}H NMR Spectrum of 1-7m (400 MHz, $CDCl_3$)

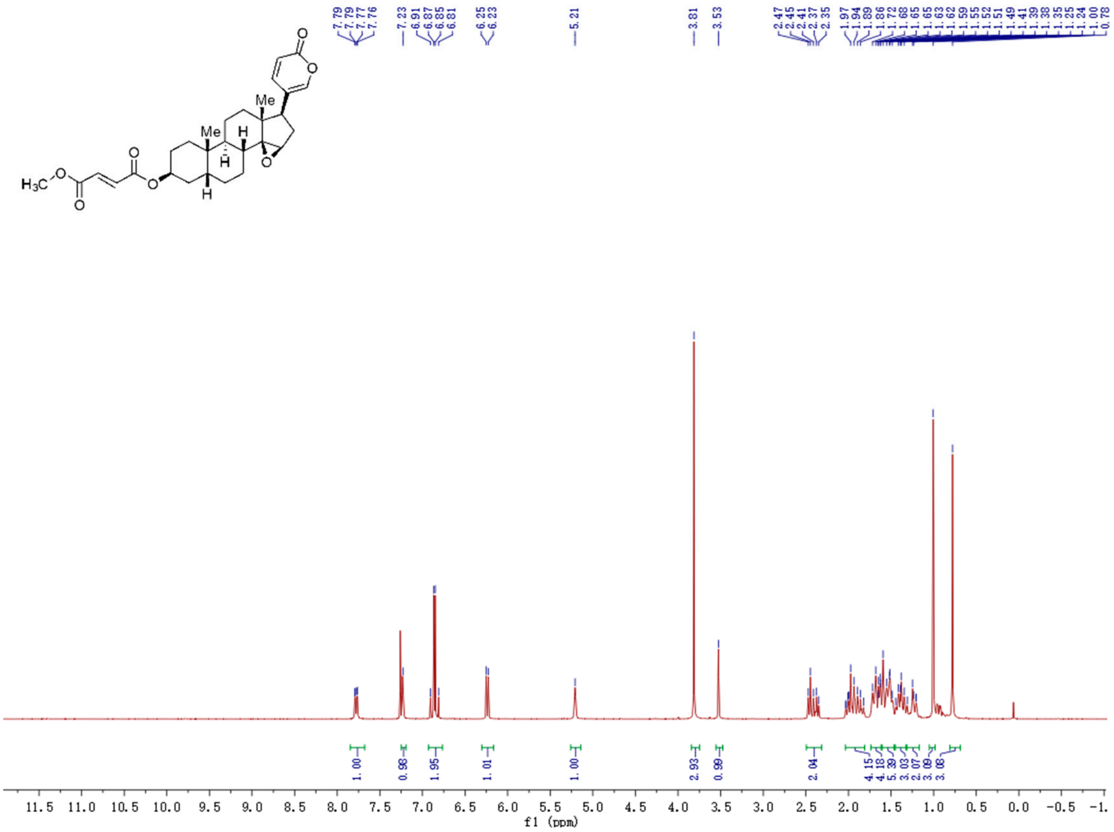

^{13}C NMR Spectrum of 1-7m (101 MHz, $CDCl_3$)

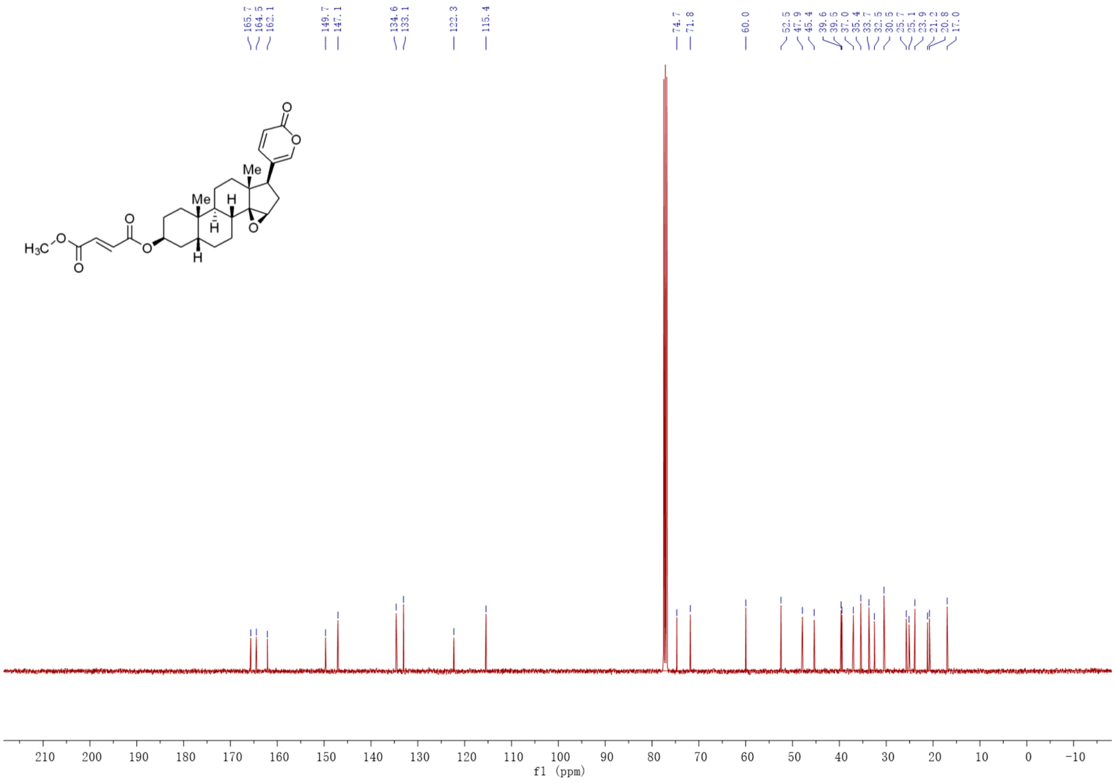

^{1}H NMR Spectrum of 1-7n (400 MHz, $CDCl_3$)

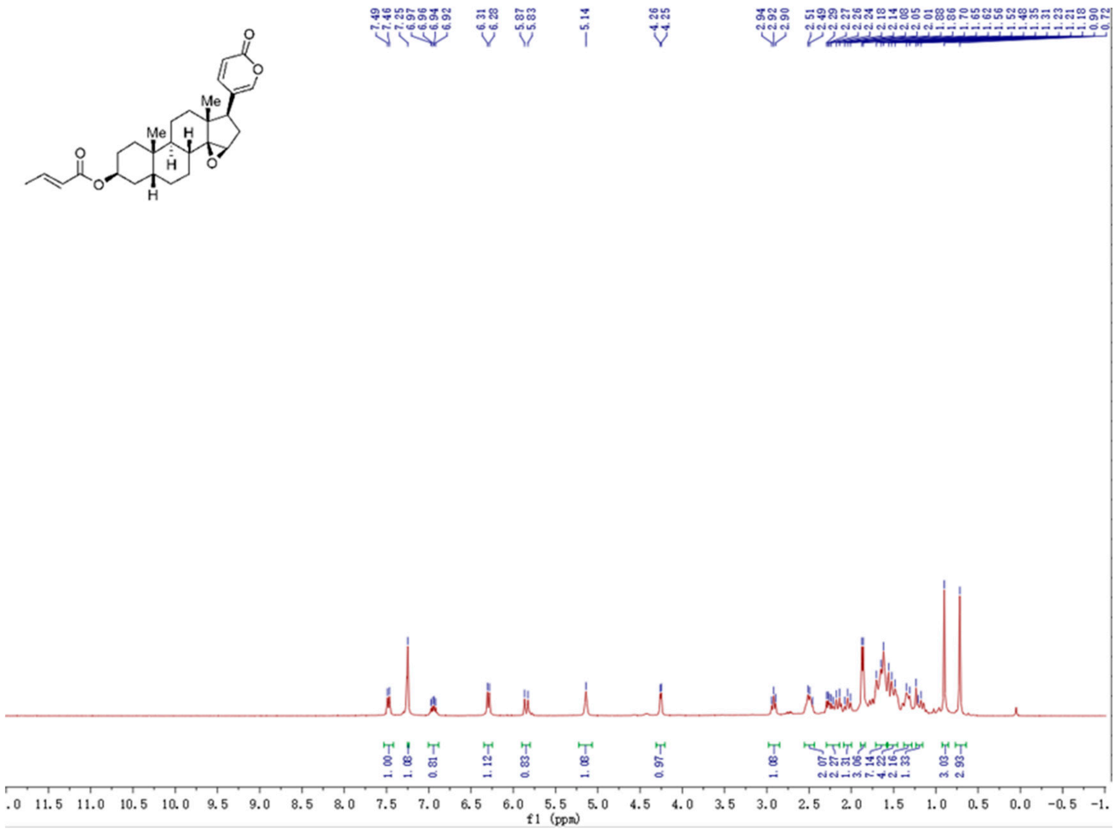

^{13}C NMR Spectrum of 1-7m (101 MHz, $CDCl_3$)

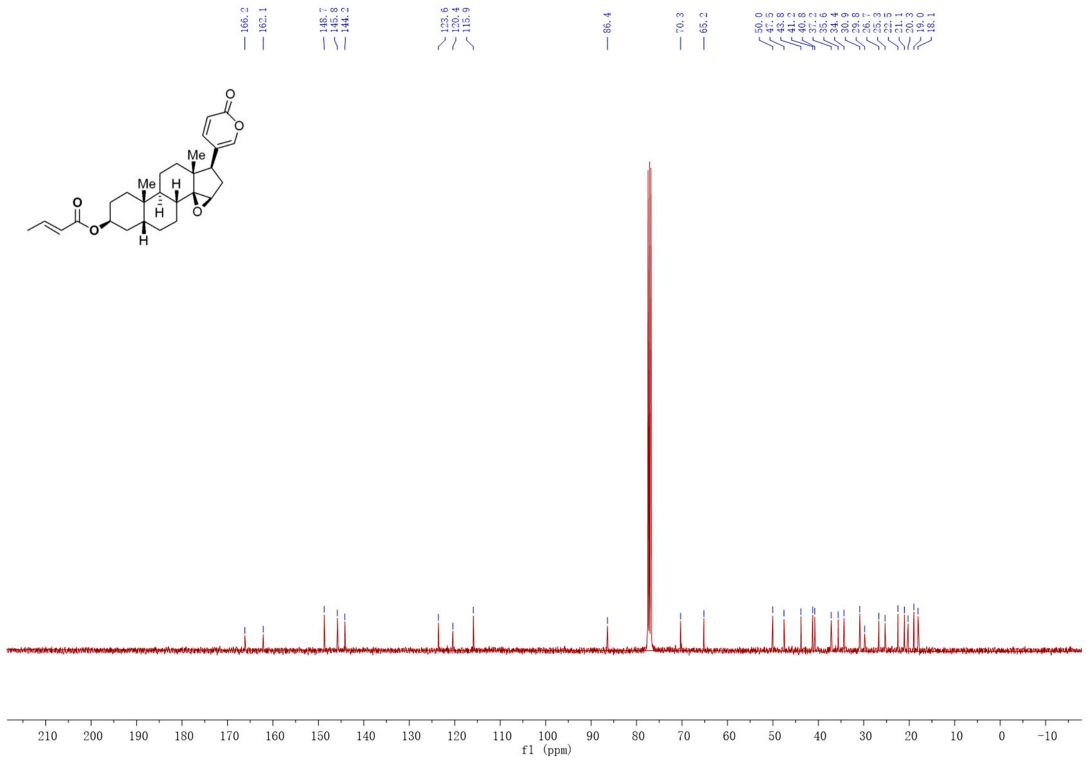

^{1}H NMR Spectrum of 1-7o (400 MHz, $CDCl_3$)

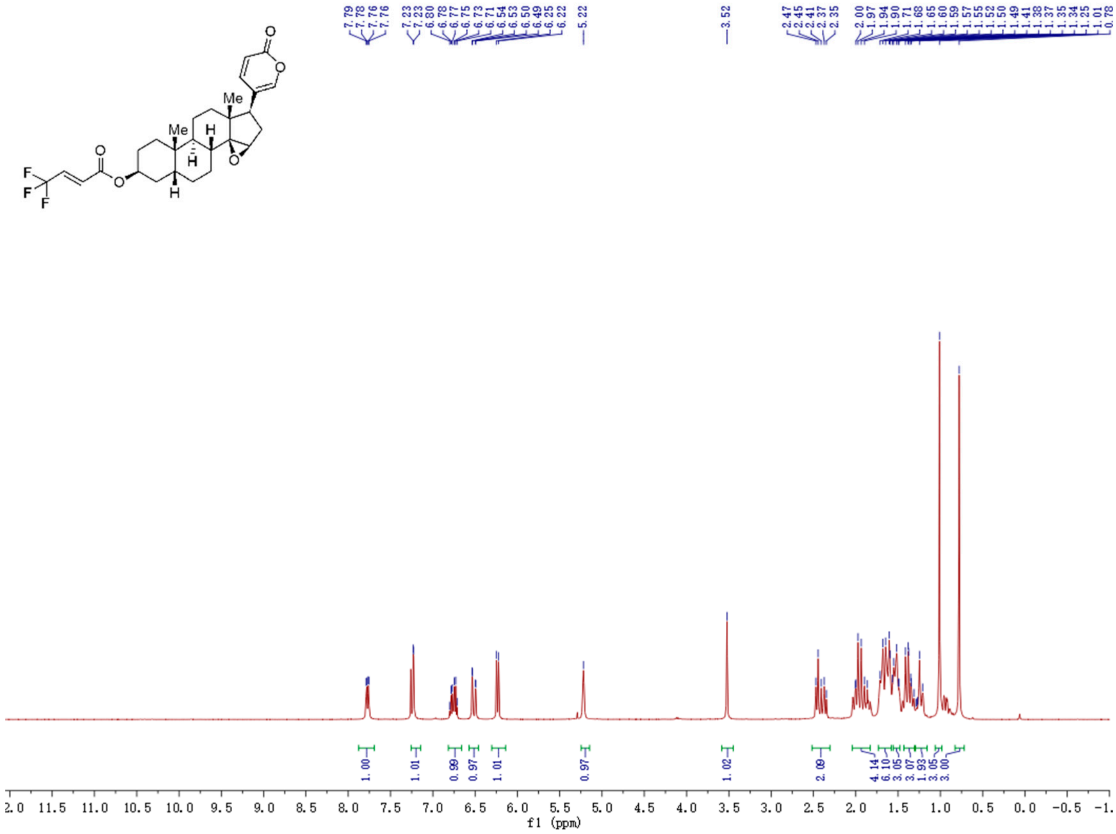

^{13}C NMR Spectrum of 1-7o (101 MHz, $CDCl_3$)

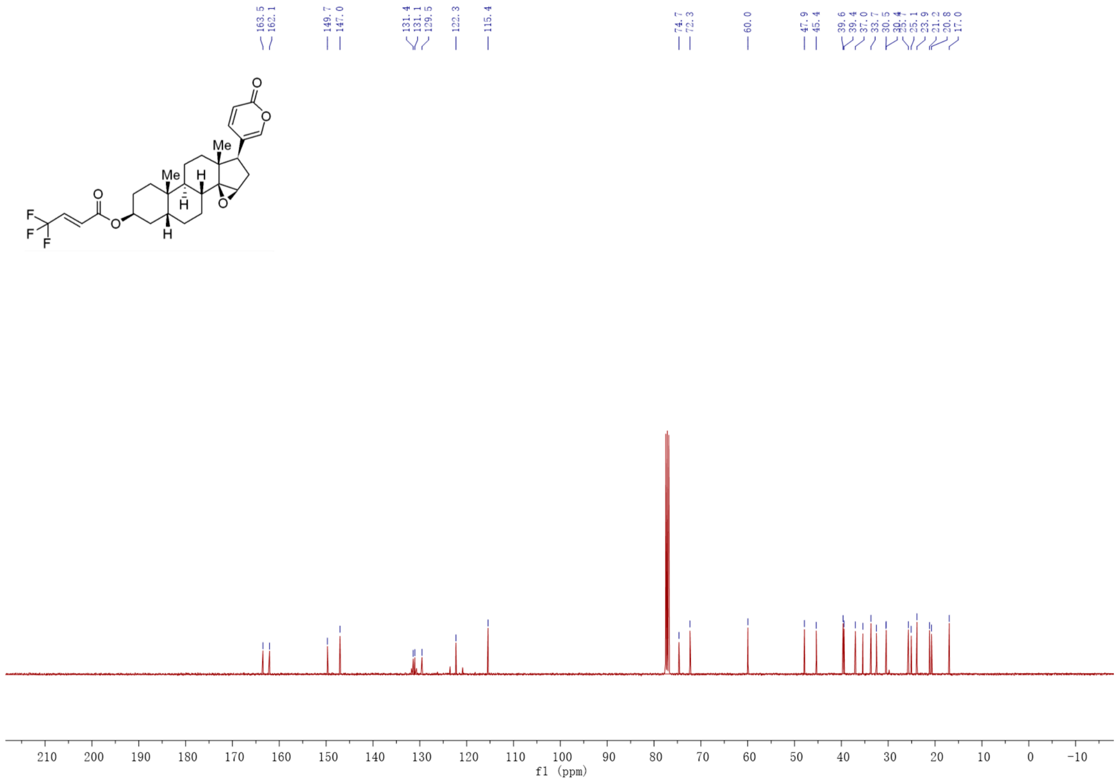

^{1}H NMR Spectrum of 2-1a (400 MHz, $CDCl_3$)

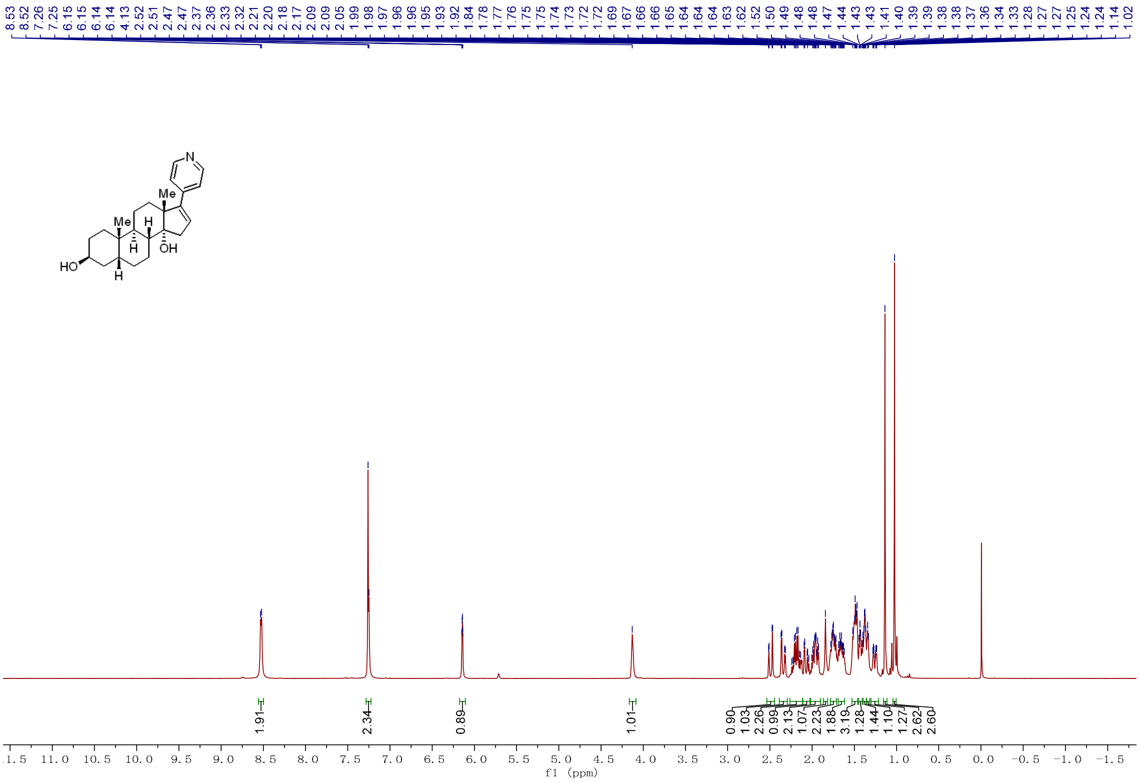

^{13}C NMR Spectrum of 2-1a (101 MHz, $CDCl_3$)

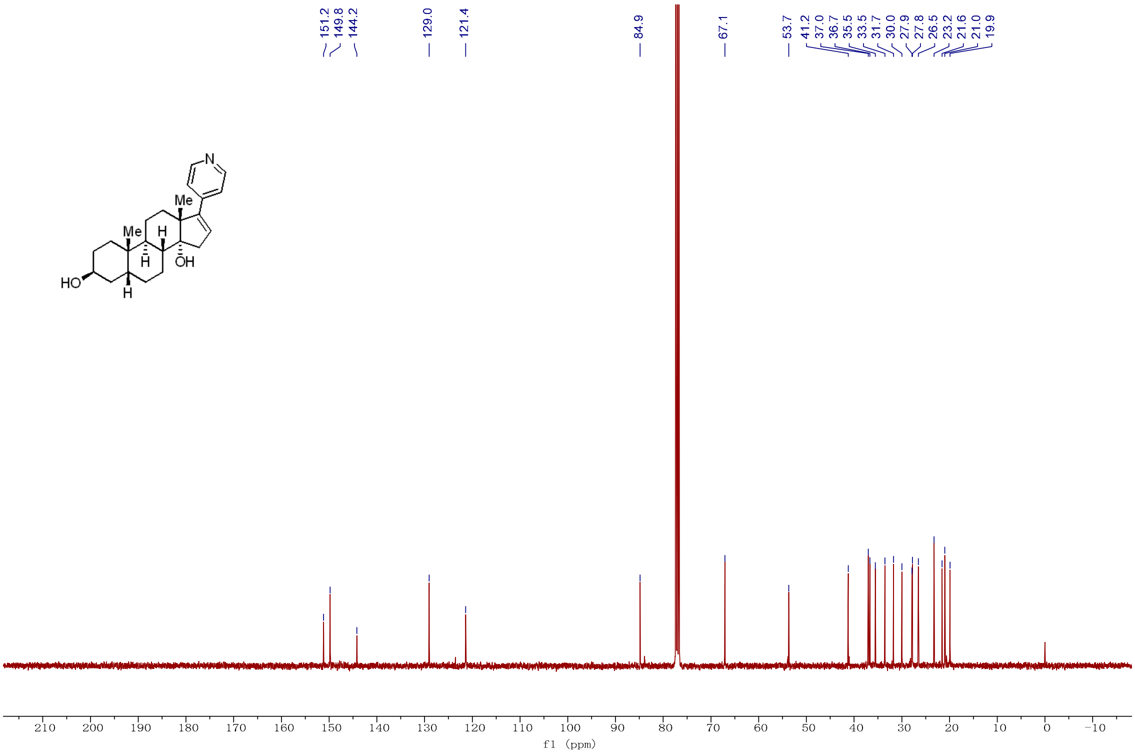

^{1}H NMR Spectrum of 2-1b (600 MHz, $CDCl_3$)

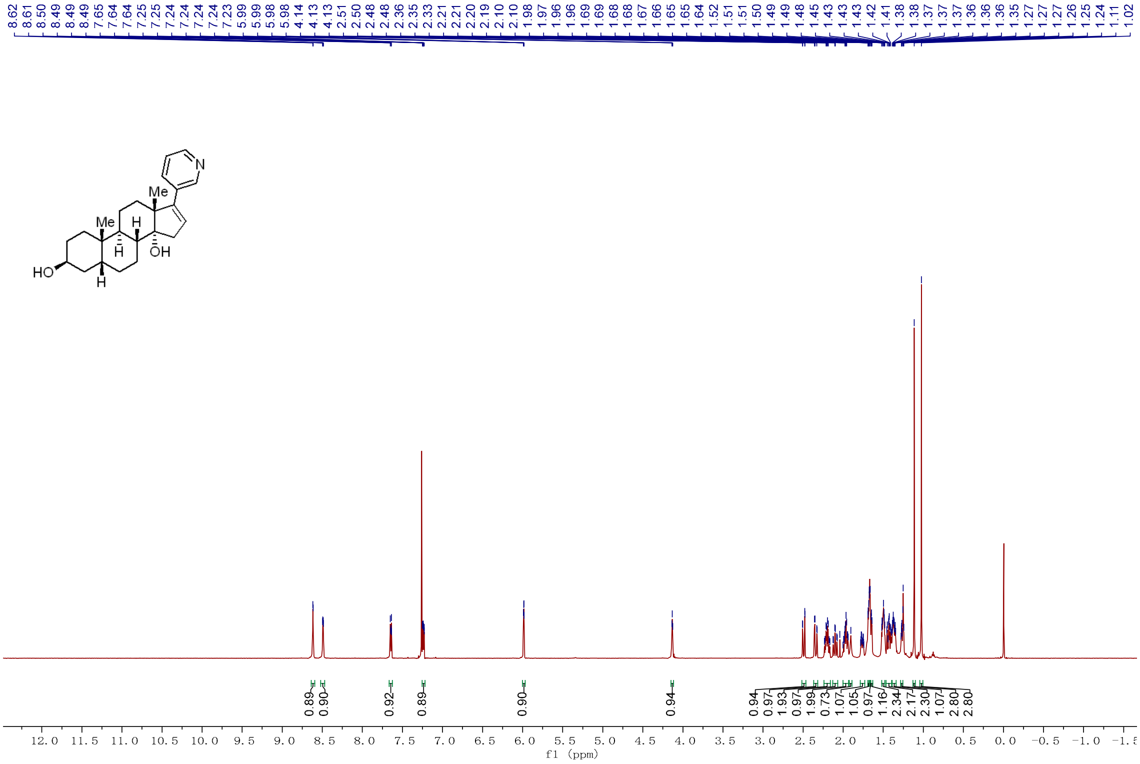

^{13}C NMR Spectrum of 2-1b (101 MHz, $CDCl_3$)

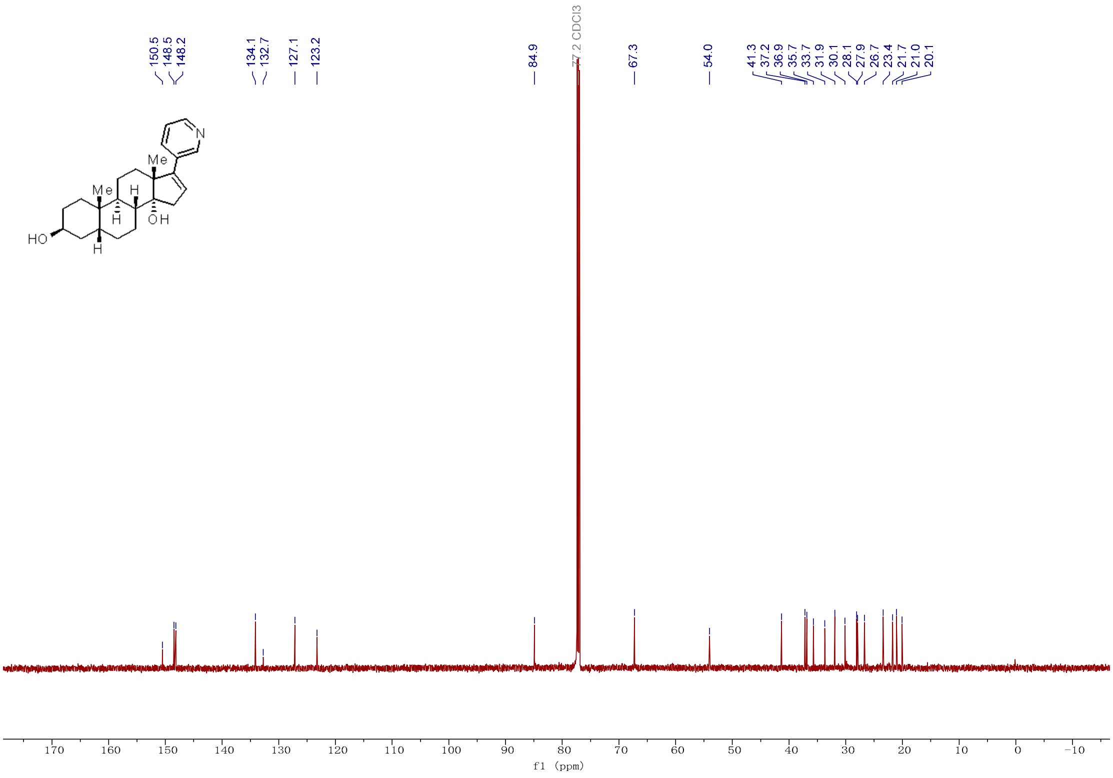

^{1}H NMR Spectrum of 2-1c (400 MHz, $CDCl_3$)

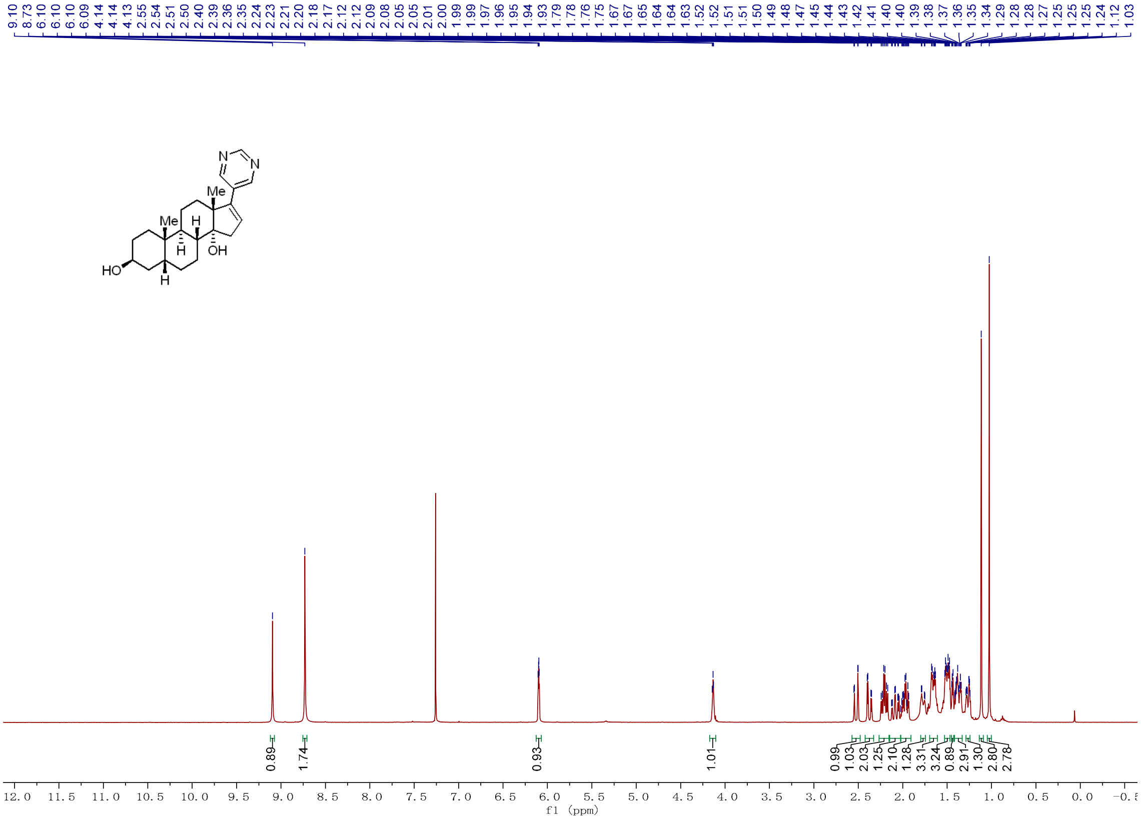

^{13}C NMR Spectrum of 2-1c (101 MHz, $CDCl_3$)

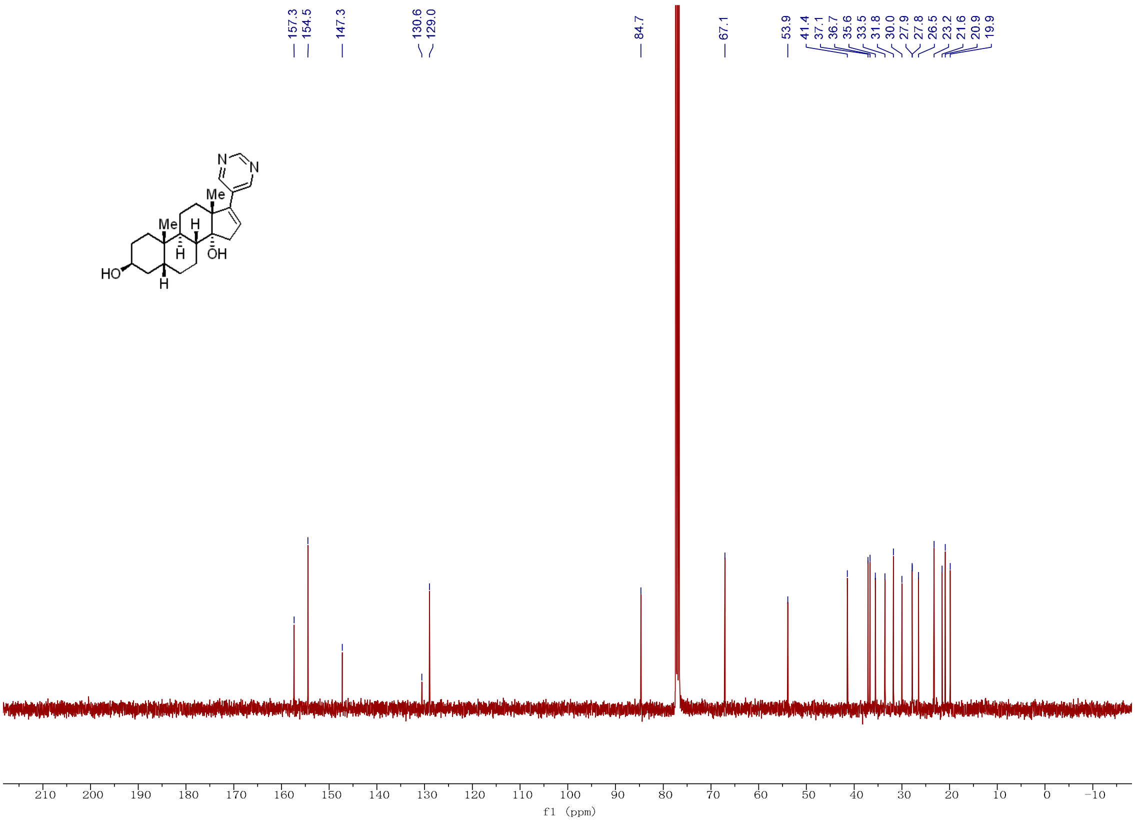

^{1}H NMR Spectrum of 2-1d (400 MHz, $CDCl_3$)

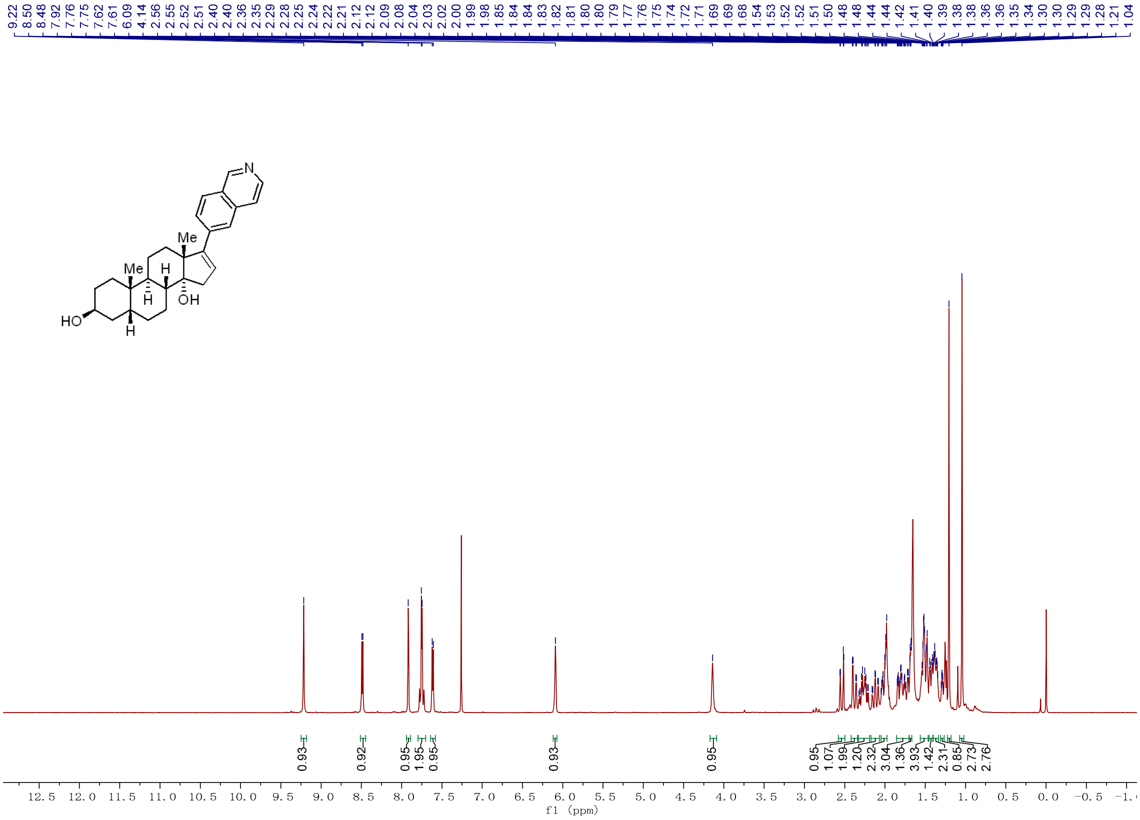

^{13}C NMR Spectrum of 2-1d (101 MHz, $CDCl_3$)

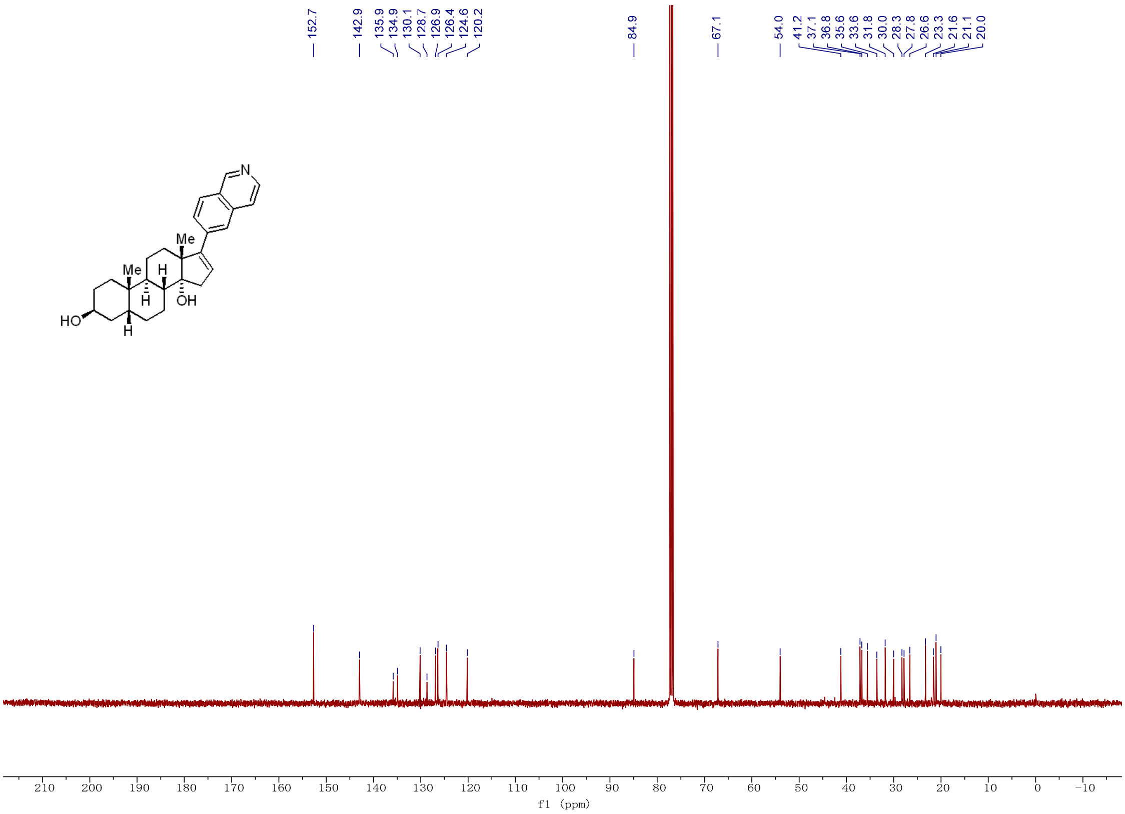

^{1}H NMR Spectrum of 2-1e (400 MHz, $CDCl_3$)

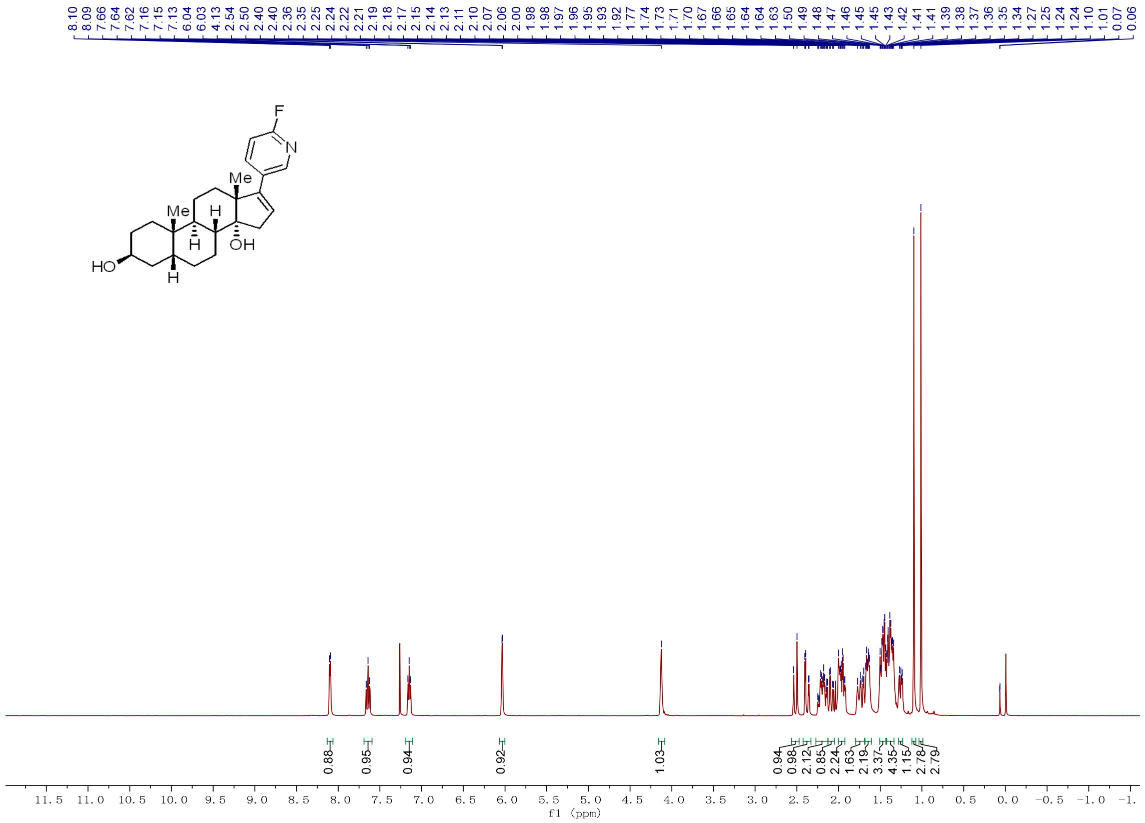

^{13}C NMR Spectrum of 2-1e (101 MHz, $CDCl_3$)

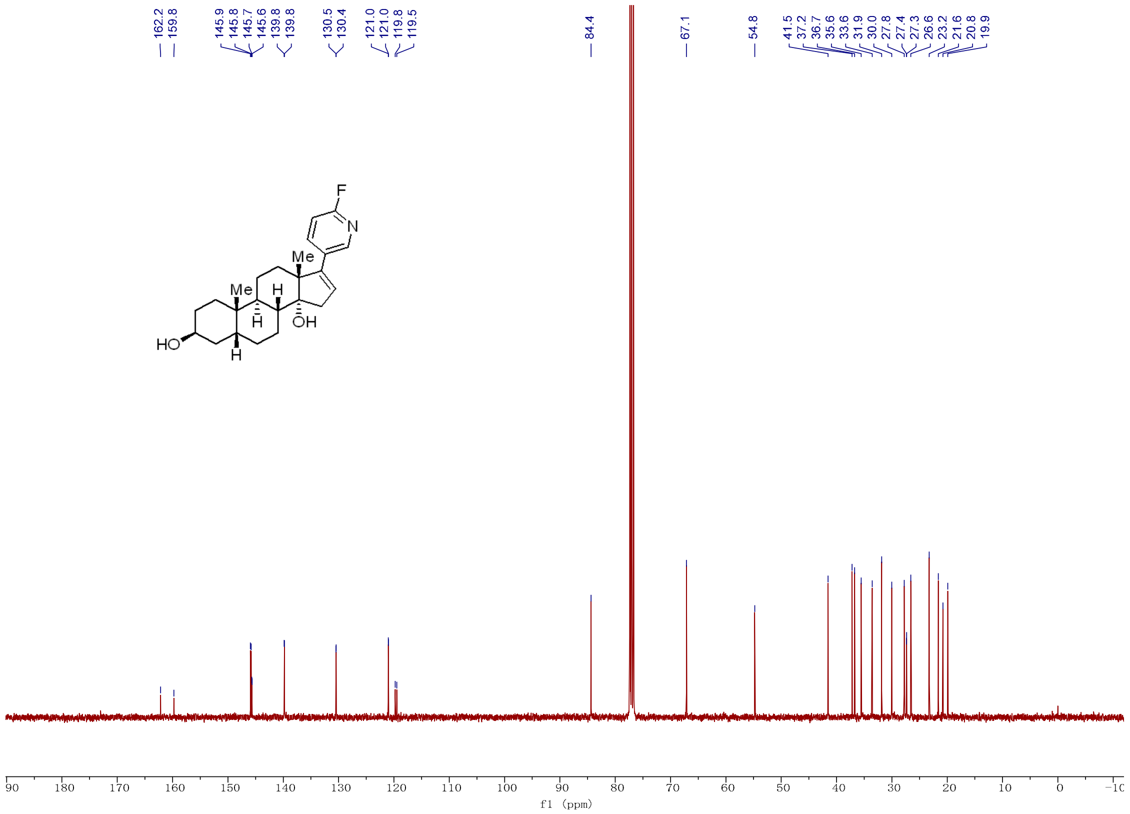

^{1}H NMR Spectrum of 2-1f (400 MHz, $CDCl_3$)

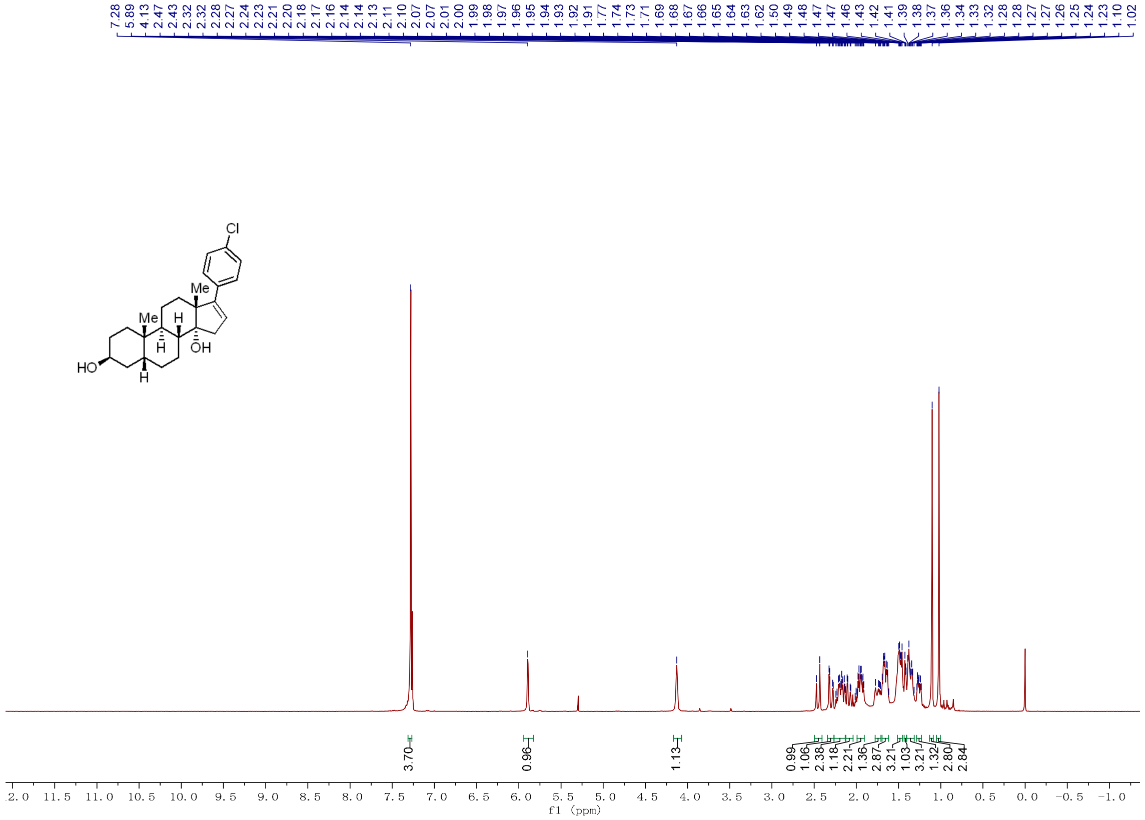

^{13}C NMR Spectrum of 2-1f (101 MHz, $CDCl_3$)

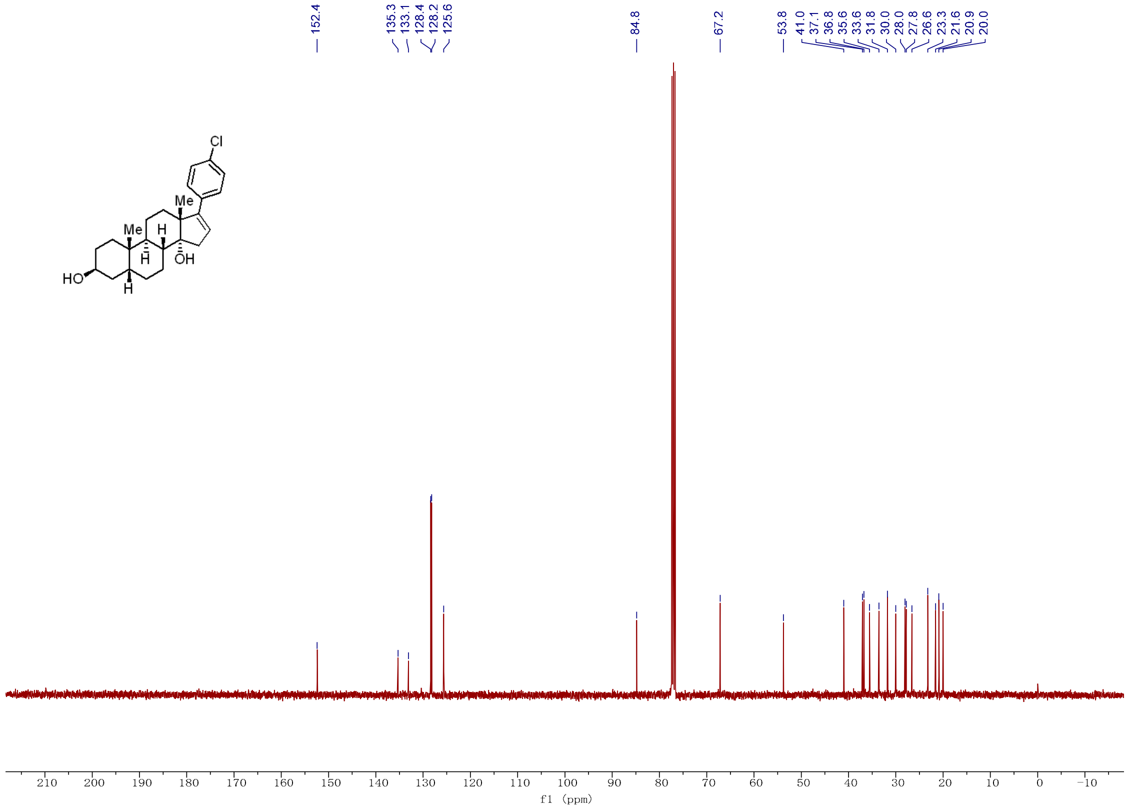

^{1}H NMR Spectrum of 2-1g (400 MHz, $CDCl_3$)

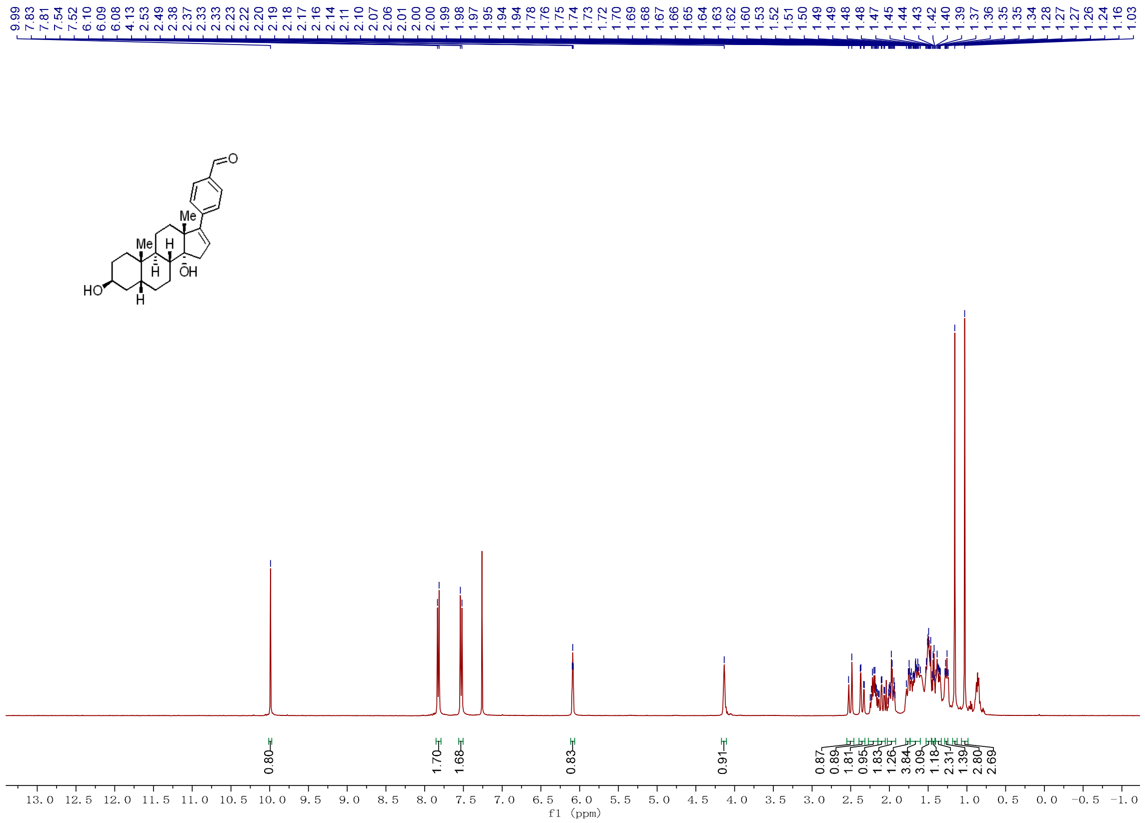

^{13}C NMR Spectrum of 2-1g (101 MHz, $CDCl_3$)

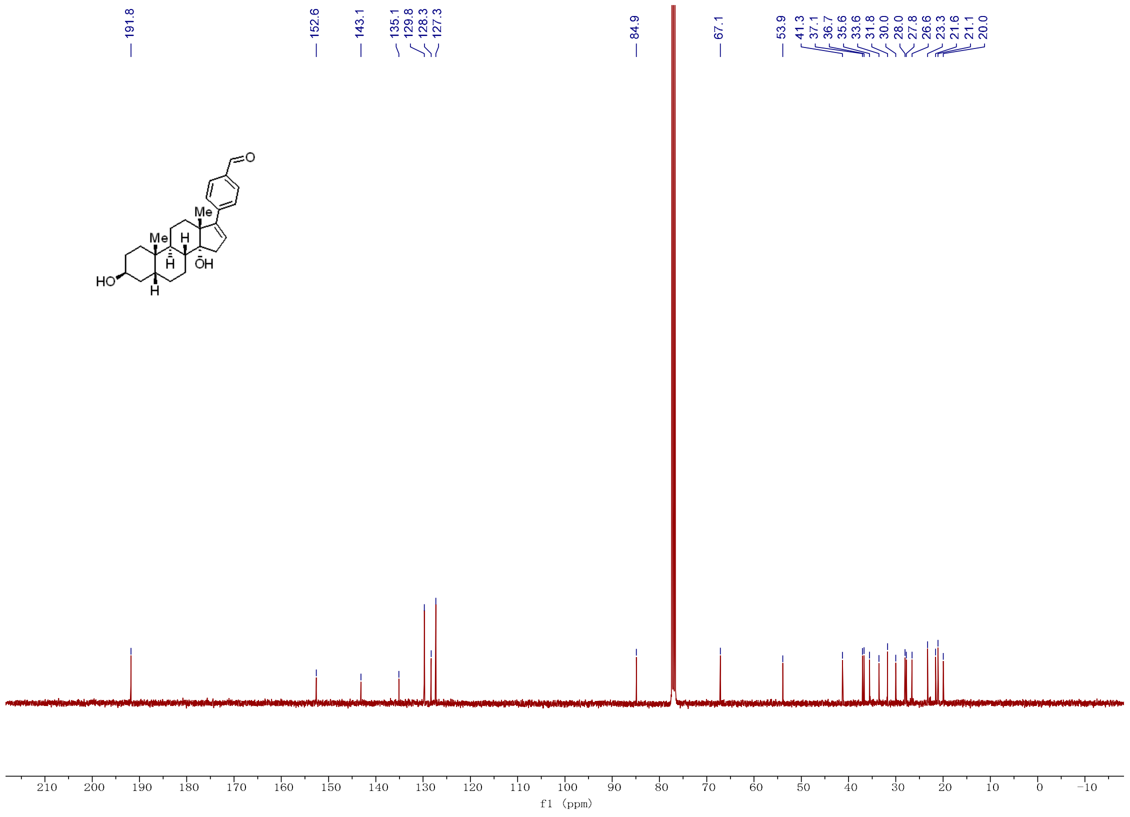

^{1}H NMR Spectrum of 2-1h (400 MHz, $CDCl_3$)

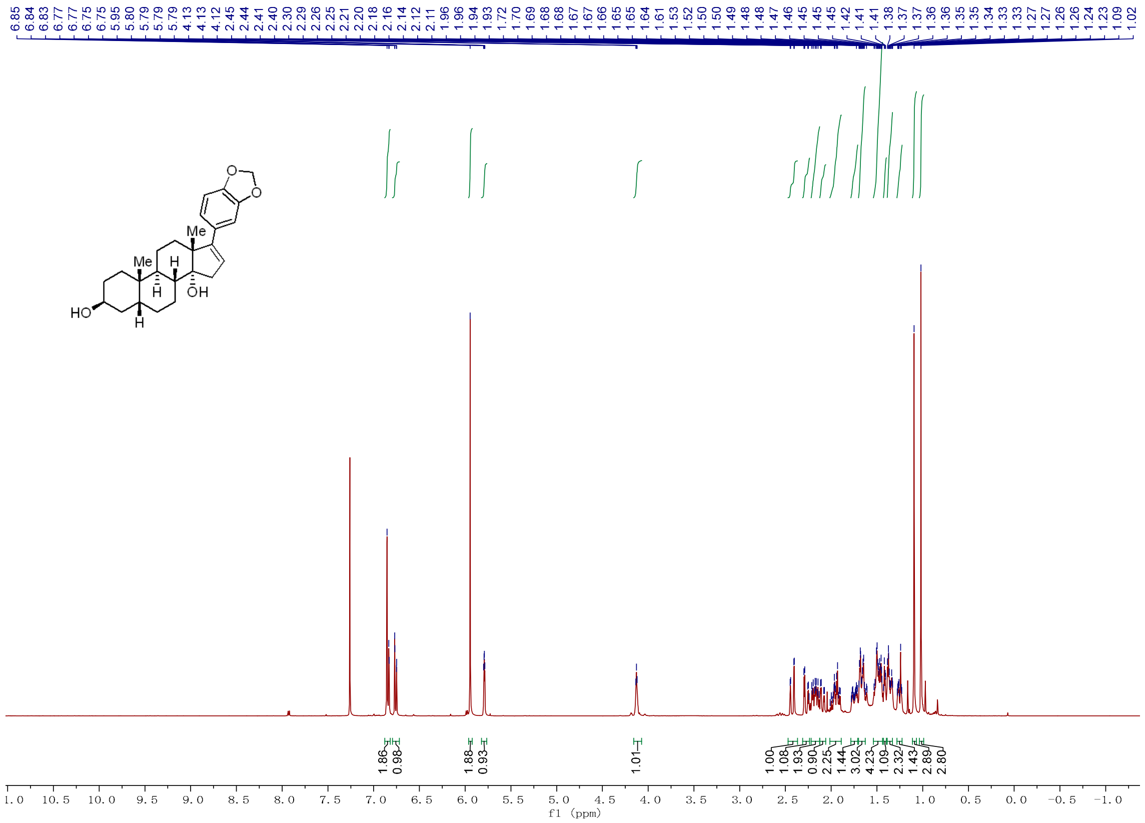

^{13}C NMR Spectrum of 2-1h (101 MHz, $CDCl_3$)

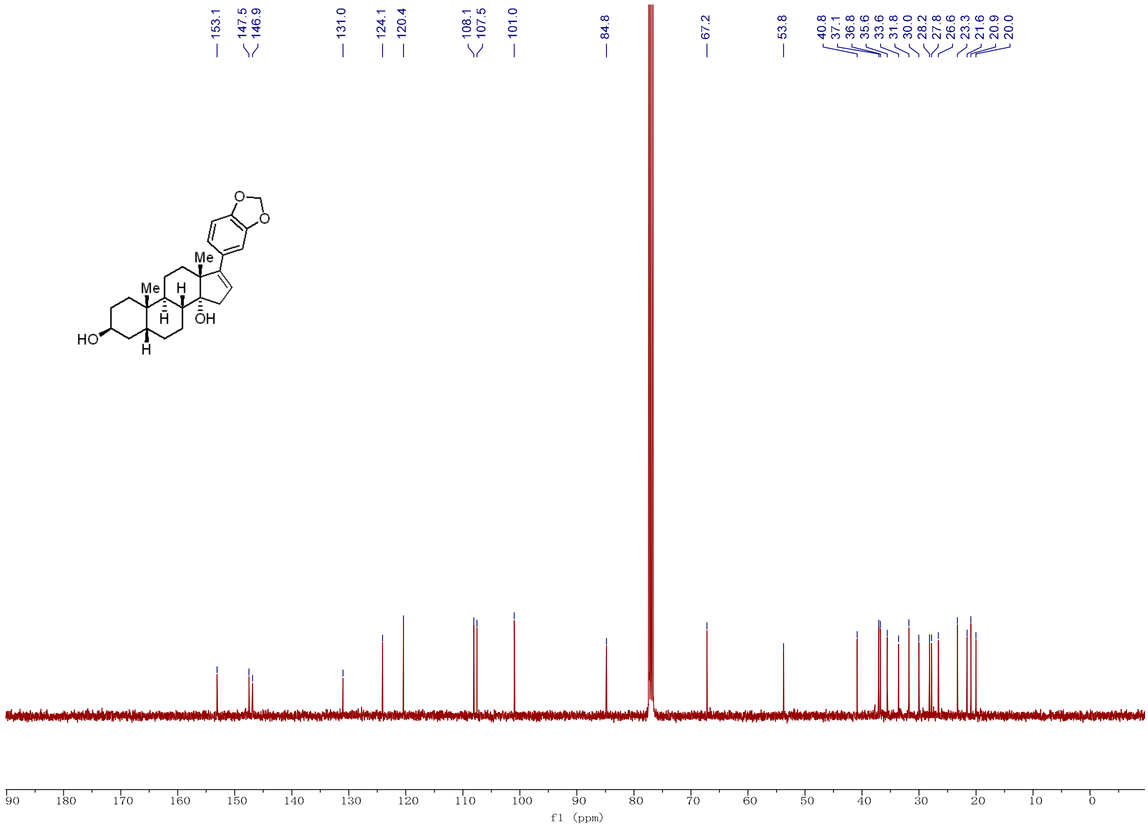

^{1}H NMR Spectrum of 2-1i (400 MHz, $CDCl_3$)

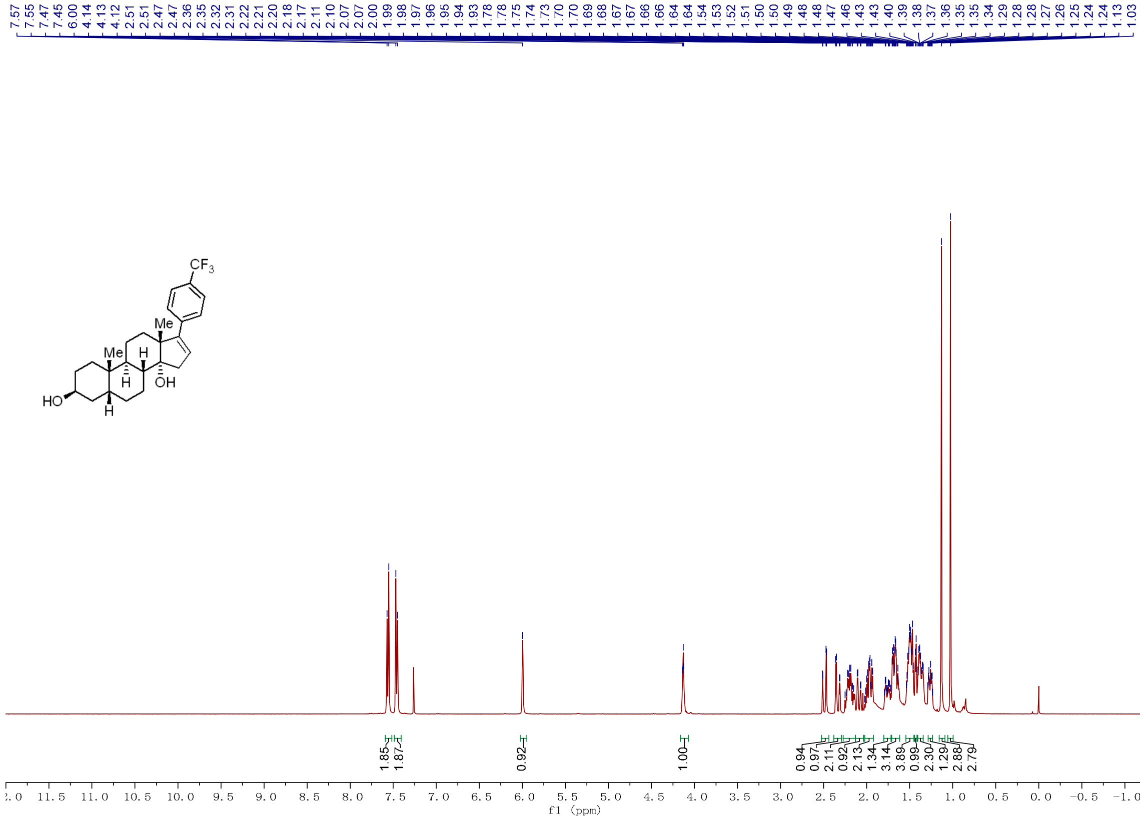

^{13}C NMR Spectrum of 2-1i (101 MHz, $CDCl_3$)

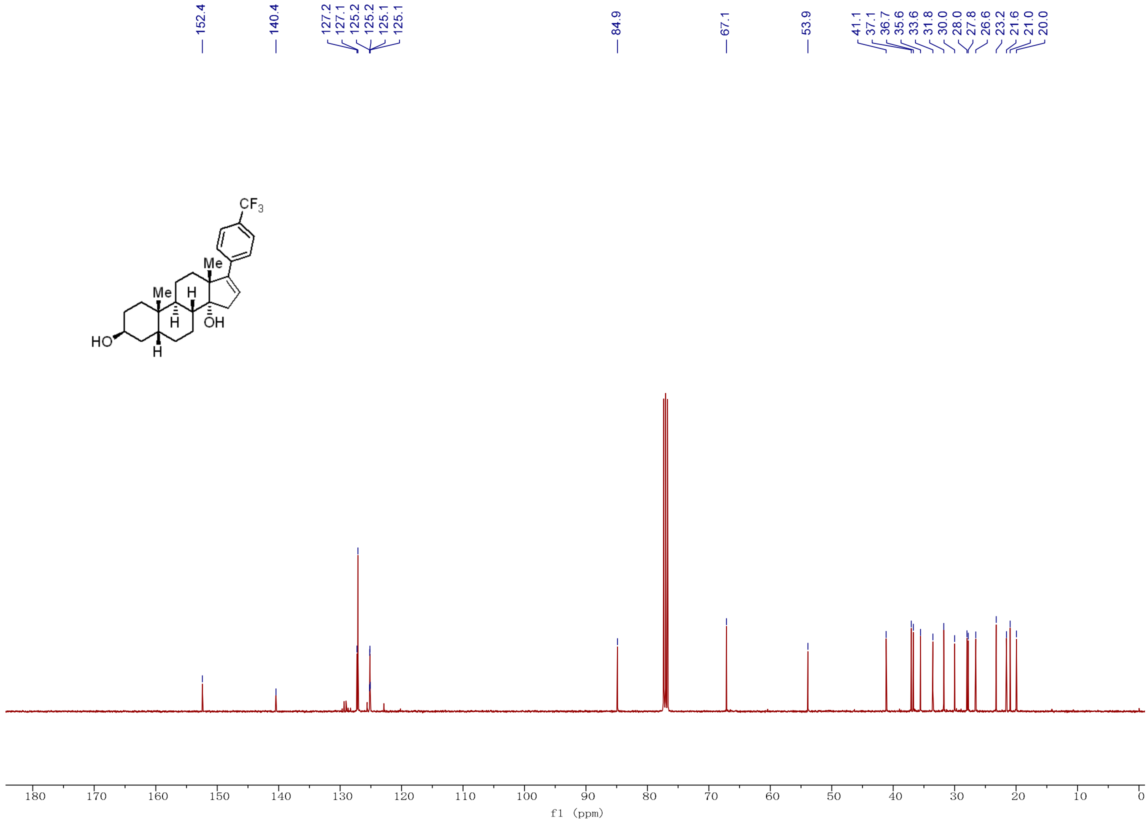

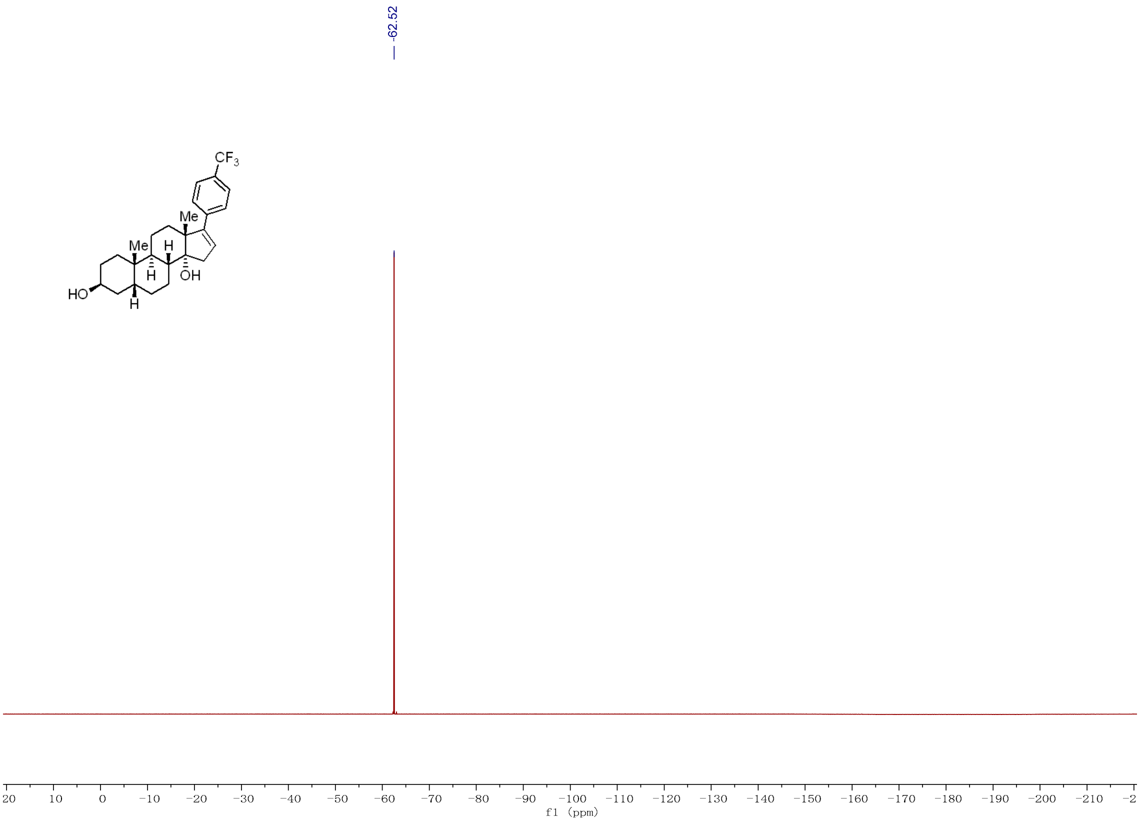

CF3
Me
Me
H
H
OH
HO
H
-62.52
20
10
0
−10
−20
−30
−40
−50
−60
−70
−80
−90
−100
−110
−120
−130
−140
−150
−160
−170
−180
−190
−200
−210
−2
f1 (ppm)

^{1}H NMR Spectrum of 2-1j (400 MHz, $CDCl_3$)

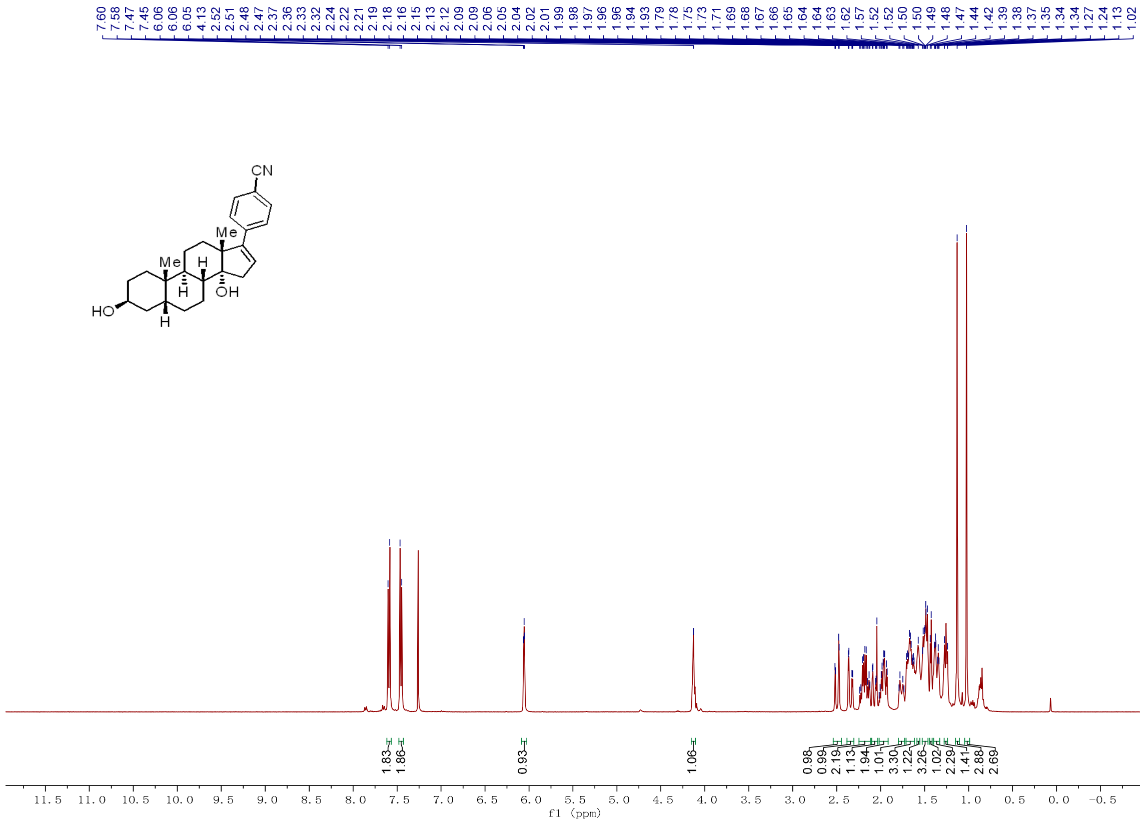

^{13}C NMR Spectrum of 2-1j (101 MHz, $CDCl_3$)

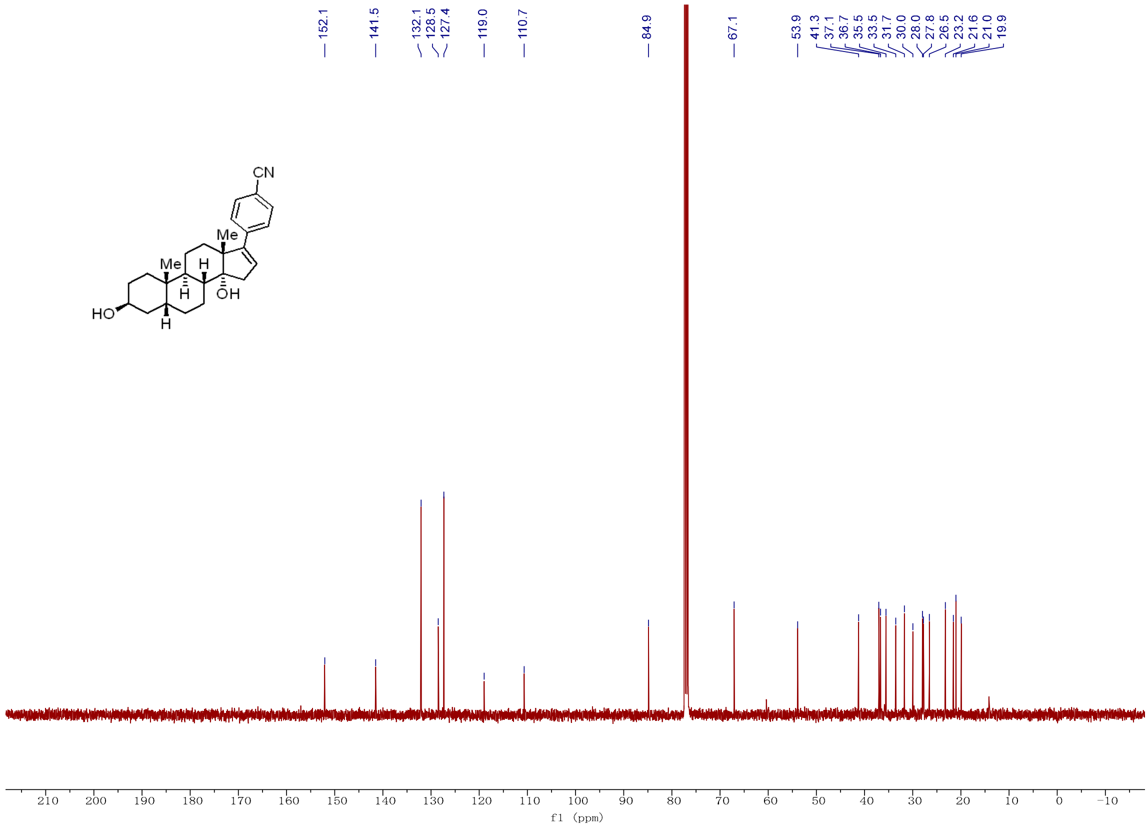

^{1}H NMR Spectrum of 2-1k (400 MHz, $CDCl_3$)

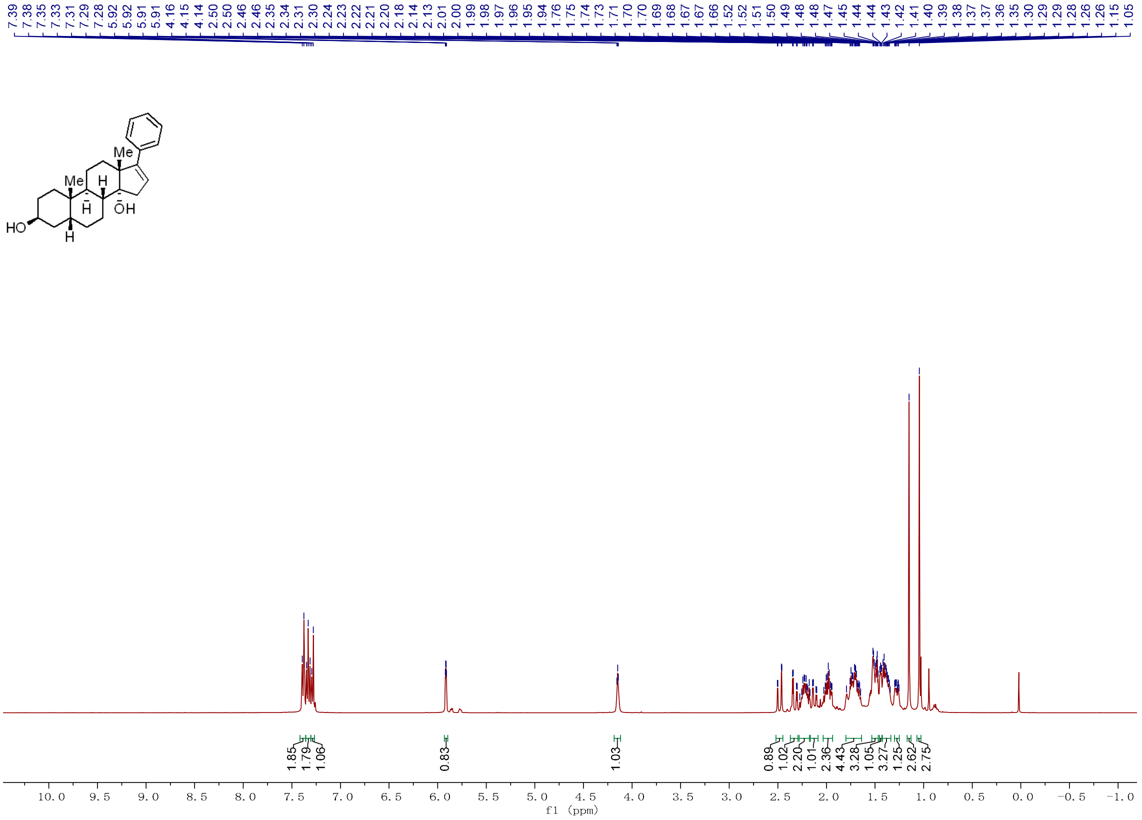

^{13}C NMR Spectrum of 2-1k (101 MHz, $CDCl_3$)

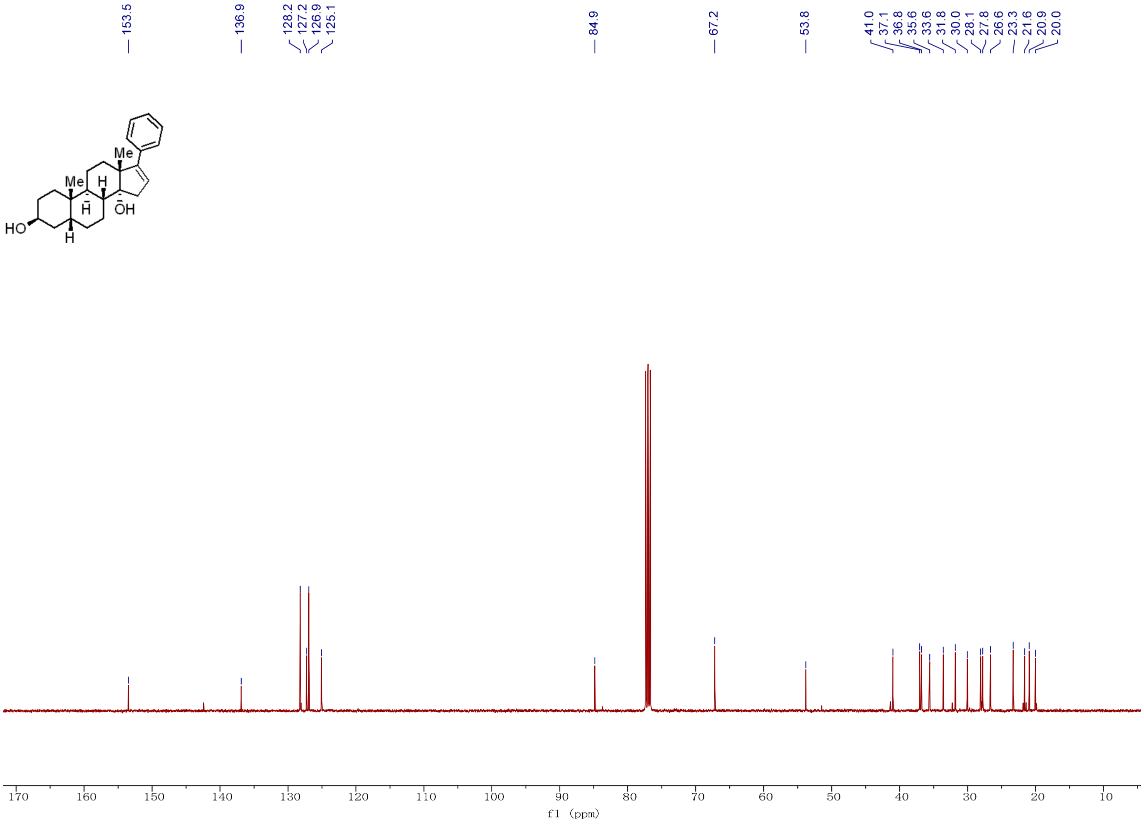

^{1}H NMR Spectrum of 2-1l (400 MHz, $CDCl_3$)

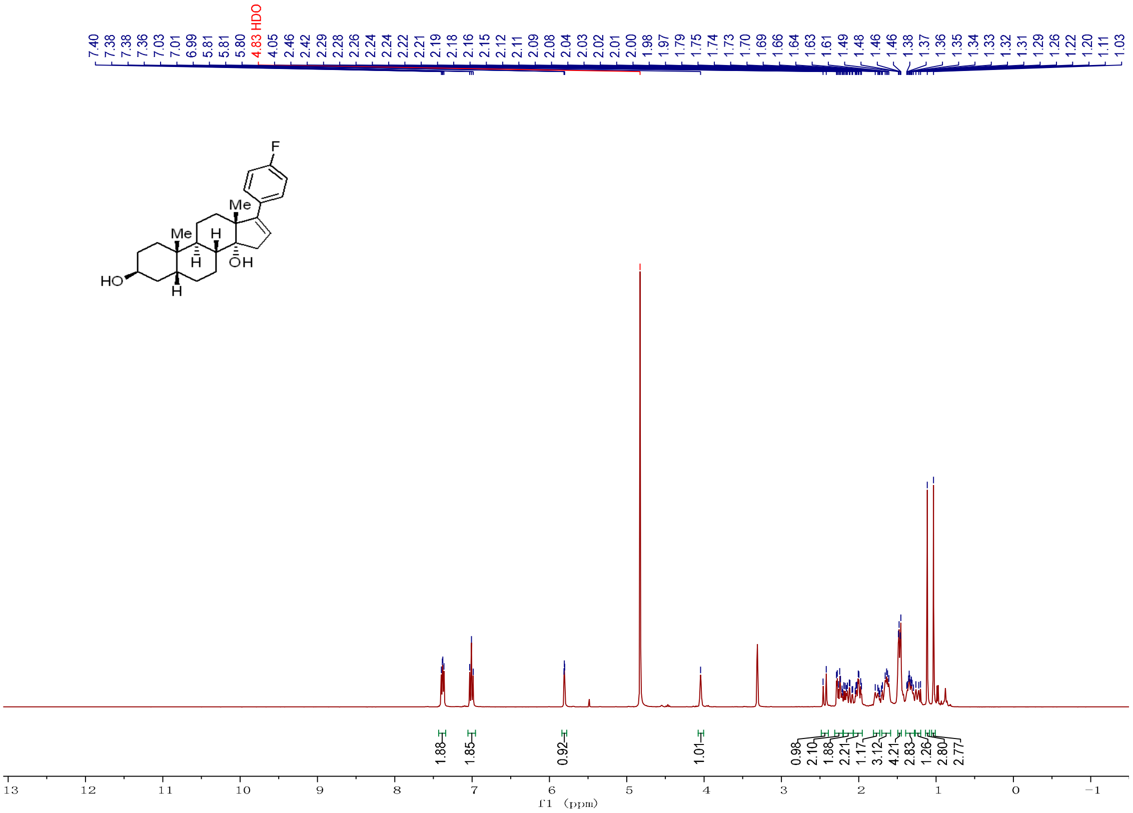

^{13}C NMR Spectrum of 2-1l (101 MHz, MeOD)

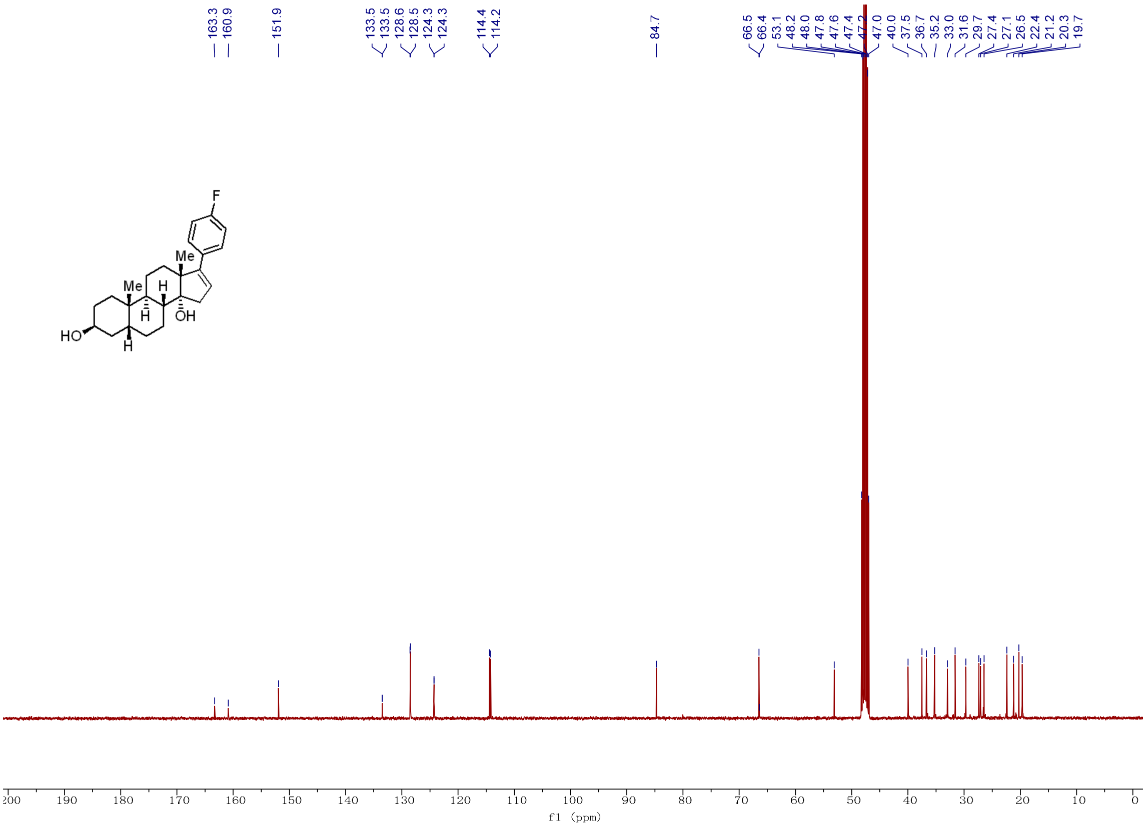

^{1}H NMR Spectrum of 2-2a (400 MHz, $CDCl_3$)

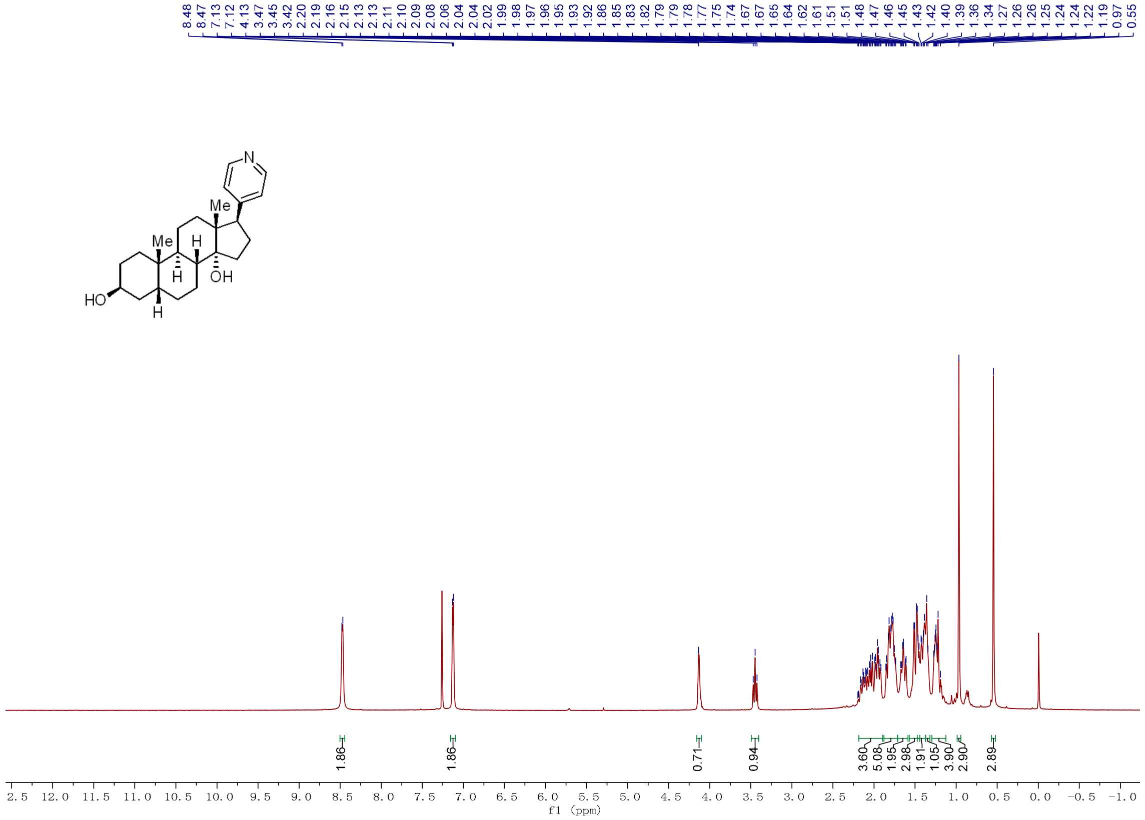

^{13}C NMR Spectrum of 2-2a (101 MHz, $CDCl_3$)

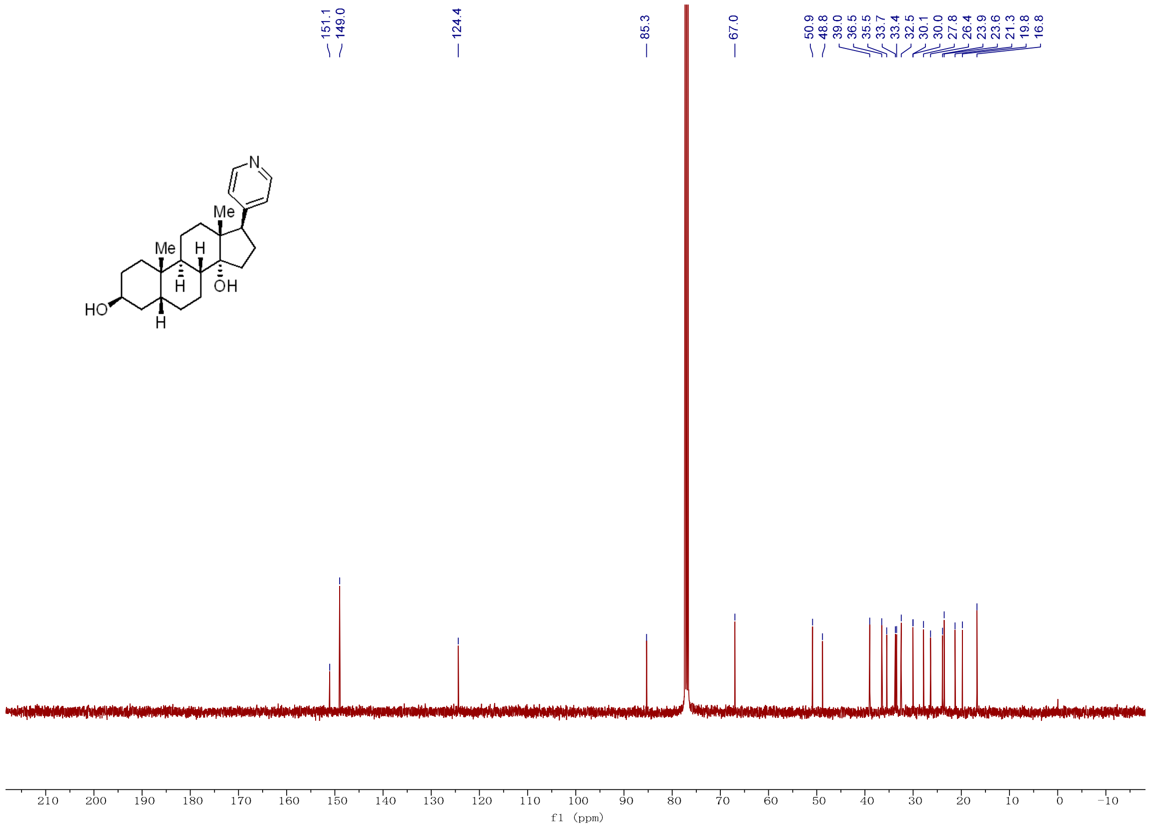

^{1}H NMR Spectrum of 2-2b (400 MHz, $CDCl_3$)

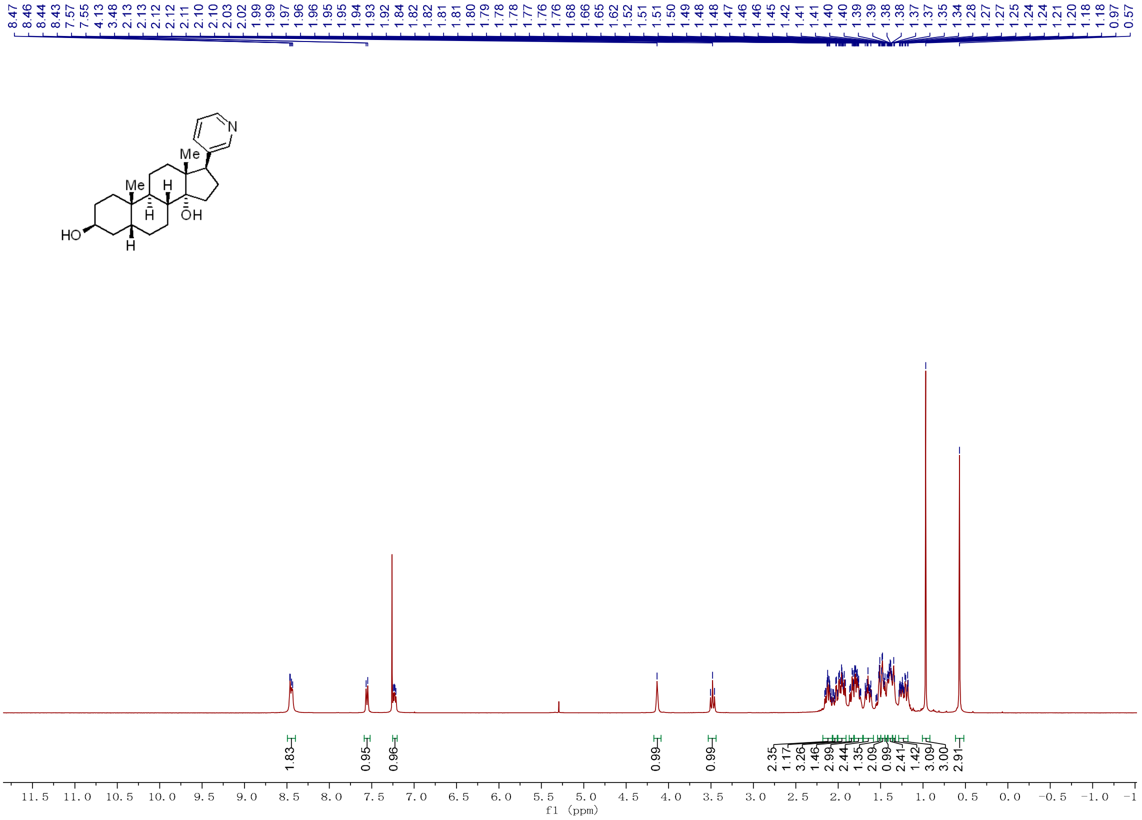

^{13}C NMR Spectrum of 2-2b (101 MHz, $CDCl_3$)

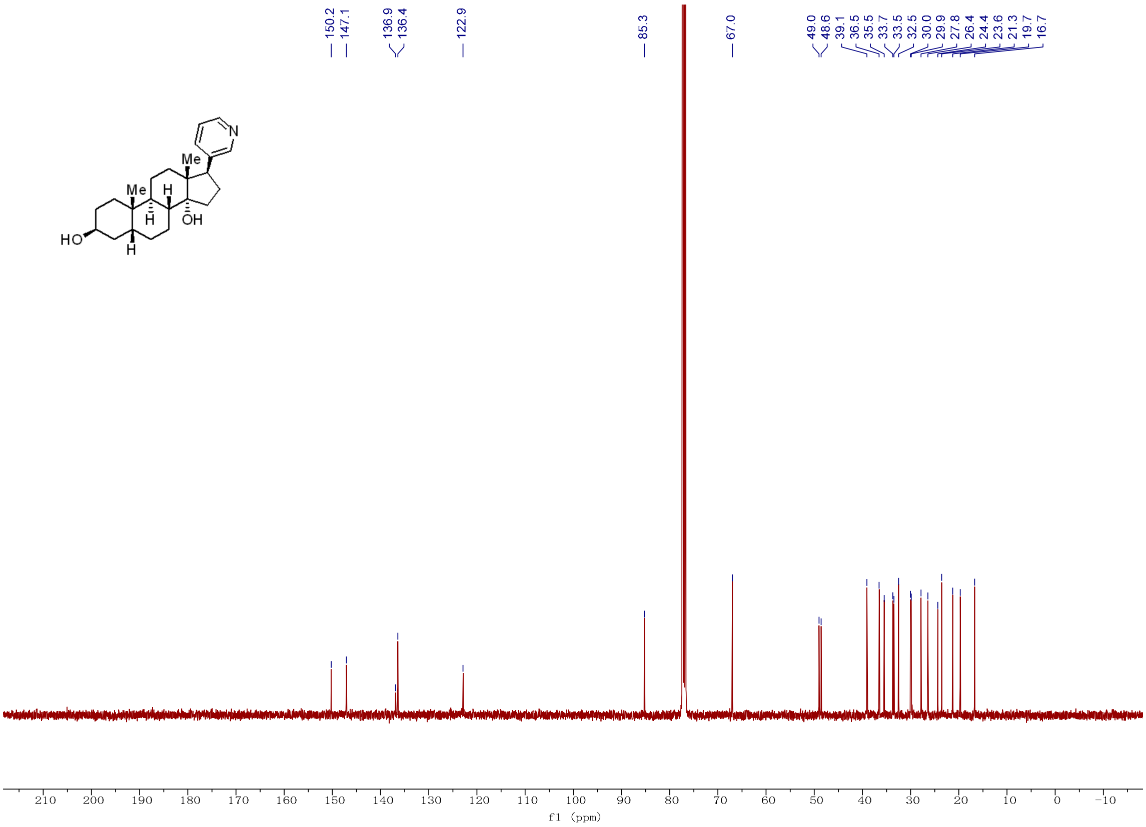

^{1}H NMR Spectrum of 2-2c (400 MHz, $CDCl_3$)

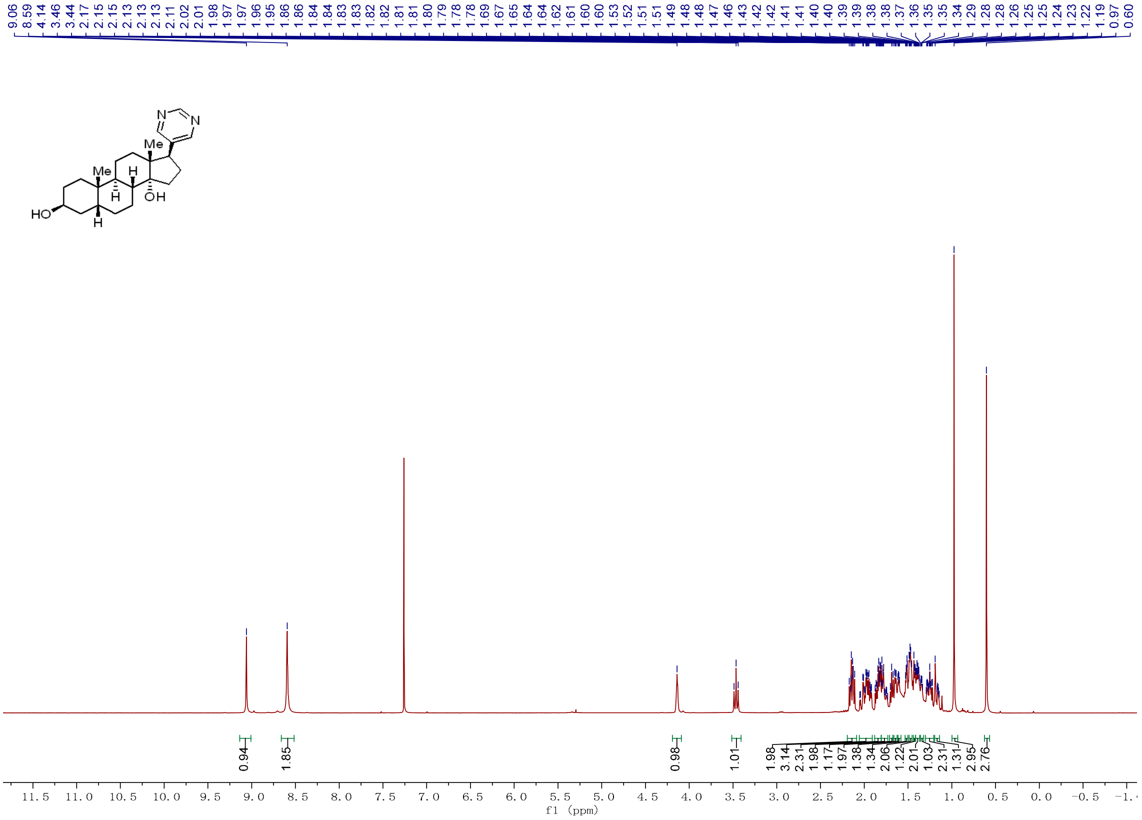

^{13}C NMR Spectrum of 2-2c (101 MHz, $CDCl_3$)

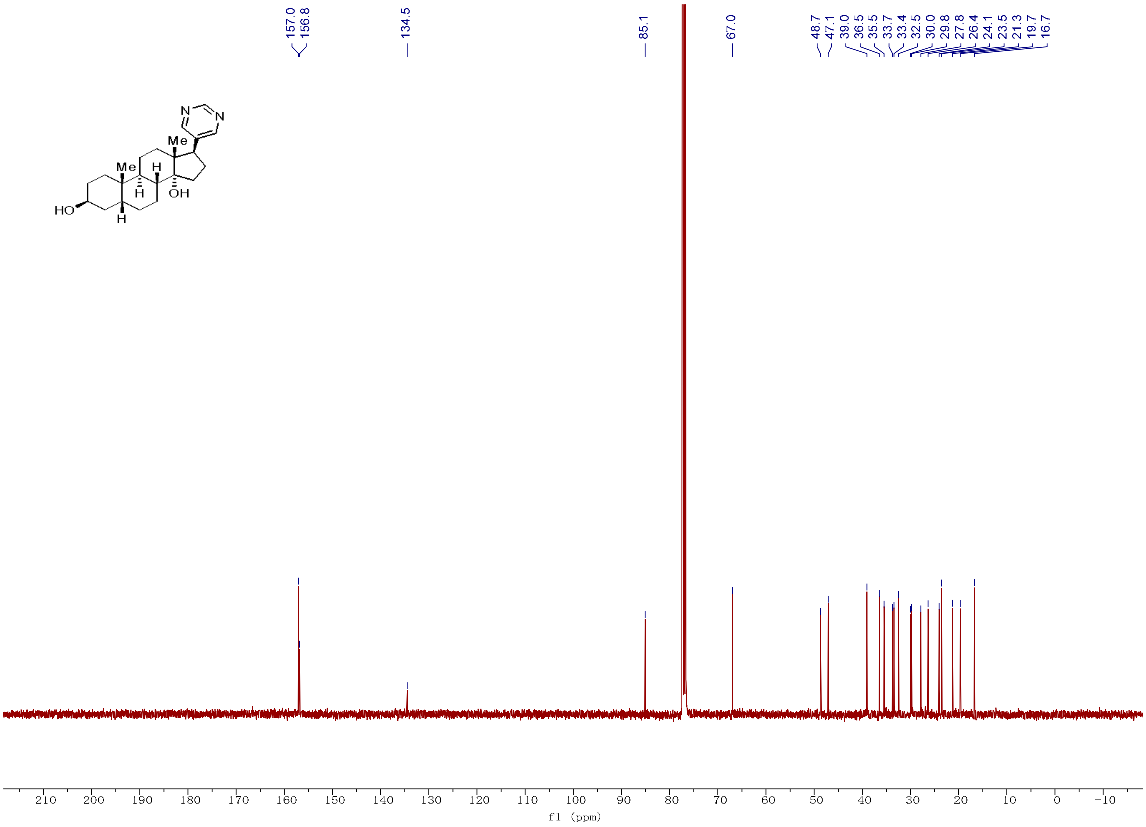

^1H NMR Spectrum of 2-2d (400 MHz, $CDCl_3$)

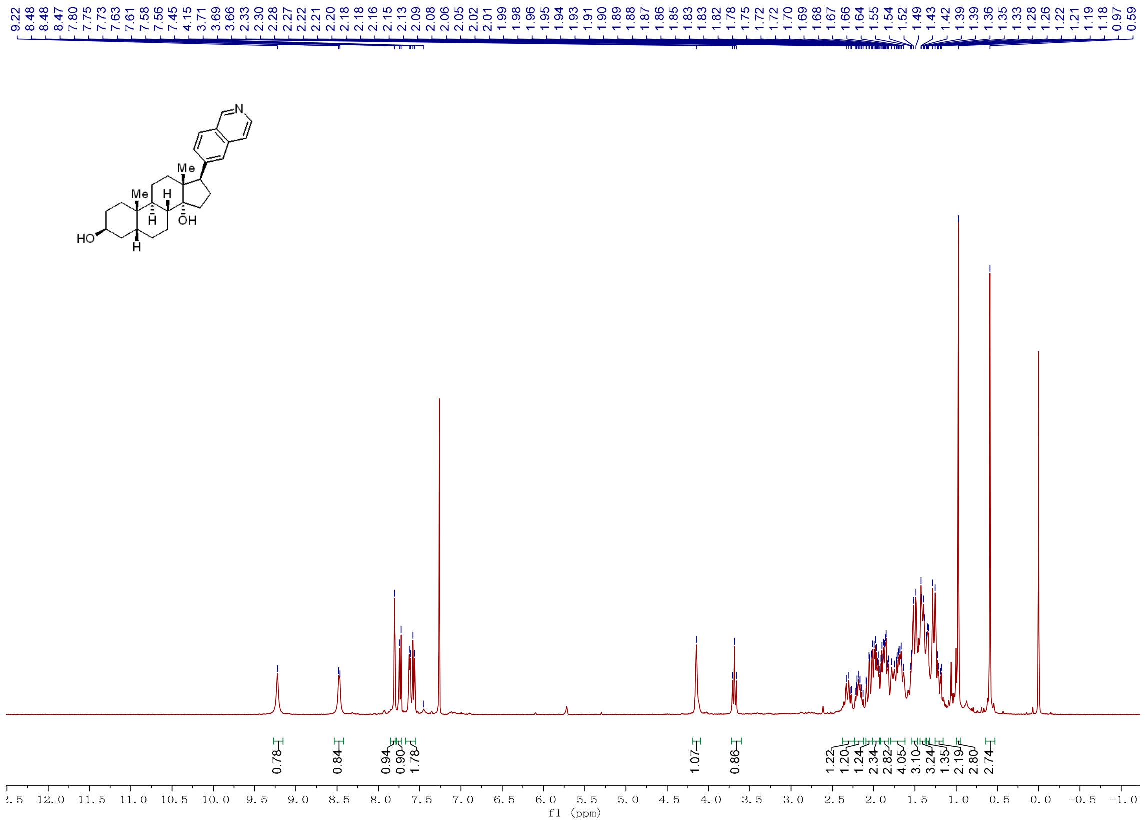

^{13}C NMR Spectrum of 2-2d (101 MHz, $CDCl_3$)

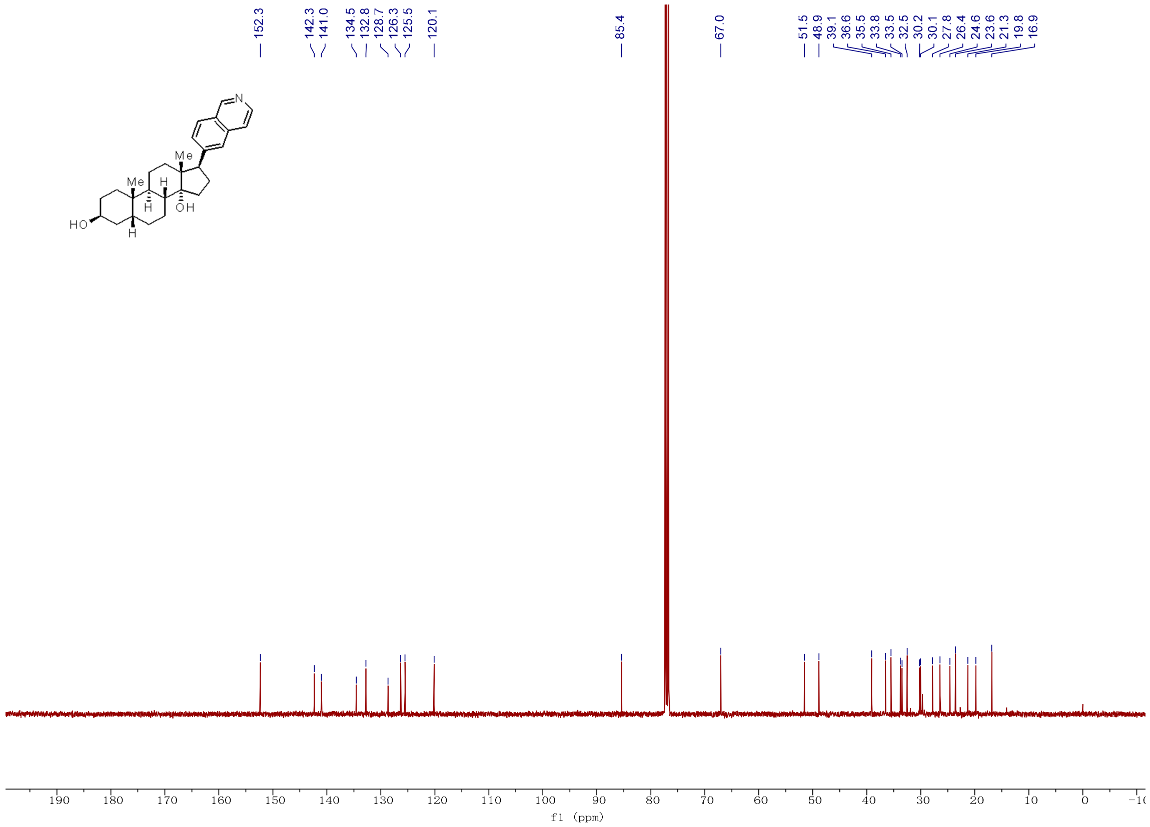

^{1}H NMR Spectrum of 2-2e (400 MHz, $CDCl_3$)

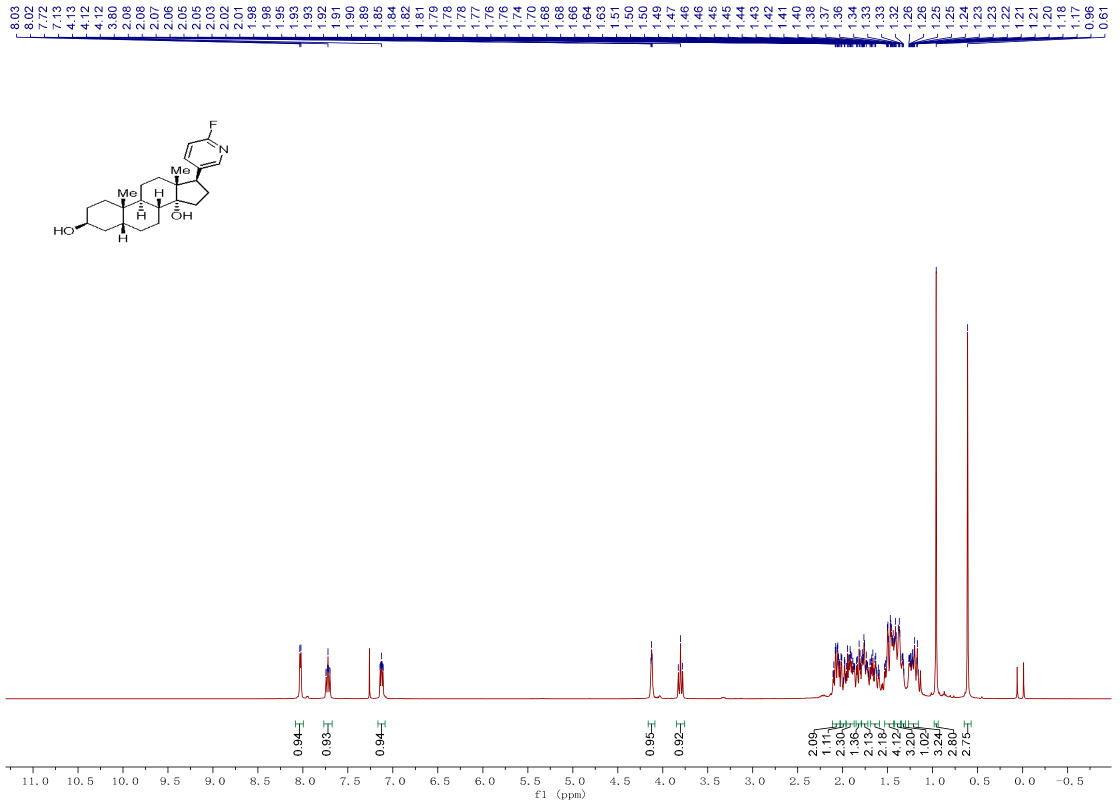

^{13}C NMR Spectrum of 2-2e (101 MHz, $CDCl_3$)

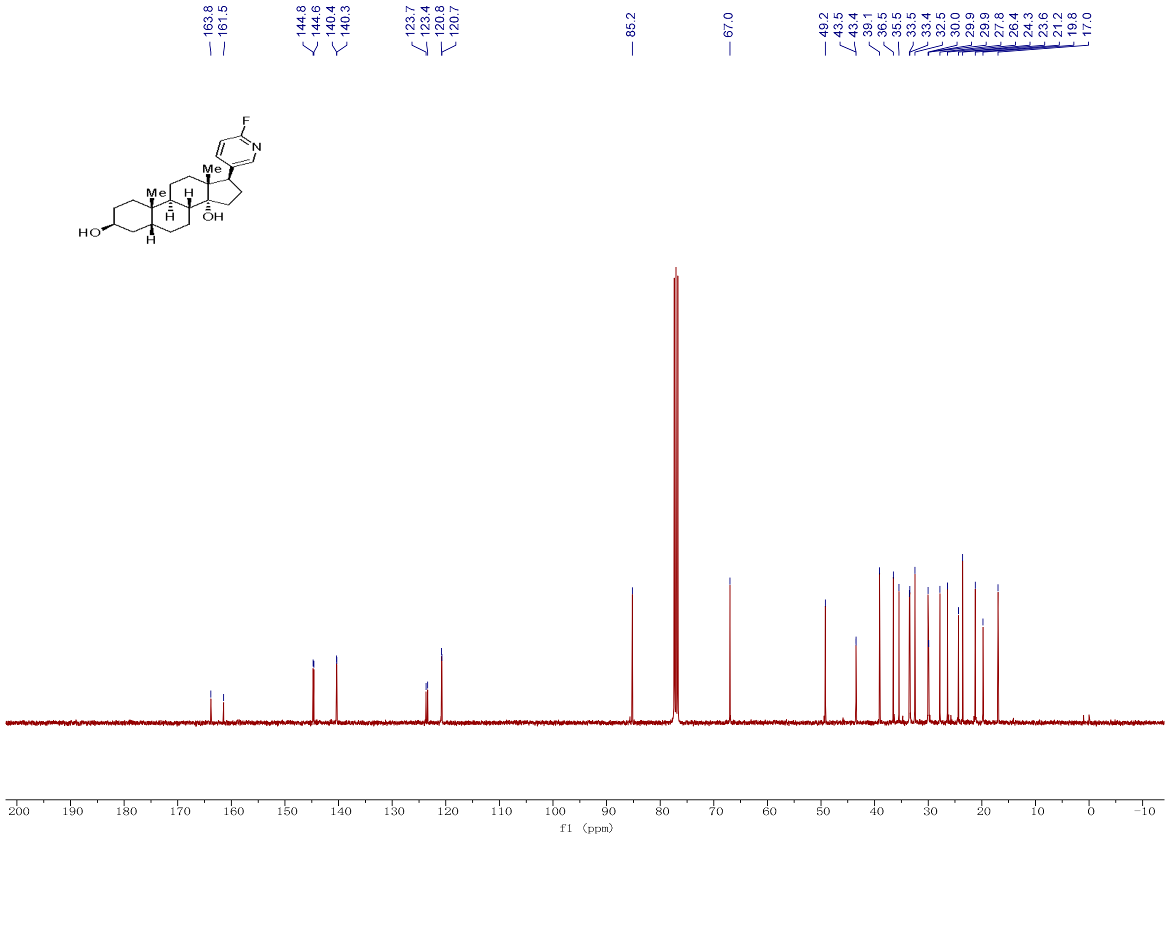

^{1}H NMR Spectrum of 2-3a (400 MHz, $CDCl_3$)

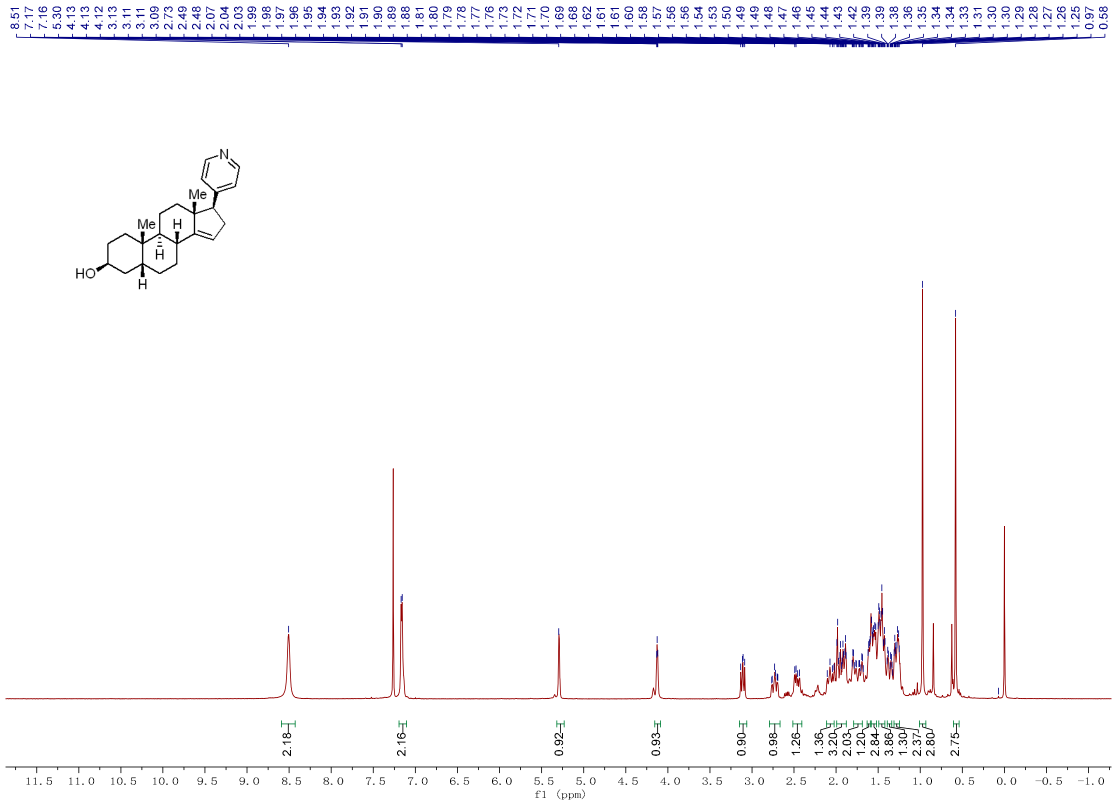

^{13}C NMR Spectrum of 2-3a (101 MHz, $CDCl_3$)

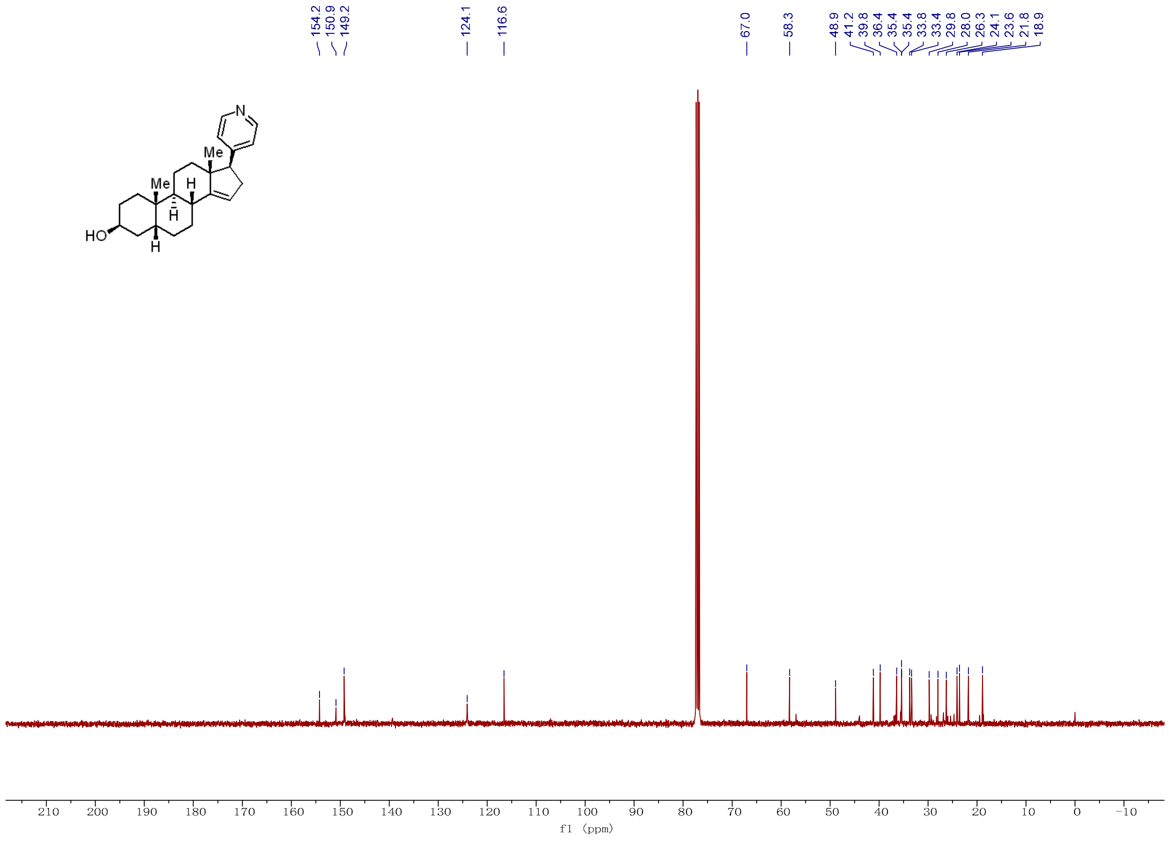

^{1}H NMR Spectrum of 2-3b (400 MHz, $CDCl_3$)

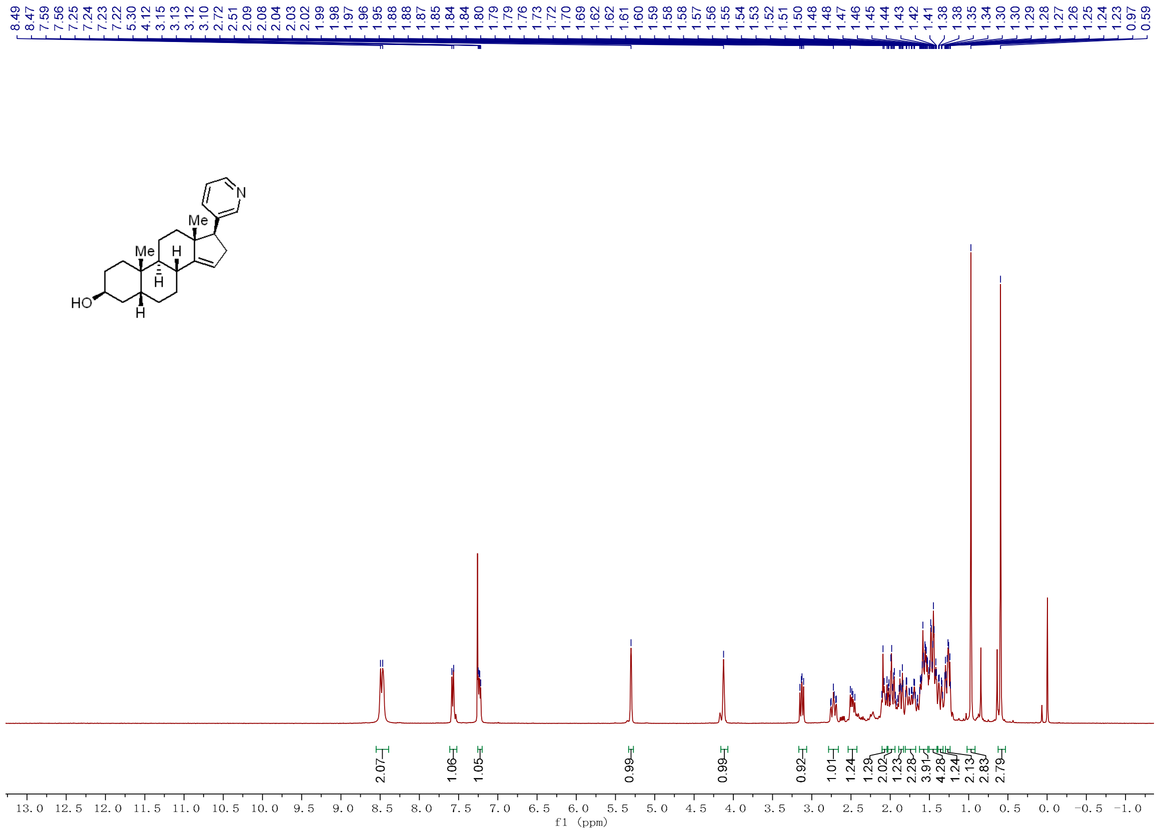

^{13}C NMR Spectrum of 2-3b (101 MHz, $CDCl_3$)

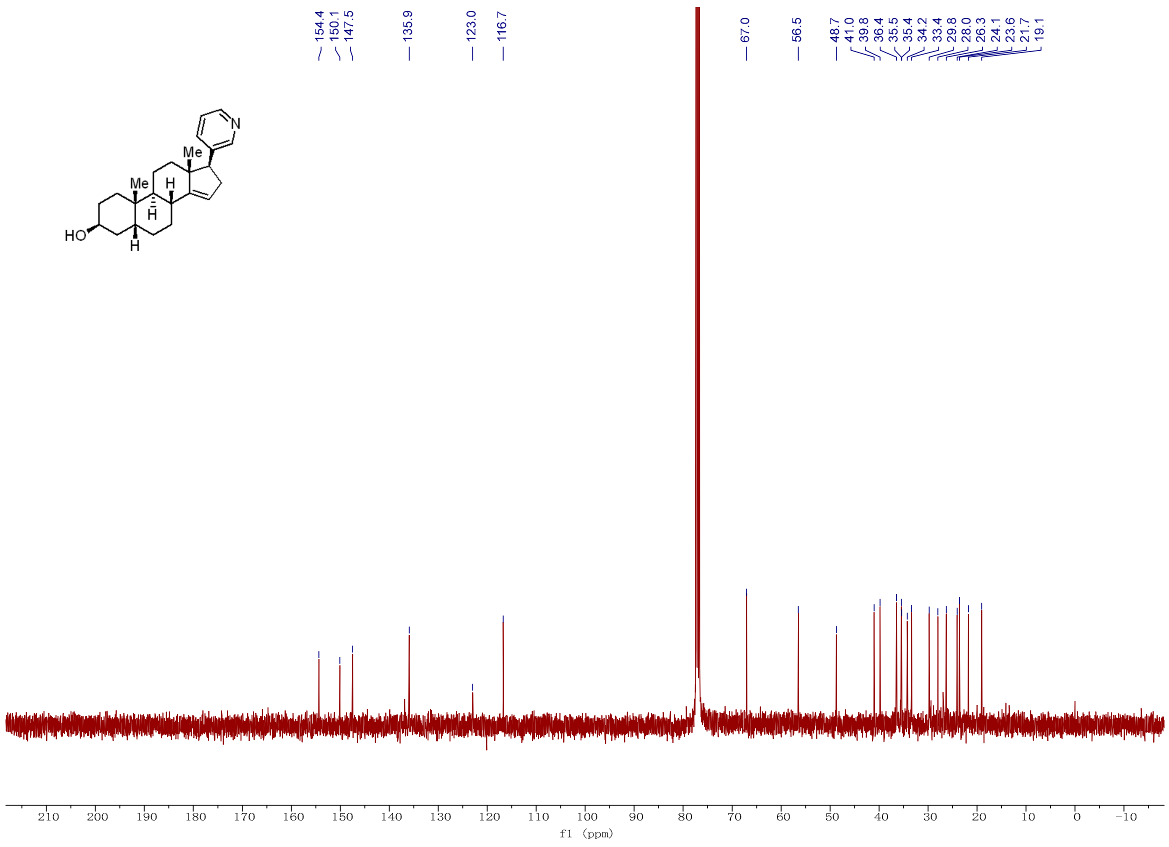

Compound **2-3c**

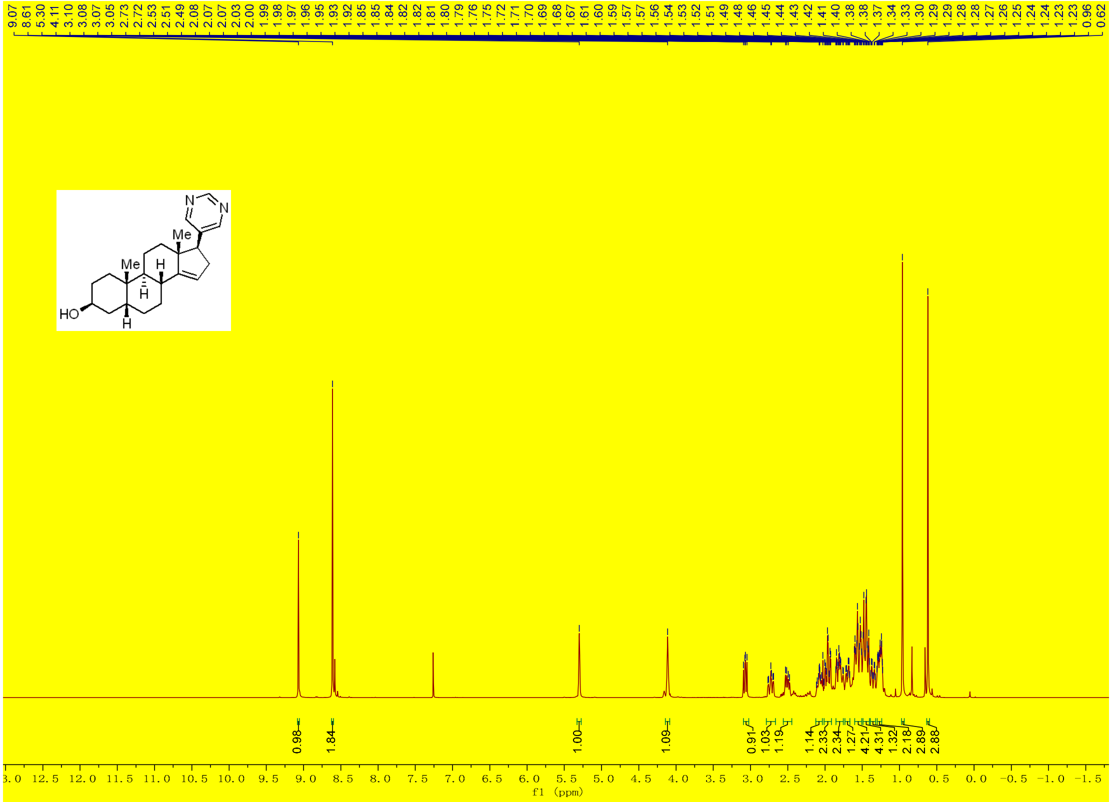

^{1}H NMR Spectrum of 2-3d (400 MHz, $CDCl_3$)

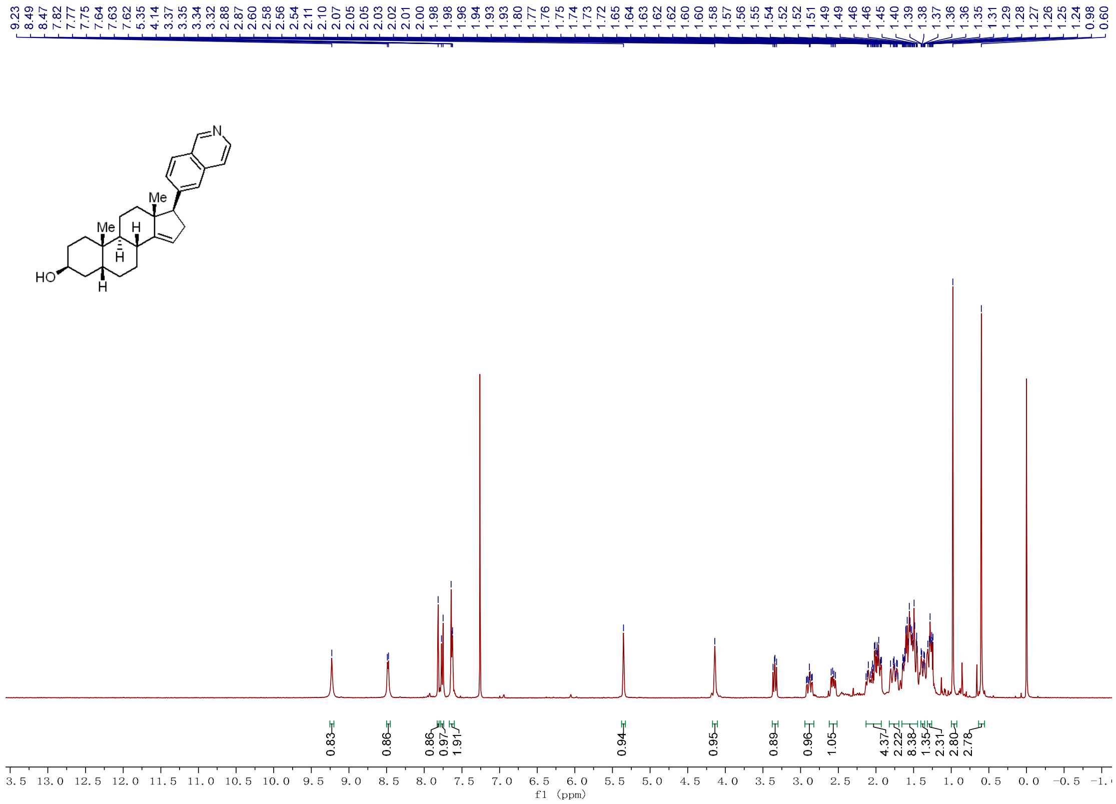

^{13}C NMR Spectrum of 2-3d (101 MHz, $CDCl_3$)

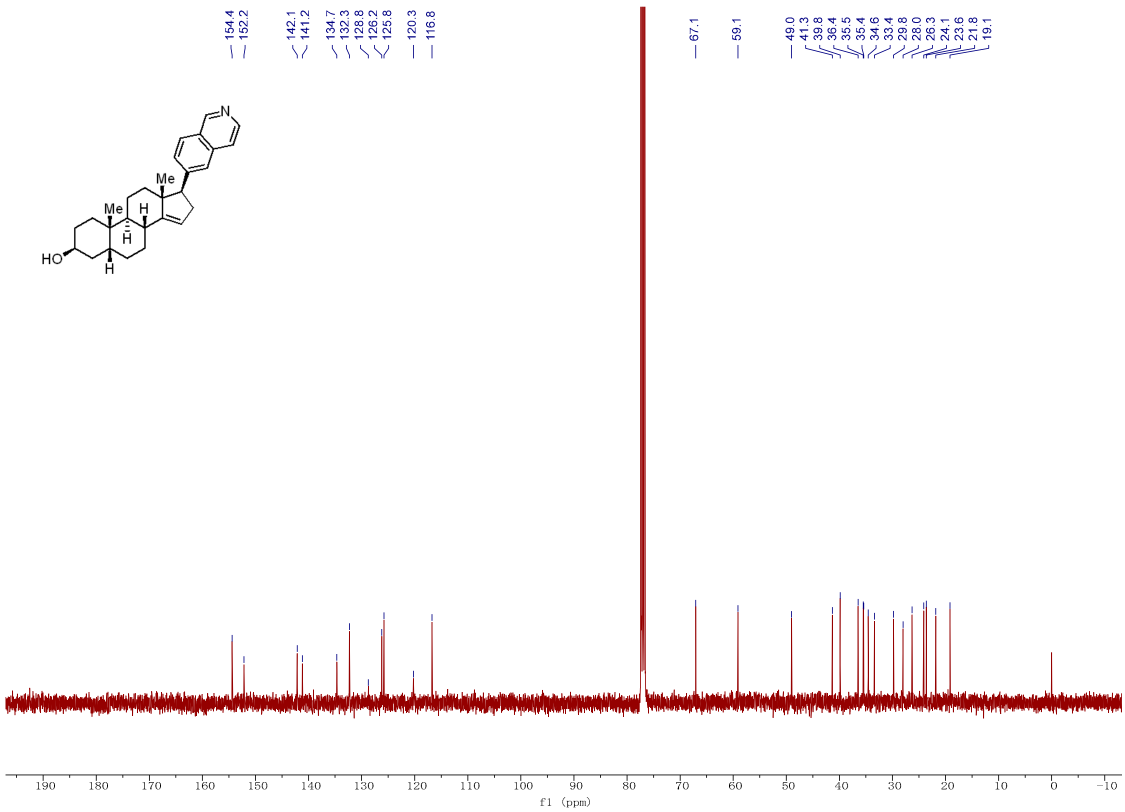

^{1}H NMR Spectrum of 2-3e (400 MHz, $CDCl_3$)

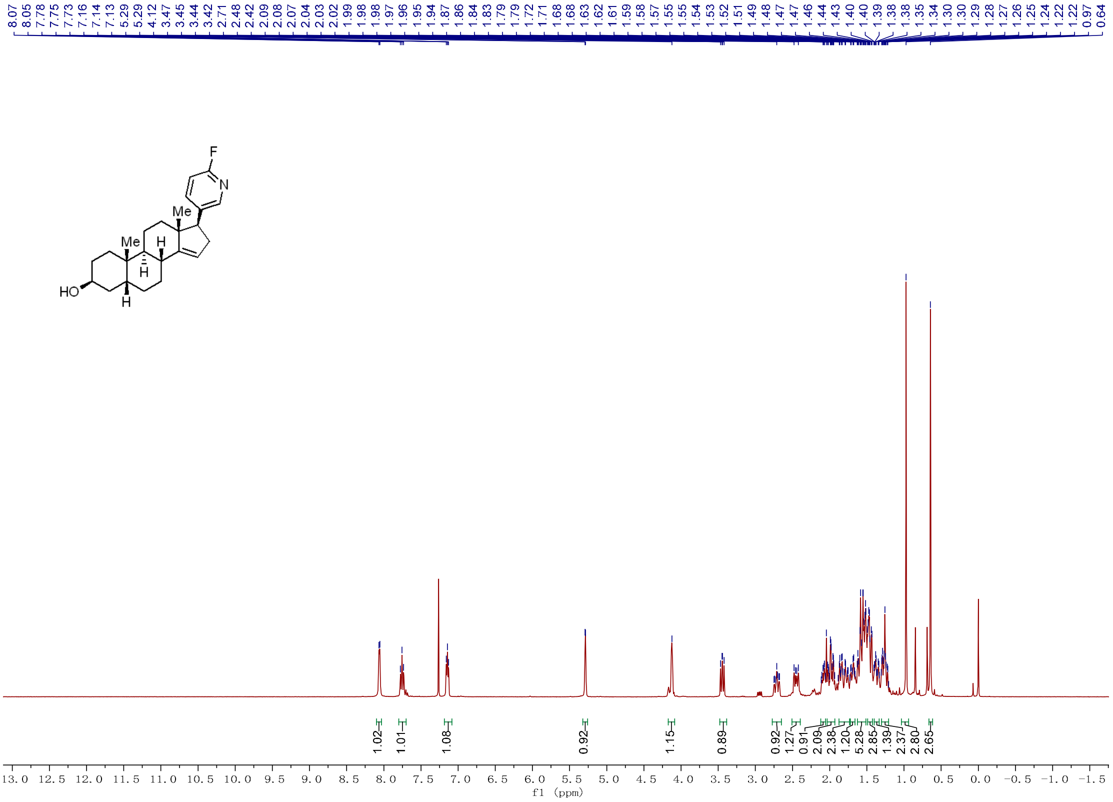

^{13}C NMR Spectrum of 2-3e (101 MHz, $CDCl_3$)

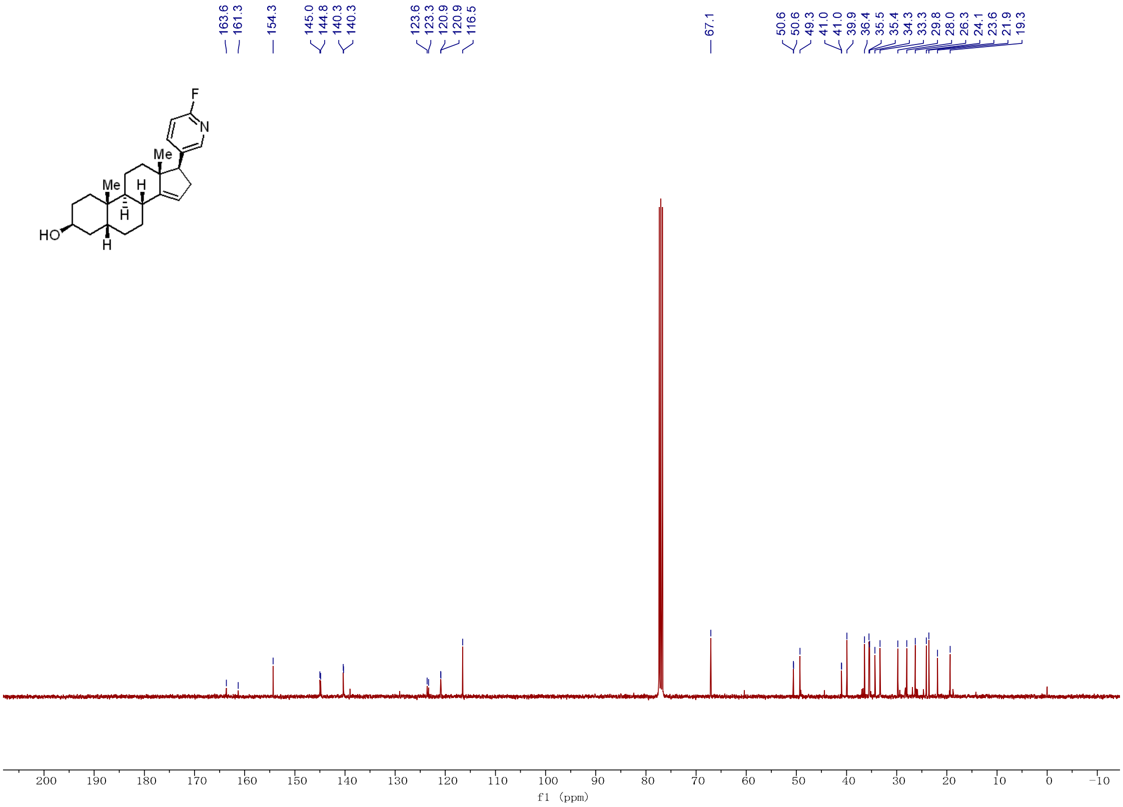

^{1}H NMR Spectrum of 2-3f (400 MHz, $CDCl_3$)

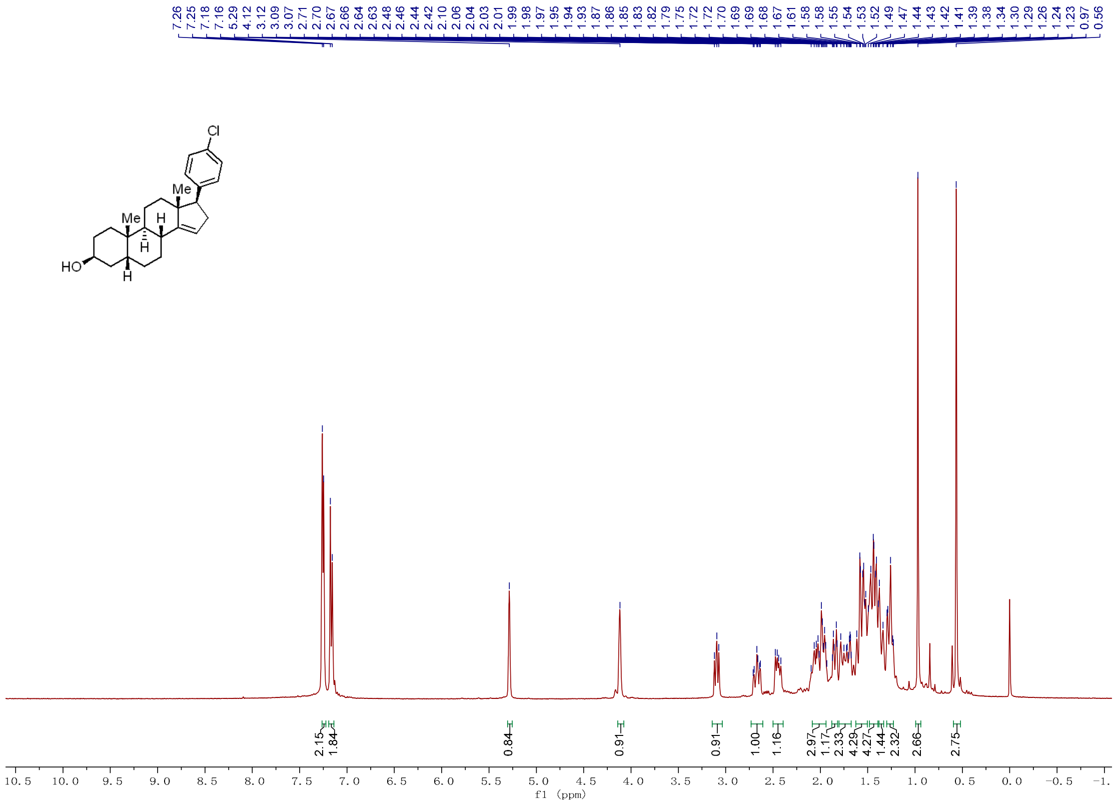

^{13}C NMR Spectrum of 2-3f (101 MHz, $CDCl_3$)

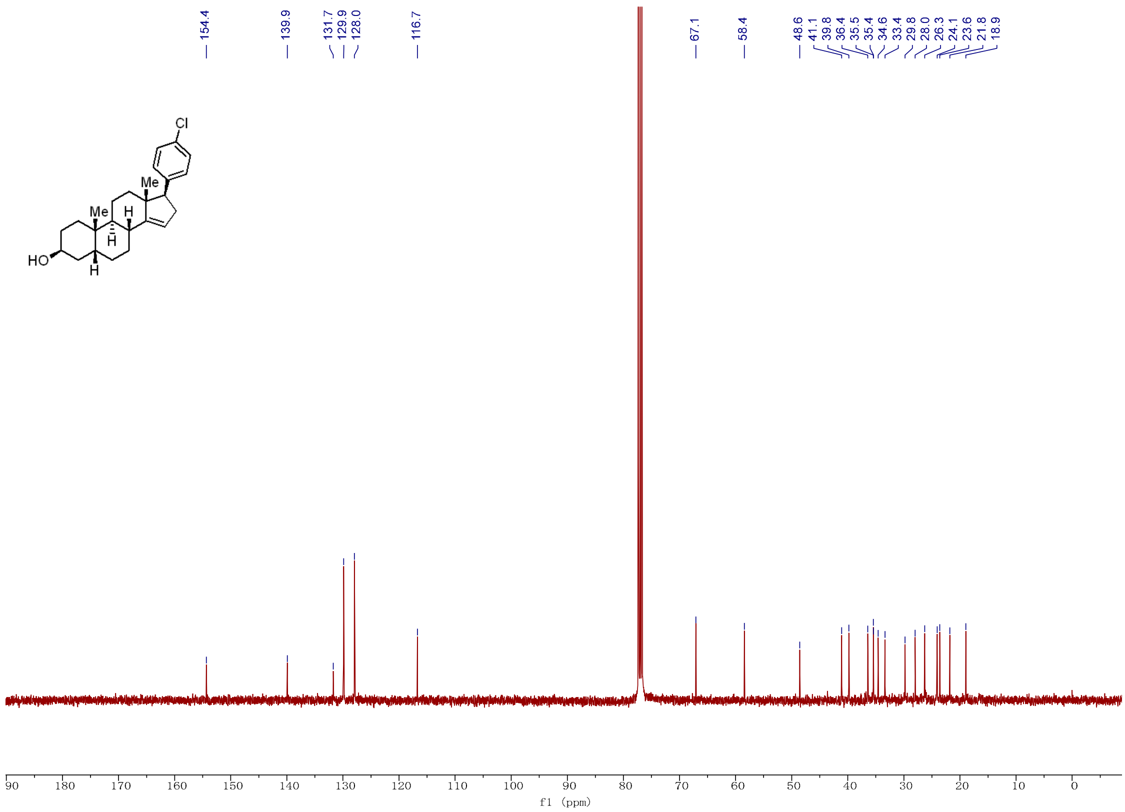

^{1}H NMR Spectrum of 2-3g (400 MHz, $CDCl_3$)

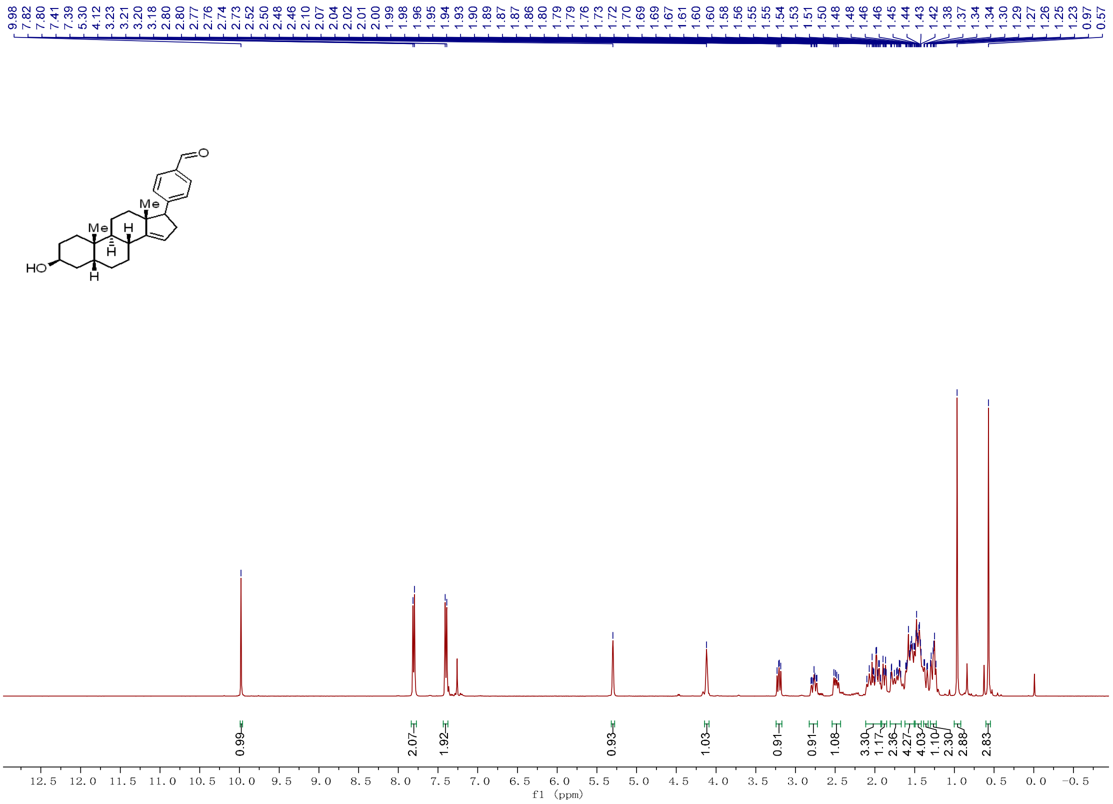

^{13}C NMR Spectrum of 2-3g (101 MHz, $CDCl_3$)

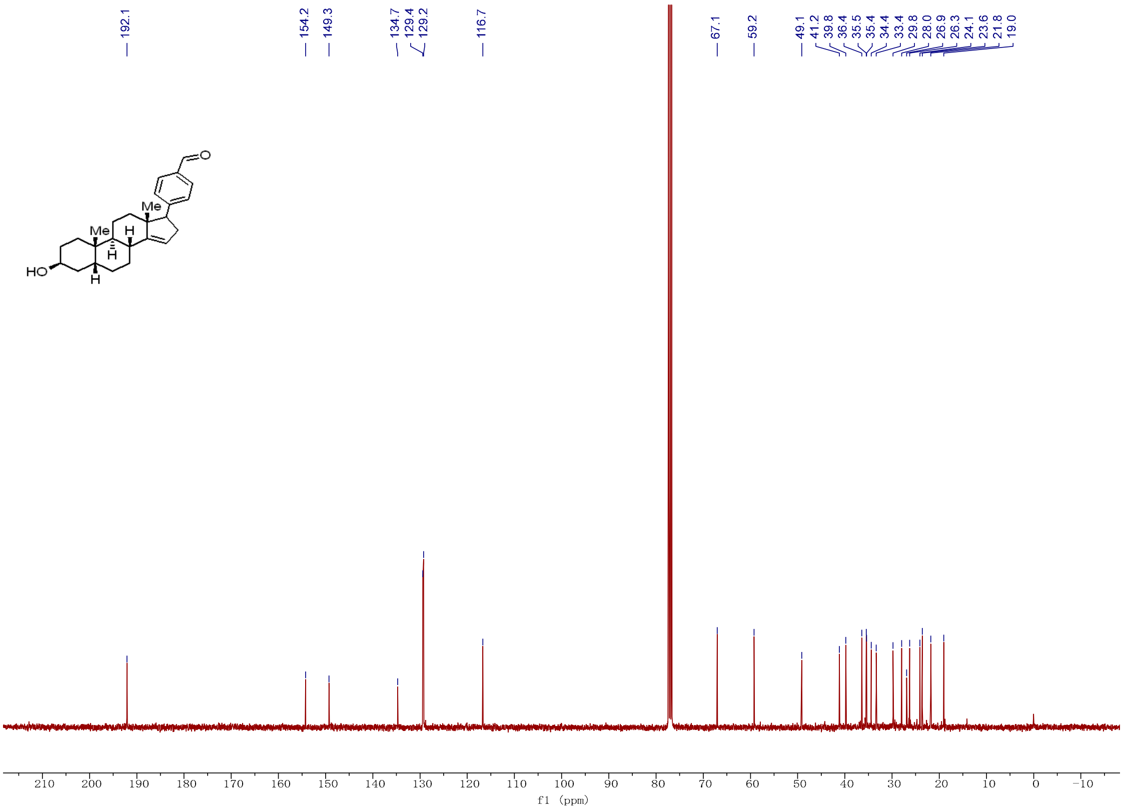

^{1}H NMR Spectrum of 2-3h (400 MHz, CDCl$_3$)

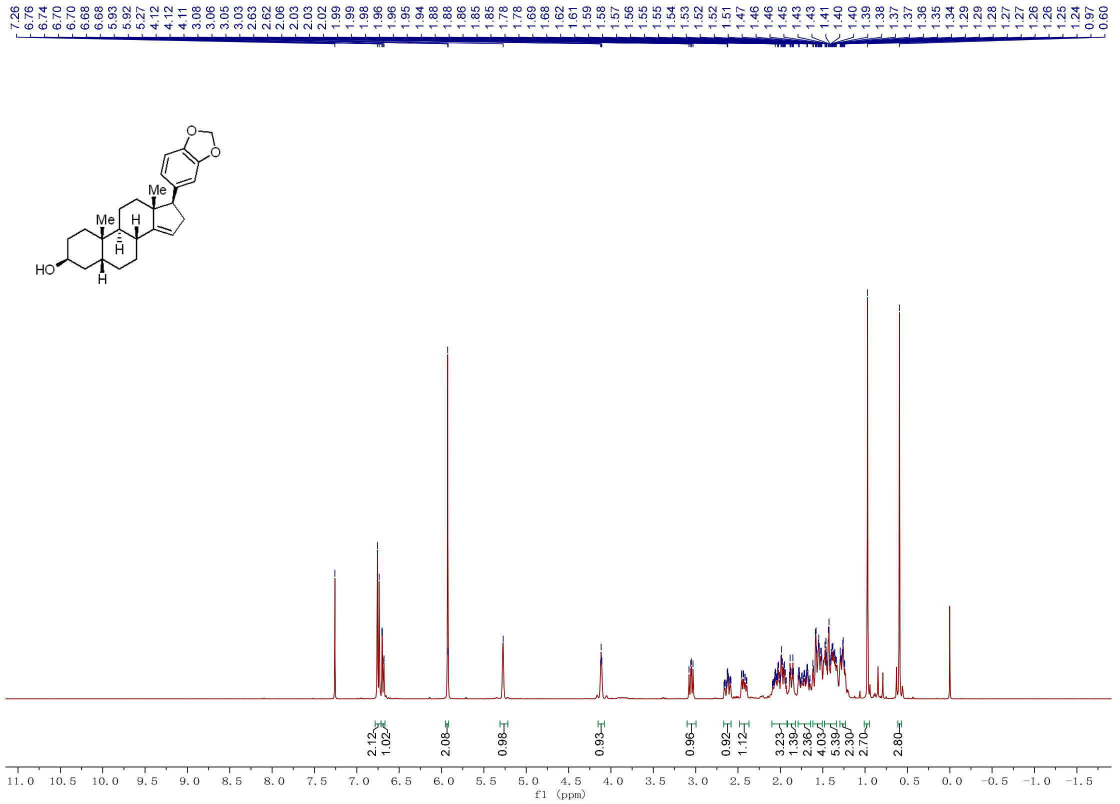

^{13}C NMR Spectrum of 2-3h (101 MHz, CDCl$_3$)

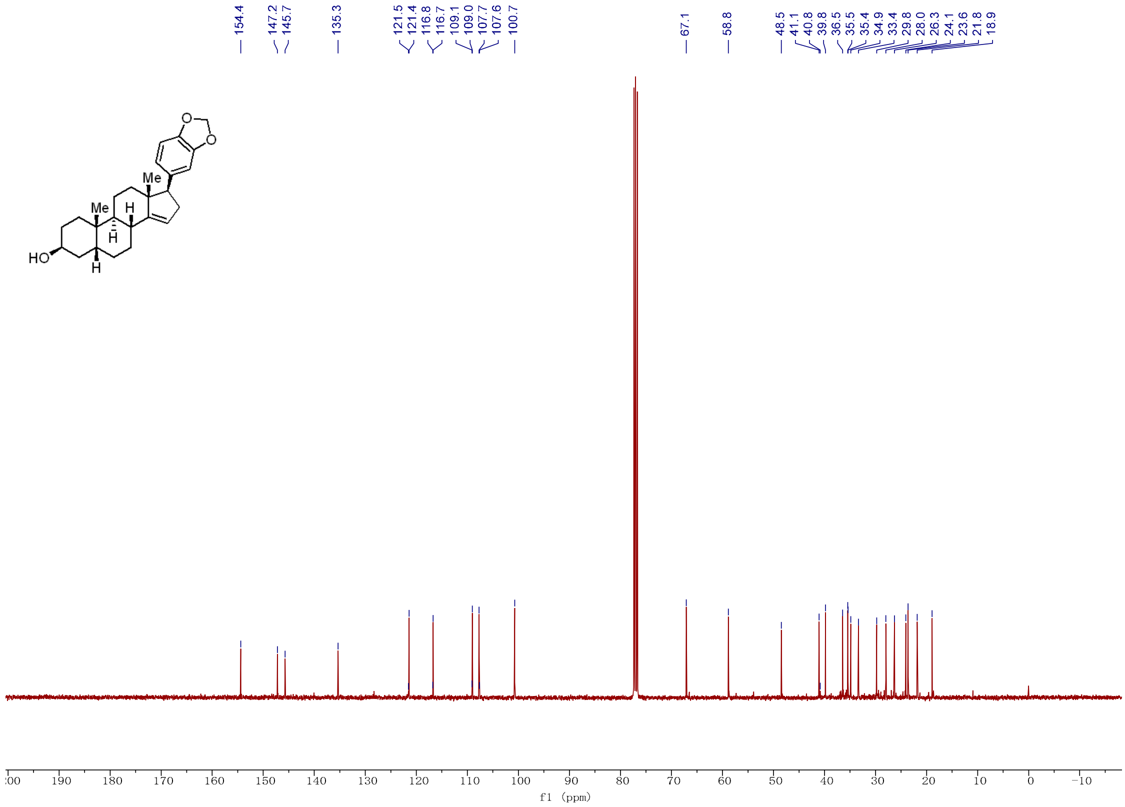

HMQC NMR Spectrum of 2-3h (101 MHz, CDCl₃)

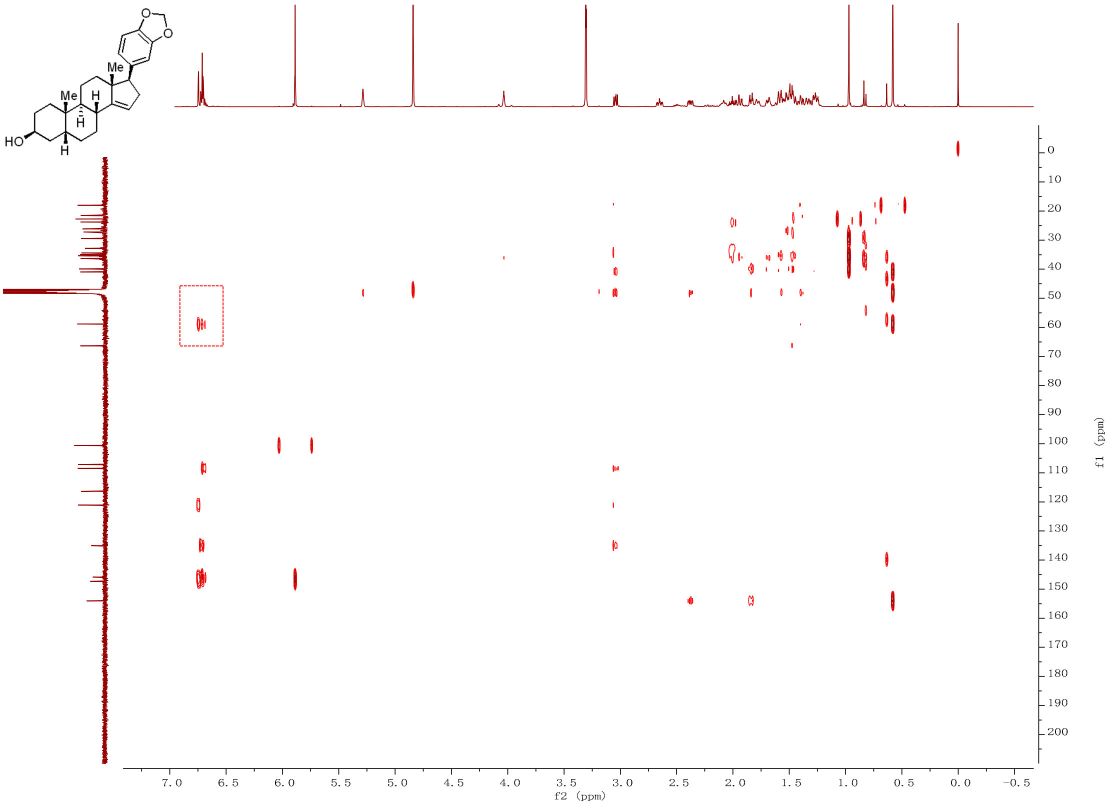

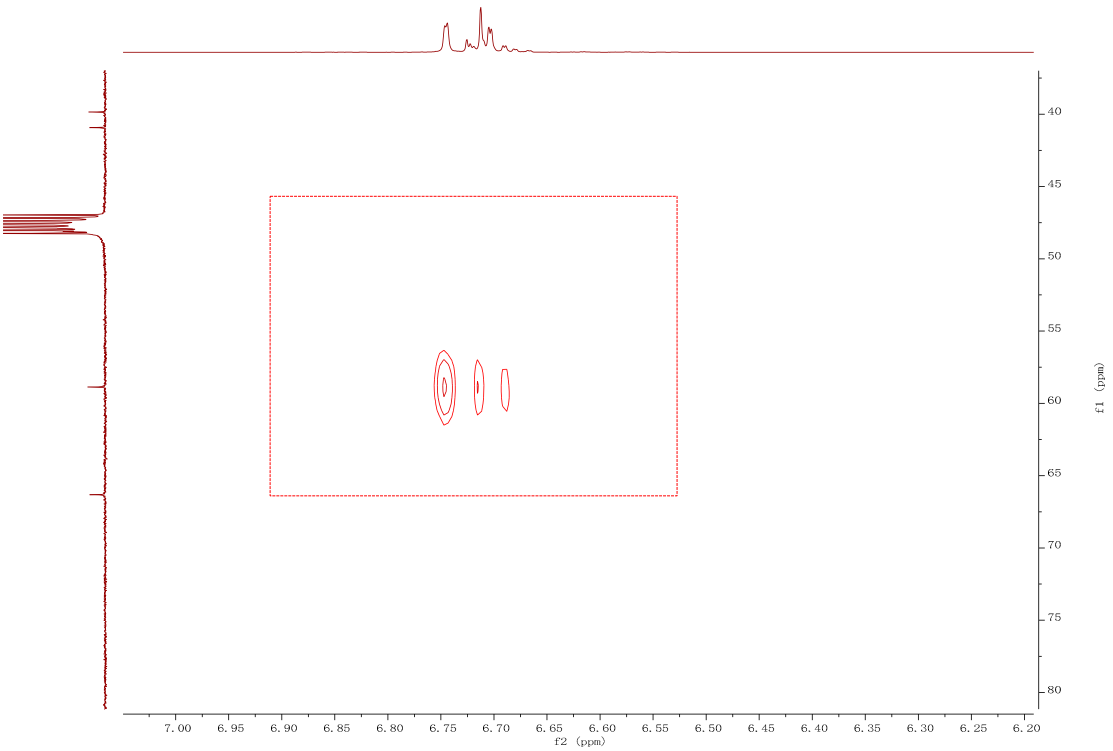

^{1}H NMR Spectrum of 2-3i (400 MHz, $CDCl_3$)

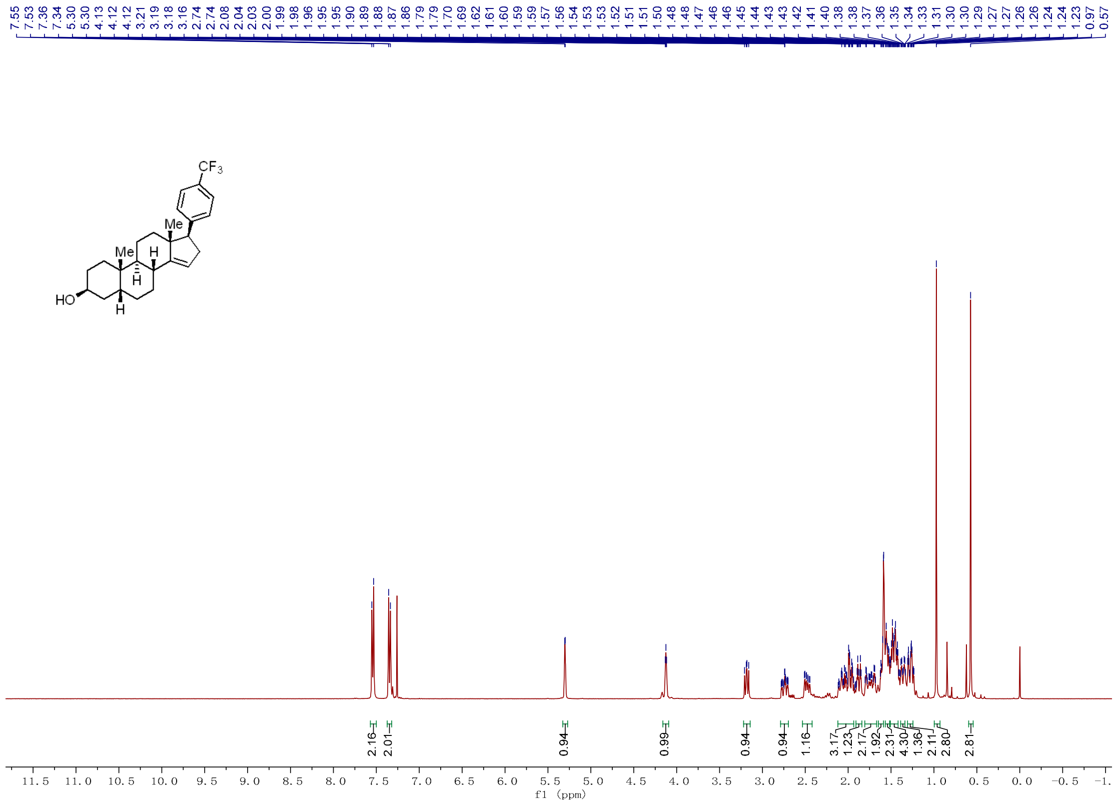

^{13}C NMR Spectrum of 2-3i (101 MHz, $CDCl_3$)

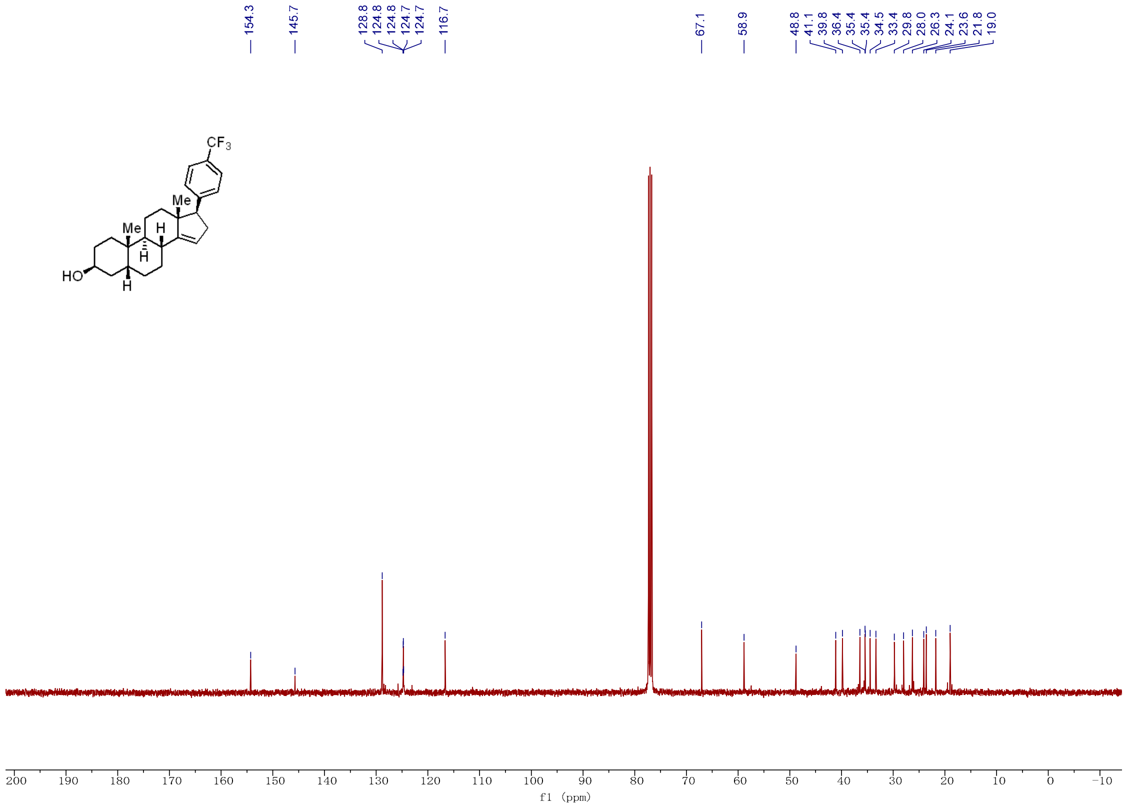

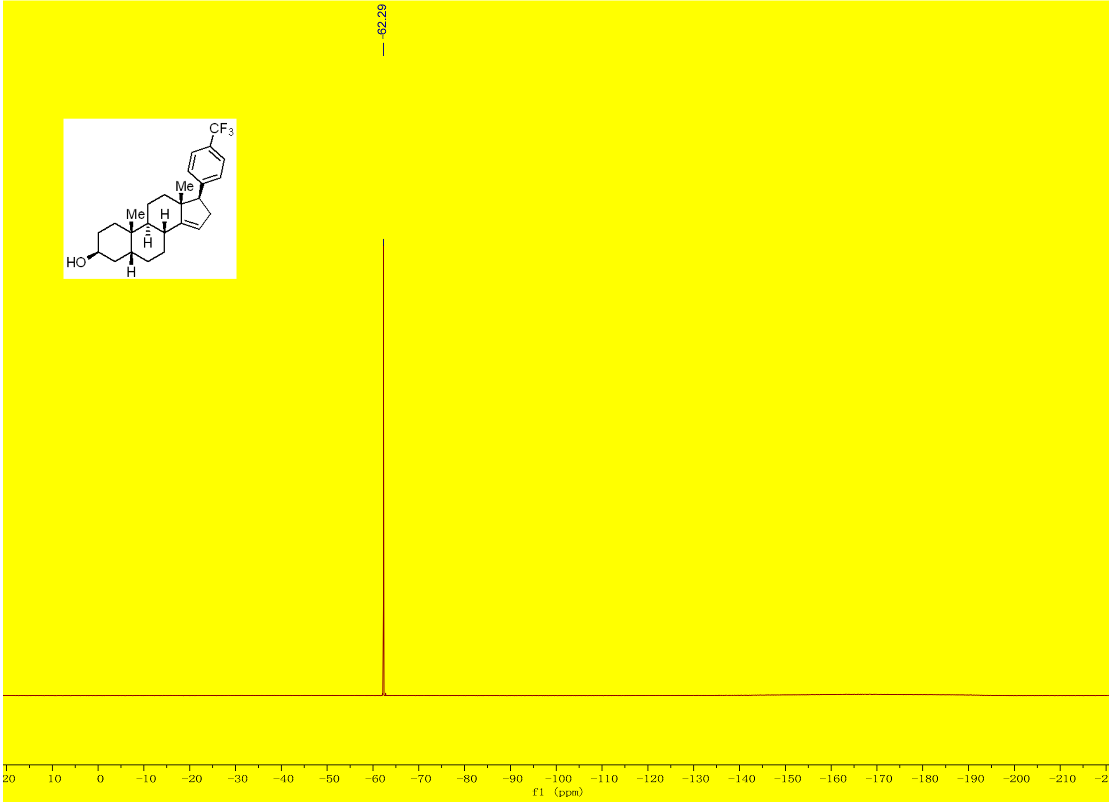

-62.29
CF3
Me
Me
H
H
H
HO
20 10 0 −10 −20 −30 −40 −50 −60 −70 −80 −90 −100 −110 −120 −130 −140 −150 −160 −170 −180 −190 −200 −210
f1 (ppm)

^{1}H NMR Spectrum of 2-3j (400 MHz, CDCl$_3$)

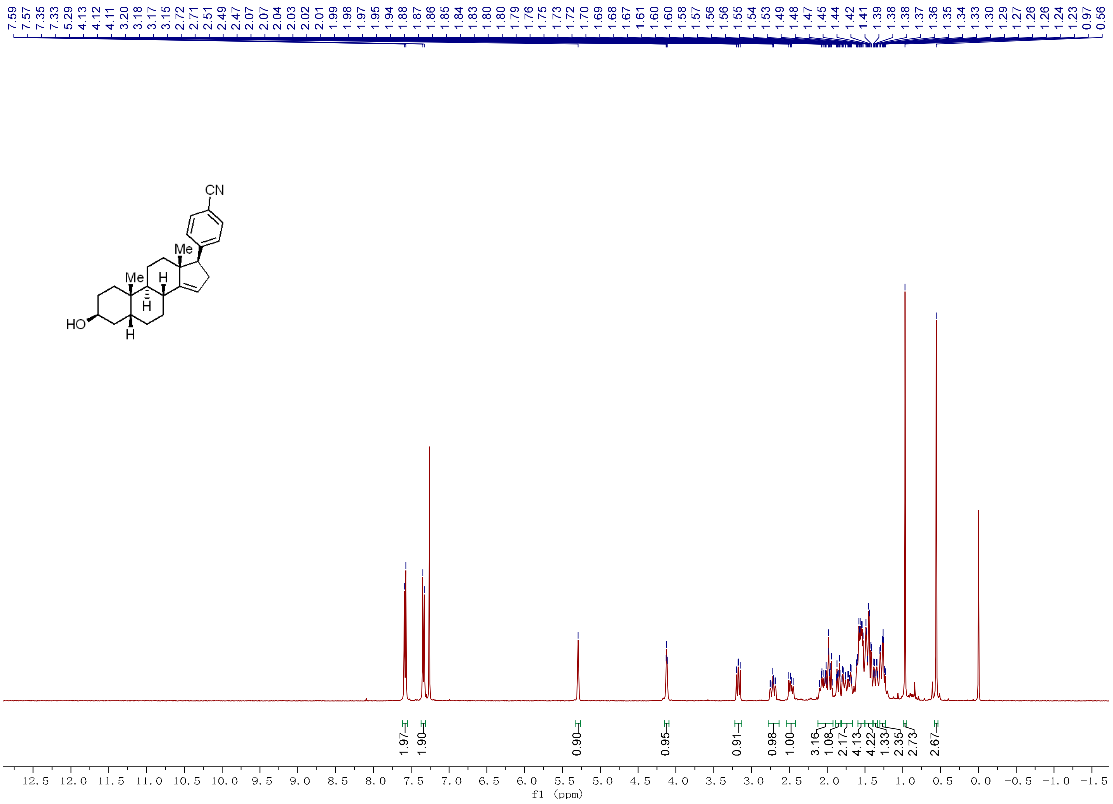

^{13}C NMR Spectrum of 2-3j (101 MHz, CDCl$_3$)

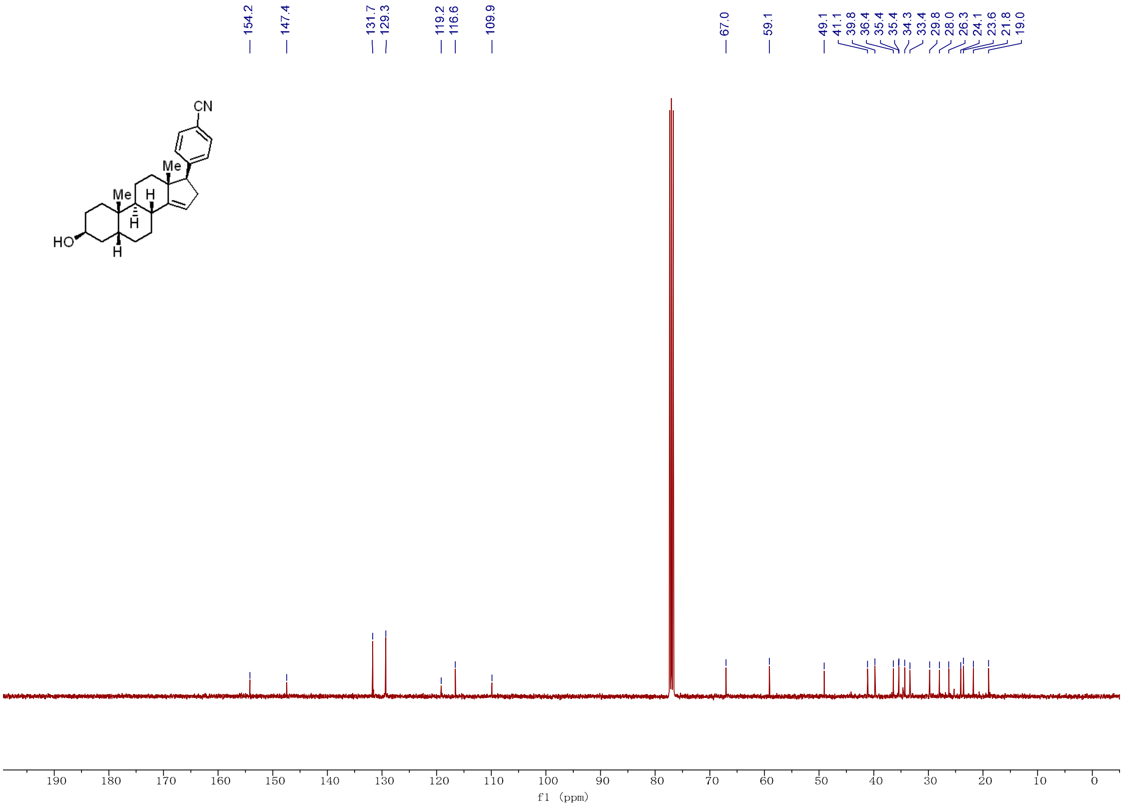

^{1}H NMR Spectrum of 2-3k (400 MHz, CDCl$_3$)

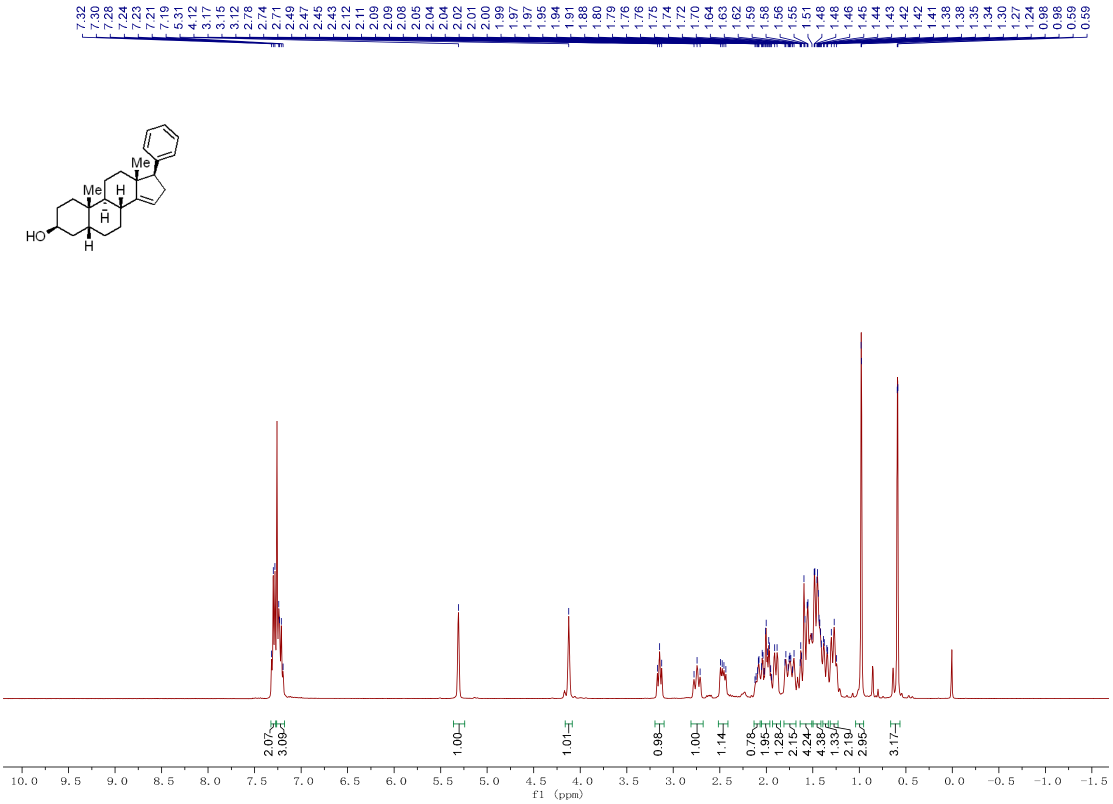

^{13}C NMR Spectrum of 2-3k (101 MHz, CDCl$_3$)

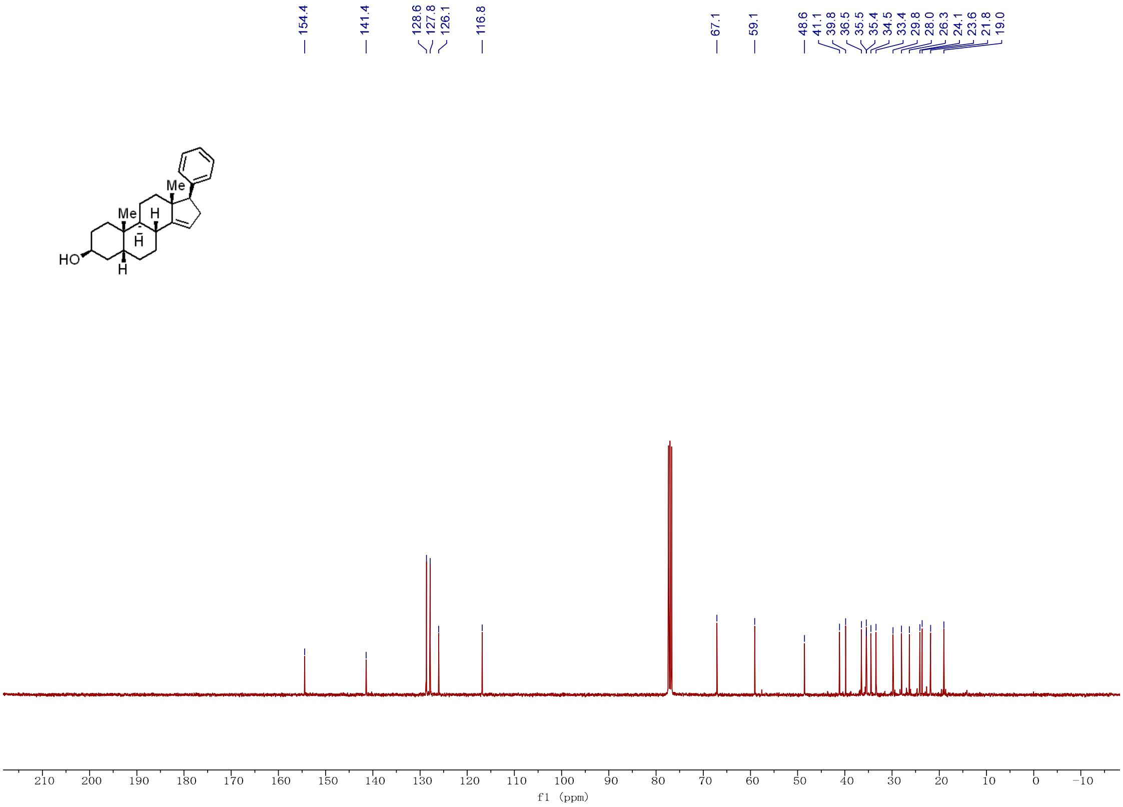

^{1}H NMR Spectrum of 2-3l (400 MHz, CDCl$_3$)

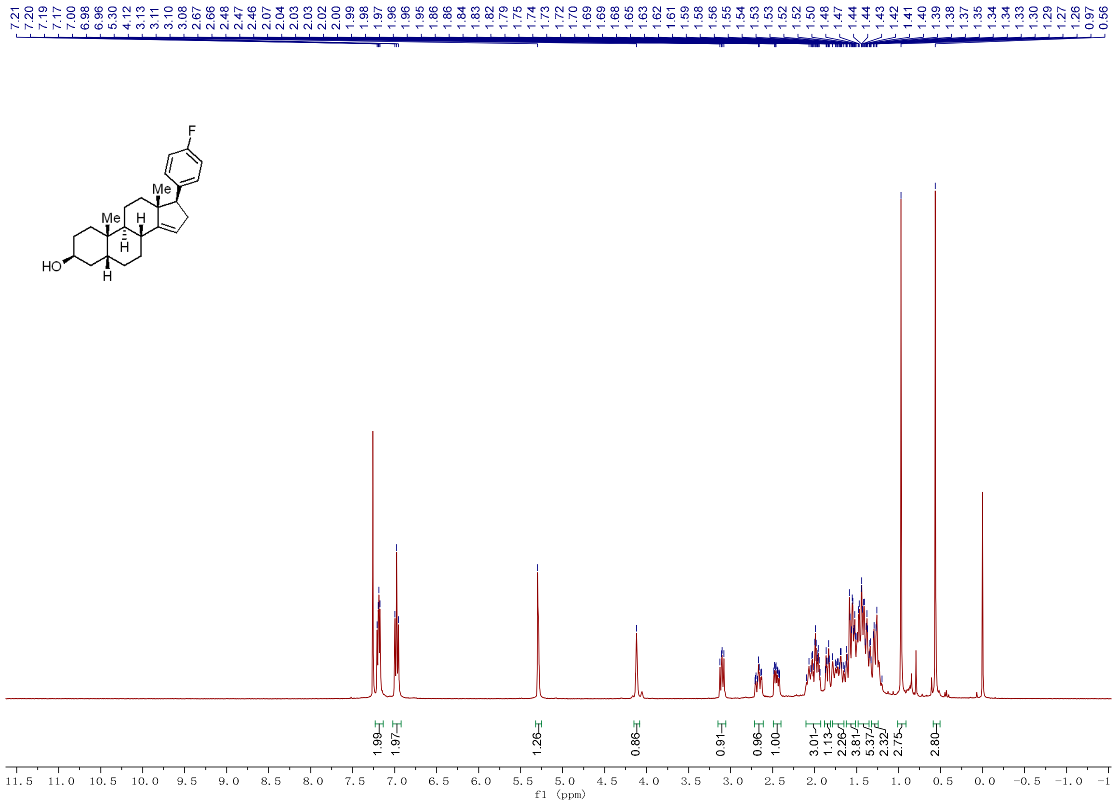

^{13}C NMR Spectrum of 2-3l (101 MHz, CDCl$_3$)

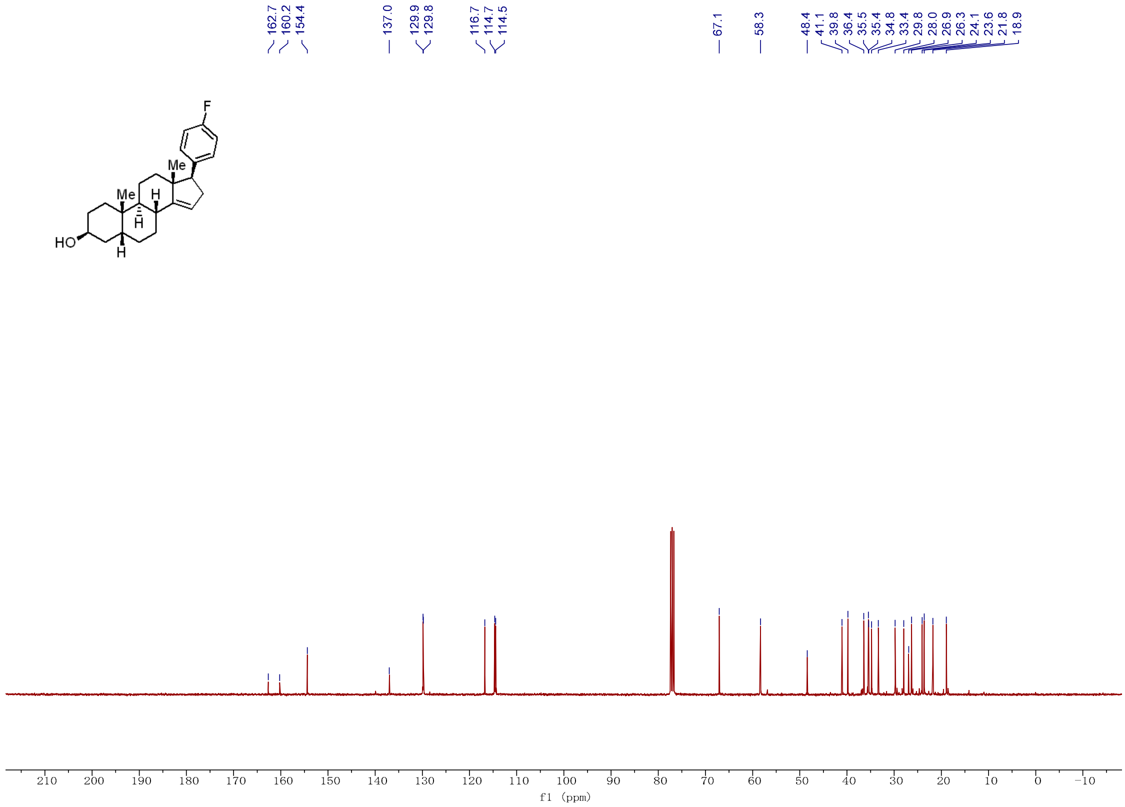

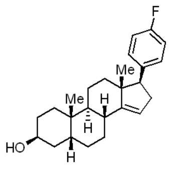
F
Me
Me
H
H
H
HO

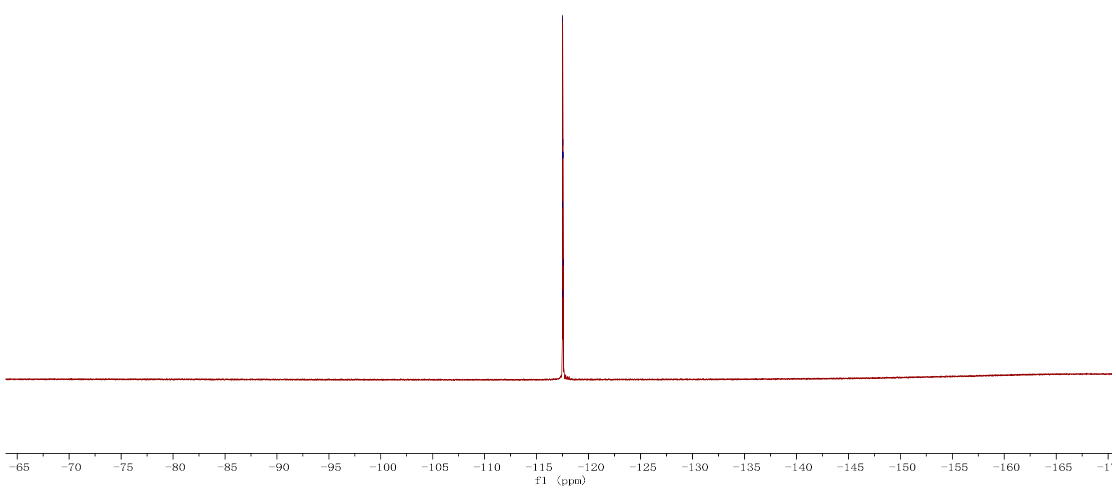
-117.48
-117.50
-117.51
-117.52
-117.53
-117.54
-117.56
−65
−70
−75
−80
−85
−90
−95
−100
−105
−110
−115
−120
−125
−130
−135
−140
−145
−150
−155
−160
−165
f1 (ppm)

^{1}H NMR Spectrum of 2-4a (400 MHz, $CDCl_3$)

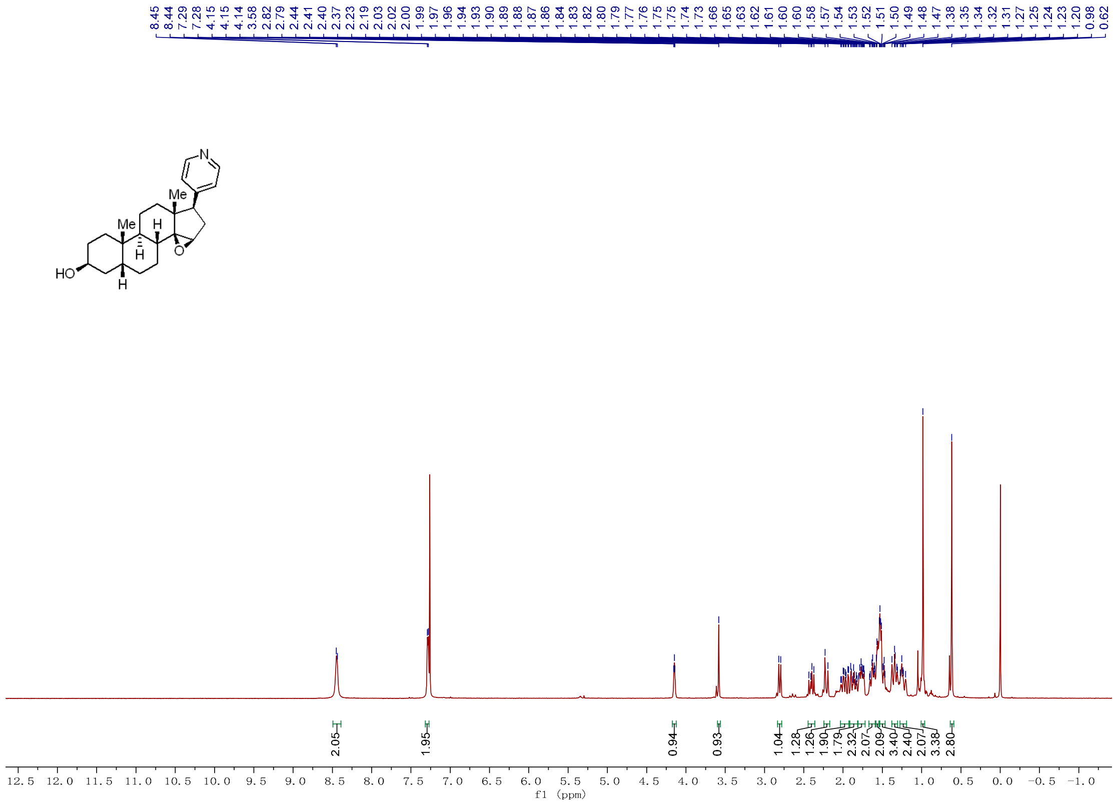

^{13}C NMR Spectrum of 2-4a (101 MHz, $CDCl_3$)

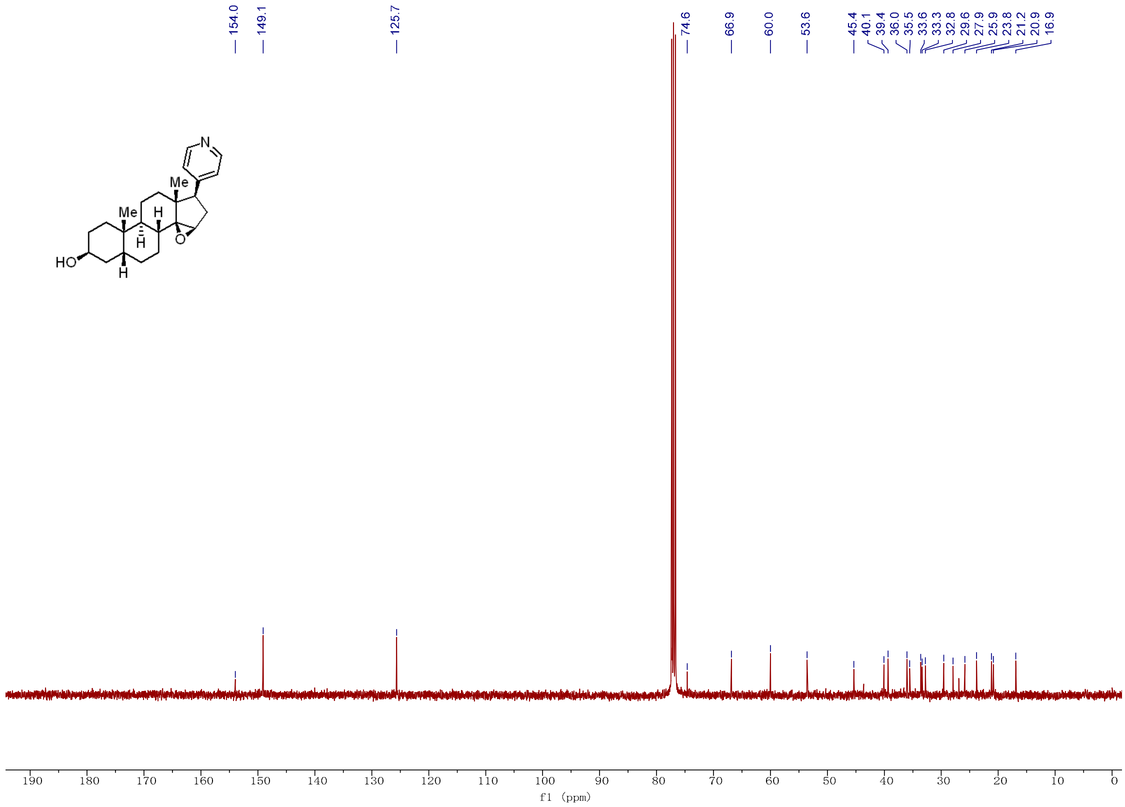

^{1}H NMR Spectrum of 2-4b (400 MHz, CDCl$_3$)

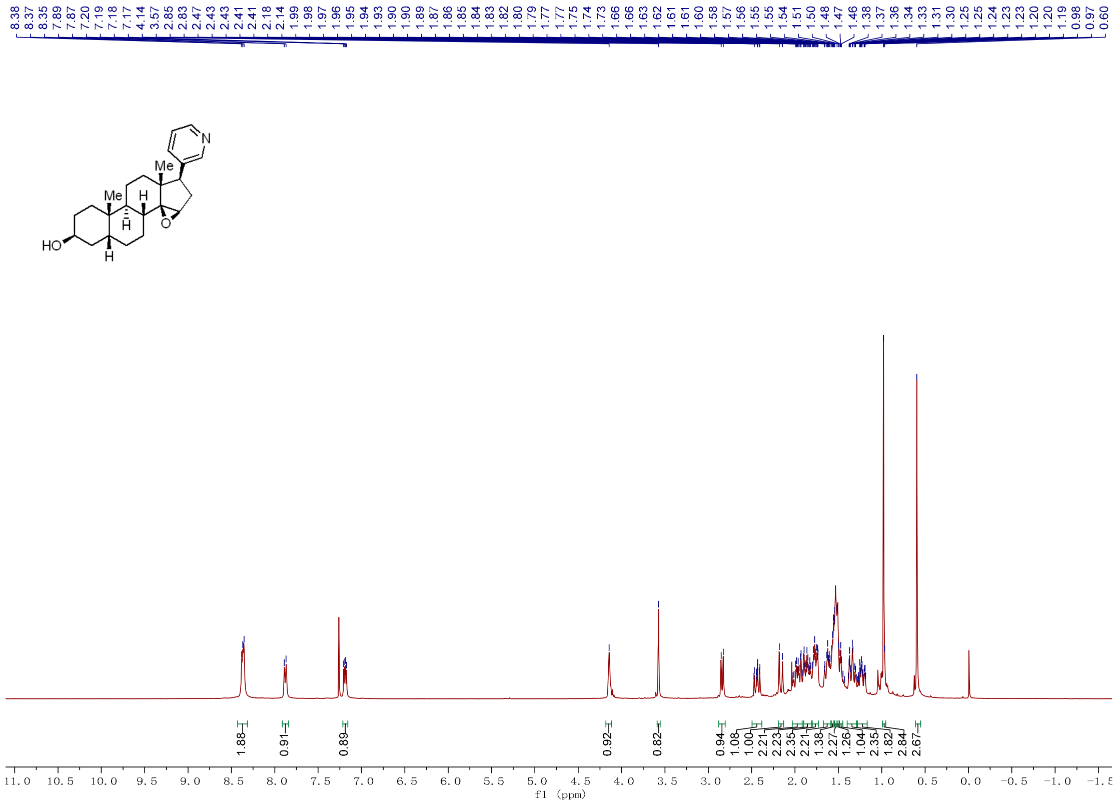

^{13}C NMR Spectrum of 2-4b (101 MHz, CDCl$_3$)

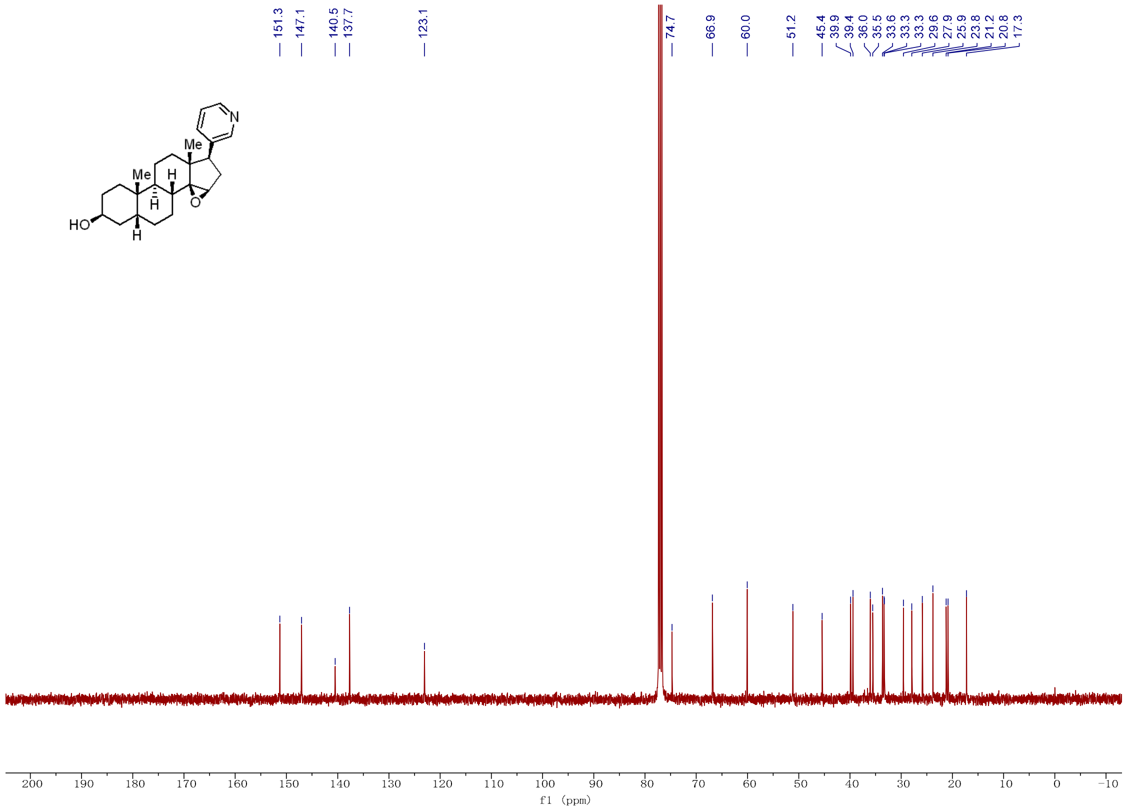

^{1}H NMR Spectrum of 2-4c (400 MHz, $CDCl_3$)

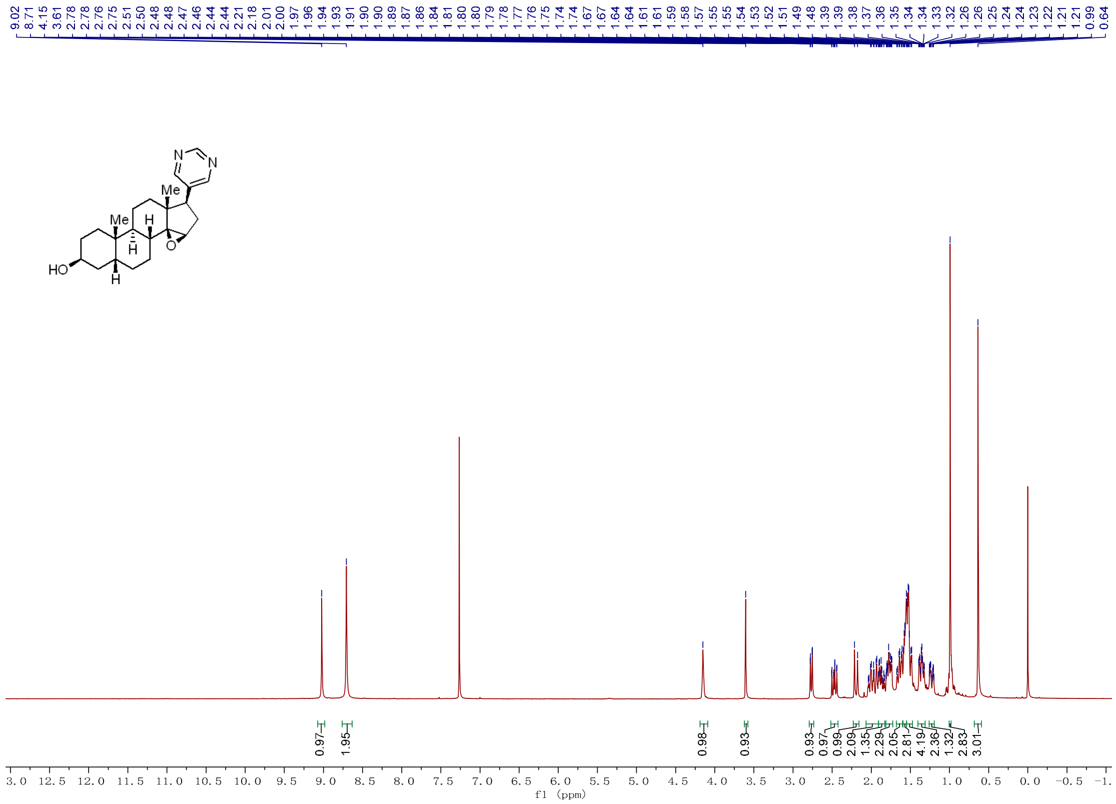

^{13}C NMR Spectrum of 2-4c (101 MHz, $CDCl_3$)

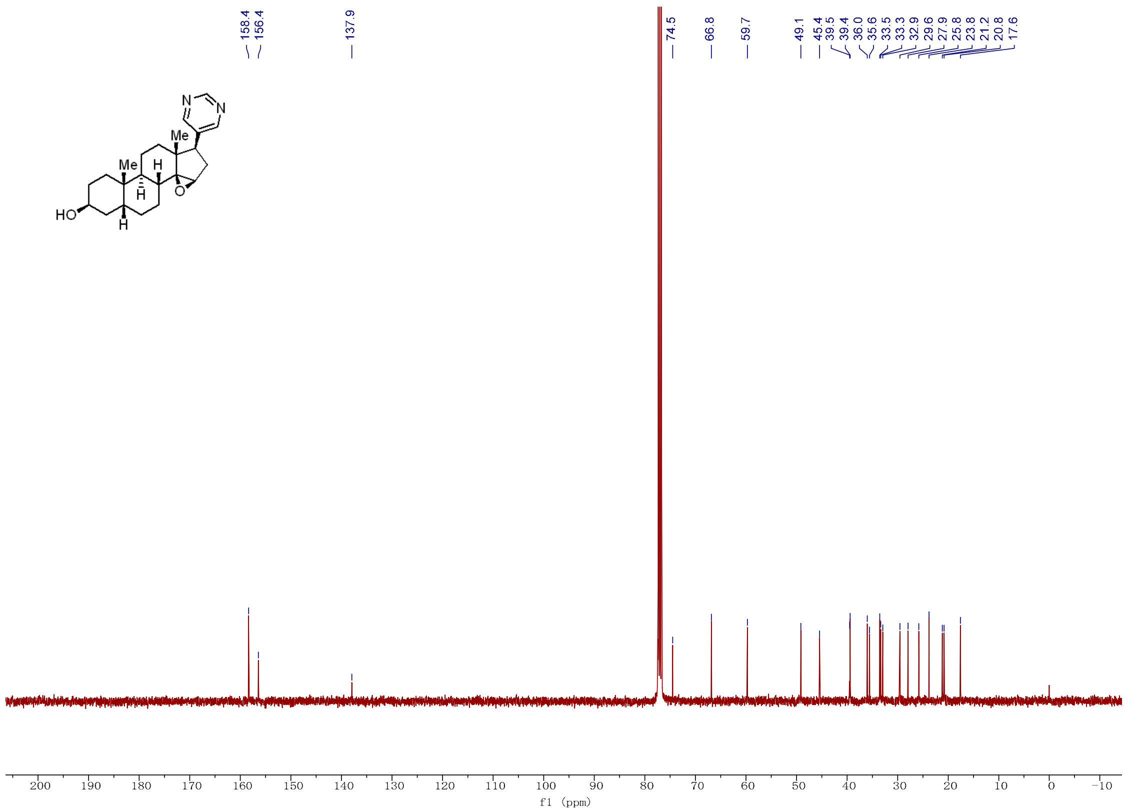

^{1}H NMR Spectrum of 2-4d (400 MHz, $CDCl_3$)

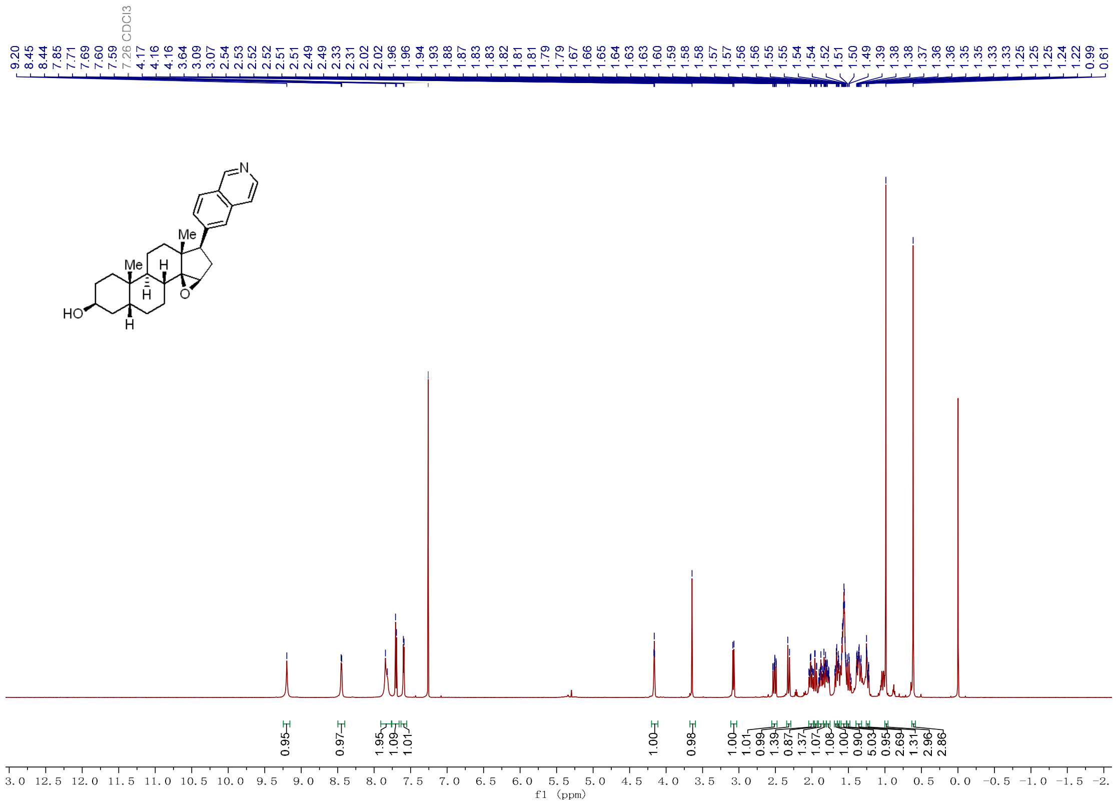

^{13}C NMR Spectrum of 2-4d (151 MHz, $CDCl_3$)

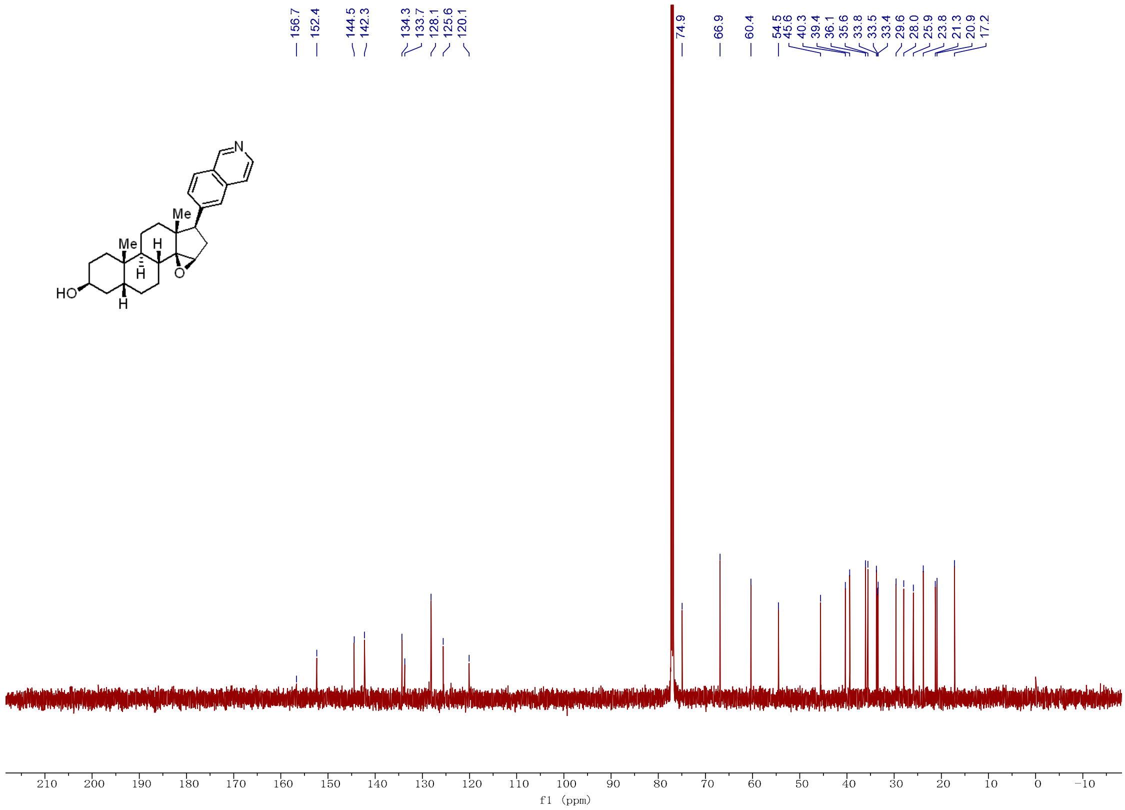

^{1}H NMR Spectrum of 2-4e (400 MHz, CDCl$_3$)

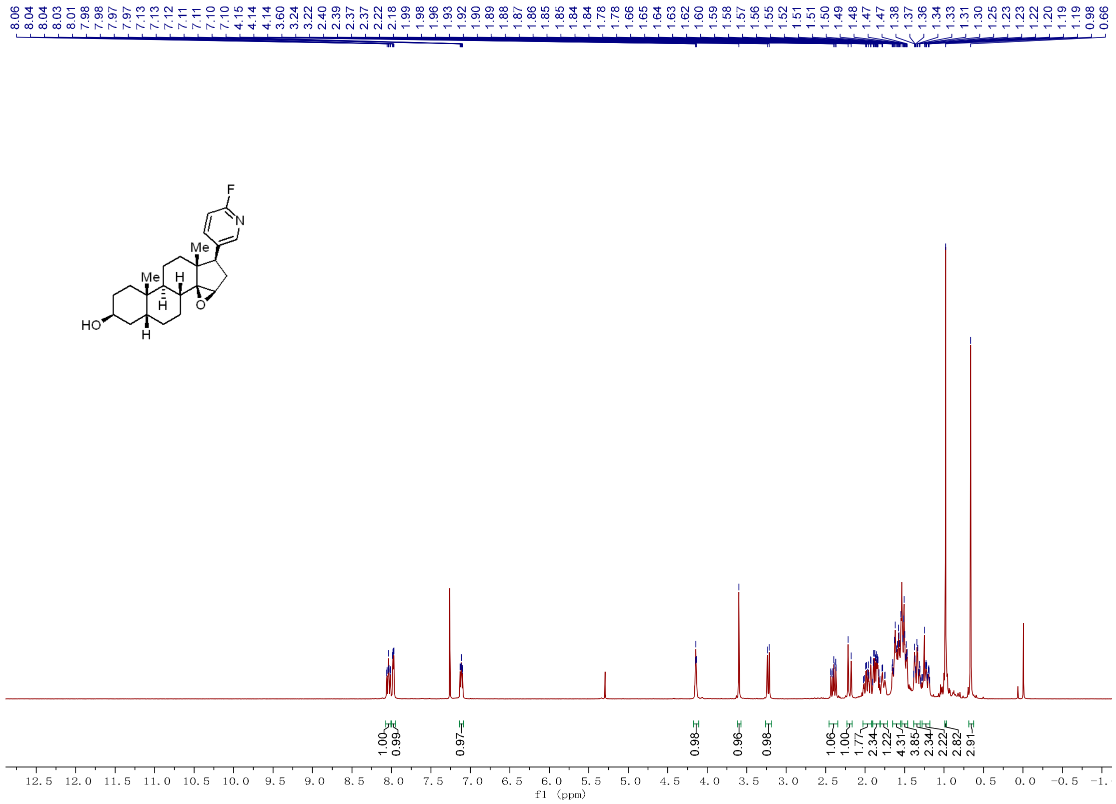

^{13}C NMR Spectrum of 2-4e (101 MHz, CDCl$_3$)

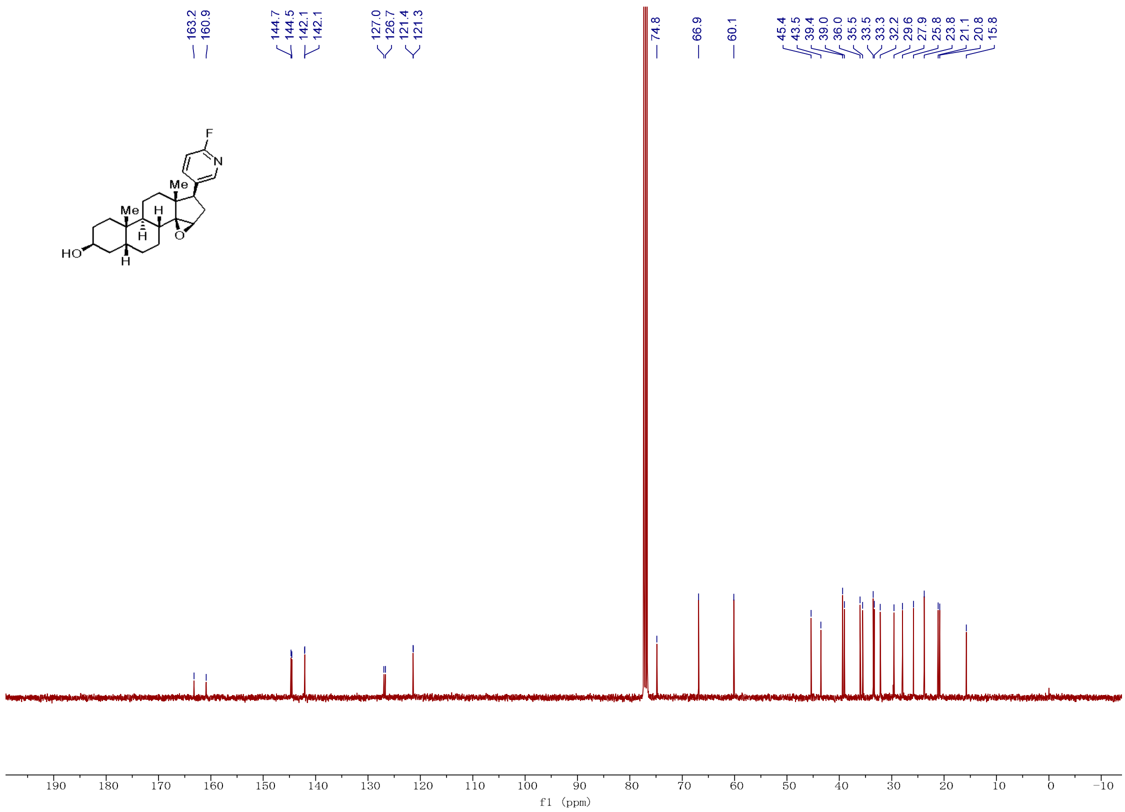

^{1}H NMR Spectrum of 2-4f (400 MHz, $CDCl_3$)

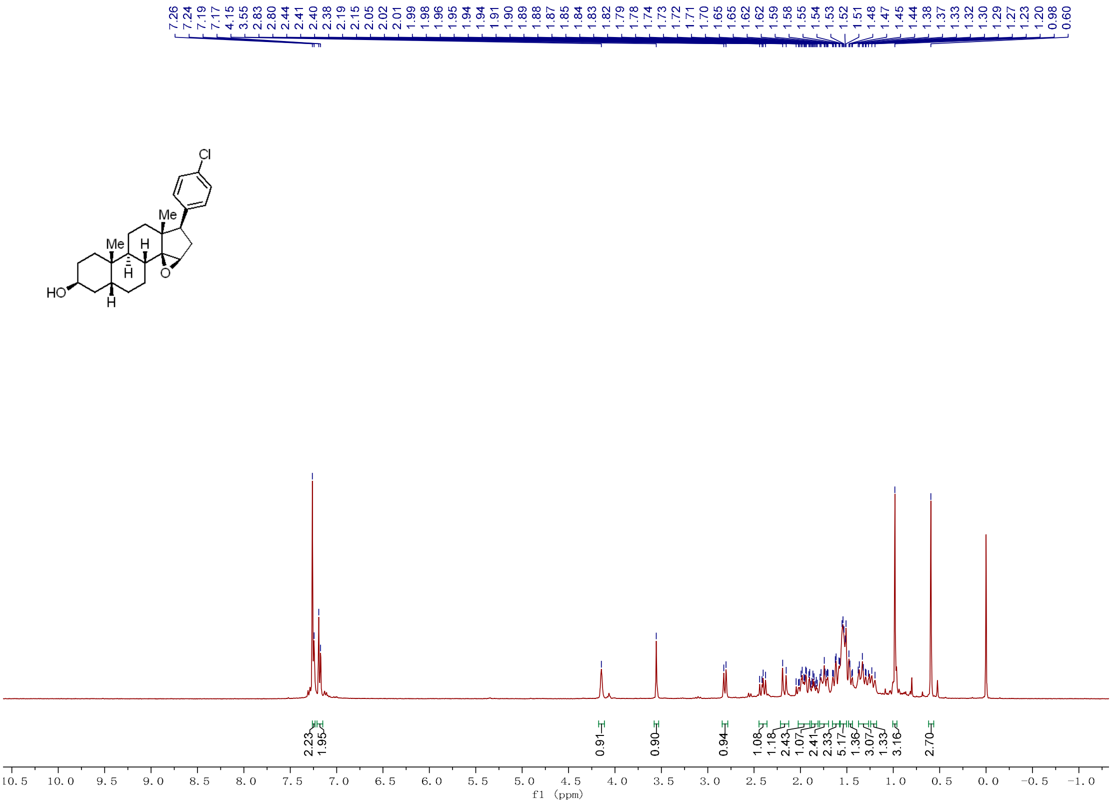

^{13}C NMR Spectrum of 2-4f (101 MHz, $CDCl_3$)

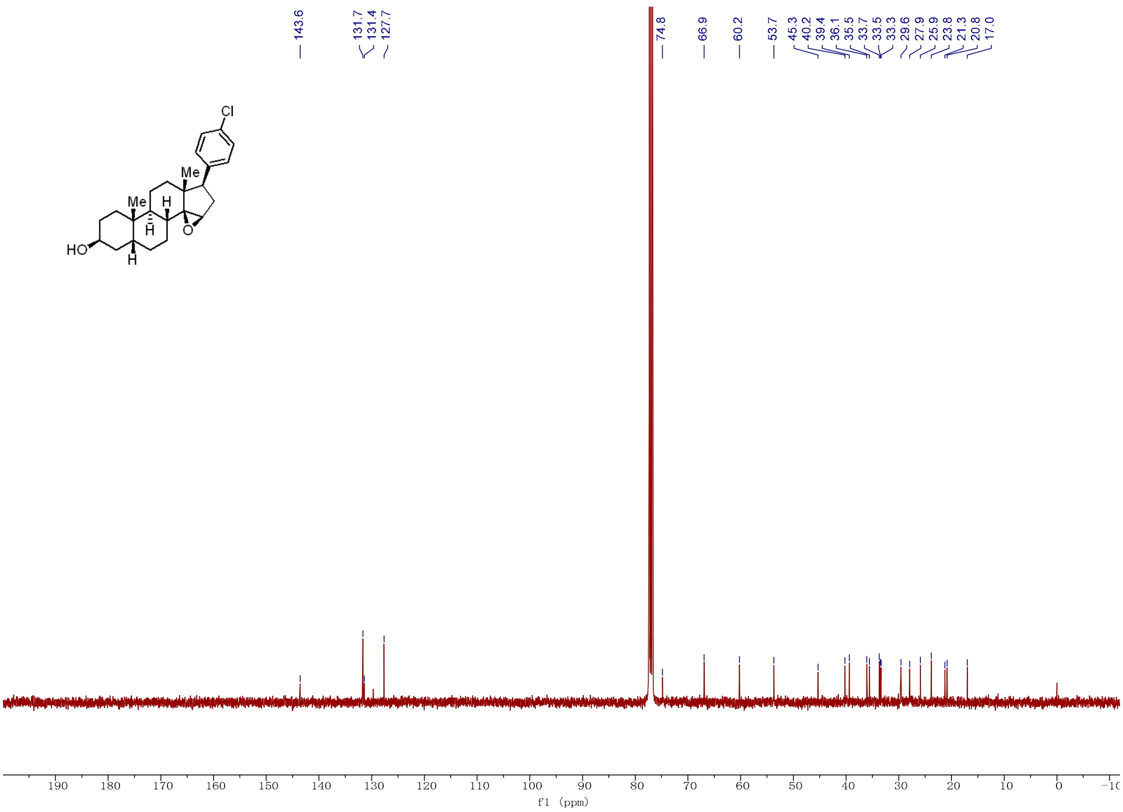

^{1}H NMR Spectrum of 2-4g (400 MHz, $CDCl_3$)

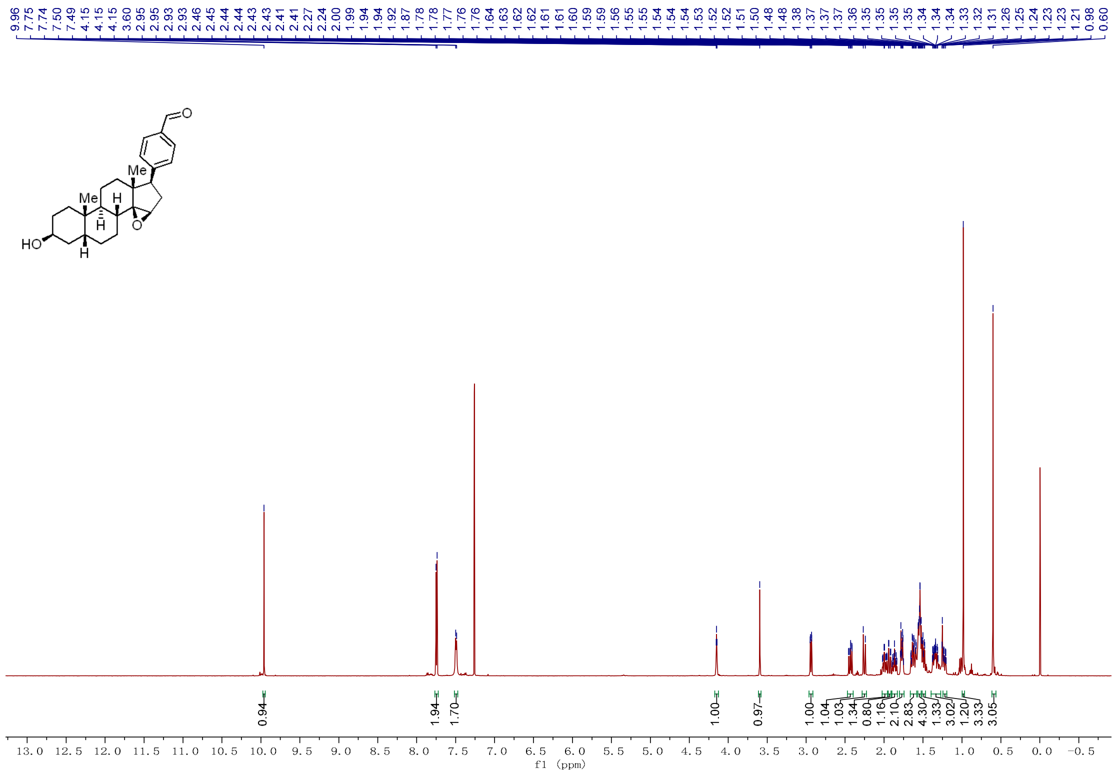

^{13}C NMR Spectrum of 2-4g (101 MHz, $CDCl_3$)

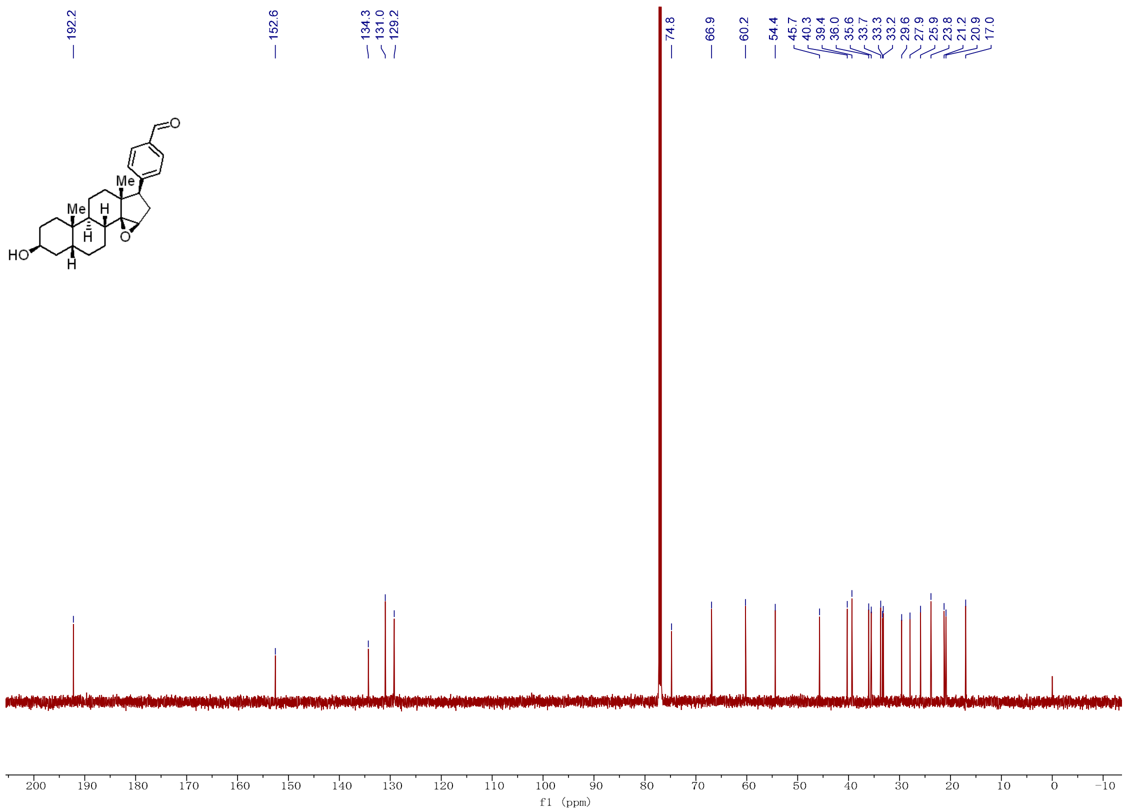

^{1}H NMR Spectrum of 2-4h (400 MHz, $CDCl_3$)

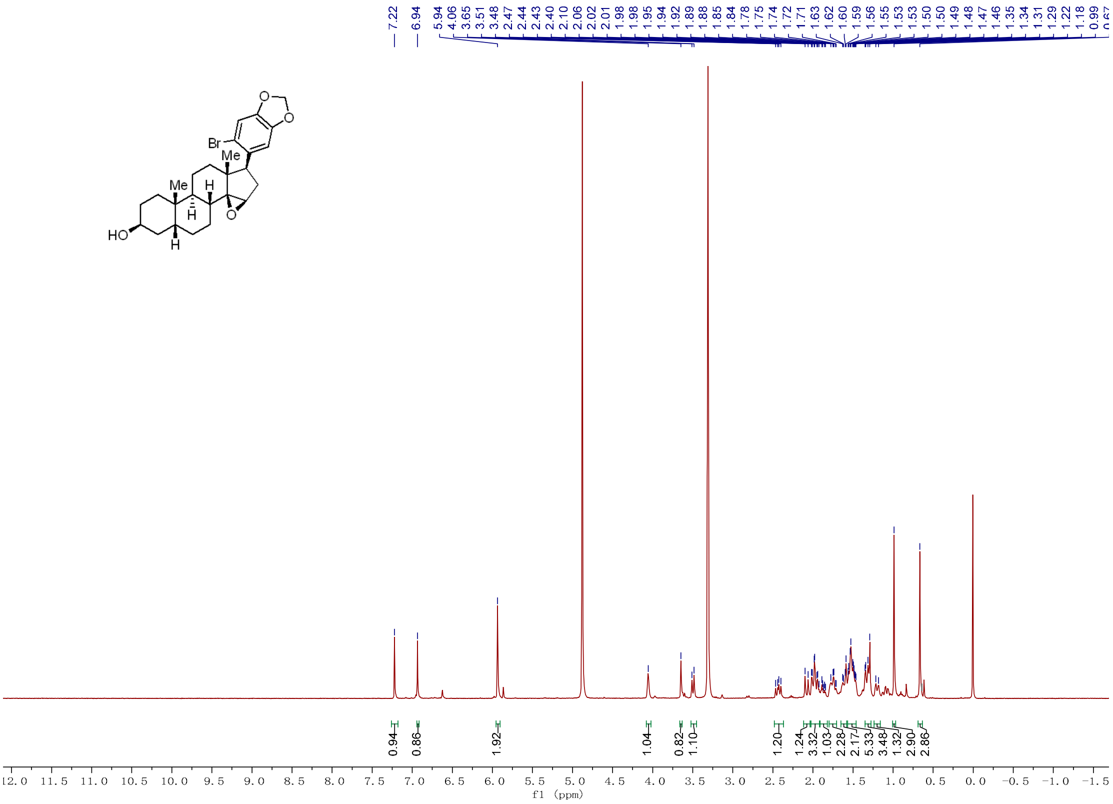

^{13}C NMR Spectrum of 2-4h (101 MHz, $CDCl_3$)

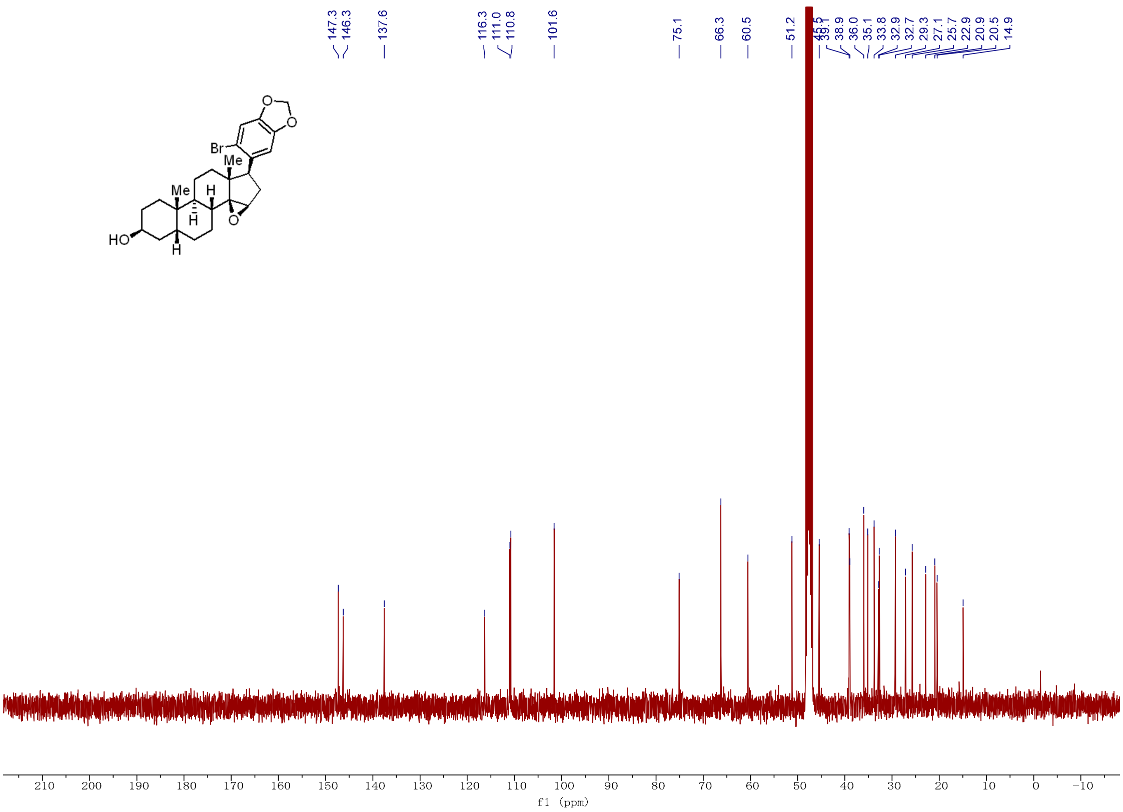

HMQC NMR Spectrum of 2-4h (101 MHz, $CDCl_3$)

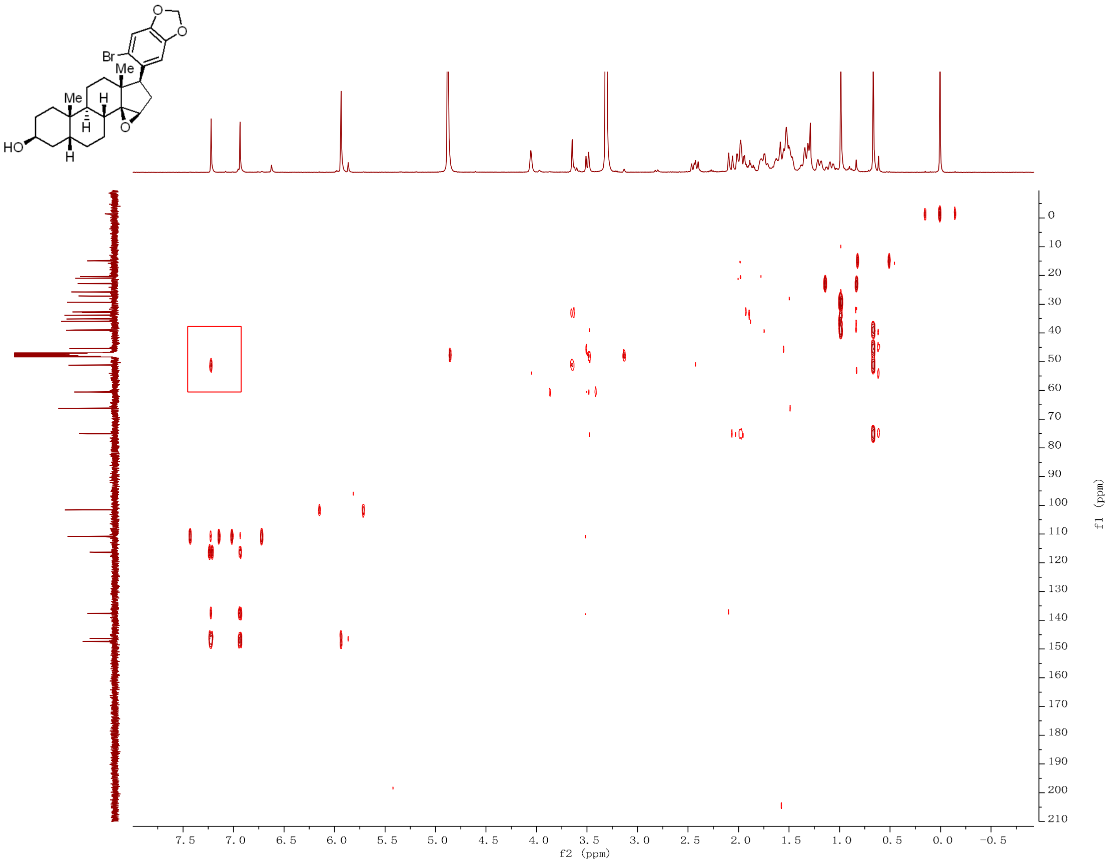

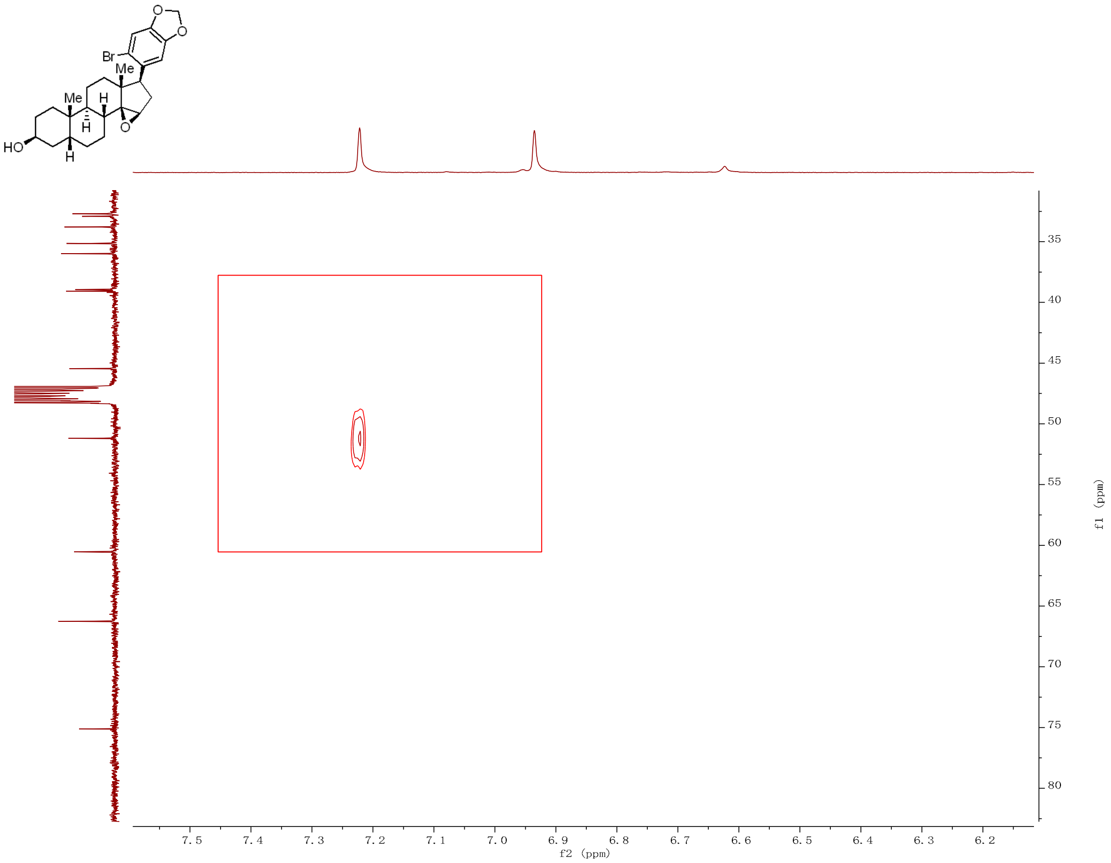

^{1}H NMR Spectrum of 2-4i (400 MHz, $CDCl_3$)

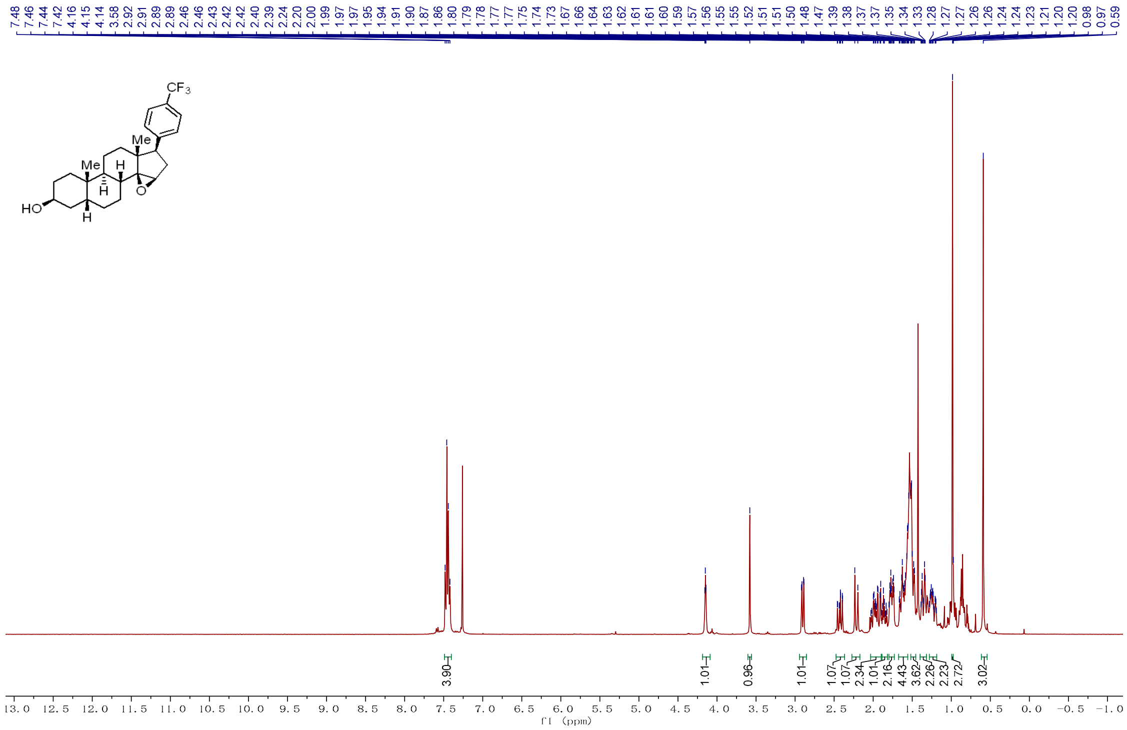

^{13}C NMR Spectrum of 2-4i (101 MHz, $CDCl_3$)

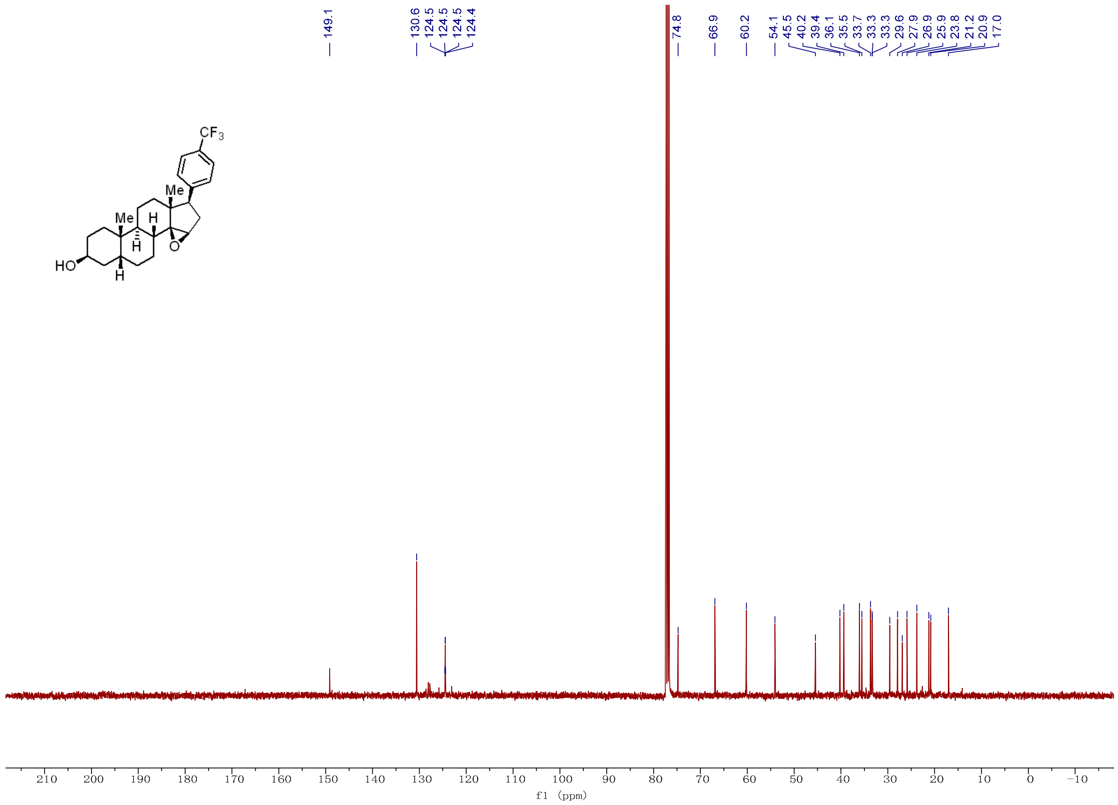

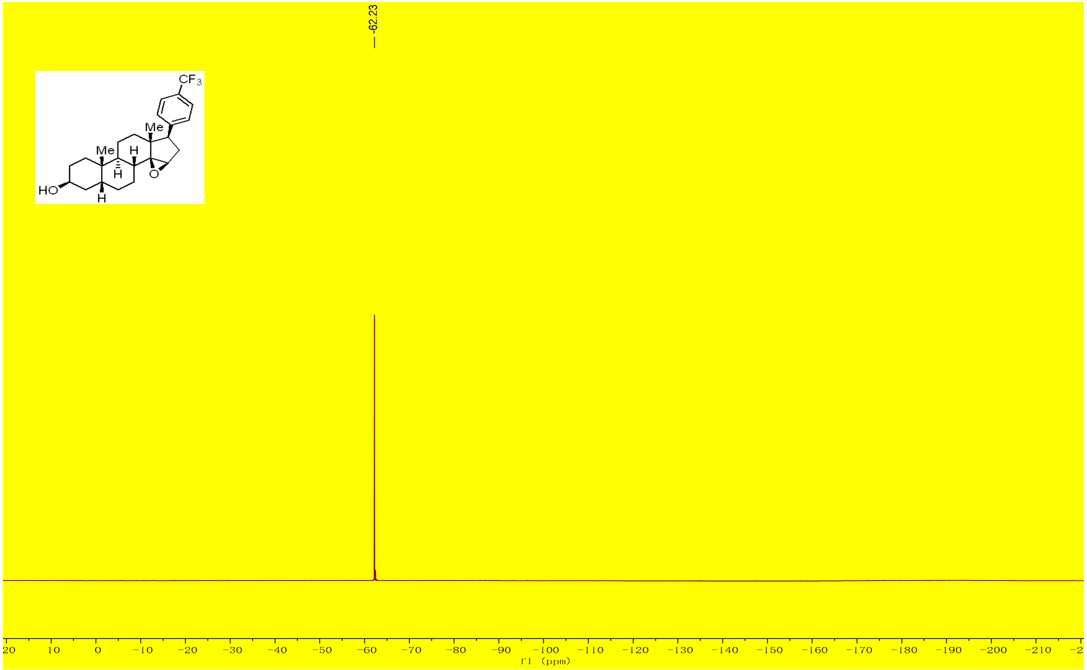

-62.23
CF3
Me
Me
H
H
H
HO
O
20 10 0 −10 −20 −30 −40 −50 −60 −70 −80 −90 −100 −110 −120 −130 −140 −150 −160 −170 −180 −190 −200 −210
f1 (ppm)

^{1}H NMR Spectrum of 2-4j (400 MHz, $CDCl_3$)

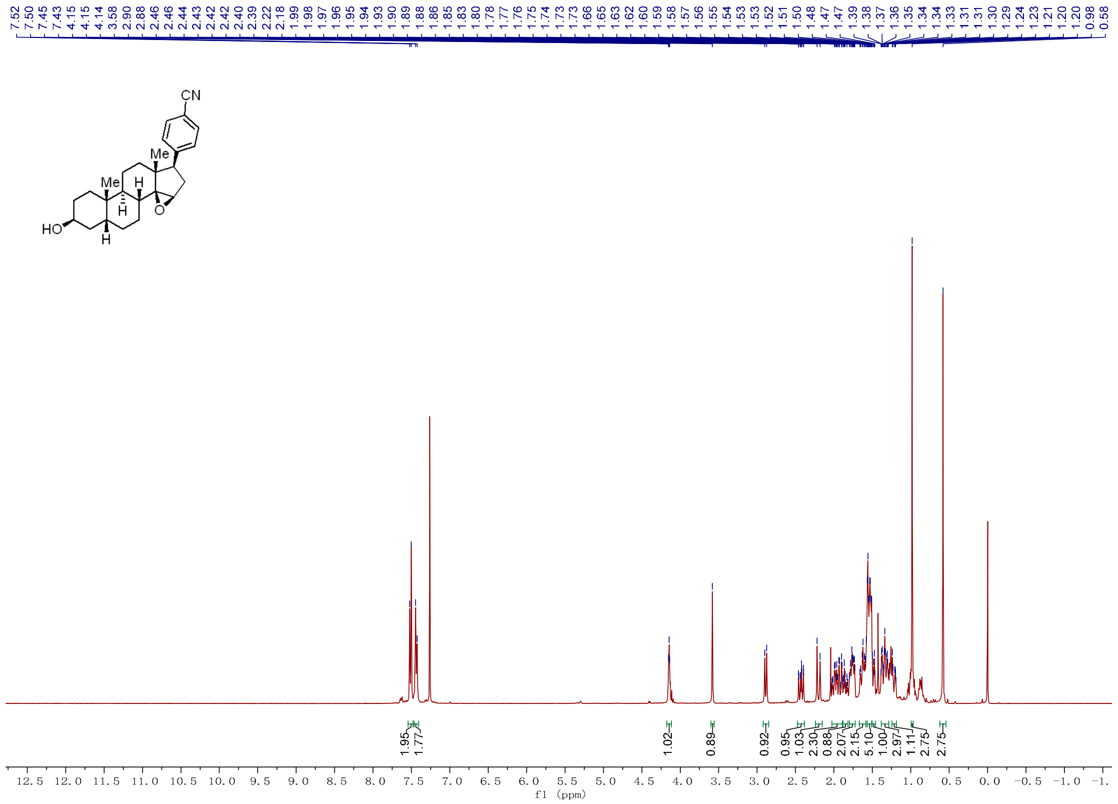

^{13}C NMR Spectrum of 2-4j (101 MHz, $CDCl_3$)

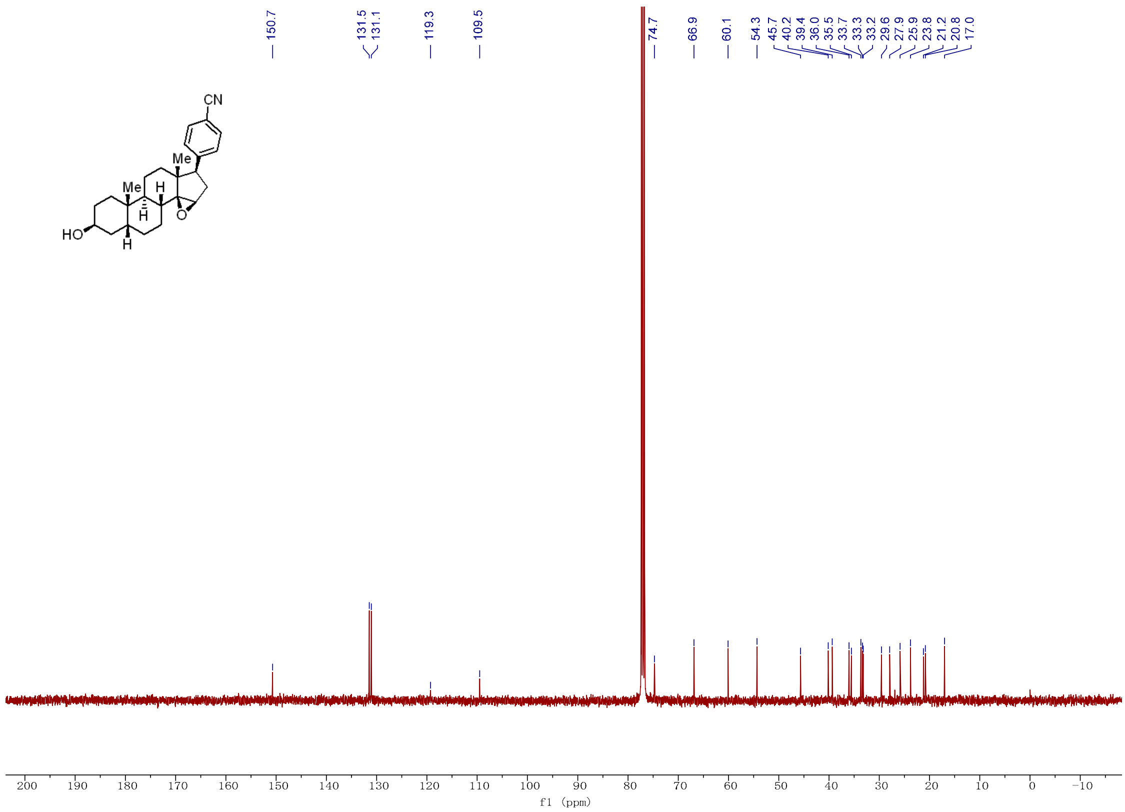

^{1}H NMR Spectrum of 2-4k (400 MHz, $CDCl_3$)

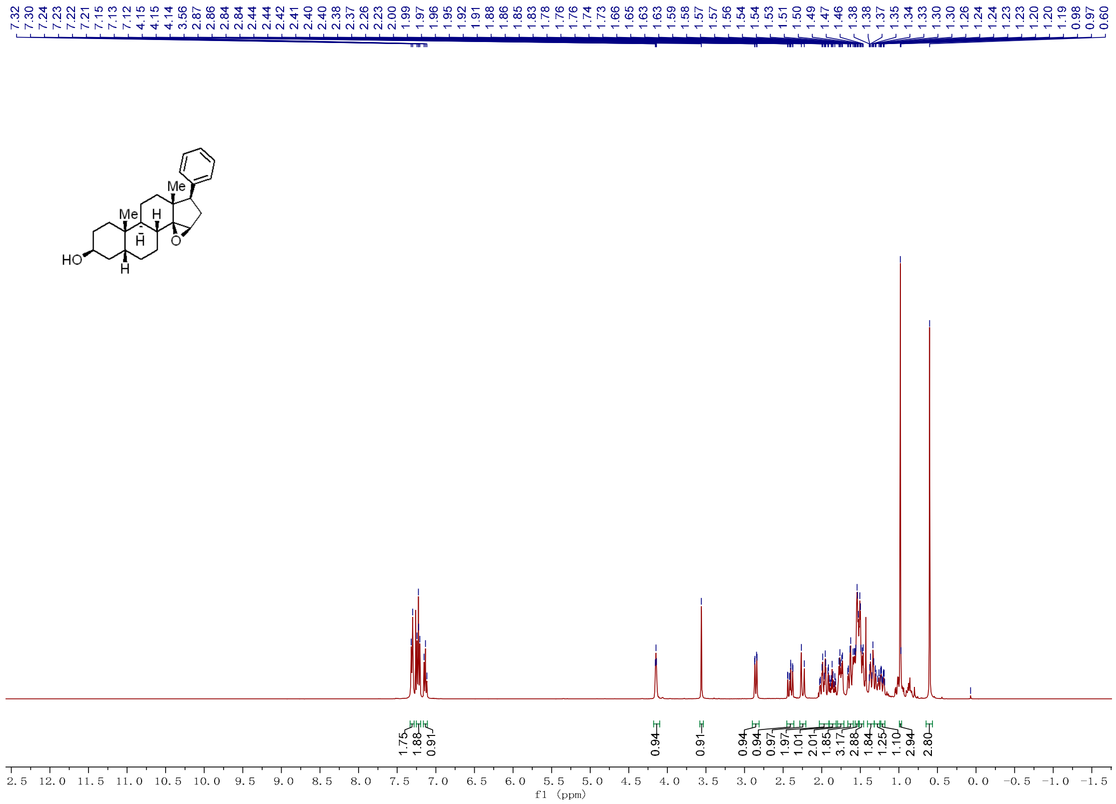

^{13}C NMR Spectrum of 2-4k (101 MHz, $CDCl_3$)

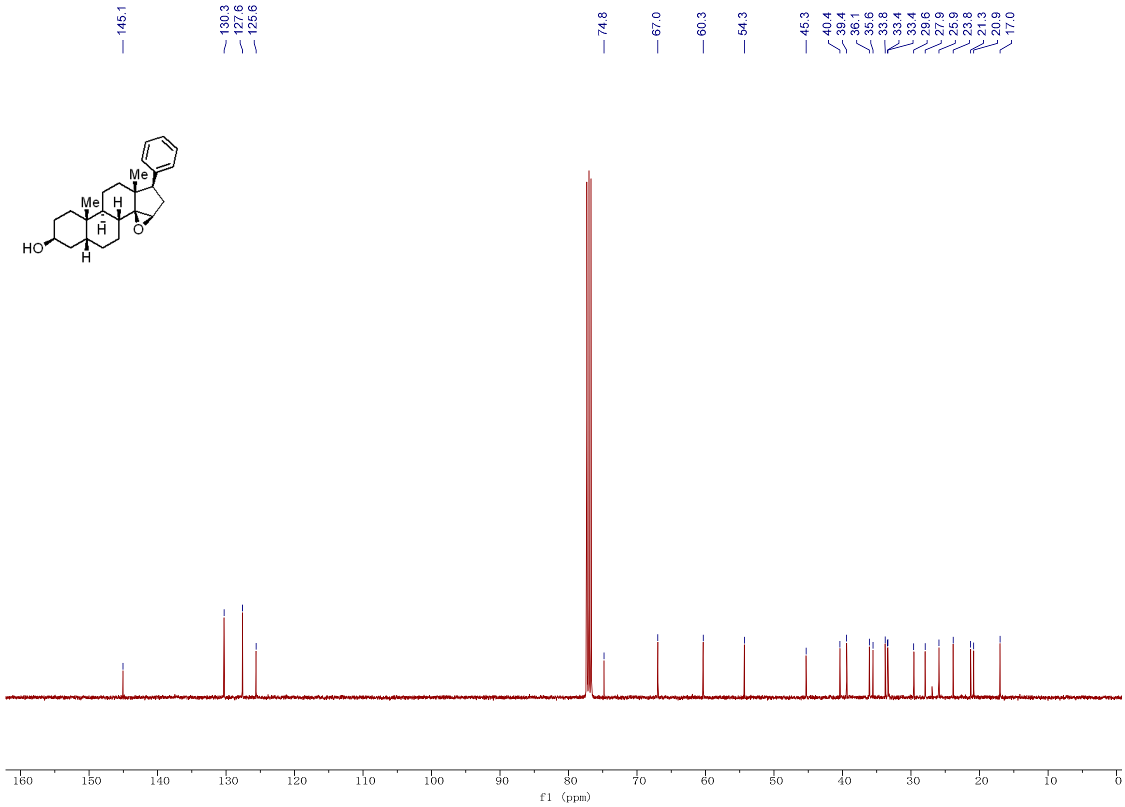

^{1}H NMR Spectrum of 2-4l (400 MHz, $CDCl_3$)

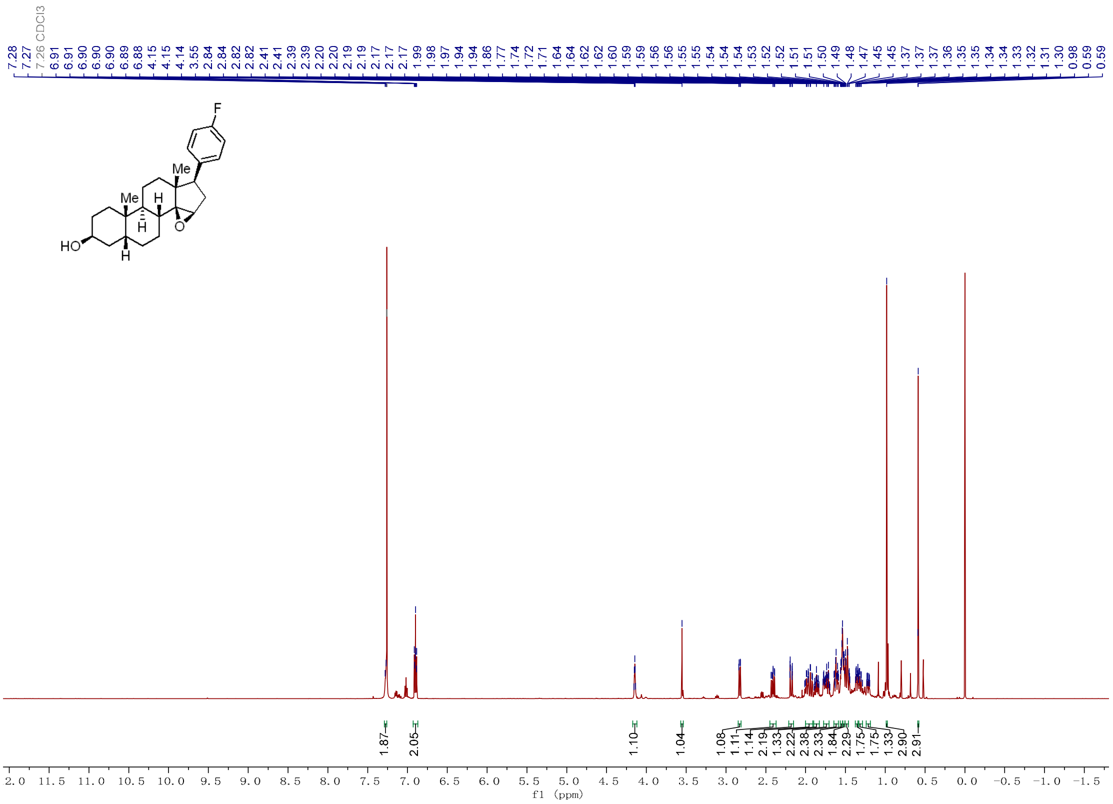

^{13}C NMR Spectrum of 2-4l (101 MHz, $CDCl_3$)

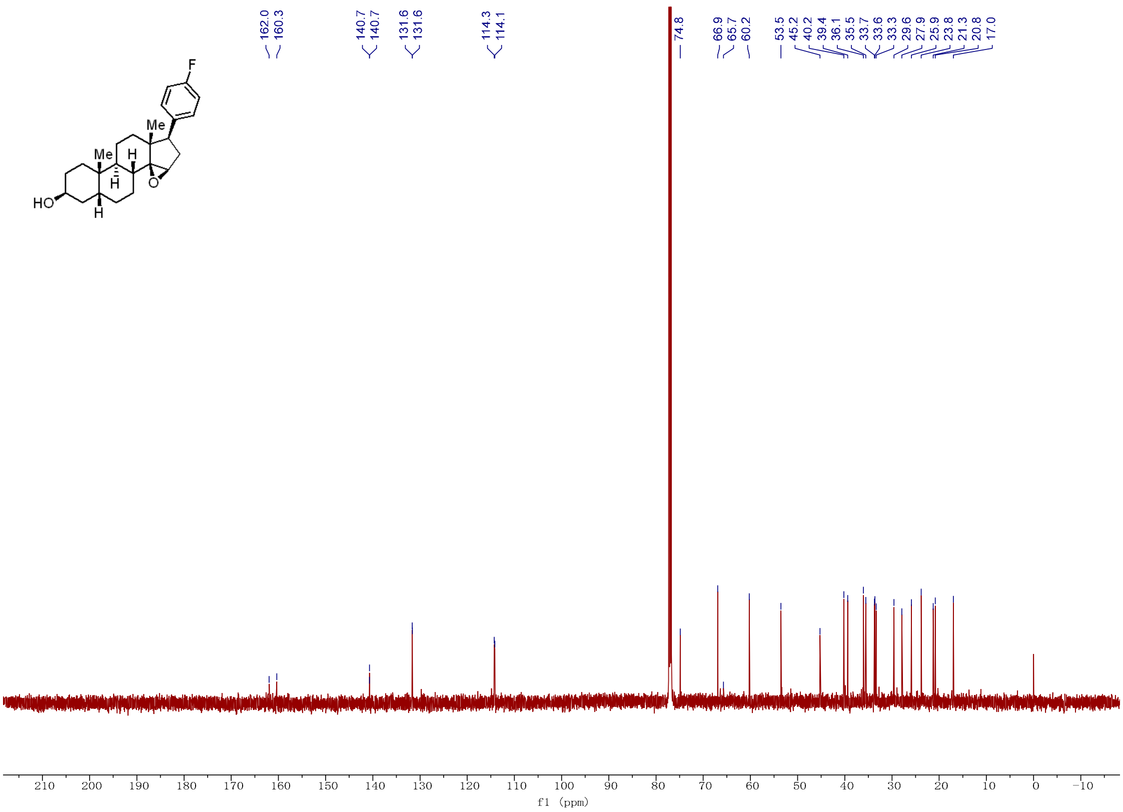

^{1}H NMR Spectrum of 2-5a (400 MHz, $CDCl_3$)

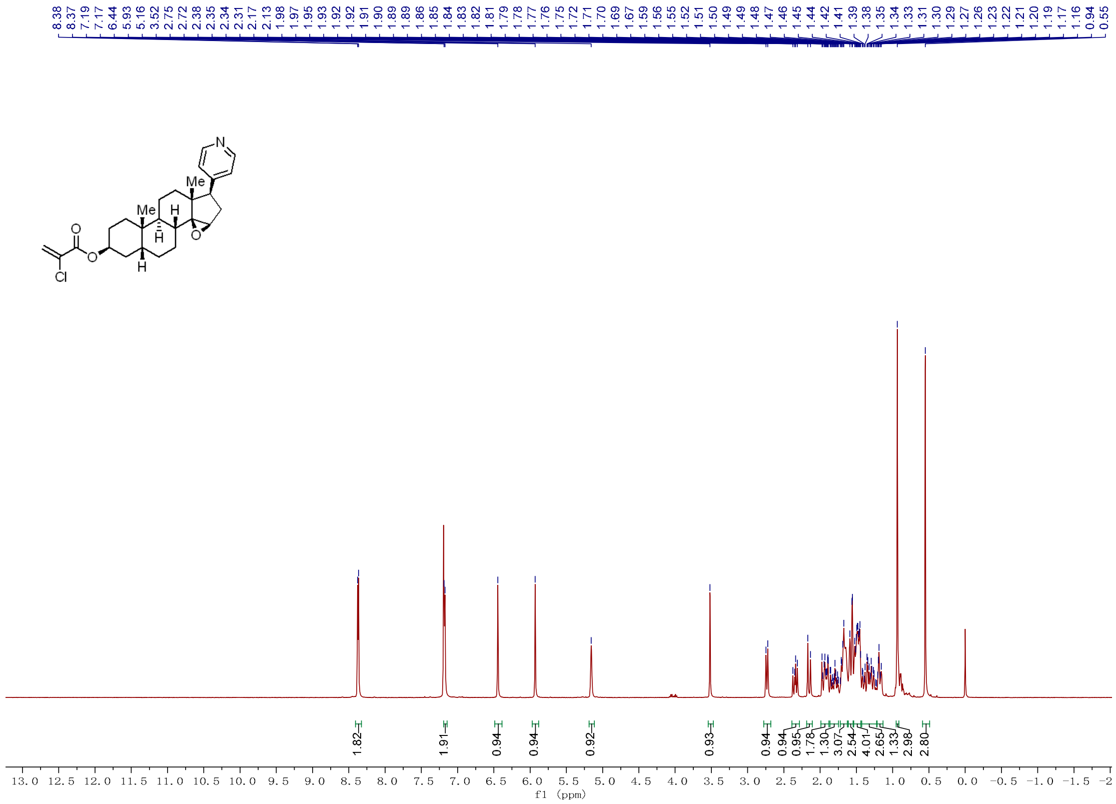

^{13}C NMR Spectrum of 2-5a (101 MHz, $CDCl_3$)

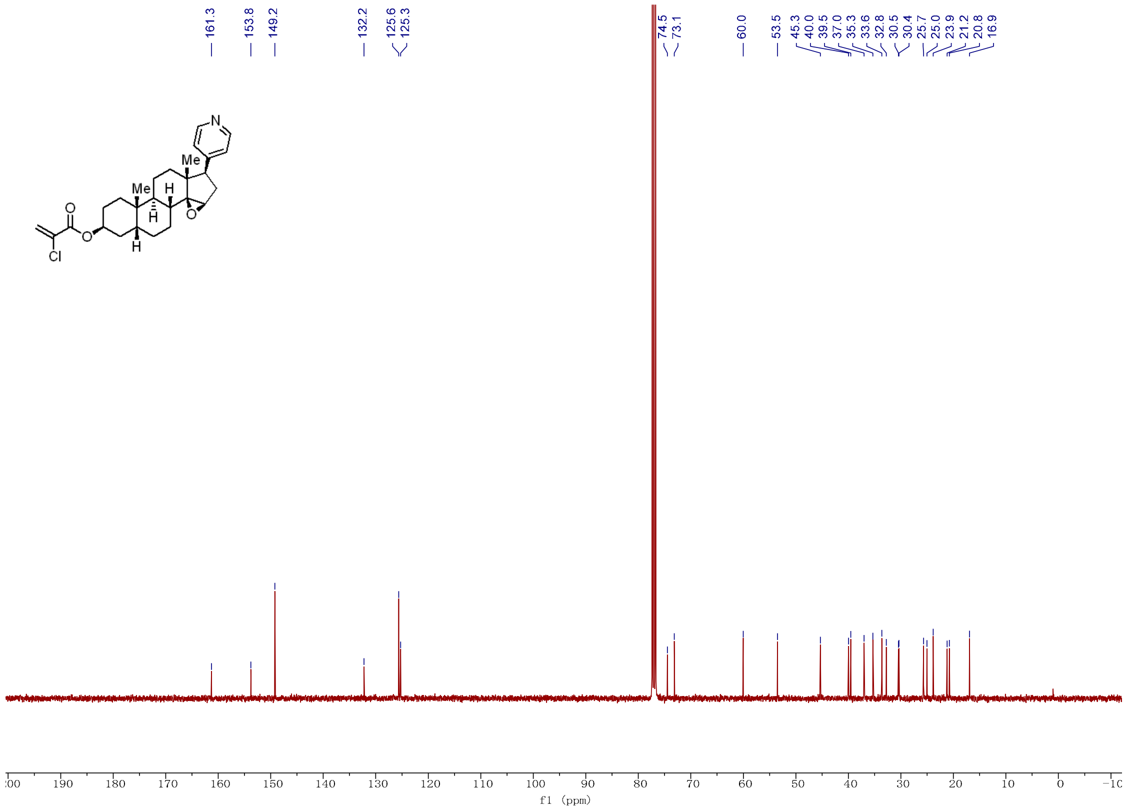

^{1}H NMR Spectrum of 2-5b (400 MHz, $CDCl_3$)

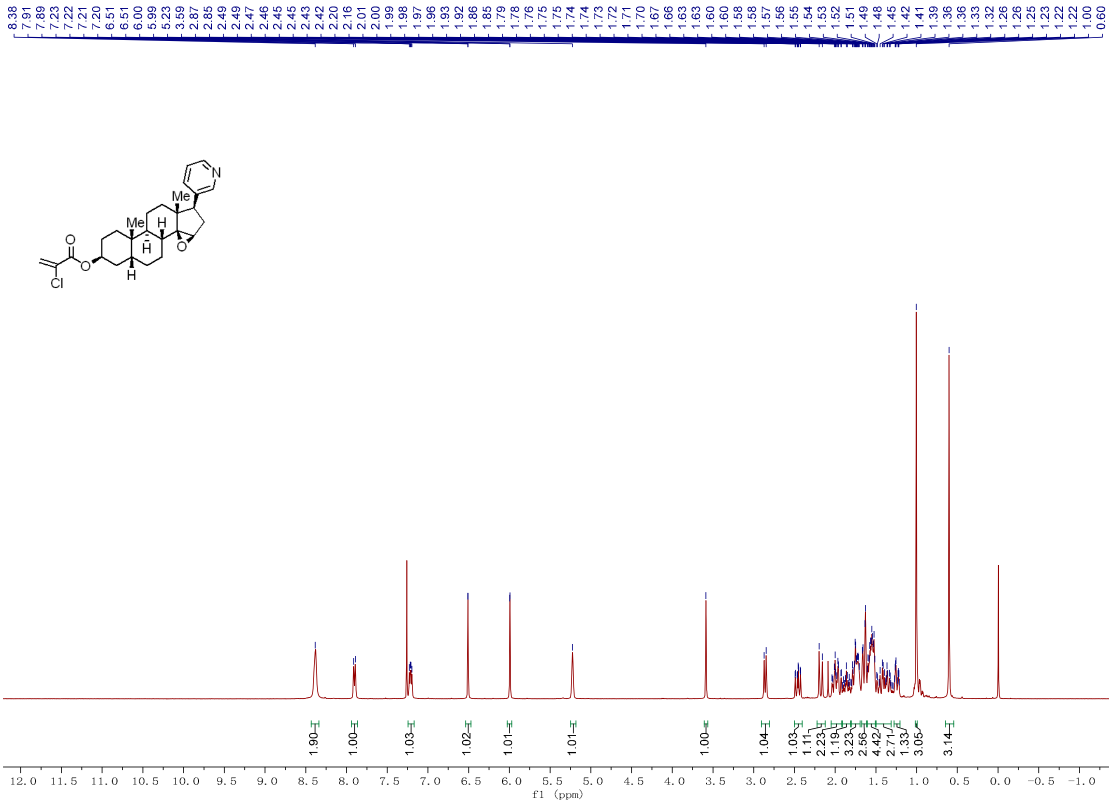

^{13}C NMR Spectrum of 2-5b (101 MHz, $CDCl_3$)

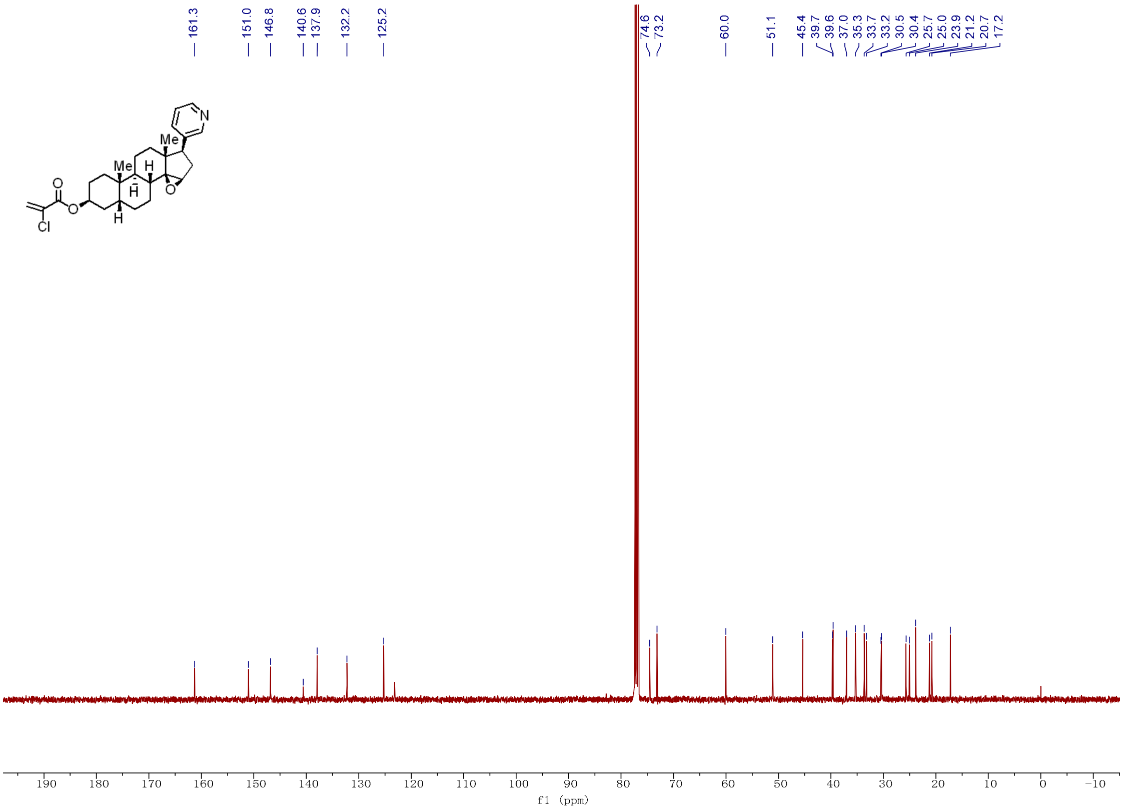

^{1}H NMR Spectrum of 2-5c (400 MHz, $CDCl_3$)

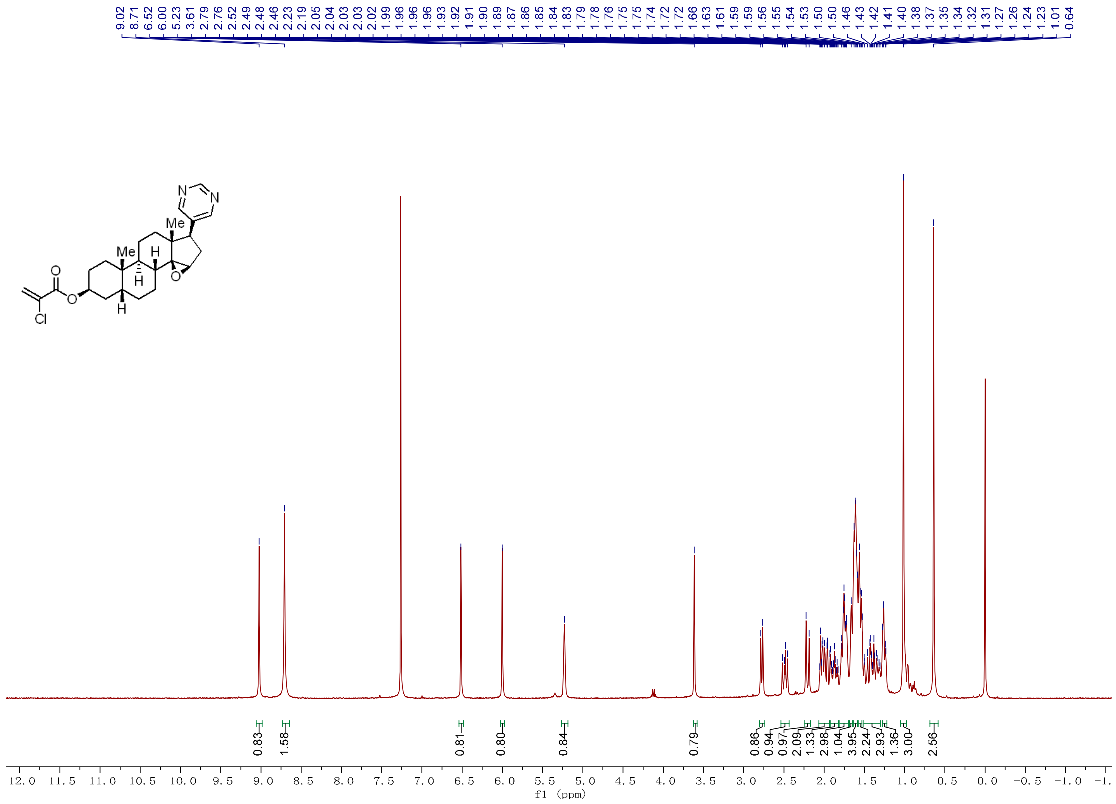

^{13}C NMR Spectrum of 2-5c (101 MHz, $CDCl_3$)

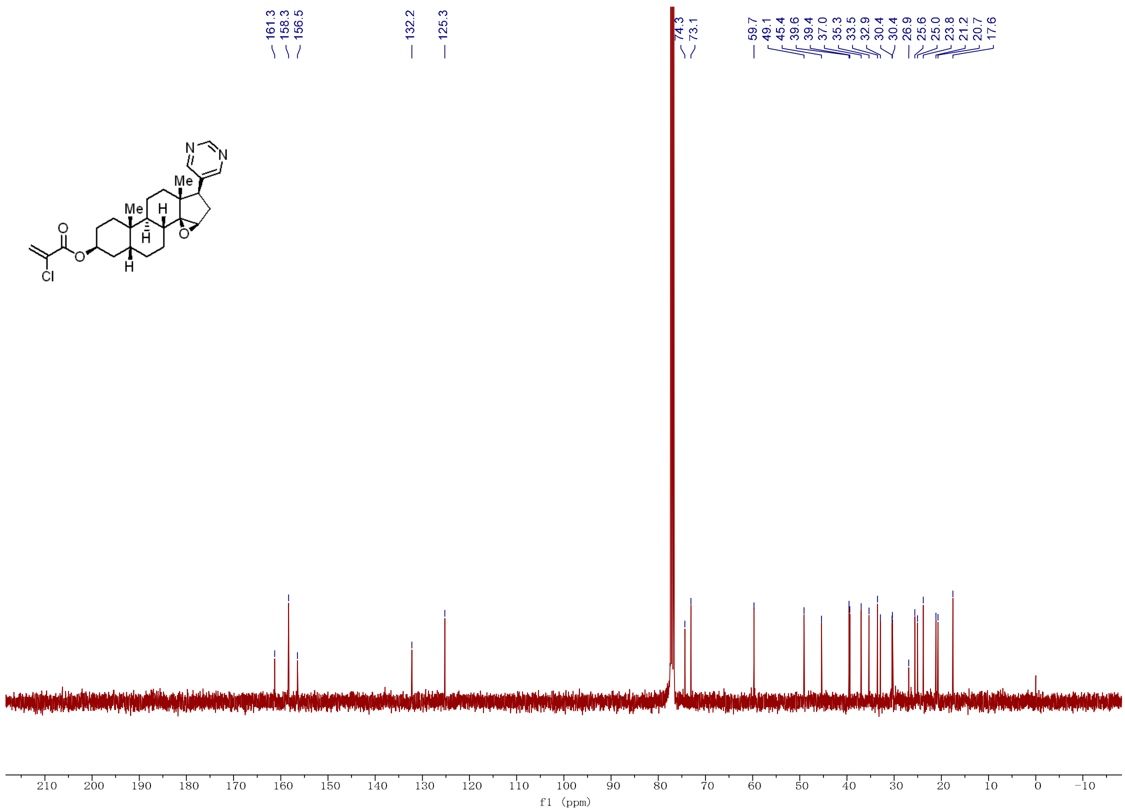

^{1}H NMR Spectrum of 2-5d (400 MHz, $CDCl_3$)

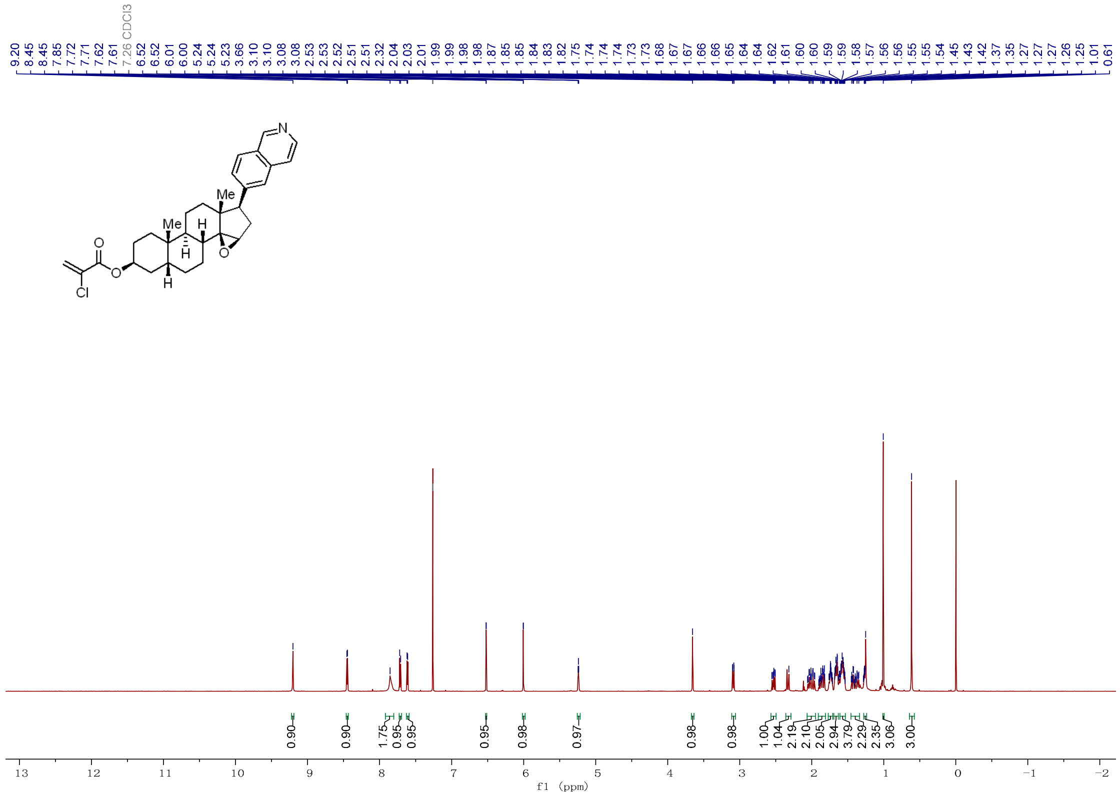

^{13}C NMR Spectrum of 2-5d (151 MHz, $CDCl_3$)

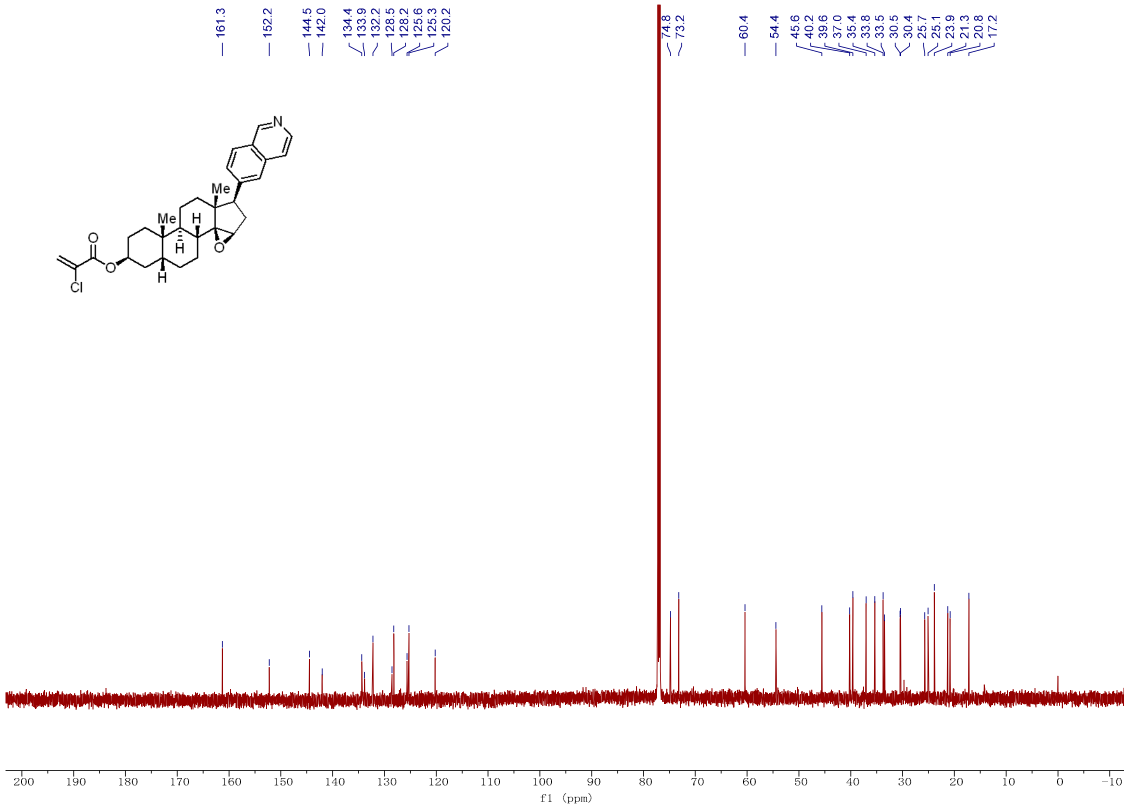

^{1}H NMR Spectrum of 2-5e (400 MHz, $CDCl_3$)

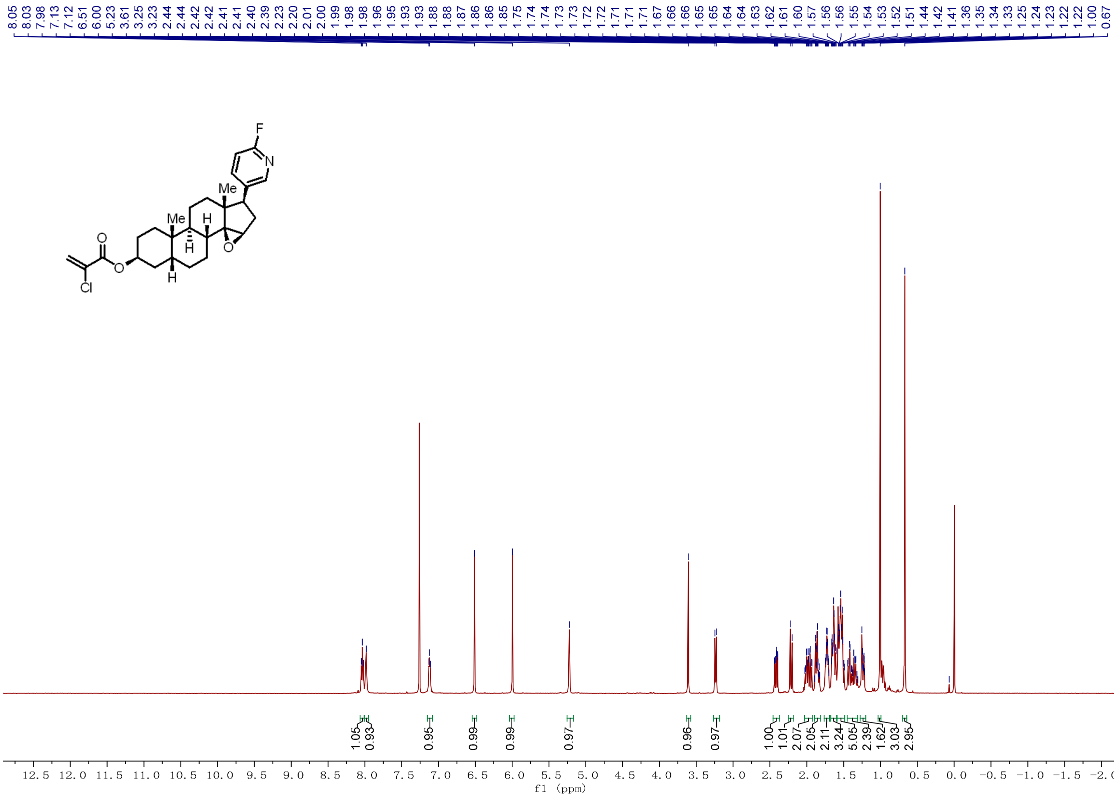

^{13}C NMR Spectrum of 2-5e (151 MHz, $CDCl_3$)

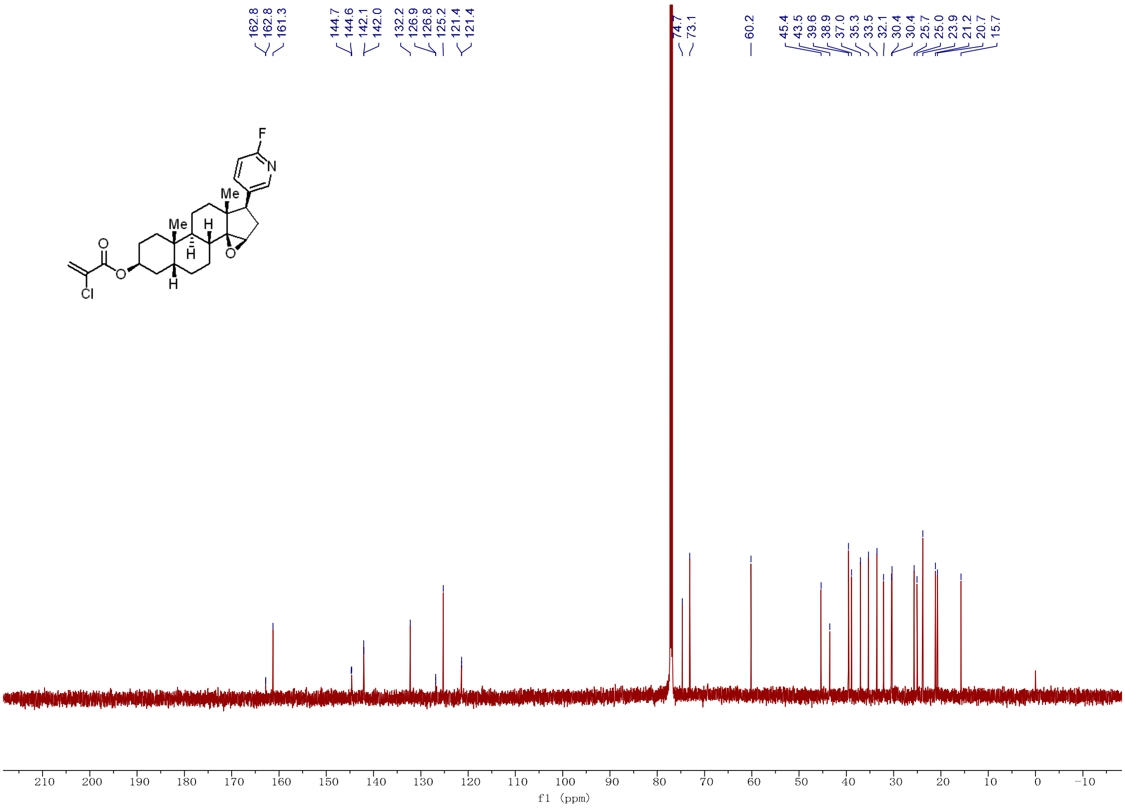

^{1}H NMR Spectrum of 2-5f (400 MHz, CDCl$_3$)

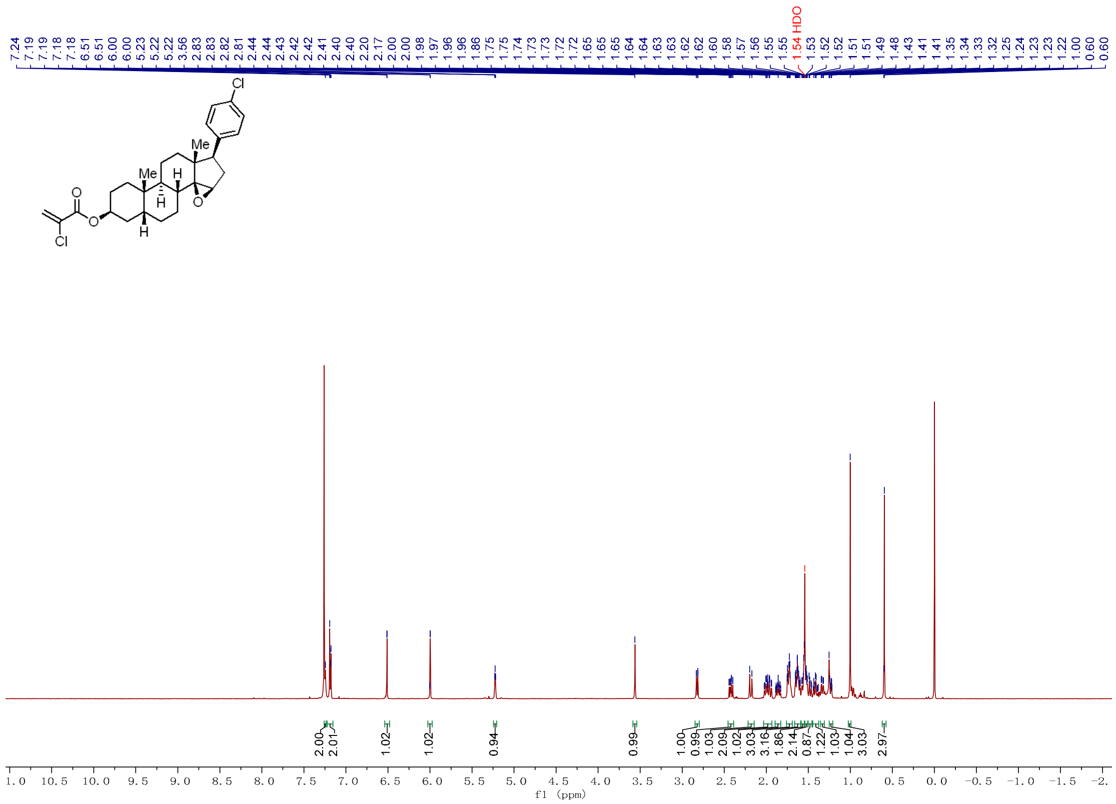

^{13}C NMR Spectrum of 2-5f (151 MHz, CDCl$_3$)

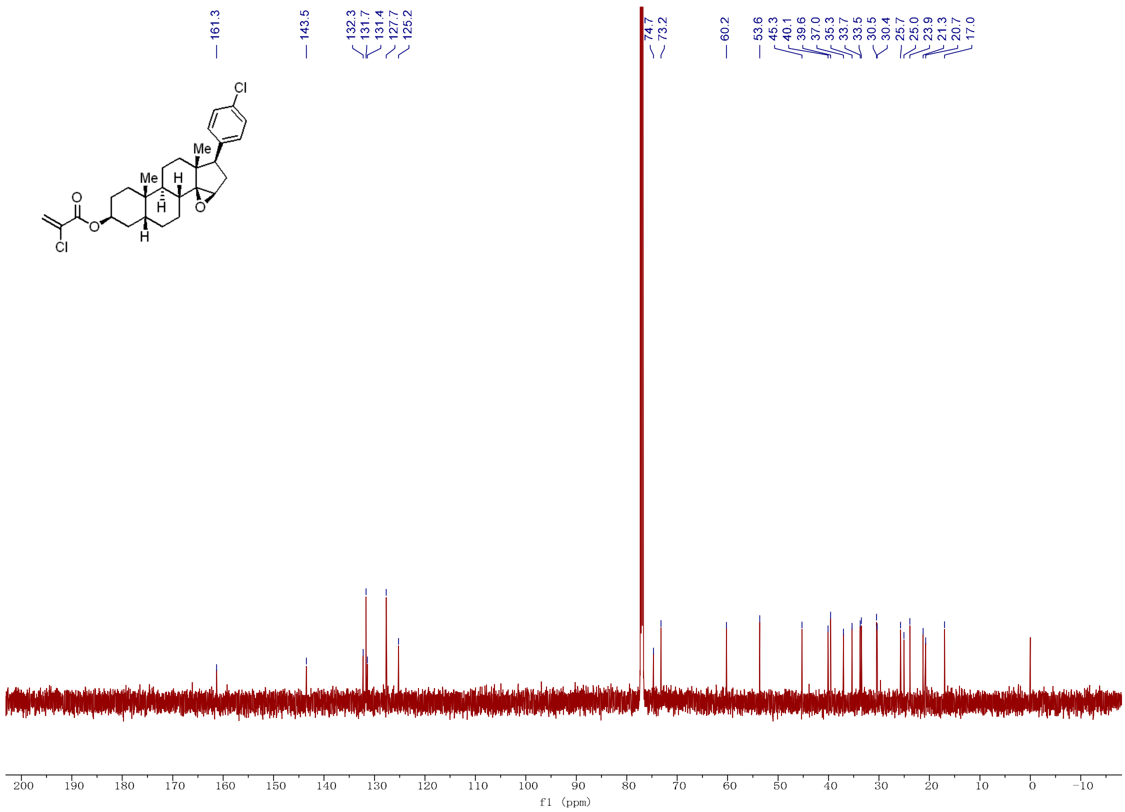

^{1}H NMR Spectrum of 2-5g (400 MHz, $CDCl_3$)

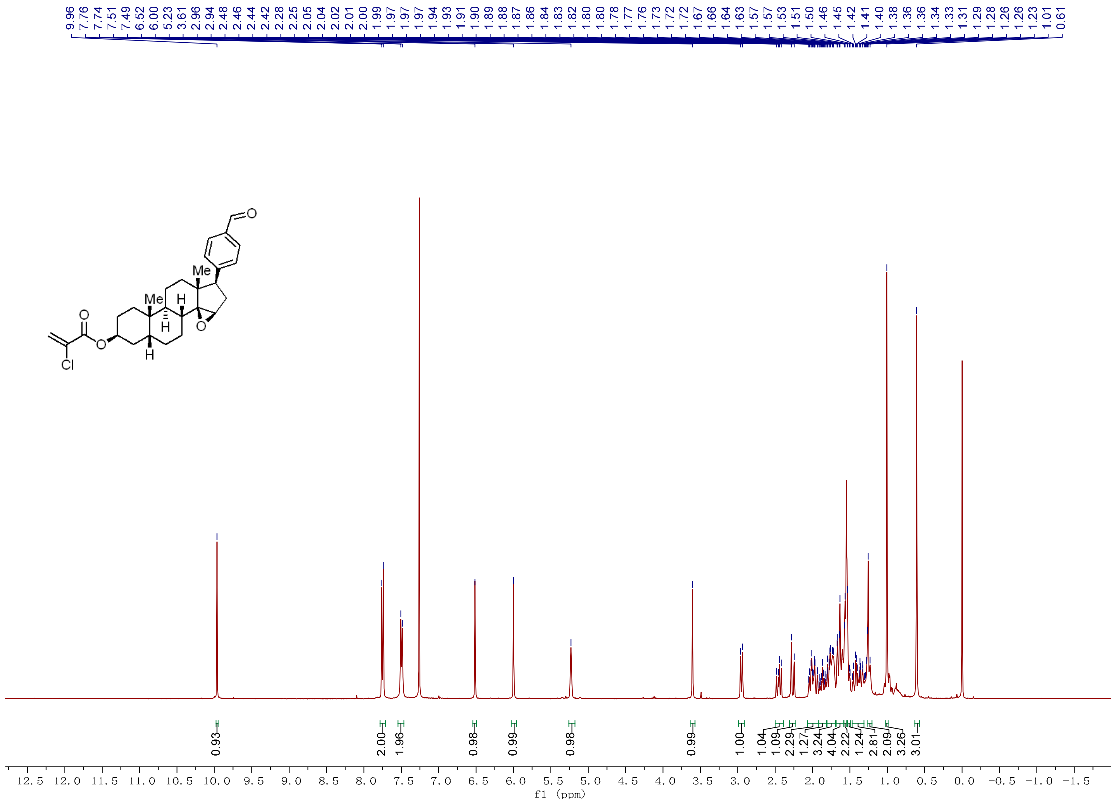

^{13}C NMR Spectrum of 2-5g (101 MHz, $CDCl_3$)

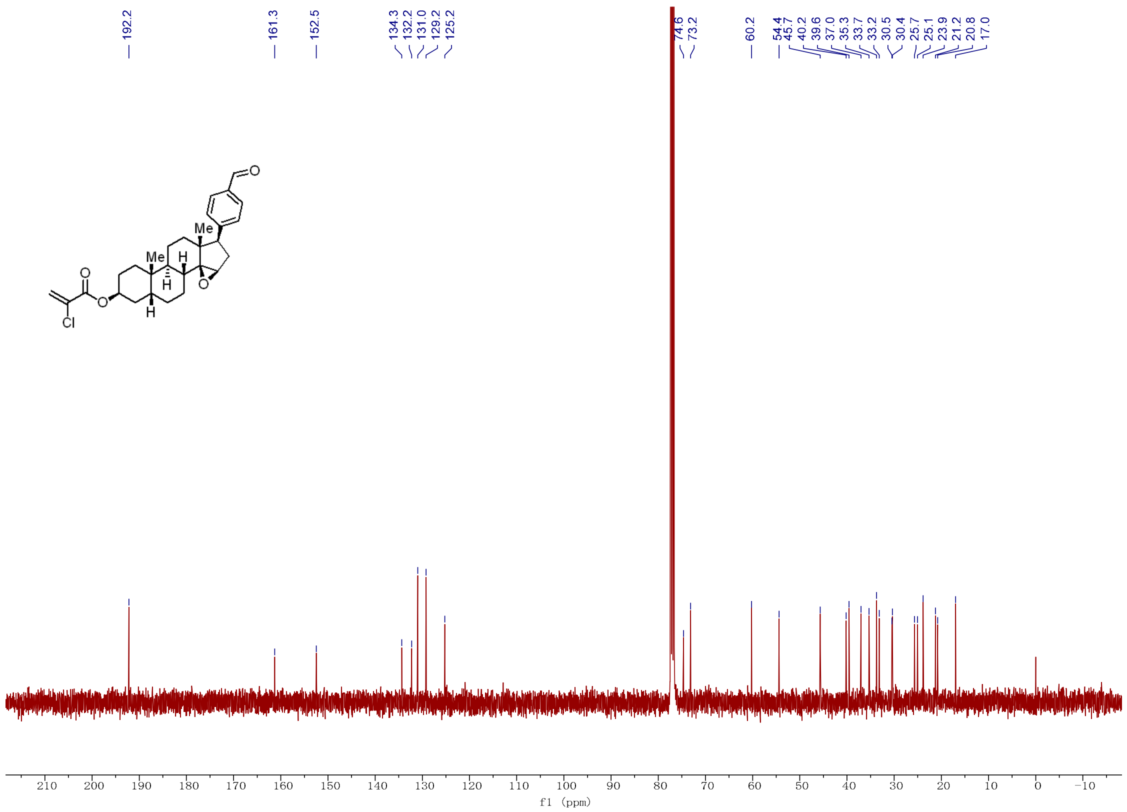

^{1}H NMR Spectrum of 2-5h (400 MHz, $CDCl_3$)

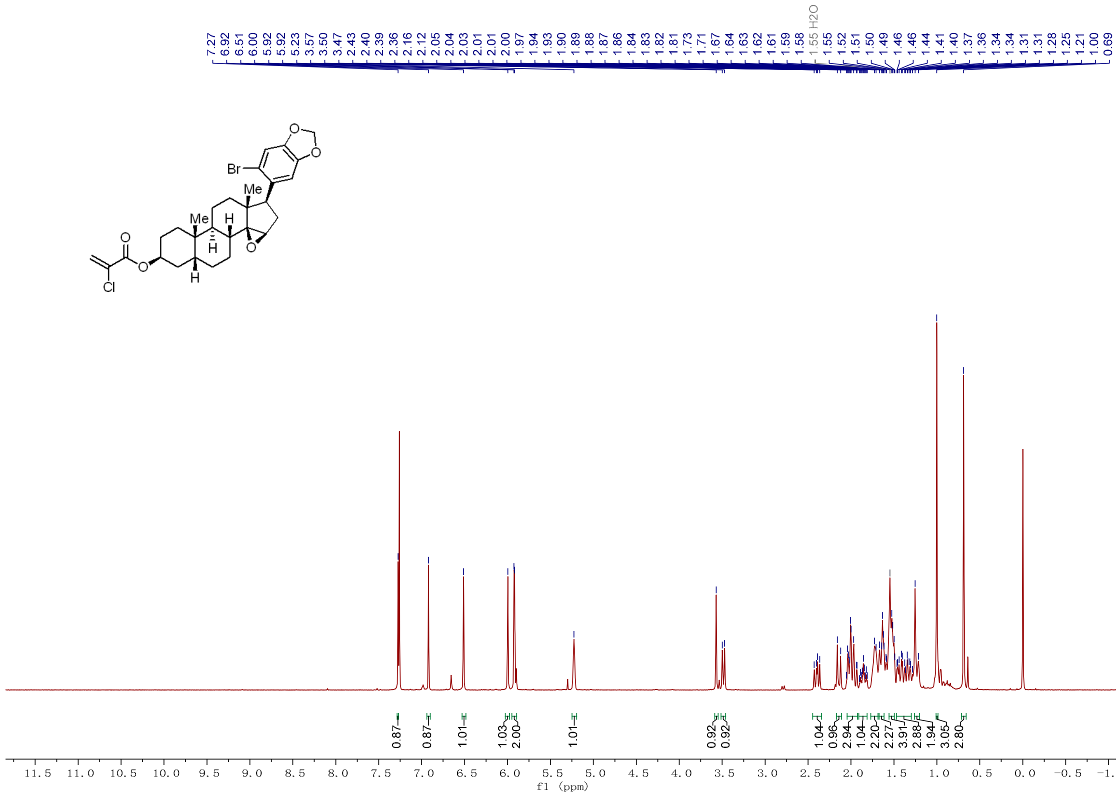

^{13}C NMR Spectrum of 2-5h (101 MHz, $CDCl_3$)

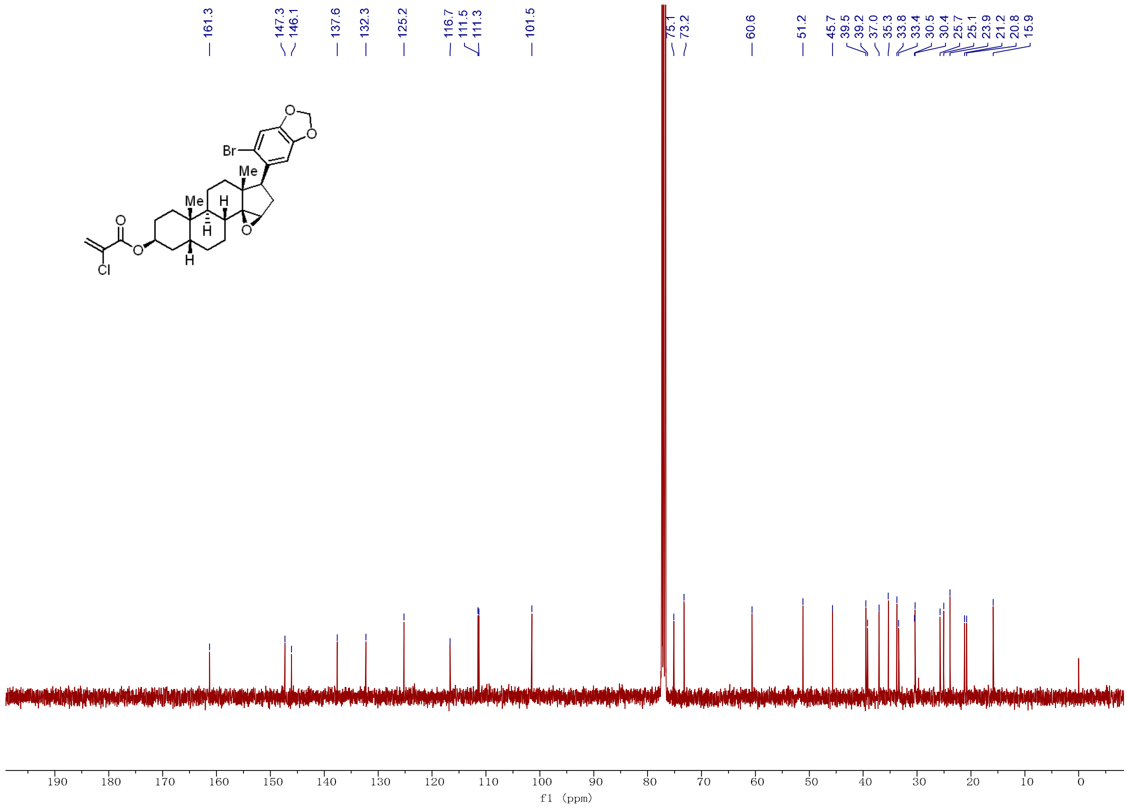

^{1}H NMR Spectrum of 2-5i (400 MHz, $CDCl_3$)

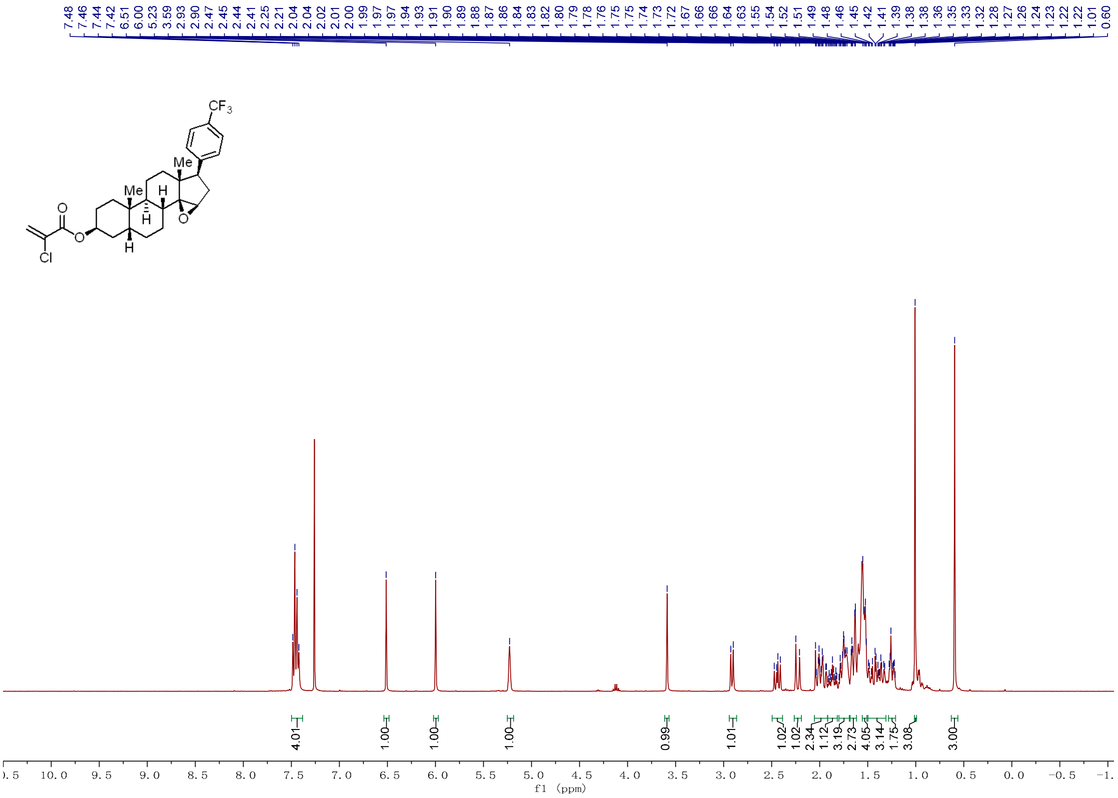

^{13}C NMR Spectrum of 2-5i (101 MHz, $CDCl_3$)

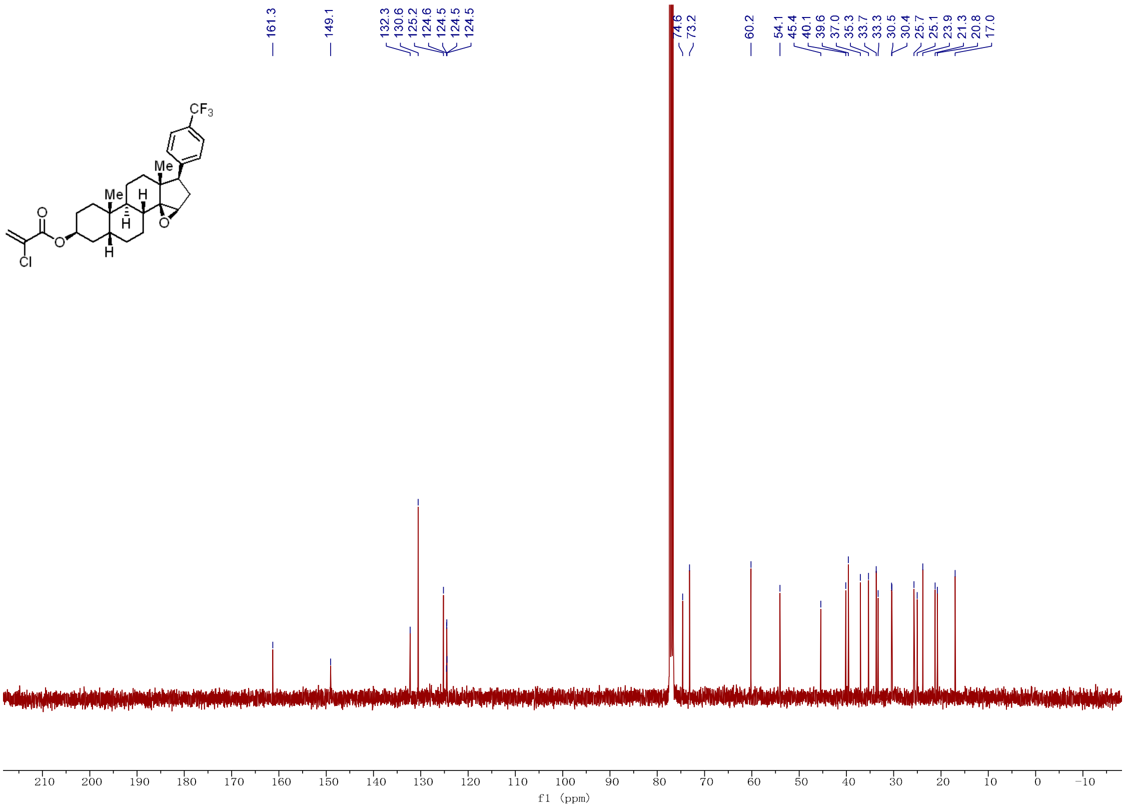

^{1}H NMR Spectrum of 2-5j (400 MHz, $CDCl_3$)

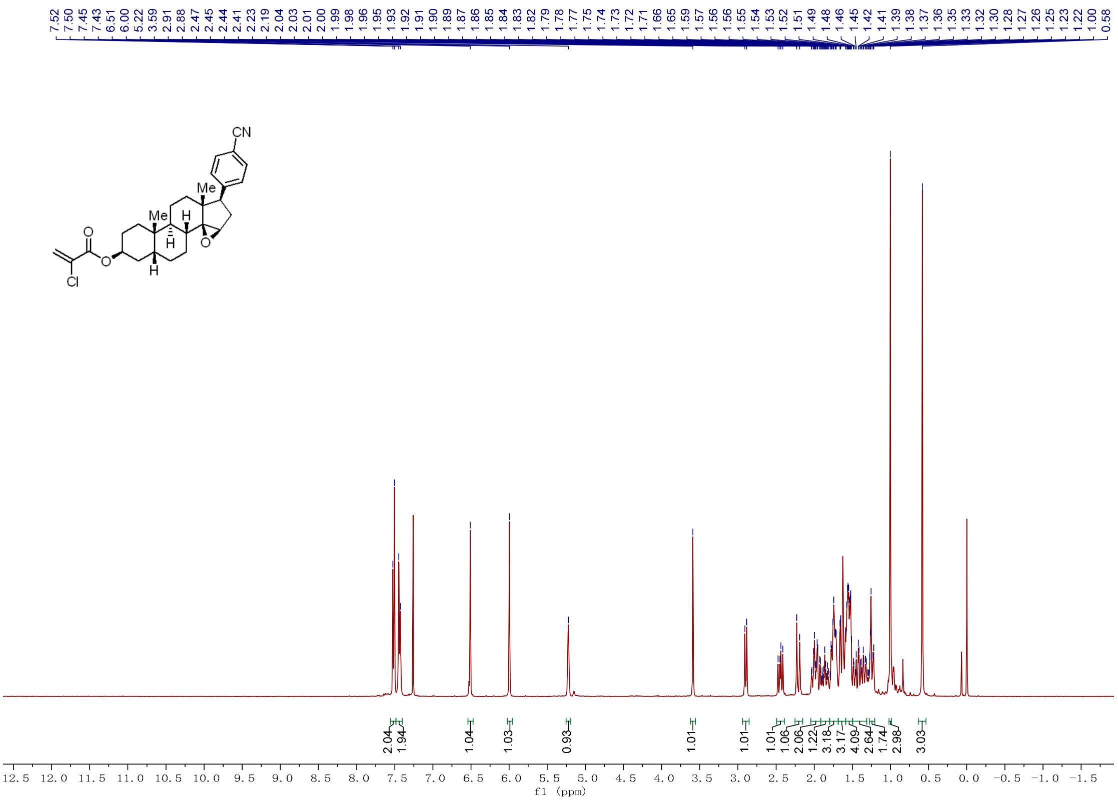

^{13}C NMR Spectrum of 2-5j (101 MHz, $CDCl_3$)

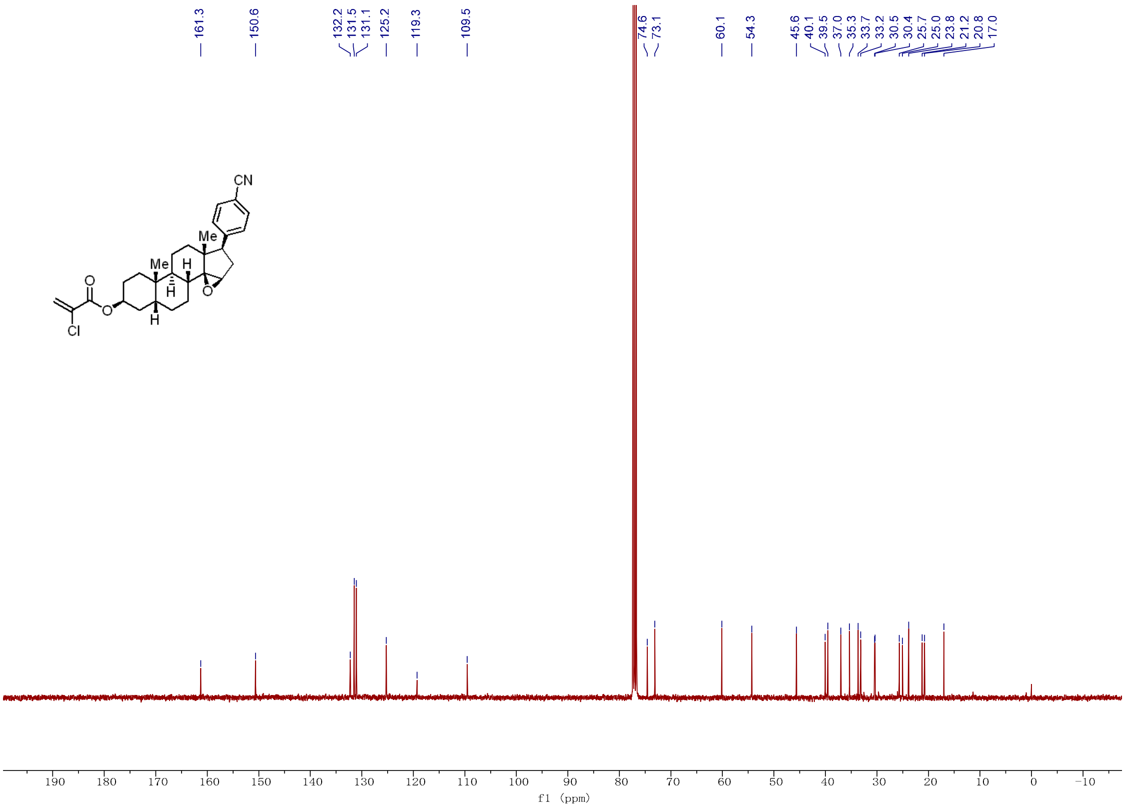

^{1}H NMR Spectrum of 2-5k (400 MHz, $CDCl_3$)

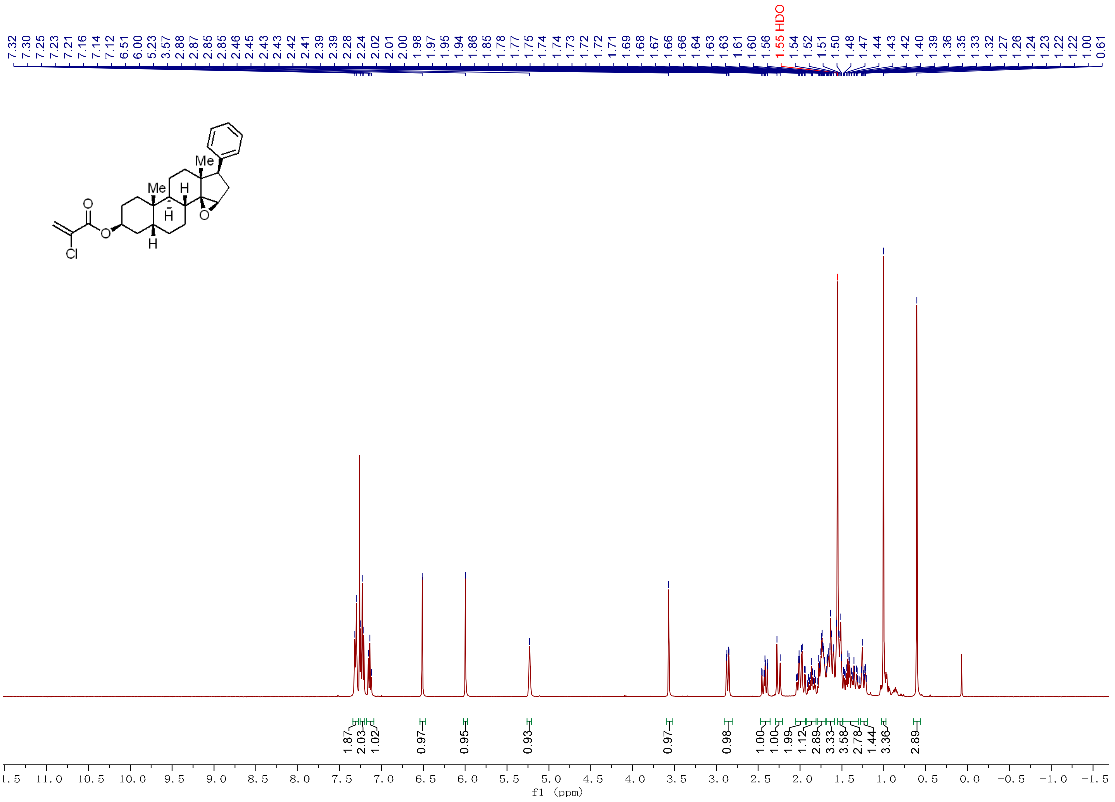

^{13}C NMR Spectrum of 2-5k (101 MHz, $CDCl_3$)

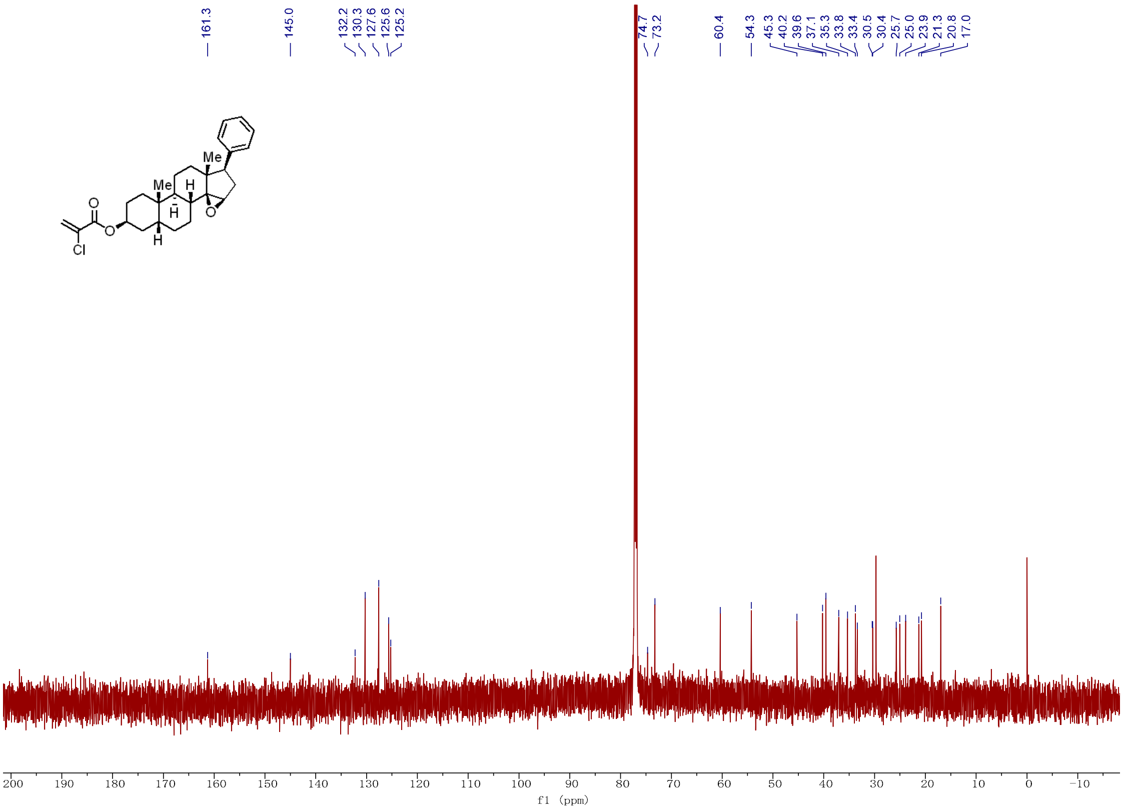

^{1}H NMR Spectrum of 2-5l (400 MHz, $CDCl_3$)

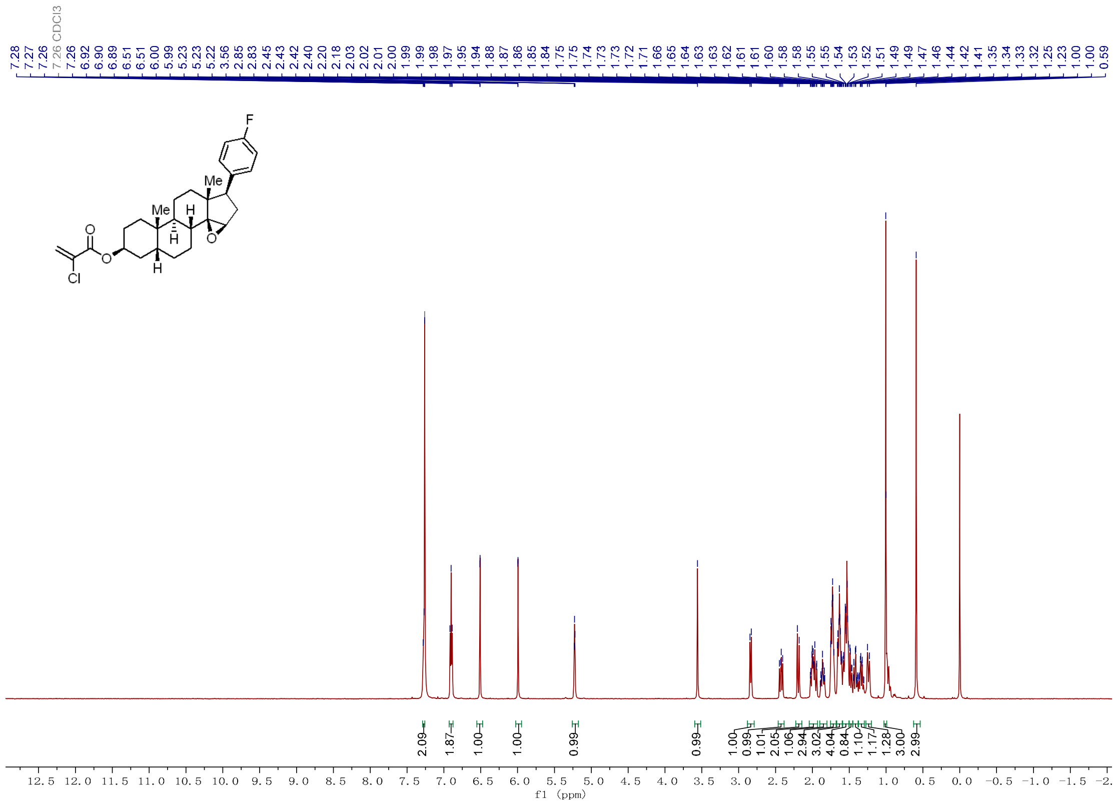

^{13}C NMR Spectrum of 2-5l (151 MHz, $CDCl_3$)

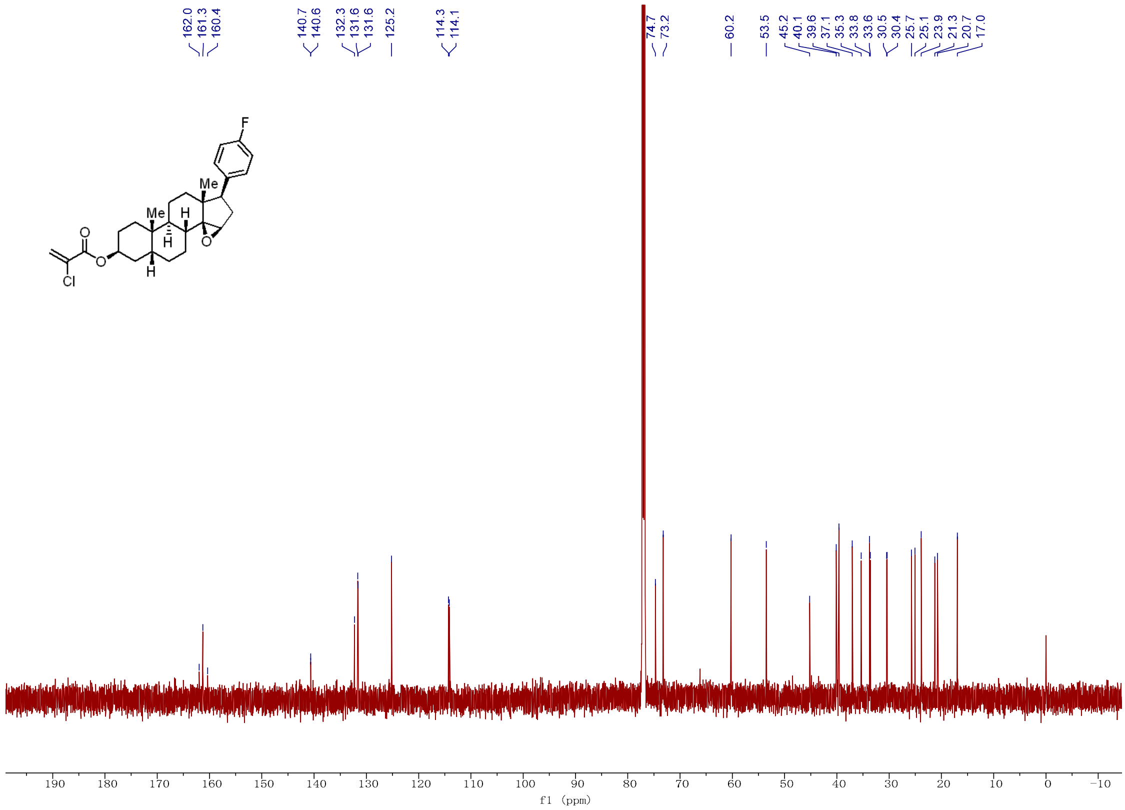

^{1}H NMR Spectrum of 2-6c (400 MHz, $CDCl_3$)

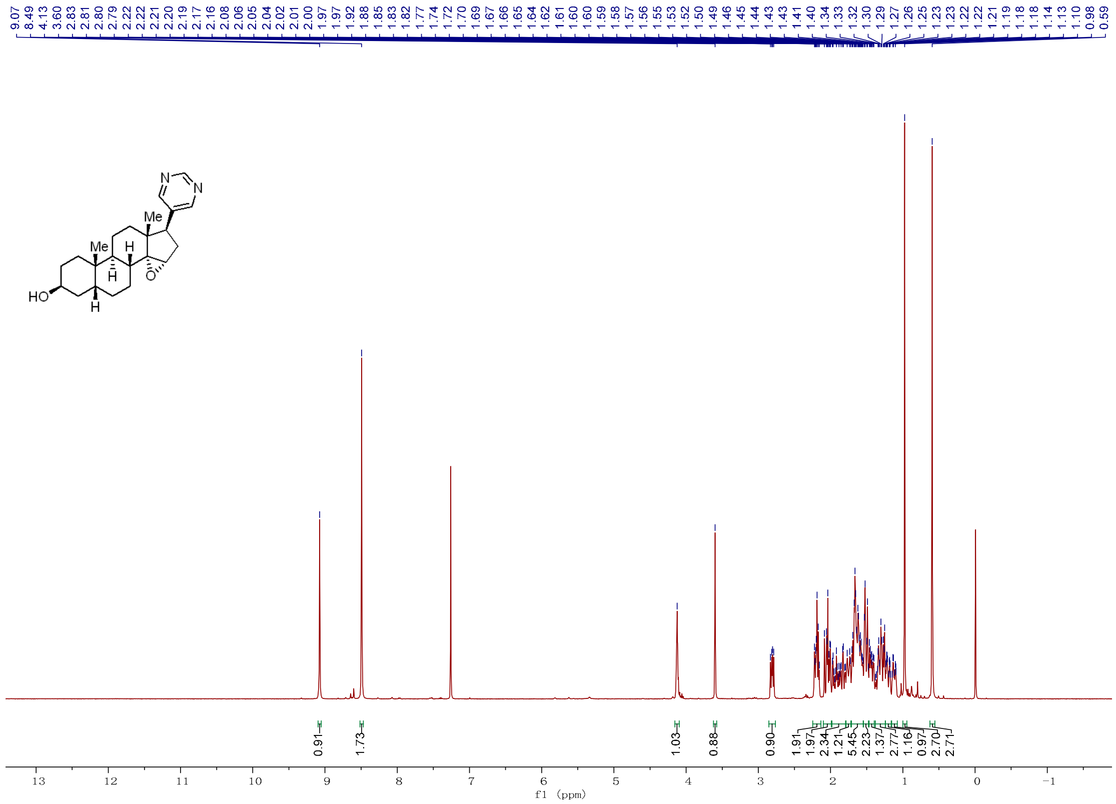

^{13}C NMR Spectrum of 2-6c (101 MHz, $CDCl_3$)

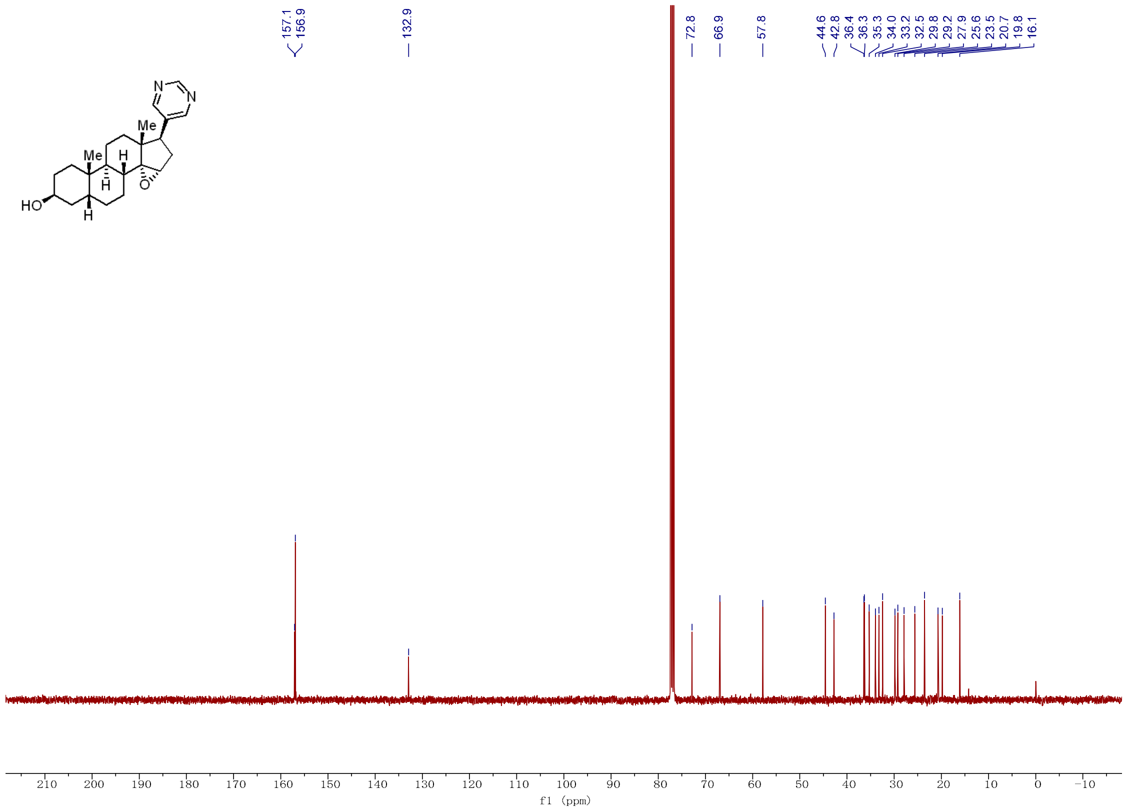

^{1}H NMR Spectrum of 2-7c (400 MHz, $CDCl_3$)

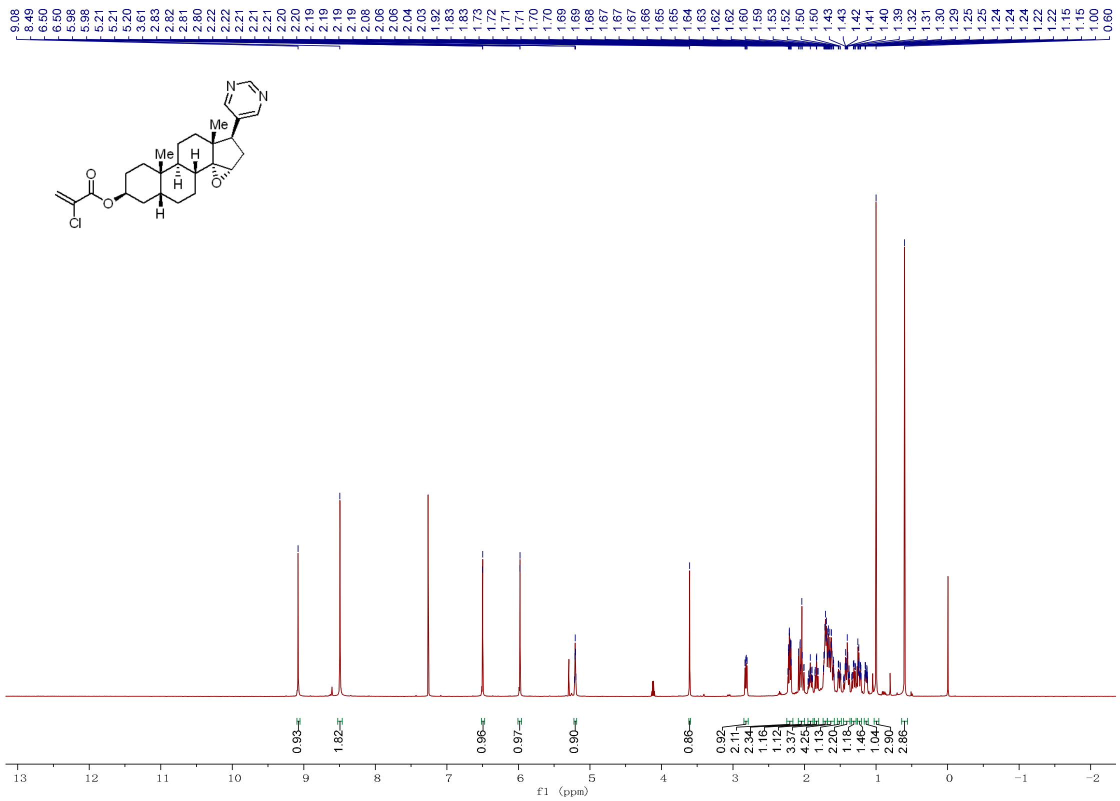

^{13}C NMR Spectrum of 2-7c (151 MHz, $CDCl_3$)

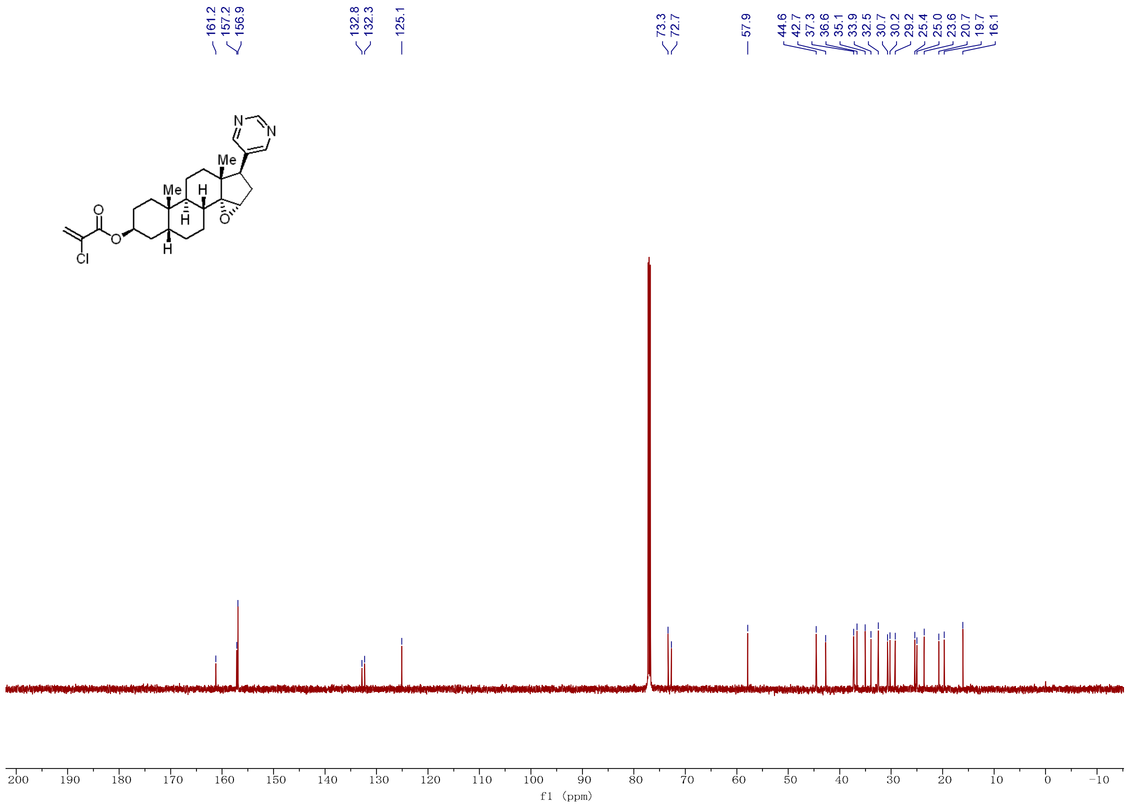

^{1}H NMR Spectrum of 2-8c (400 MHz, $CDCl_3$)

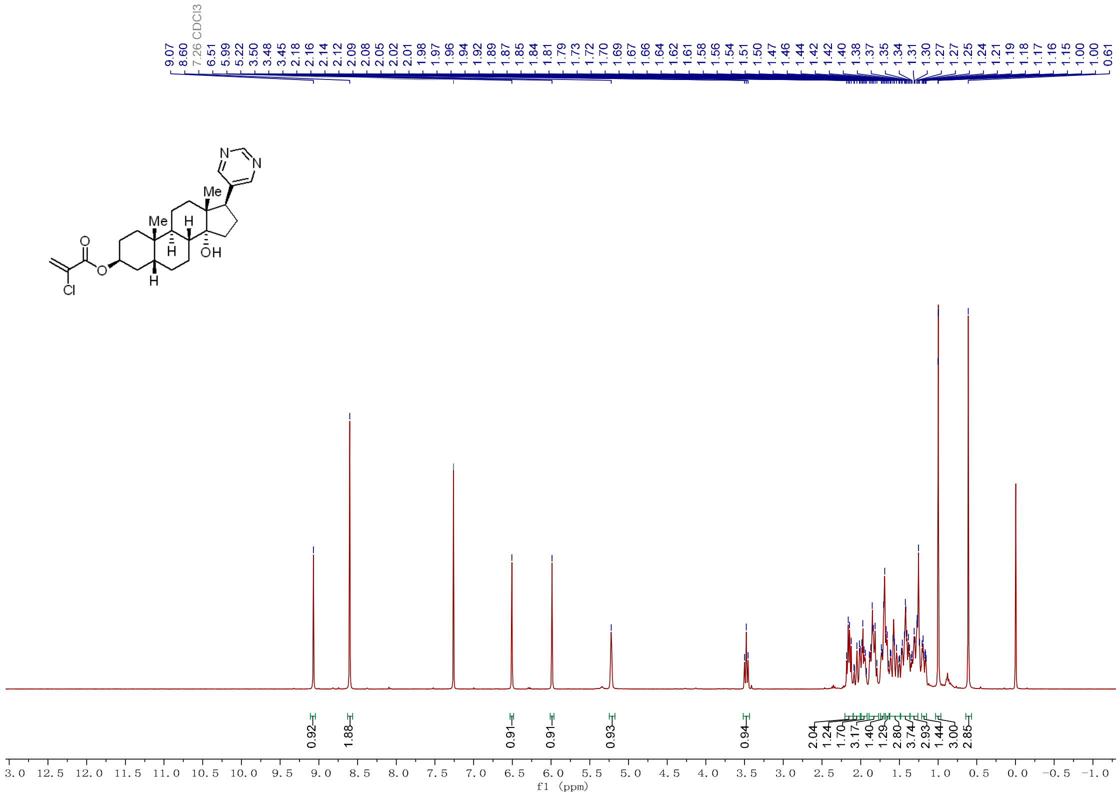

^{13}C NMR Spectrum of 2-8c (101 MHz, $CDCl_3$)

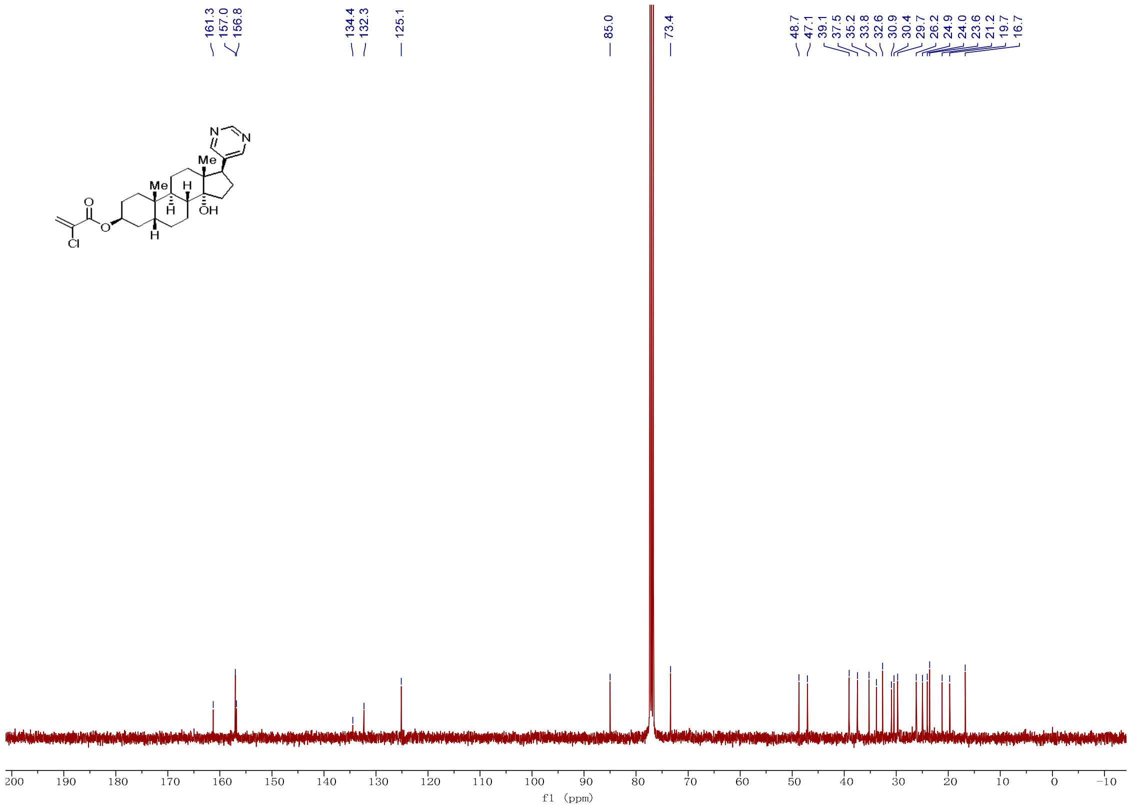

Supplement: Supplementary file 1 [file ijms-27-03326-s001.zip › ijms-4208549-supplementary.pdf]
